# Supplementary material for: Visualizing chaperonin function in situ by cryo-electron tomography
Source: Nature. 2024 Aug 21;633(8029):459–64. doi: 10.1038/s41586-024-07843-w (PMC11390479; doi:10.1038/s41586-024-07843-w)
Supplement: Supplementary file 1 — Classification of GroEL–GroES complexes following template matching. [file 41586_2024_7843_MOESM1_ESM.pdf]

nature portfolio

https://doi.org/10.1038/s41586-024-07843-w

Supplementary information

Visualizing chaperonin function in situ by cryo-electron tomography

In the format provided by the
authors and unedited

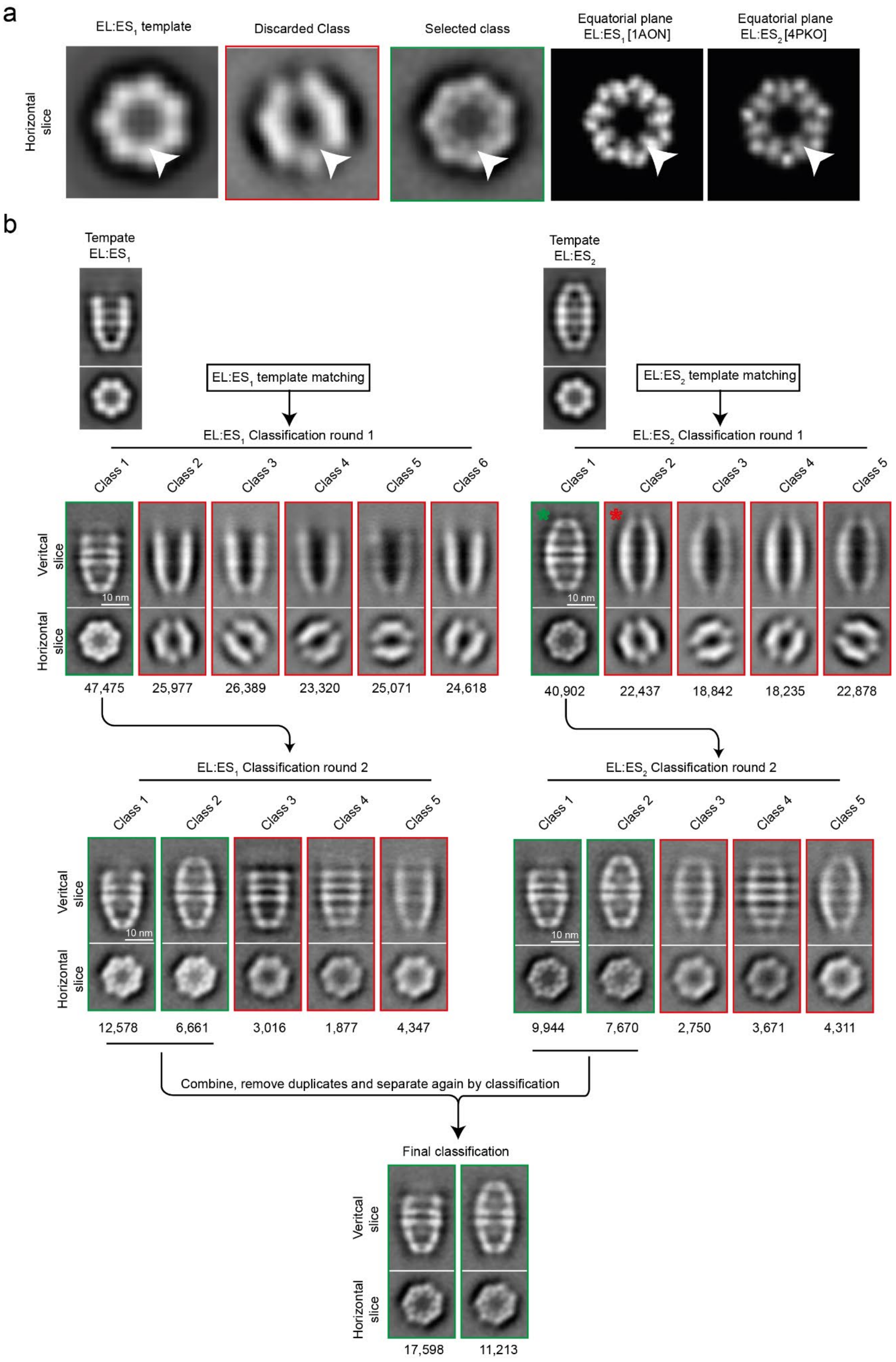
a
EL:ES1 template
Discarded Class
Selected class
Equatorial plane EL:ES1 [1AON]
Equatorial plane EL:ES2 [4PKO]
Horizontal slice
b
Tempate EL:ES1
Tempate EL:ES2
EL:ES1 template matching
EL:ES2 template matching
EL:ES1 Classification round 1
EL:ES2 Classification round 1
Class 1
Class 2
Class 3
Class 4
Class 5
Class 6
Veritcal slice
Horizontal slice
10 nm
47,475
25,977
26,389
23,320
25,071
24,618
40,902
22,437
18,842
18,235
22,878
EL:ES1 Classification round 2
EL:ES2 Classification round 2
12,578
6,661
3,016
1,877
4,347
9,944
7,670
2,750
3,671
4,311
Combine, remove duplicates and separate again by classification
Final classification
17,598
11,213

Supplementary Figure 1: Classification of GroEL:ES complexes after template matching. (a) Comparison of reference template, 3D classes and model crystal structures. From left to right are shown horizontal slices through the equatorial plane of the EL:ES_1 template, an example of a class that was discarded (EL:ES_1, round 1, class 2; see panel b, red asterisk), a class that was selected for further processing (EL:ES_1, round 1, class 1; see panel b, green asterisk) and density renderings of the EL:ES_1 [1AON] and EL:ES_2 [4PKO] crystal structures at the theoretical resolution limit of the cryo-ET data, 14.08 Å (the Nyquist frequency at the pixel size used for classification). The white arrowhead highlights the feature chosen as selection criterion for classes with true-positive particles, namely the bilobal appearance of the equatorial domains. We selected this feature, because in crystal and cryo-EM structures of GroEL/ES and bacterial chaperonins these rings were always the best-defined (i.e. most rigid, as judged from atomic B-factors) and most uniform region, while intermediate and apical domains can undergo extensive reorientations. This feature is visible in the exemplary selected class, but not in the reference template and the exemplary discarded class. (b) Detailed overview of the 3D classification workflow described in Methods and Extended Data Fig. 1b. The data-derived templates for the EL:ES_1 and EL:ES_2 classification arms are shown on top. Round 1 classification in Stopgap was performed without angular search and was repeated 5 times starting from different, random initial starting sets. Only particles with consistent class assignments throughout the repeats were retained for selection. Classes were manually selected according to the criterion described in a. The images show vertical and horizontal slices through the class averages. The numbers below are the particle numbers in the class. Classes selected for further processing are highlighted by a green outline, while classes that were discarded as false-positives are marked with a red outline. The classes shown in (a) are marked with a red/green asterisk. In round 2, local search with an angular sampling of 6° in 2° steps was performed and the classification was repeated 5 times as above. Only particles that ended up consistently in the same class in all independent runs were considered for further processing, as previously described [1]. After selection of true-positive classes, the respective particles were combined and duplicates removed. 9,944 (~57 %) and 6661 (~60 %) of the other particle class, respectively, were cross-identified in the EL:ES_1 and EL:ES_2 classification branches.

1 Erdmann, P. S. *et al.* In situ cryo-electron tomography reveals gradient organization of ribosome biogenesis in intact nucleoli. *Nat Commun* **12**, 5364 (2021). https://doi.org:10.1038/s41467-021-25413-w
