# Supplementary material for: Visualizing chaperonin function in situ by cryo-electron tomography
Source: Nature. 2024 Aug 21;633(8029):459–64. doi: 10.1038/s41586-024-07843-w (PMC11390479; doi:10.1038/s41586-024-07843-w)
Supplement: Supplementary file 2 — Reporting Summary [file 41586_2024_7843_MOESM2_ESM.pdf]

nature portfolio

Corresponding author(s): F. Ulrich Hartl
Wolfgang Baumeister
Ruben Fernandez-Busnadiego

Last updated by author(s): Jul 16, 2024

Reporting Summary

Nature Portfolio wishes to improve the reproducibility of the work that we publish. This form provides structure for consistency and transparency in reporting. For further information on Nature Portfolio policies, see our Editorial Policies and the Editorial Policy Checklist.

Statistics

For all statistical analyses, confirm that the following items are present in the figure legend, table legend, main text, or Methods section.

n/a	Confirmed	
☐	☒	The exact sample size (*n*) for each experimental group/condition, given as a discrete number and unit of measurement
☐	☒	A statement on whether measurements were taken from distinct samples or whether the same sample was measured repeatedly
☐	☒	The statistical test(s) used AND whether they are one- or two-sided<br>*Only common tests should be described solely by name; describe more complex techniques in the Methods section.*
☒	☐	A description of all covariates tested
☒	☐	A description of any assumptions or corrections, such as tests of normality and adjustment for multiple comparisons
☐	☒	A full description of the statistical parameters including central tendency (e.g. means) or other basic estimates (e.g. regression coefficient) AND variation (e.g. standard deviation) or associated estimates of uncertainty (e.g. confidence intervals)
☐	☒	For null hypothesis testing, the test statistic (e.g. *F*, *t*, *r*) with confidence intervals, effect sizes, degrees of freedom and *P* value noted<br>*Give P values as exact values whenever suitable.*
☒	☐	For Bayesian analysis, information on the choice of priors and Markov chain Monte Carlo settings
☒	☐	For hierarchical and complex designs, identification of the appropriate level for tests and full reporting of outcomes
☒	☐	Estimates of effect sizes (e.g. Cohen's *d*, Pearson's *r*), indicating how they were calculated

Our web collection on statistics for biologists contains articles on many of the points above.

Software and code

Policy information about availability of computer code

Data collection	Tomograms of 37 °C, MetK, HS E. coli cells were recorded on FEI Titan Krios transmission electron microscopes with SerialEM 3.9.056 software. Tomograms of EL+ E. coli cells were collected on a FEI Titan Krios transmission electron microscope with TEM Tomography 5 software (Thermo Fisher Scientific). CryoEM data for single particle averaging were recorded on a FEI Titan Krios transmission electron microscope with SerialEM software. On-the-fly image processing and contrast transfer function (CTF) refinement of the cryo-EM micrographs were carried out using the Focus 1.1 software package.<br>Mass spectrometry data were aquired on a Q-Exactive HF mass spectrometer (Thermo) with Xcalibur 4.0 and Q-Exactive HF-Orbitrap MS 2.7 software.
Data analysis	Cryo Electron Microscopy data was analysed using crYOLO 1.5.3, RELION 3.1.3 and CryoSPARC 2.0-3.3.2 as well as CryoDRGN 0.3.1-1.0.x. For display and initial analysis ChimeraX 1.4 and Chimera 1.13.1 was used. Model building was performed using Coot 0.9.4.1. Real-space refinement of molecular models was performed with Phenix 1.19.2.<br>Electron tomography data was analyzed with a combination of the following software packages: TOMOMAN 0.6, CTFfind 4.1.14, Stopgap 0.7.1, NovaCTF, MotionCor2 1.4.0, IMOD 4.11.1, Amira 2021.2, TomoSegMemTV 1.0, Relion 3.0.8, Warp 1.0.9 and M 1.0.9 Visualisation was performed with either IMOD 4.11.1 or ChimeraX 1.4.<br>Mass spectrometry data were analysed with MaxQuant 2.2.0.0.<br>Statistical analysis was performed with the R Studio 4.4.1 software package.

For manuscripts utilizing custom algorithms or software that are central to the research but not yet described in published literature, software must be made available to editors and reviewers. We strongly encourage code deposition in a community repository (e.g. GitHub). See the Nature Portfolio guidelines for submitting code & software for further information.

Data

Policy information about availability of data

All manuscripts must include a data availability statement. This statement should provide the following information, where applicable:

- Accession codes, unique identifiers, or web links for publicly available datasets
- A description of any restrictions on data availability
- For clinical datasets or third party data, please ensure that the statement adheres to our policy

The mass spectrometry data have been deposited to the ProteomeXchange Consortium via the PRIDE partner repository with the dataset identifier PXD042587 (https://www.ebi.ac.uk/pride/archive/projects/PXD042587). Model coordinates and electron density maps have been deposited to the wwPDB database under PDB/EMDB accession codes 8P4M/EMD-17418 (empty GroEL:ES chamber), 8P4N/EMD-17420 (GroEL:ES chamber with no or disordered MetK), 8P4O/EMD-17421/EMD-17422 (GroEL:ES chamber with ordered MetK), 8QXS/EMD-18735 (EL:ES1:MetK wide), 8QXT/EMD-18736 (EL:ES1:MetK narrow), 8P4R/EMD-17426 (in situ EL:ES2), 8QXU/EMD-18737 (in situ EL:ES1 wide), 8QXV/EMD-18738 (in situ EL:ES1 narrow) and 8P4P/EMD-17425 (in situ EL), respectively. Primary electron density maps have been deposited to the wwPDB database under EMDB accession codes EMD-17423 (in vitro GroEL:ES chamber with no or disordered MetK), EMD-17424 (in vitro GroEL:ES chamber with ordered MetK), EMD-17534 (empty EL:ES2), EMD-17535 (empty EL:ES1), EMD-17559 (GroEL:ES chamber with no or disordered substrate), EMD-17560 (GroEL:ES chamber with encapsulated, ordered substrate), EMD-17561 (70S ribosomes in 37 °C, HS and MetK E. coli cells), EMD-17562 (70S ribosomes in EL+ E. coli cells), EMD-17563 (EL:ES1 with encapsulated ordered MetK), EMD-17564 (EL:ES1 with no or encapsulated disordered MetK), EMD-17565 (EL:ES2 with two chambers with no or disordered MetK), EMD-17566 (EL:ES2 with ordered MetK in one chamber and no or disordered MetK substrate in the other chamber) and EMD-17567/EMD-17568/EMD-17569/EMD-17570/EMD-17571/EMD-17572/EMD-17573 (Conformers 1-7 of EL-ES2 with two encapsulated, ordered MetK). Because of their large file sizes, original cryo-ET imaging data are available from the corresponding author upon request. Source data to Fig. 1c and 3c and to Extended Data Fig. 2c, 3, 4g and 5 are provided with this paper.

Research involving human participants, their data, or biological material

Policy information about studies with human participants or human data. See also policy information about sex, gender (identity/presentation), and sexual orientation and race, ethnicity and racism.

Reporting on sex and gender	Not applicable.
Reporting on race, ethnicity, or other socially relevant groupings	Not applicable.
Population characteristics	Not applicable.
Recruitment	Not applicable.
Ethics oversight	Not applicable.

Note that full information on the approval of the study protocol must also be provided in the manuscript.

Field-specific reporting

Please select the one below that is the best fit for your research. If you are not sure, read the appropriate sections before making your selection.

☒ Life sciences ☐ Behavioural & social sciences ☐ Ecological, evolutionary & environmental sciences

For a reference copy of the document with all sections, see nature.com/documents/nr-reporting-summary-flat.pdf

Life sciences study design

All studies must disclose on these points even when the disclosure is negative.

Sample size	The amount of tomographic data acquired and used in this study was limited by the availability of microscopy time. For 37°C, HS and MetK at least 3 different biological replicates with several tomograms from different cells were acquired and analysed. For MetK only two independent biological replicates were used for data collection on dozens of different cells.<br>For cryoET a set of 48, 58, 60 and 64 curated tomograms for 37 °C, HS, MetK and EL+ were analyzed.<br>One in vitro GroEL:GroES:MetK samples was used for cryo-ET, resulting in 20 tomograms.<br>Three liquid culture samples, each of 37 °C, HS and EL+ E. coli cells were used for growth analysis.<br>Three liquid culture samples, each of MetK, EL+/MetK and EL+/MetK(n.i.) E. coli cells were used for growth analysis.<br>Three liquid culture samples, each of 37 °C, HS, MetK and EL+ E. coli cells were used for SDS-PAGE analysis of GroEL, GroES and GAPDH, for mass-spectroscopic analysis of ribosomal proteins, GroEL, GroES and MetK, and for immunoprecipitation and SDS-PAGE analysis of GroEL and MetK.<br>To determine via mass-spectroscopic analysis the stoichiometry of GroEL and MetK in GroEL:GroES:MetK complexes that were reconstituted in vitro, two independent samples were analyzed.<br>For cryoEM single-particle averaging of GroEL:GroES and GroEL:GroES:MetK complexes, one cryo-EM grid sample each was used for data collection and analysis.

No sample size calculation was performed. At least three independent experiments were performed in all cases as per commonly accepted standards of the field and to enable statistical analysis.

Data exclusions

For cryoEM, micrographs with an ice thickness score < 1.05, drift 0.4 Å < x < 70 Å, refined defocus 0.5 µm < x < 5.5 µm, estimated CTF resolution < 6 Å were selected on the fly, resulting in a dataset of 8,945 micrographs for the GroEL:ES:MetK sample. After 2D classification, only classes with visible secondary strucuture and a high estimated resolution were retained.
For cryoET, tilt series showing reflections from non-vitreous ice were discarded immediately after collection. Tilt series that had low aligment scores (residual error > 0.8) were discarded after reconstruction in IMOD. Particles that did not repeatedly end up in classes showing clear intermediate resolution features were discarded after 3D classification. This resulted in a selection of 19,239 subtomograms out of 176,408 starting subtomograms after template matching for EL:ES1 and 17,614 subtomograms out of 125,860 for EL:ES2, respectively.

Replication

48, 58, 60 and 64 cryo-ET tomograms were used from 37 °C, MetK, HS and EL+ E. coli cells, respectively.
Biochemical experiments to establish growth rates, to quantify proteins by label-free mass spectrometry, to analyze protein expression by SDS-PAGE and Western blotting, and to test MetK association with GroEL by immune precipitation and label-free mass spectrometry were replicated 3 times. All attempts of replication were successful.

Randomization

Randomization did not apply to this study, because there was no assignment of data points to distinct group. The only relevant randomization is that of determining random half-sets of particles for resolution assessment in cryo-EM and cryo-ET reconstructions. Randomization in half sets for FSC determination was done internally in RELION, CryoSPARC and Stopgap 0.71, respectively.

Blinding

No blinding was applied since data collection and analysis were not strongly dependent on subjective interpretation of the data. The findings are supported by quantitative measurements and statistical analysis when relevant.

Reporting for specific materials, systems and methods

We require information from authors about some types of materials, experimental systems and methods used in many studies. Here, indicate whether each material, system or method listed is relevant to your study. If you are not sure if a list item applies to your research, read the appropriate section before selecting a response.

Materials & experimental systems

n/a	Involved in the study
☐	☒ Antibodies
☒	☐ Eukaryotic cell lines
☒	☐ Palaeontology and archaeology
☒	☐ Animals and other organisms
☒	☐ Clinical data
☒	☐ Dual use research of concern
☒	☐ Plants

Methods

n/a	Involved in the study
☒	☐ ChIP-seq
☒	☐ Flow cytometry
☒	☐ MRI-based neuroimaging

Antibodies

Antibodies used

The polyclonal antisera against GroEL (1:10,000), GroES (1:10,000), MetK (1:5000) and GAPDH (1:10,000) were produced in house. Rabbit antiserum against alpha-lactalbumin was a product of East Acres Biologicals, Southbridge, MA immunization service (1992). Anti-Rabbit IgG (whole molecule)–Peroxidase antibody produced in goat (Sigma-Aldrich A9169, 1:10,000).

Validation

Inhouse antibodies were validated by binding to purified protein using Western blot.
Western blot application of GroEL and MetK antisera was demonstrated in Kerner, Michael J., et al. "Proteome-wide analysis of chaperonin-dependent protein folding in Escherichia coli." Cell 122.2 (2005): 209-220.

Plants

Seed stocks

Report on the source of all seed stocks or other plant material used. If applicable, state the seed stock centre and catalogue number. If plant specimens were collected from the field, describe the collection location, date and sampling procedures.

Novel plant genotypes

Describe the methods by which all novel plant genotypes were produced. This includes those generated by transgenic approaches, gene editing, chemical/radiation-based mutagenesis and hybridization. For transgenic lines, describe the transformation method, the number of independent lines analyzed and the generation upon which experiments were performed. For gene-edited lines, describe the editor used, the endogenous sequence targeted for editing, the targeting guide RNA sequence (if applicable) and how the editor was applied.

Authentication

Describe any authentication procedures for each seed stock used or novel genotype generated. Describe any experiments used to assess the effect of a mutation and, where applicable, how potential secondary effects (e.g. second site T-DNA insertions, mosiacism, off-target gene editing) were examined.
