# Supplementary material for: Visualizing chaperonin function in situ by cryo-electron tomography
Source: Nature. 2024 Aug 21;633(8029):459–64. doi: 10.1038/s41586-024-07843-w (PMC11390479; doi:10.1038/s41586-024-07843-w)
Supplement: Supplementary file 4 — Validation reports for wwPDB and emDB deposition. [file 41586_2024_7843_MOESM4_ESM.zip › 2024-01-00698C-s4/val-report_pdb_8QXU_EMD-18737.pdf]

Full wwPDB EM Validation Report (i)

Oct 25, 2023 – 06:13 pm BST

PDB ID	:	8QXU
EMDB ID	:	EMD-18737
Title	:	In situ structure average of GroEL14-GroES7 complexes with wide GroEL7 trans ring conformation in Escherichia coli cytosol obtained by cryo electron tomography
Deposited on	:	2023-10-25
Resolution	:	13.50 Å(reported)
Based on initial models	:	8P4M, 1KP8

This wwPDB validation report is for manuscript review

For Manuscript Review

This is a Full wwPDB EM Validation Report.

This report is produced by the wwPDB biocuration pipeline after annotation of the structure.

We welcome your comments at *validation@mail.wwpdb.org*
A user guide is available at
https://www.wwpdb.org/validation/2017/EMValidationReportHelp
with specific help available everywhere you see the (i) symbol.

The types of validation reports are described at
http://www.wwpdb.org/validation/2017/FAQs#types.

The following versions of software and data (see references (i)) were used in the production of this report:

EMDB validation analysis	:	0.0.1.dev70
Mogul	:	1.8.4, CSD as541be (2020)
MolProbity	:	4.02b-467
buster-report	:	1.1.7 (2018)
Percentile statistics	:	20191225.v01 (using entries in the PDB archive December 25th 2019)

1 Overall quality at a glance (i)

The following experimental techniques were used to determine the structure:
ELECTRON MICROSCOPY

The reported resolution of this entry is 13.50 Å.

Percentile scores (ranging between 0-100) for global validation metrics of the entry are shown in the following graphic. The table shows the number of entries on which the scores are based.

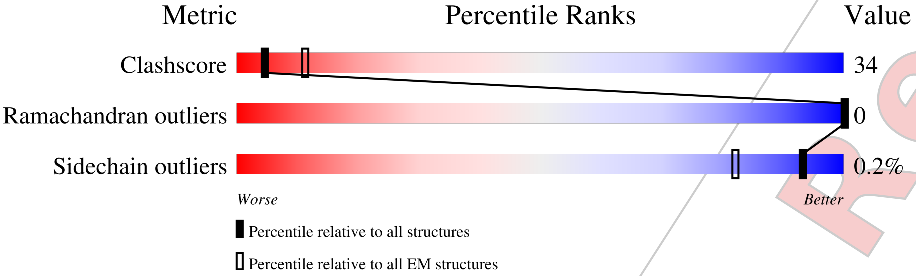

Metric	Whole archive (#Entries)	EM structures (#Entries)
Clashscore	158937	4297
Ramachandran outliers	154571	4023
Sidechain outliers	154315	3826

The table below summarises the geometric issues observed across the polymeric chains and their fit to the map. The red, orange, yellow and green segments of the bar indicate the fraction of residues that contain outliers for >=3, 2, 1 and 0 types of geometric quality criteria respectively. A grey segment represents the fraction of residues that are not modelled. The numeric value for each fraction is indicated below the corresponding segment, with a dot representing fractions <=5% The upper red bar (where present) indicates the fraction of residues that have poor fit to the EM map (all-atom inclusion < 40%). The numeric value is given above the bar.

Mol	Chain	Length	Quality of chain
1	A	547	43% 53% •
1	B	547	44% 52% •
1	C	547	44% 52% •
1	D	547	46% 50% •
1	E	547	45% 50% •

Continued on next page...

MapQ : 1.9.9
Ideal geometry (proteins) : Engh & Huber (2001)
Ideal geometry (DNA, RNA) : Parkinson et al. (1996)
Validation Pipeline (wwPDB-VP) : 2.36

For Manuscript Review

Continued from previous page...

Mol	Chain	Length	Quality of chain
1	F	547	44% 51% •
1	G	547	44% 52% •
1	H	547	43% 53% •
1	I	547	44% 52% •
1	J	547	41% 54% •
1	K	547	43% 53% •
1	L	547	43% 53% •
1	M	547	42% 54% •
1	N	547	44% 52% •
2	O	97	42% 56% •
2	P	97	40% 58% •
2	Q	97	43% 55% •
2	R	97	43% 55% •
2	S	97	44% 54% •
2	T	97	43% 55% •
2	U	97	42% 56% •

For Manuscript Review

2 Entry composition (i)

There are 7 unique types of molecules in this entry. The entry contains 59458 atoms, of which 0 are hydrogens and 0 are deuteriums.

In the tables below, the AltConf column contains the number of residues with at least one atom in alternate conformation and the Trace column contains the number of residues modelled with at most 2 atoms.

- Molecule 1 is a protein called Chaperonin GroEL.

Mol	Chain	Residues	Atoms					AltConf	Trace
			Total	C	N	O	S		
1	A	524	3851	2395	665	771	20	0	0
1	B	524	3851	2395	665	771	20	0	0
1	C	524	3851	2395	665	771	20	0	0
1	D	524	3851	2395	665	771	20	0	0
1	E	524	3851	2395	665	771	20	0	0
1	F	524	3851	2395	665	771	20	0	0
1	G	524	3851	2395	665	771	20	0	0
1	H	525	3864	2403	667	774	20	0	0
1	I	525	3864	2403	667	774	20	0	0
1	J	525	3864	2403	667	774	20	0	0
1	K	525	3864	2403	667	774	20	0	0
1	L	525	3864	2403	667	774	20	0	0
1	M	525	3864	2403	667	774	20	0	0
1	N	525	3864	2403	667	774	20	0	0

- Molecule 2 is a protein called Co-chaperonin GroES.

Mol	Chain	Residues	Atoms					AltConf	Trace
			Total	C	N	O	S		
2	O	95	687	430	125	131	1	0	0

Continued on next page...

For Manuscript Review

Continued from previous page...

Mol	Chain	Residues	Atoms	AltConf	Trace
2	P	95	Total C N O S<br>687 430 125 131 1	0	0
2	Q	95	Total C N O S<br>687 430 125 131 1	0	0
2	R	95	Total C N O S<br>687 430 125 131 1	0	0
2	S	95	Total C N O S<br>687 430 125 131 1	0	0
2	T	95	Total C N O S<br>687 430 125 131 1	0	0
2	U	95	Total C N O S<br>687 430 125 131 1	0	0

- Molecule 3 is ADENOSINE-5'-TRIPHOSPHATE (three-letter code: ATP) (formula: $C_{10}H_{16}N_5O_{13}P_3$).

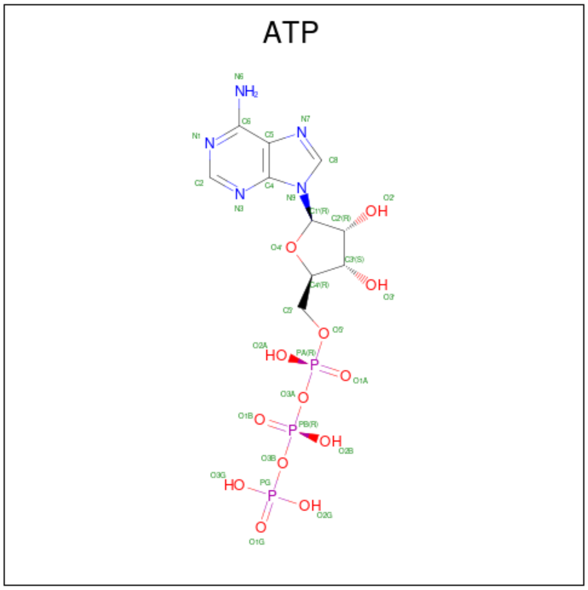

Mol	Chain	Residues	Atoms	AltConf
3	A	1	Total C N O P<br>31 10 5 13 3	0
3	B	1	Total C N O P<br>31 10 5 13 3	0
3	C	1	Total C N O P<br>31 10 5 13 3	0
3	D	1	Total C N O P<br>31 10 5 13 3	0
3	E	1	Total C N O P<br>31 10 5 13 3	0

Continued on next page...

For Manuscript Review

Continued from previous page...

Mol	Chain	Residues	Atoms					AltConf
			Total	C	N	O	P	
3	F	1	31	10	5	13	3	0
3	G	1	31	10	5	13	3	0

- Molecule 4 is MAGNESIUM ION (three-letter code: MG) (formula: Mg).

Mol	Chain	Residues	Atoms		AltConf
			Total	Mg	
4	A	1	1	1	0
4	B	1	1	1	0
4	C	1	1	1	0
4	D	1	1	1	0
4	E	1	1	1	0
4	F	1	1	1	0
4	G	1	1	1	0
4	H	1	1	1	0
4	I	1	1	1	0
4	J	1	1	1	0
4	K	1	1	1	0
4	L	1	1	1	0
4	M	1	1	1	0
4	N	1	1	1	0

- Molecule 5 is POTASSIUM ION (three-letter code: K) (formula: K).

Mol	Chain	Residues	Atoms		AltConf
			Total	K	
5	A	1	1	1	0

Continued on next page...

For Manuscript Review

Continued from previous page...

Mol	Chain	Residues	Atoms	AltConf
5	B	1	Total K<br>1 1	0
5	C	1	Total K<br>1 1	0
5	D	1	Total K<br>1 1	0
5	E	1	Total K<br>1 1	0
5	F	1	Total K<br>1 1	0
5	G	1	Total K<br>1 1	0
5	H	1	Total K<br>1 1	0
5	I	1	Total K<br>1 1	0
5	J	1	Total K<br>1 1	0
5	K	1	Total K<br>1 1	0
5	L	1	Total K<br>1 1	0
5	M	1	Total K<br>1 1	0
5	N	1	Total K<br>1 1	0

- Molecule 6 is ADENOSINE-5'-DIPHOSPHATE (three-letter code: ADP) (formula: $C_{10}H_{15}N_5O_{10}P_2$).

For Manuscript Review

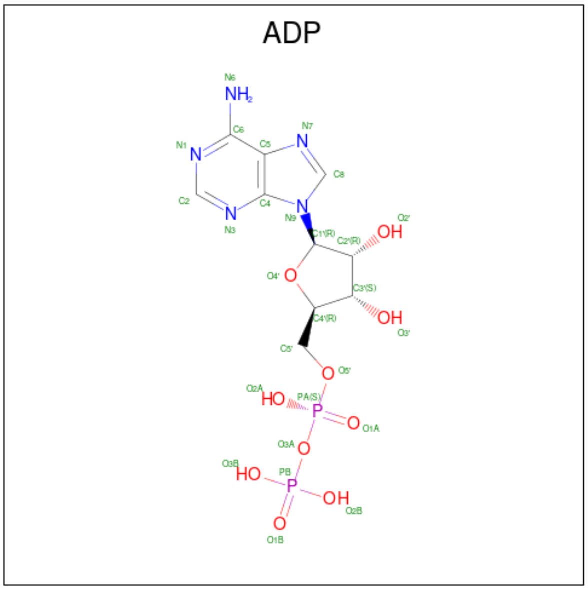

Mol	Chain	Residues	Atoms					AltConf
6	H	1	Total 27	C 10	N 5	O 10	P 2	0
6	I	1	Total 27	C 10	N 5	O 10	P 2	0
6	J	1	Total 27	C 10	N 5	O 10	P 2	0
6	K	1	Total 27	C 10	N 5	O 10	P 2	0
6	L	1	Total 27	C 10	N 5	O 10	P 2	0
6	M	1	Total 27	C 10	N 5	O 10	P 2	0
6	N	1	Total 27	C 10	N 5	O 10	P 2	0

- Molecule 7 is water.

Mol	Chain	Residues	Atoms		AltConf
7	A	30	Total 30	O 30	0
7	B	29	Total 29	O 29	0
7	C	28	Total 28	O 28	0
7	D	30	Total 30	O 30	0
7	E	29	Total 29	O 29	0

Continued on next page...

For Manuscript Review

Continued from previous page...

Mol	Chain	Residues	Atoms	AltConf
7	F	30	Total O<br>30 30	0
7	G	27	Total O<br>27 27	0
7	H	1	Total O<br>1 1	0
7	I	1	Total O<br>1 1	0
7	J	1	Total O<br>1 1	0
7	K	1	Total O<br>1 1	0
7	L	1	Total O<br>1 1	0
7	M	1	Total O<br>1 1	0
7	N	1	Total O<br>1 1	0

For Manuscript Review

3 Residue-property plots (i)

These plots are drawn for all protein, RNA, DNA and oligosaccharide chains in the entry. The first graphic for a chain summarises the proportions of the various outlier classes displayed in the second graphic. The second graphic shows the sequence view annotated by issues in geometry and atom inclusion in map density. Residues are color-coded according to the number of geometric quality criteria for which they contain at least one outlier: green = 0, yellow = 1, orange = 2 and red = 3 or more. A red diamond above a residue indicates a poor fit to the EM map for this residue (all-atom inclusion < 40%). Stretches of 2 or more consecutive residues without any outlier are shown as a green connector. Residues present in the sample, but not in the model, are shown in grey.

- Molecule 1: Chaperonin GroEL

Chain A:

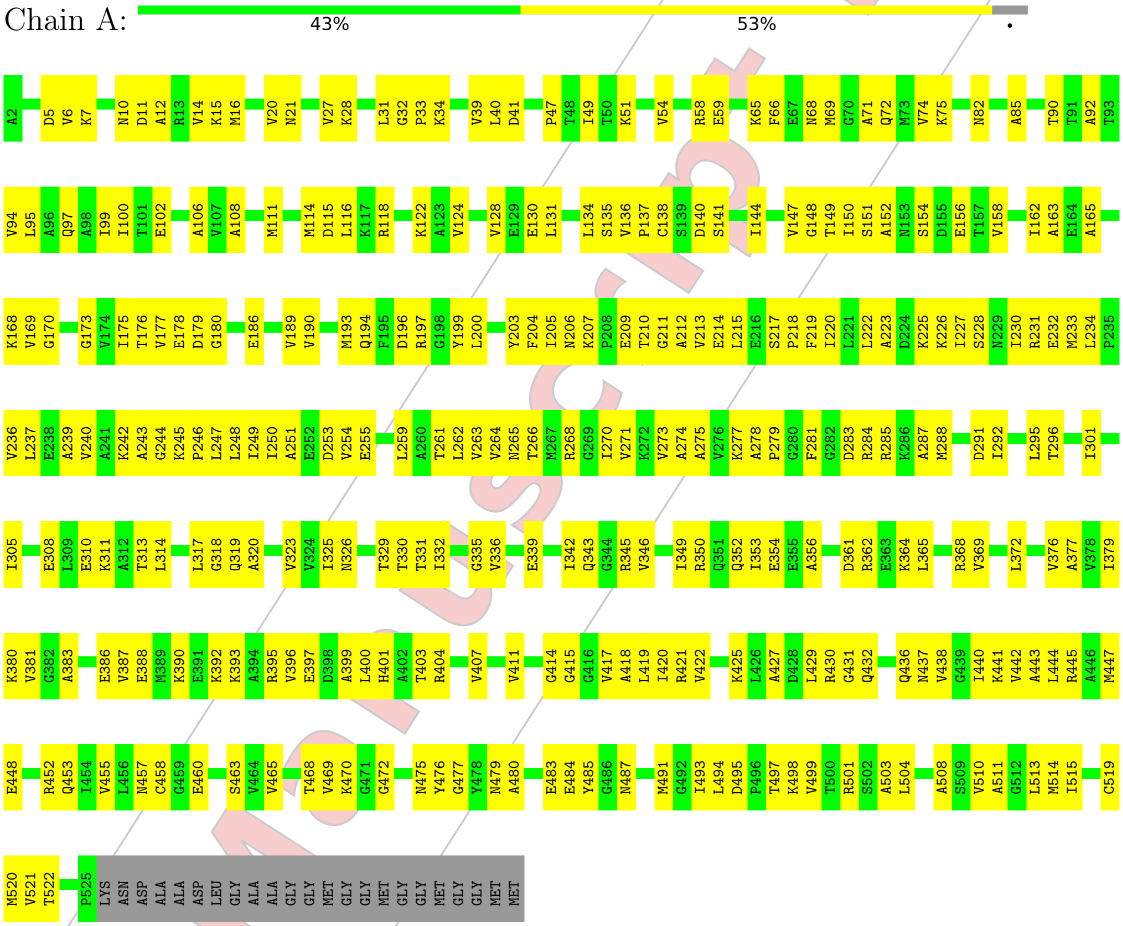

- Molecule 1: Chaperonin GroEL

Chain B:

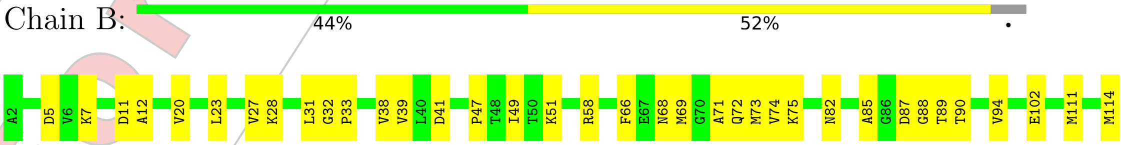

For Manuscript Review

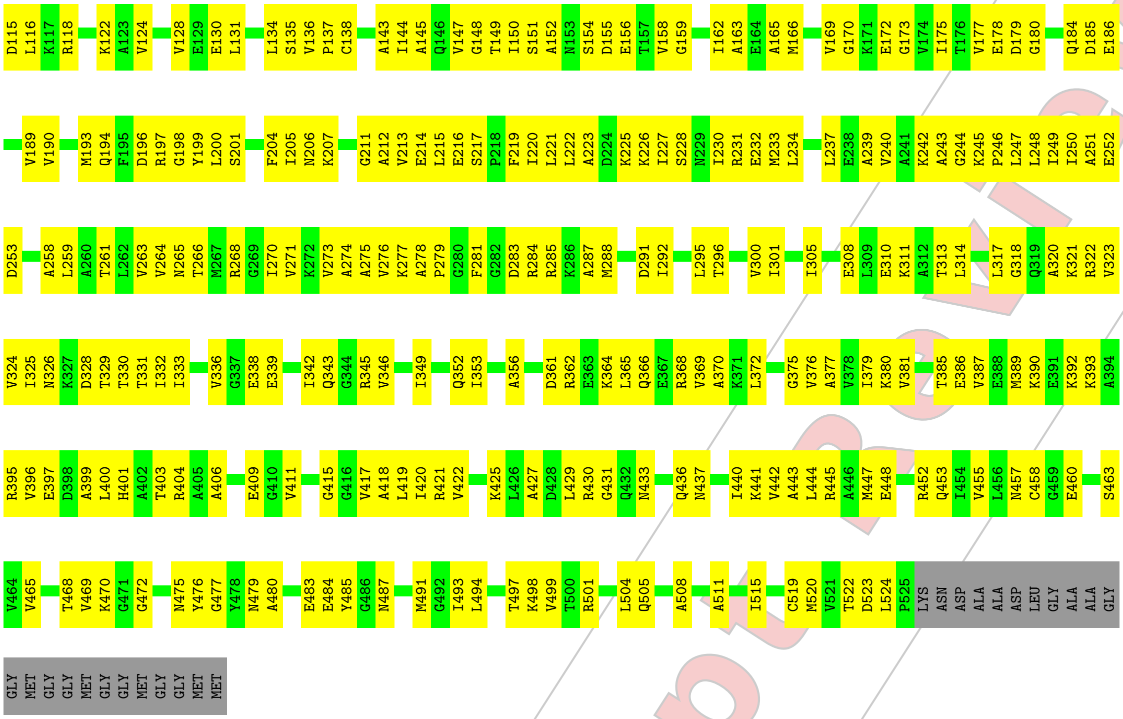

• Molecule 1: Chaperonin GroEL

Chain C:

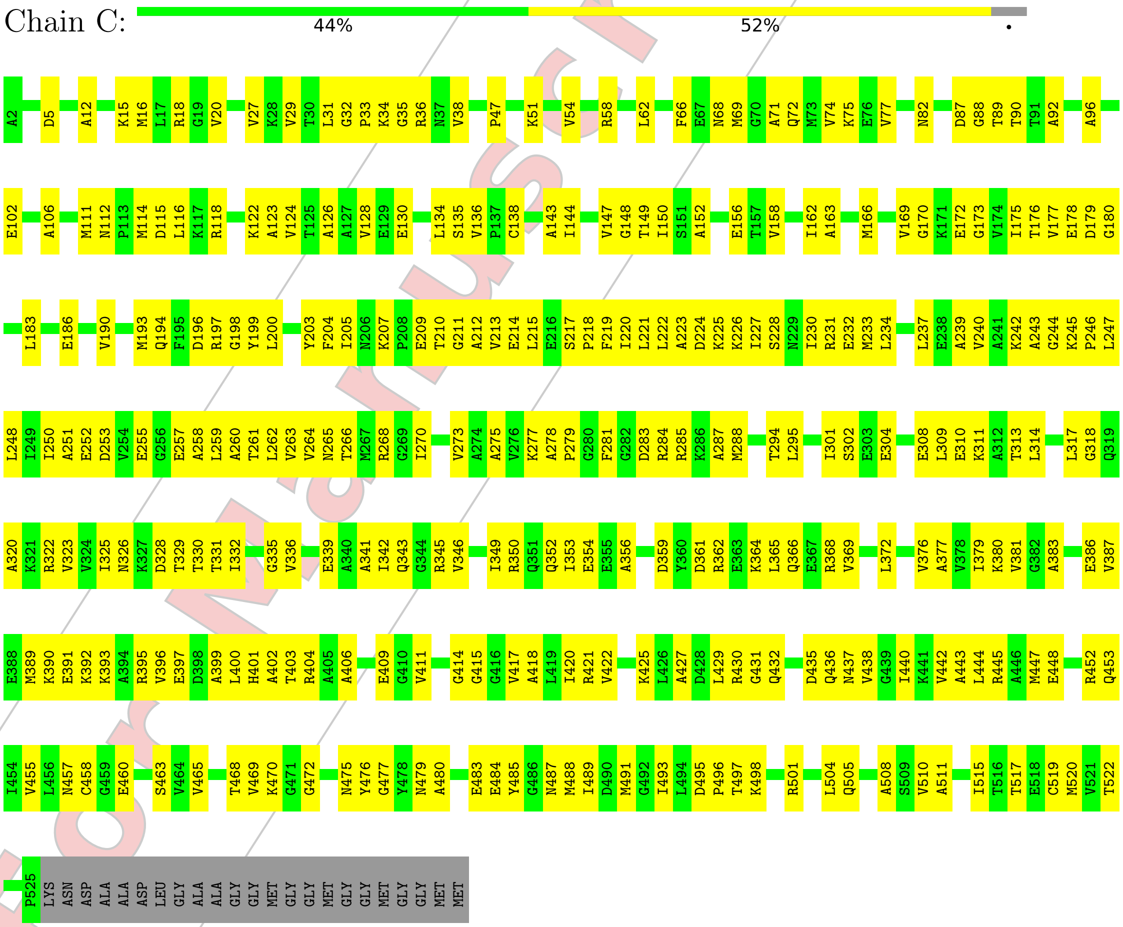

For Manuscript Review

• Molecule 1: Chaperonin GroEL

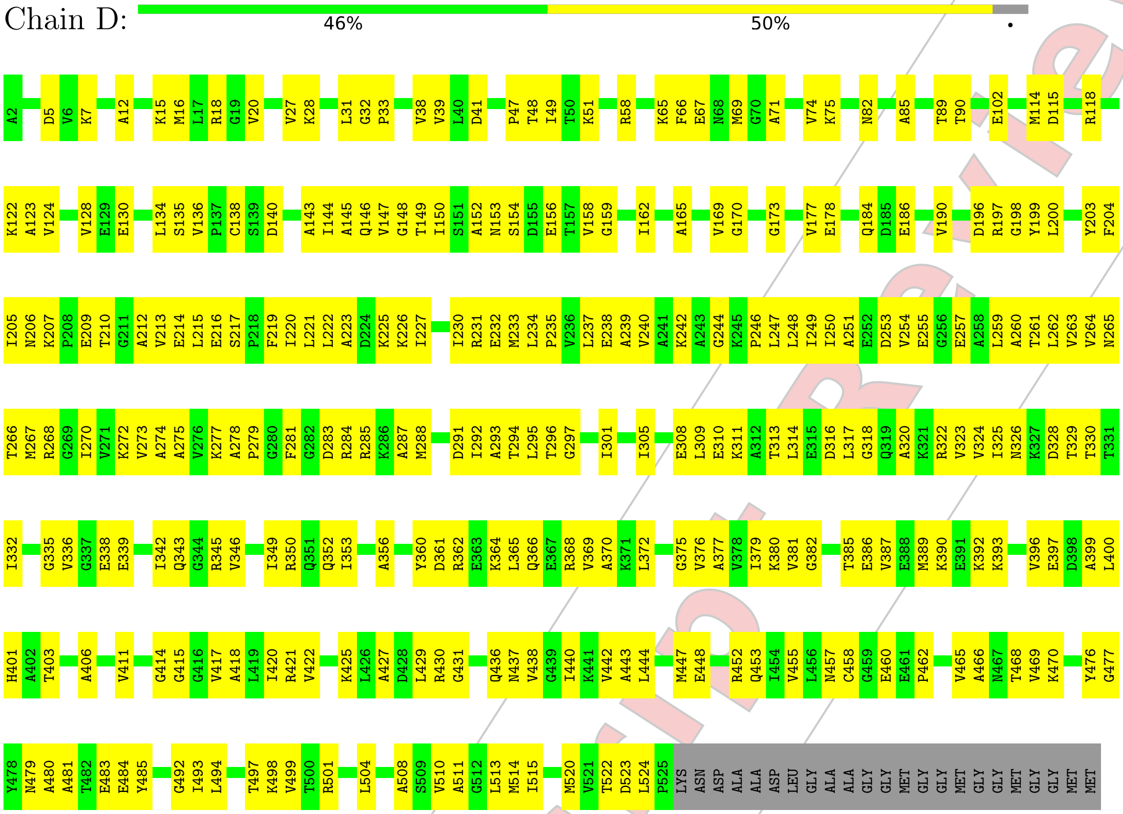

• Molecule 1: Chaperonin GroEL

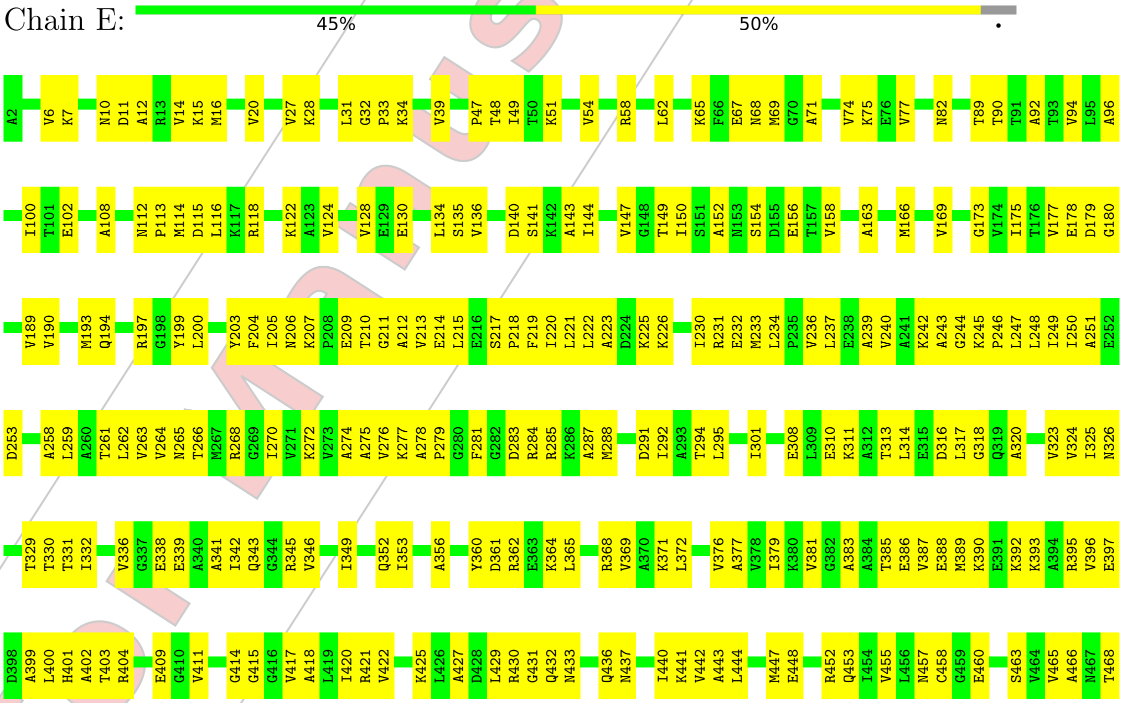

For Manuscript Review

V469 K470 G471 G472 N475 Y476 G477 Y478 N479 A480 E483 E484 Y485 G486 N487 M491 G492 I493 L494 D495 P496 T497 K498 R501 L504 Q505 A508 A511 G512 L513 M514 I515 C519 M520 V521 T522 D523 L524 P525 LYS ASN ASP ALA ALA ASP LEU GLY ALA ALA GLY GLY MET

GLY GLY MET GLY GLY MET GLY GLY MET MET

• Molecule 1: Chaperonin GroEL

Chain F: 44% 51% •

A2 D5 K15 M16 L17 R18 G19 V20 N21 V22 V27 K28 V29 T30 L31 G32 G35 V38 V39 L40 D41 P47 T48 I49 T50 K51 R58 E59 L62 F66 E67 N68 M69 G70 A71 Q72 M73 V74 K75 E76 V77 N82 T89 T90 T91 A92 T93 V94

Q97 E102 A108 N112 D115 L116 K117 R118 K122 A123 V124 T125 A126 A127 E130 L134 S135 V136 P137 C138 S139 D140 S141 I144 G148 T149 I150 S151 A152 N153 S154 D155 E156 T157 V158 L161 I162 A163 M166 D167 K168 V169 G173 V174 I175 T176 V177 E178

D179 G180 Q184 D185 E186 V189 V190 M193 Q194 F195 D196 R197 G198 Y199 L200 Y203 F204 I205 N206 K207 P208 E209 T210 G211 A212 V213 E214 L215 P218 F219 I220 L221 L222 A223 D224 K225 K226 I230 R231 E232 M233 L234 P235 V236 L237 E238 A239 V240 A241 K242 A243 G244 K245 P246

L247 L248 I249 I250 A251 E252 D253 V254 E255 L259 A260 T261 L262 V263 V264 N265 T266 M267 R268 G269 I270 V273 A274 A275 V276 K277 A278 P279 G280 F281 G282 D283 R284 R285 K286 A287 M288 D291 I292 A293 T294 L295 I301 E308 L309 E310 K311 A312 T313 L314 L317 G318 Q319 A320

K321 R322 V323 V324 I325 N326 K327 D328 T329 T330 T331 I332 G335 V336 E339 A340 A341 I342 Q343 G344 R345 V346 I349 R350 Q351 Q352 I353 A356 D359 Y360 D361 R362 E363 K364 L365 Q366 E367 R368 V369 L372 V376 A377 V378 I379 K380 V381 E386 V387 E388 M389 K390 E391

K392 K393 A394 R395 V396 E397 D398 A399 L400 H401 A402 T403 R404 V407 V411 V412 A413 G414 G415 G416 V417 A418 L419 I420 R421 V422 K425 L426 A427 D428 L429 R430 G431 Q432 Q436 N437 V438 G439 I440 K441 V442 A443 L444 R445 E448 L451 R452 Q453 I454 V455 L456 N457 C458 G459

E460 S463 V464 V465 T468 V469 K470 G471 G472 N475 Y476 G477 Y478 N479 A480 E483 E484 Y485 M488 M491 G492 I493 L494 K498 R501 S502 A503 L504 A508 S509 V510 A511 M514 I515 C519 M520 V521 T522 P525 LYS ASN ASP ALA ALA ASP LEU GLY ALA

ALA GLY GLY MET GLY GLY MET GLY GLY MET GLY GLY MET MET

• Molecule 1: Chaperonin GroEL

Chain G: 44% 52% •

A2 D5 D11 A12 R13 V14 K15 M16 V20 V27 K28 L31 G32 P33 V38 V39 P47 T48 I49 T50 K51 R58 E61 L62 E63 D64 K65 F66 E67 N68 M69 Q72 M73 V74 K75 E76 V77 N82 A85 T89 T90 T91 A92 T93 V94 L95 A96

E102 G103 L104 K105 A106 M111 N112 P113 M114 D115 R118 K122 A123 V124 V128 E129 E130 L134 S135 V136 P137 C138 S139 D140 A143 I144 V147 G148 T149 I150 S151 A152 N153 S154 D155 E156 T157 V158 I162 M166 V169 G173 V174 I175 T176 V177 E178 D179

G180 Q184 D185 E186 V189 V190 M193 Q194 F195 D196 R197 G198 Y199 L200 S201 P202 Y203 F204 I205 N206 K207 P208 E209 T210 G211 A212 V213 E214 L215 E216 S217 P218 F219 I220 L221 L222 A223 D224 K225 K226 I227 S228 N229 I230 R231 E232 M233 L234 L237 E238 A239 V240 A241 K242 A243 G244

K245 P246 L247 L248 I249 I250 A251 E255 A258 T261 L262 V263 V264 N265 T266 M267 R268 G269 I270 V273 A274 A275 V276 K277 A278 P279 G280 F281 G282 D283 R284 R285 K286 A287 M288 D291 I292 A293 T294 L295 I301 I305 L309 E310 T313 L314 E315 D316 L317 G318 Q319

A320 K321 R322 V323 V324 I325 N326 T329 T330 T331 I332 I333 D334 G335 V336 E339 A340 A341 I342 Q343 G344 R345 V346 A347 Q348 I349 R350 Q351 Q352 I353 A356 D361 R362 E363 K364 L365 R368 V369 A370 K371 L372 G375 V376 A377 V378 I379 K380 V381 T385 K392 K393 A394

R395 V396 E397 D398 A399 L400 H401 A402 T403 R404 A405 A406 E409 G410 V411 G414 G415 G416 V417 A418 L419 I420 R421 V422 K425 L426 A427 D428 L429 R430 G431 Q432 N433 Q436 N437 V438 G439 I440 K441 V442 A443 L444 R445 A446 M447 E448 R452 Q453 I454 V455 L456 N457 C458 G459 E460

S463 V464 V465 T468 V469 K470 G471 G472 Y476 G477 Y478 N479 A480 A481 T482 E483 E484 Y485 M488 M491 G492 I493 L494 D495 P496 T497 K498 V499 T500 R501 L504 A508 M514 I515 T516 T517 E518 C519 M520 V521 T522 D523 L524 P525 LYS ASN ASP ALA ALA ASP LEU GLY

ALA ALA GLY GLY MET GLY GLY MET GLY GLY MET GLY GLY MET MET

- Molecule 1: Chaperonin GroEL

Chain H: 43% 53% •

A2 A3 K4 D5 V6 K7 A12 R13 V14 K15 M16 L17 R18 G19 V20 V27 T30 L31 G32 P33 K34 G35 R36 N37 V38 V39 L40 D41 P47 T48 I49 T50 K51 A57 I60 E61 D64 E67 N68 M69 G70 A71 Q72 M73 V74 K75 E76 V77 K80 A81 N82

A85 G86 T89 T90 L95 I99 I100 T101 E102 G103 L104 A109 D115 L116 K117 R118 A123 V124 V128 K132 P137 C138 S139 D140 S141 K142 A143 I144 A145 Q146 T149 I150 S151 A152 N153 S154 D155 E156 L161 I162 A163 E164 A165 M166 D167 K168 V169 G170

K171 V174 I175 T176 V177 E178 D179 D185 E186 L187 D188 V189 V190 M193 Q194 F195 D196 R197 G198 Y199 L200 S201 P202 Y203 F204 I205 N206 K207 V213 E214 L215 E216 S217 P218 F219 I220 L221 L222 A223 D224 K225 K226 I227 I230 R231 E232 M233 L234 P235 V236 L237 V240 A241

K245 P246 L247 L248 I249 I250 A251 E252 D253 V254 L262 N265 T266 G269 I270 V271 K272 V273 A274 A275 V276 K277 A278 P279 G280 D283 R284 R285 K286 A287 M288 L289 Q290 D291 T294 L295 T296 V300 I301 S302 E303 M307 E308 L309 E310 L314 Q319 A320 K321 R322 V323

V324 I325 N326 K327 D328 T329 T330 T331 I332 I333 D334 G335 V336 E339 I342 R345 V346 A347 Q348 I349 R350 Q351 Q352 I353 E354 E355 A356 R362 E363 K364 L365 Q366 E367 R368 K371 L372 V376 A377 V378 I379 K380 V381 G382 A383 A384 K393 A394 R395 T403 R404 A405 A406

V407 E408 E409 G410 V411 V412 A413 G414 V417 A418 L419 I420 R421 A427 D428 L429 R430 G431 Q432 N433 E434 D435 Q436 N437 V438 G439 I440 K441 V442 A443 L444 M447 I454 V455 L456 N457 C458 G459 E460 E461 P462 S463 V464 V465 T468 V469 G472 N475 Y476 G477 Y478 N479 A480

E483 E484 Y485 G486 N487 M488 I493 L494 D495 P496 T497 K498 V499 T500 R501 S502 A503 L504 Q505 Y506 A507 A508 S509 V510 A511 M514 I515 C519 M520 V521 T522 D523 L524 P525 K526 ASN ASP ALA ALA ASP LEU GLY ALA ALA GLY GLY MET GLY GLY MET GLY GLY MET

MET

- Molecule 1: Chaperonin GroEL

Chain I: 44% 52% •

A2 A3 K4 D5 V6 K7 N10 D11 A12 R13 V14 K15 M16 L17 R18 G19 V20 V27 K28 V29 T30 L31 G32 P33 K34 G35 R36 N37 V38 V39 L40 D41 K42 P47 T48 I49 T50 K51 E59 I60 E61 L62 E63 D64 E67 N68 M69 G70 A71 Q72 M73 V74 K75 E76 V77

K80 A81 N82 A85 T89 T90 L95 A98 I99 E102 G103 L104 K105 A106 M111 M114 D115 L116 K117 R118 K122 A123 V124 A127 V128 K132 P137 D140 S141 I144 V147 G148 T149 I150 S151 A152 D155 E156 L161 I162 A163 E164 A165

For Manuscript Review

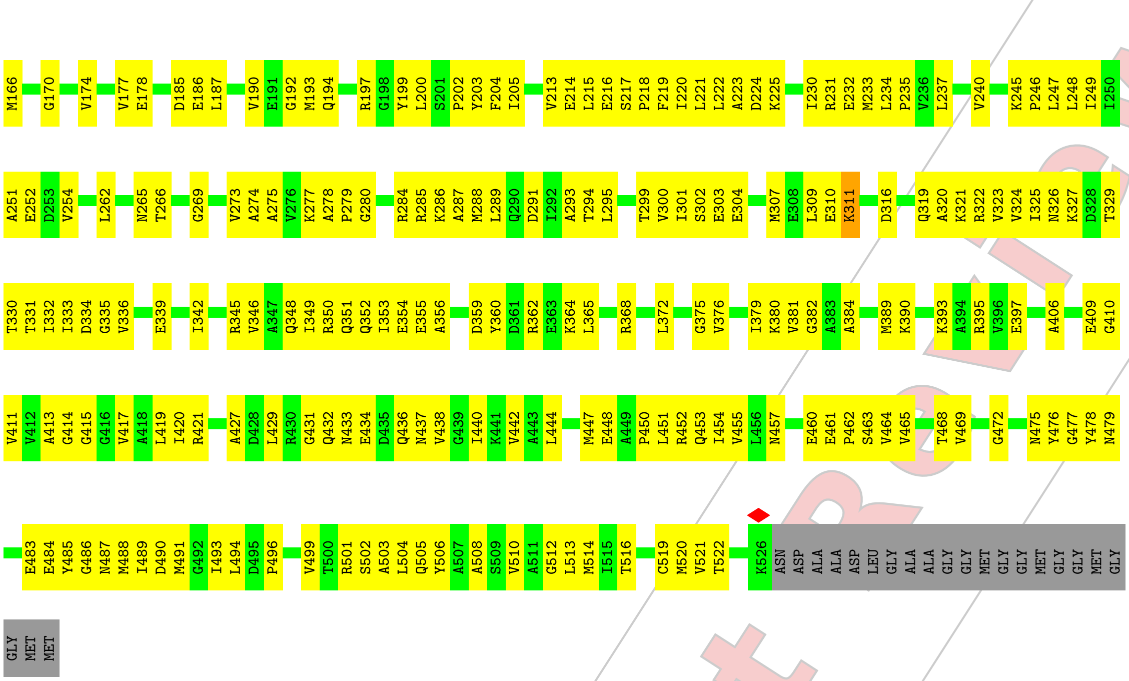

- Molecule 1: Chaperonin GroEL

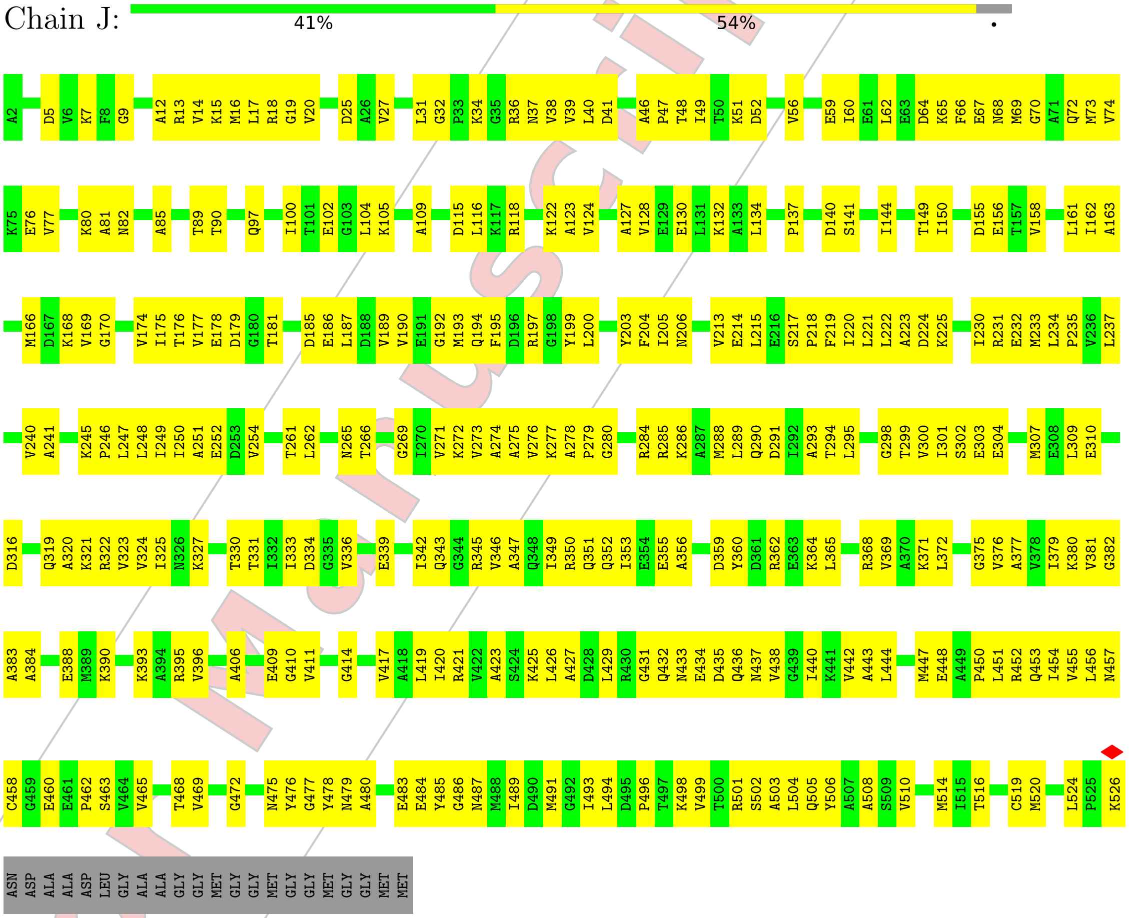

- Molecule 1: Chaperonin GroEL

For Manuscript Review

Chain K:

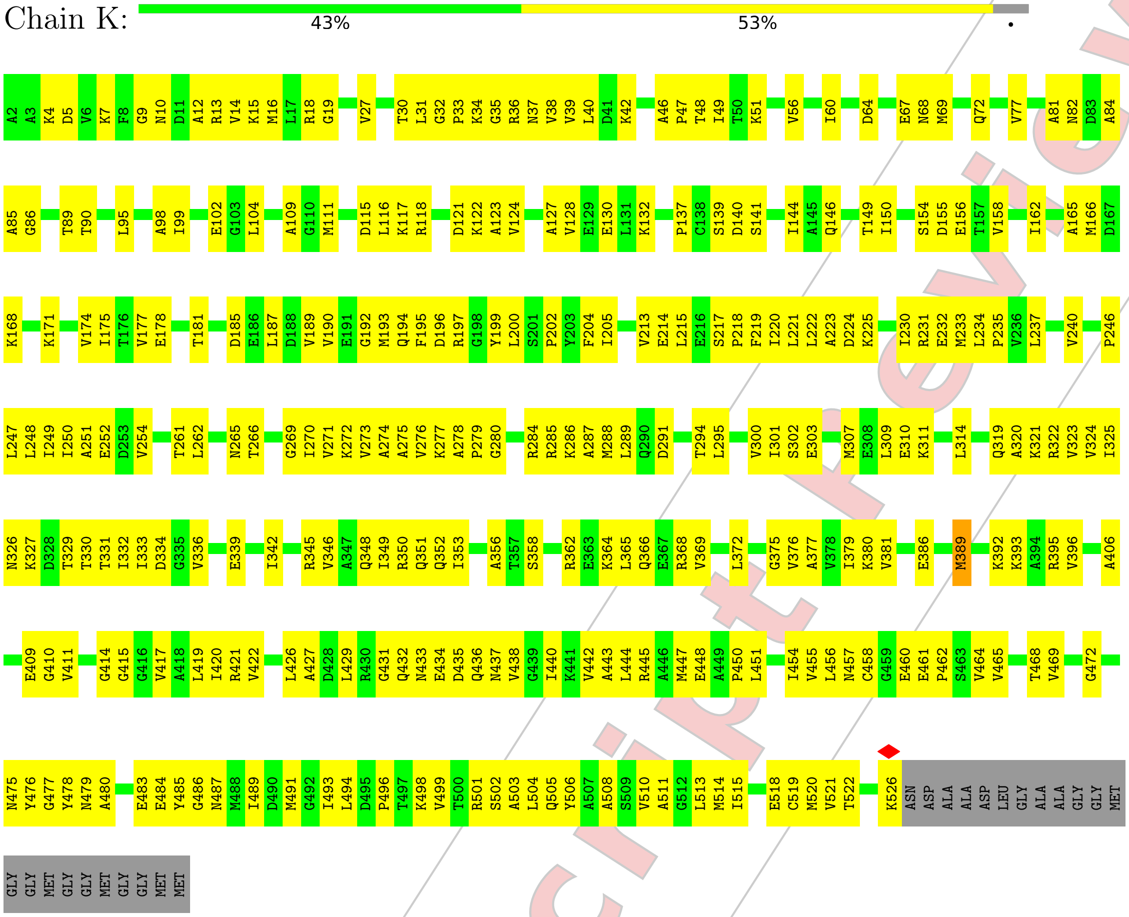

• Molecule 1: Chaperonin GroEL

Chain L:

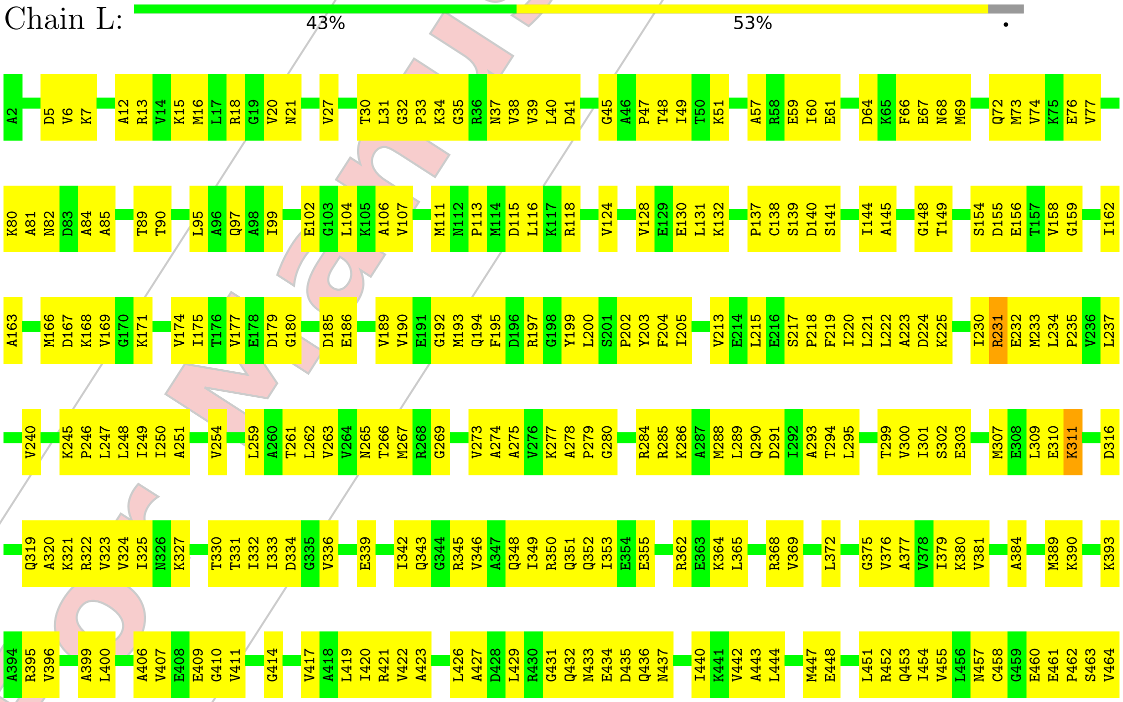

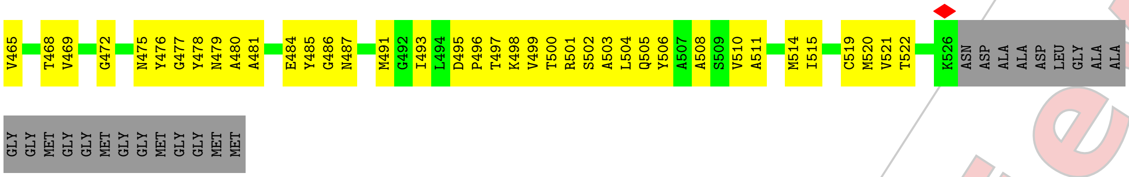

• Molecule 1: Chaperonin GroEL

Chain M:

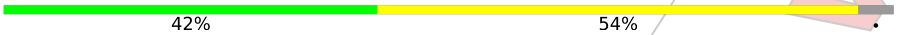

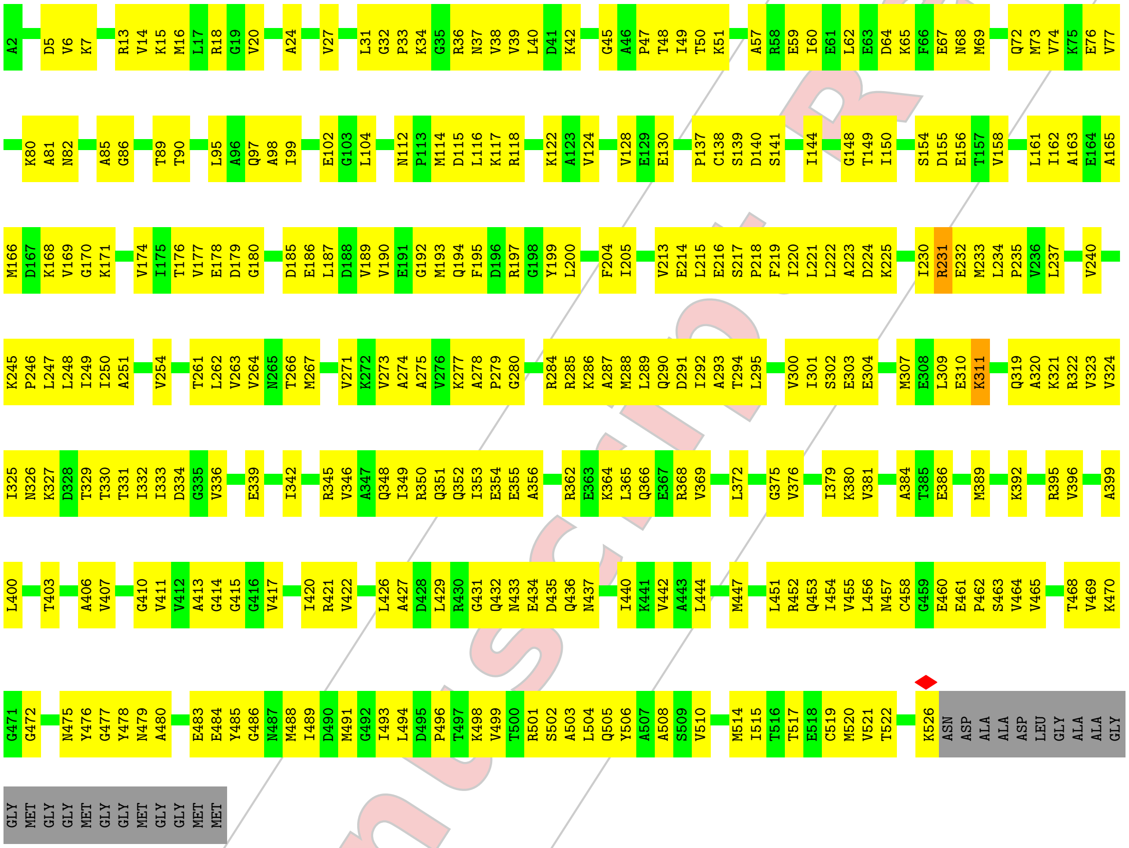

• Molecule 1: Chaperonin GroEL

Chain N:

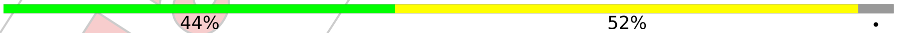

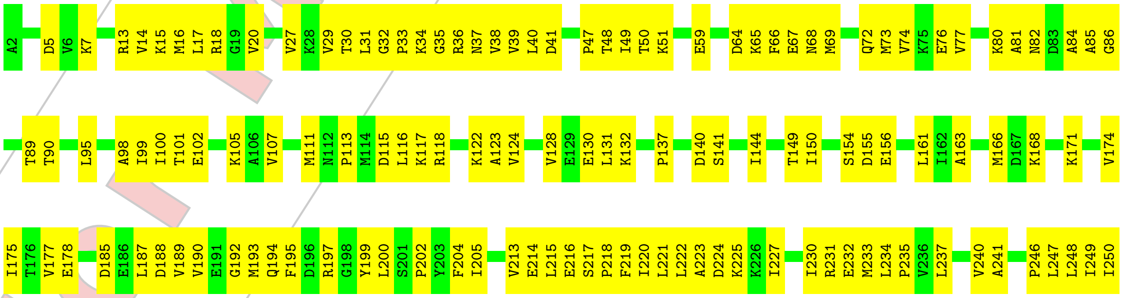

A251 E252 D253 V254 L262 T266 V271 K272 V273 A274 A275 V276 K277 A278 P279 G280 R284 R285 K286 A287 M288 L289 Q290 D291 I292 A293 T294 L295 V300 I301 S302 E303 M307 E308 L309 E310 K311 L314 Q319 A320 K321 R322 V323 V324 I325 N326 K327 D328 T329 T330 T331 I332

I333 D334 G335 V336 E339 I342 R345 V346 A347 Q348 I349 R350 Q351 Q352 E355 A356 R362 E363 K364 L365 Q366 E367 R368 L372 G375 V376 I379 K380 V381 G382 A383 E388 M389 K392 R395 T403 A406 G410 V411 V412 A413 G414 G415 G416 V417 A418

L419 I420 R421 V422 A427 D428 L429 R430 G431 Q432 N433 E434 D435 Q436 N437 V438 G439 I440 K441 V442 A443 L444 R445 A446 M447 E448 A449 P450 L451 R452 Q453 I454 V455 L456 N457 C458 G459 E460 E461 P462 S463 V464 V465 V469 G472 N475 Y476 G477 Y478 N479 A480 E483 E484 Y485 G486

N487 M488 M491 G492 I493 L494 D495 P496 T497 K498 V499 T500 R501 S502 A503 L504 Q505 Y506 A507 A508 S509 V510 A511 G512 L513 M514 I515 T516 T517 E518 C519 M520 V521 T522 K526 ASN ASP ALA ALA ASP LEU GLY ALA ALA GLY GLY MET GLY GLY MET GLY GLY MET GLY GLY MET MET

- Molecule 2: Co-chaperonin GroES

Chain O: 42% 56% •

MET N2 I3 R4 P5 L6 H7 D8 R9 V10 I11 V12 K13 R14 K20 G24 I25 V26 L27 T28 A31 R37 G38 E39 V40 L41 A42 V43 G46 R47 K55 P56 L57 D58 V59 K60 V61 G62 D63 I64 V65 I66 F67 N68 D69 V73 E76 K77 I78 V83 L84

I85 M86 S87 E88 S89 D90 I91 I94 V95 E96 ALA

- Molecule 2: Co-chaperonin GroES

Chain P: 40% 58% •

MET N2 I3 R4 P5 L6 H7 D8 R9 V10 I11 V12 K13 R14 K15 K20 G23 G24 I25 V26 L27 A31 R37 G38 E39 V40 L41 A42 V43 G46 R47 I48 L49 E53 V54 K55 P56 L57 D58 V59 G62 D63 I64 V65 I66 F67 N68 D69 V73 K74 S75 E76

K77 I78 D79 N80 V83 L84 I85 M86 S87 E88 S89 D90 I91 L92 A93 I94 V95 E96 ALA

- Molecule 2: Co-chaperonin GroES

Chain Q: 43% 55% •

MET N2 I3 R4 P5 L6 H7 D8 R9 V10 I11 V12 K13 R14 K15 I25 V26 L27 T28 G29 S30 A31 R37 G38 E39 V40 L41 A42 V43 G44 N45 G46 R47 K55 P56 L57 D58 V59 G62 D63 I64 V65 I66 F67 N68 D69 V73 K74 S75 E76 K77 I78 V83 L84

I85 M86 S87 E88 S89 D90 I91 I94 V95 E96 ALA

- Molecule 2: Co-chaperonin GroES

Chain R: 43% 55% •

MET N2 I3 R4 P5 L6 H7 D8 R9 V10 I11 V12 K13 R14 K15 K20 S21 G24 I25 V26 L27 A31 R37 G38 E39 V40 L41 A42 V43 G46 R47 K55 P56 L57 D58 V59 K60 D63 I64 V65 I66 F67 N68 D69 V73 K74 S75 E76 K77 I78 D79 N80

For Manuscript Review

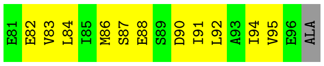

• Molecule 2: Co-chaperonin GroES

Chain S:
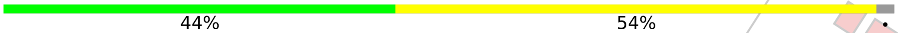

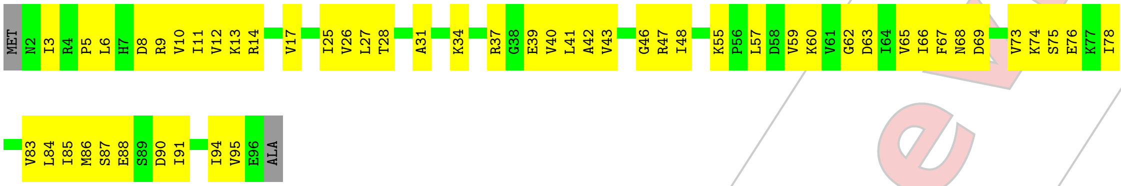

• Molecule 2: Co-chaperonin GroES

Chain T:
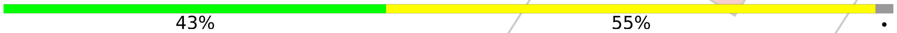

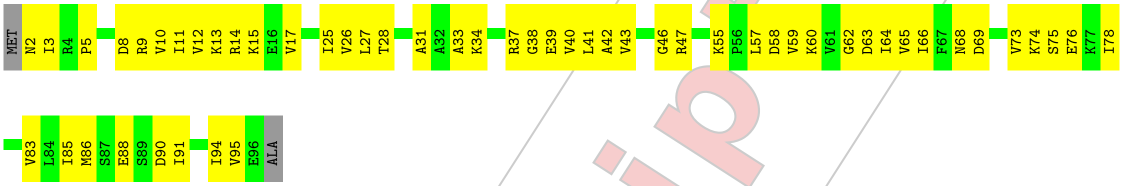

• Molecule 2: Co-chaperonin GroES

Chain U:
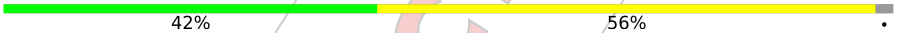

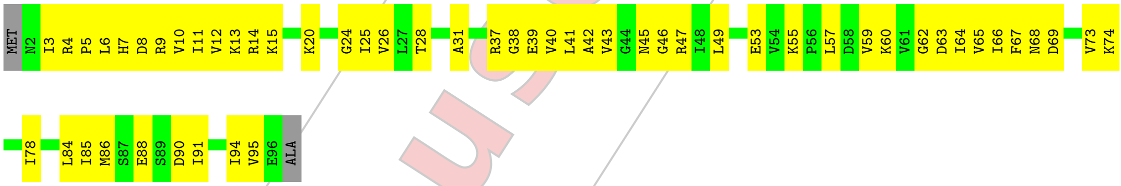

For Manuscript Review

4 Experimental information (i)

Property	Value	Source
EM reconstruction method	SUBTOMOGRAM AVERAGING	Depositor
Imposed symmetry	POINT, C7	Depositor
Number of subtomograms used	10130	Depositor
Resolution determination method	FSC 0.143 CUT-OFF	Depositor
CTF correction method	PHASE FLIPPING AND AMPLITUDE CORRECTION	Depositor
Microscope	FEI TITAN KRIOS	Depositor
Voltage (kV)	300	Depositor
Electron dose ($e^-/\text{Å}^2$)	120	Depositor
Minimum defocus (nm)	2500	Depositor
Maximum defocus (nm)	5000	Depositor
Magnification	Not provided	
Image detector	GATAN K2 SUMMIT (4k x 4k)	Depositor
Maximum map value	0.512	Depositor
Minimum map value	-0.283	Depositor
Average map value	-0.000	Depositor
Map value standard deviation	0.052	Depositor
Recommended contour level	0.0981	Depositor
Map size (Å)	450.56, 450.56, 450.56	wwPDB
Map dimensions	128, 128, 128	wwPDB
Map angles (°)	90.0, 90.0, 90.0	wwPDB
Pixel spacing (Å)	3.52, 3.52, 3.52	Depositor

For Manuscript Review

5 Model quality ⓘ

5.1 Standard geometry ⓘ

Bond lengths and bond angles in the following residue types are not validated in this section: K, MG, ADP, ATP

The Z score for a bond length (or angle) is the number of standard deviations the observed value is removed from the expected value. A bond length (or angle) with $|Z| > 5$ is considered an outlier worth inspection. RMSZ is the root-mean-square of all Z scores of the bond lengths (or angles).

Mol	Chain	Bond lengths		Bond angles	
		RMSZ	#$\|Z\|$ >5	RMSZ	#$\|Z\|$ >5
1	A	0.28	0/3879	0.52	0/5238
1	B	0.28	0/3879	0.52	0/5238
1	C	0.28	0/3879	0.52	0/5238
1	D	0.28	0/3879	0.53	0/5238
1	E	0.28	0/3879	0.52	0/5238
1	F	0.28	0/3879	0.52	0/5238
1	G	0.28	0/3879	0.53	1/5238 (0.0%)
1	H	0.28	0/3892	0.53	0/5254
1	I	0.29	0/3892	0.54	0/5254
1	J	0.29	0/3892	0.53	0/5254
1	K	0.28	0/3892	0.53	1/5254 (0.0%)
1	L	0.28	0/3892	0.54	0/5254
1	M	0.29	0/3892	0.55	0/5254
1	N	0.28	0/3892	0.52	0/5254
2	O	0.30	0/690	0.56	0/930
2	P	0.30	0/690	0.56	0/930
2	Q	0.29	0/690	0.57	0/930
2	R	0.29	0/690	0.56	0/930
2	S	0.28	0/690	0.54	0/930
2	T	0.29	0/690	0.55	0/930
2	U	0.29	0/690	0.54	0/930
All	All	0.28	0/59227	0.53	2/79954 (0.0%)

There are no bond length outliers.

All (2) bond angle outliers are listed below:

Mol	Chain	Res	Type	Atoms	Z	Observed(^{o})	Ideal(^{o})
1	K	389	MET	CA-CB-CG	5.27	122.25	113.30
1	G	514	MET	CA-CB-CG	5.22	122.17	113.30

For Manuscript Review

There are no chirality outliers.

There are no planarity outliers.

5.2 Too-close contacts ⓘ

In the following table, the Non-H and H(model) columns list the number of non-hydrogen atoms and hydrogen atoms in the chain respectively. The H(added) column lists the number of hydrogen atoms added and optimized by MolProbity. The Clashes column lists the number of clashes within the asymmetric unit, whereas Symm-Clashes lists symmetry-related clashes.

Mol	Chain	Non-H	H(model)	H(added)	Clashes	Symm-Clashes
1	A	3851	0	3970	274	0
1	B	3851	0	3971	274	0
1	C	3851	0	3971	268	0
1	D	3851	0	3970	256	0
1	E	3851	0	3970	263	0
1	F	3851	0	3970	278	0
1	G	3851	0	3970	256	0
1	H	3864	0	3989	274	0
1	I	3864	0	3989	266	0
1	J	3864	0	3989	279	0
1	K	3864	0	3989	283	0
1	L	3864	0	3989	269	0
1	M	3864	0	3989	291	0
1	N	3864	0	3989	281	0
2	O	687	0	718	65	0
2	P	687	0	718	69	0
2	Q	687	0	718	64	0
2	R	687	0	718	66	0
2	S	687	0	718	62	0
2	T	687	0	718	66	0
2	U	687	0	718	61	0
3	A	31	0	12	6	0
3	B	31	0	12	8	0
3	C	31	0	12	6	0
3	D	31	0	12	6	0
3	E	31	0	12	8	0
3	F	31	0	12	6	0
3	G	31	0	12	7	0
4	A	1	0	0	0	0
4	B	1	0	0	0	0
4	C	1	0	0	0	0
4	D	1	0	0	0	0
4	E	1	0	0	0	0

Continued on next page...

For Manuscript Review

Continued from previous page...

Mol	Chain	Non-H	H(model)	H(added)	Clashes	Symm-Clashes
4	F	1	0	0	0	0
4	G	1	0	0	0	0
4	H	1	0	0	0	0
4	I	1	0	0	0	0
4	J	1	0	0	0	0
4	K	1	0	0	0	0
4	L	1	0	0	0	0
4	M	1	0	0	0	0
4	N	1	0	0	0	0
5	A	1	0	0	0	0
5	B	1	0	0	0	0
5	C	1	0	0	0	0
5	D	1	0	0	0	0
5	E	1	0	0	0	0
5	F	1	0	0	0	0
5	G	1	0	0	0	0
5	H	1	0	0	0	0
5	I	1	0	0	0	0
5	J	1	0	0	0	0
5	K	1	0	0	0	0
5	L	1	0	0	0	0
5	M	1	0	0	0	0
5	N	1	0	0	0	0
6	H	27	0	12	5	0
6	I	27	0	12	4	0
6	J	27	0	12	5	0
6	K	27	0	12	5	0
6	L	27	0	12	4	0
6	M	27	0	12	4	0
6	N	27	0	12	7	0
7	A	30	0	0	2	0
7	B	29	0	0	2	0
7	C	28	0	0	1	0
7	D	30	0	0	4	0
7	E	29	0	0	2	0
7	F	30	0	0	4	0
7	G	27	0	0	2	0
7	H	1	0	0	0	0
7	I	1	0	0	0	0
7	J	1	0	0	0	0
7	K	1	0	0	0	0
7	L	1	0	0	0	0

Continued on next page...

For Manuscript Review

For Manuscript Review

Continued from previous page...

Mol	Chain	Non-H	H(model)	H(added)	Clashes	Symm-Clashes
7	M	1	0	0	0	0
7	N	1	0	0	0	0
All	All	59458	0	60909	4107	0

The all-atom clashscore is defined as the number of clashes found per 1000 atoms (including hydrogen atoms). The all-atom clashscore for this structure is 34.

All (4107) close contacts within the same asymmetric unit are listed below, sorted by their clash magnitude.

Atom-1	Atom-2	Interatomic distance (Å)	Clash overlap (Å)
1:D:233:MET:HB3	1:D:237:LEU:HD23	1.49	0.95
1:L:166:MET:HB3	1:L:175:ILE:HD11	1.50	0.93
1:C:233:MET:HB3	1:C:237:LEU:HD23	1.50	0.92
1:J:249:ILE:HB	1:J:275:ALA:HA	1.53	0.90
1:D:240:VAL:HG21	1:D:247:LEU:HD12	1.52	0.89
1:K:192:GLY:H	1:K:375:GLY:HA2	1.38	0.88
1:L:417:VAL:HG11	1:L:477:GLY:HA3	1.54	0.88
2:O:57:LEU:HD23	2:O:88:GLU:HB2	1.56	0.88
1:K:249:ILE:HB	1:K:275:ALA:HA	1.53	0.87
1:F:233:MET:HB3	1:F:237:LEU:HD23	1.55	0.87
1:M:192:GLY:H	1:M:375:GLY:HA2	1.40	0.86
1:C:342:ILE:HG23	1:C:372:LEU:HG	1.54	0.86
1:K:279:PRO:HG2	1:K:288:MET:HB3	1.58	0.86
1:L:249:ILE:HB	1:L:275:ALA:HA	1.56	0.86
1:N:249:ILE:HB	1:N:275:ALA:HA	1.58	0.86
1:L:77:VAL:HG21	1:L:510:VAL:HB	1.58	0.86
1:M:249:ILE:HB	1:M:275:ALA:HA	1.54	0.86
1:B:342:ILE:HG23	1:B:372:LEU:HG	1.55	0.86
1:G:342:ILE:HG23	1:G:372:LEU:HG	1.55	0.86
2:U:12:VAL:HG12	2:U:40:VAL:HG12	1.58	0.86
1:E:342:ILE:HG23	1:E:372:LEU:HG	1.56	0.85
1:H:249:ILE:HB	1:H:275:ALA:HA	1.57	0.85
1:I:249:ILE:HB	1:I:275:ALA:HA	1.58	0.85
1:M:166:MET:O	1:M:170:GLY:N	2.09	0.84
1:M:279:PRO:HG2	1:M:288:MET:HB3	1.59	0.84
2:Q:67:PHE:HB3	2:Q:91:ILE:HD13	1.59	0.84
1:H:352:GLN:OE1	1:H:368:ARG:NH2	2.09	0.84
1:L:240:VAL:HG11	1:L:247:LEU:HB2	1.60	0.84
1:K:77:VAL:HG21	1:K:510:VAL:HB	1.59	0.84
1:A:281:PHE:H	1:A:284:ARG:HG3	1.41	0.84
1:M:352:GLN:OE1	1:M:368:ARG:NH2	2.10	0.83

Continued on next page...

Continued from previous page...

Atom-1	Atom-2	Interatomic distance (Å)	Clash overlap (Å)
1:H:279:PRO:HG2	1:H:288:MET:HB3	1.58	0.83
1:I:240:VAL:HG11	1:I:247:LEU:HB2	1.59	0.83
1:M:240:VAL:HG11	1:M:247:LEU:HB2	1.59	0.83
1:J:417:VAL:HG11	1:J:477:GLY:HA3	1.59	0.83
1:M:85:ALA:HB1	1:M:499:VAL:HA	1.57	0.83
1:H:417:VAL:HG11	1:H:477:GLY:HA3	1.60	0.82
1:N:15:LYS:HD3	1:N:18:ARG:HH21	1.42	0.82
1:H:85:ALA:HB1	1:H:499:VAL:HA	1.60	0.82
1:I:192:GLY:H	1:I:375:GLY:HA2	1.43	0.82
1:K:85:ALA:HB1	1:K:499:VAL:HA	1.61	0.82
1:E:281:PHE:H	1:E:284:ARG:HG3	1.44	0.82
1:H:240:VAL:HG11	1:H:247:LEU:HB2	1.60	0.82
1:M:77:VAL:HG21	1:M:510:VAL:HB	1.60	0.82
1:H:77:VAL:HG21	1:H:510:VAL:HB	1.61	0.82
1:L:321:LYS:HB2	1:L:334:ASP:HB3	1.62	0.82
1:I:321:LYS:HB2	1:I:334:ASP:HB3	1.60	0.82
2:O:65:VAL:HG12	2:O:94:ILE:HG22	1.62	0.82
1:J:333:ILE:HG23	1:J:376:VAL:HG21	1.62	0.81
2:O:12:VAL:HG12	2:O:40:VAL:HA	1.60	0.81
1:I:279:PRO:HG2	1:I:288:MET:HB3	1.61	0.81
1:J:85:ALA:HB1	1:J:499:VAL:HA	1.61	0.81
1:K:321:LYS:HB2	1:K:334:ASP:HB3	1.62	0.81
1:N:321:LYS:HB2	1:N:334:ASP:HB3	1.62	0.81
1:J:240:VAL:HG11	1:J:247:LEU:HB2	1.62	0.81
2:Q:73:VAL:HA	2:Q:86:MET:HB3	1.62	0.81
2:O:95:VAL:HA	2:P:3:ILE:HG22	1.62	0.81
2:P:57:LEU:HD23	2:P:88:GLU:HB2	1.63	0.81
1:K:333:ILE:HG23	1:K:376:VAL:HG21	1.63	0.81
1:N:232:GLU:HB3	1:N:309:LEU:HB2	1.62	0.81
1:J:321:LYS:HB2	1:J:334:ASP:HB3	1.63	0.81
1:F:342:ILE:HG23	1:F:372:LEU:HG	1.60	0.80
1:E:320:ALA:HA	1:E:336:VAL:H	1.45	0.80
1:N:192:GLY:HA3	1:N:376:VAL:HG13	1.64	0.80
1:K:240:VAL:HG11	1:K:247:LEU:HB2	1.62	0.80
1:L:333:ILE:HG23	1:L:376:VAL:HG21	1.64	0.80
1:N:279:PRO:HG2	1:N:288:MET:HB3	1.63	0.80
1:I:85:ALA:HB1	1:I:499:VAL:HA	1.63	0.80
1:M:321:LYS:HB2	1:M:334:ASP:HB3	1.63	0.80
1:H:69:MET:HB2	1:I:47:PRO:HG2	1.63	0.80
1:N:192:GLY:H	1:N:375:GLY:HA2	1.45	0.80
1:M:417:VAL:HG11	1:M:477:GLY:HA3	1.63	0.80

Continued on next page...

For Manuscript Review

Continued from previous page...

Atom-1	Atom-2	Interatomic distance (Å)	Clash overlap (Å)
1:M:413:ALA:HB1	1:M:488:MET:HB2	1.63	0.79
1:N:39:VAL:HG22	1:N:49:ILE:HG12	1.62	0.79
1:B:39:VAL:HG22	1:B:49:ILE:HG12	1.65	0.79
1:L:279:PRO:HG2	1:L:288:MET:HB3	1.64	0.79
1:K:365:LEU:HD23	1:K:368:ARG:HE	1.45	0.79
1:C:31:LEU:HB2	1:C:90:THR:HG21	1.65	0.79
1:H:232:GLU:HB3	1:H:309:LEU:HB2	1.63	0.79
2:U:11:ILE:HG12	2:U:85:ILE:HG12	1.65	0.79
1:K:417:VAL:HG11	1:K:477:GLY:HA3	1.65	0.79
1:B:223:ALA:HA	1:B:301:ILE:HB	1.65	0.78
1:B:248:LEU:HD22	1:B:323:VAL:HG11	1.65	0.78
1:I:417:VAL:HG11	1:I:477:GLY:HA3	1.65	0.78
1:N:333:ILE:HG23	1:N:376:VAL:HG21	1.65	0.78
1:I:69:MET:HB2	1:J:47:PRO:HG2	1.65	0.78
1:I:352:GLN:OE1	1:I:368:ARG:NH2	2.15	0.78
1:N:224:ASP:HB3	1:N:302:SER:HB3	1.65	0.78
1:N:240:VAL:HG11	1:N:247:LEU:HB2	1.64	0.78
1:A:233:MET:HB3	1:A:237:LEU:HD23	1.63	0.78
1:H:207:LYS:HD2	1:H:214:GLU:HG3	1.64	0.78
1:L:85:ALA:HB1	1:L:499:VAL:HA	1.64	0.78
1:I:232:GLU:HB3	1:I:309:LEU:HB2	1.66	0.78
2:R:57:LEU:HD23	2:R:88:GLU:HB2	1.66	0.78
1:L:365:LEU:HD23	1:L:368:ARG:HE	1.48	0.78
1:F:39:VAL:HG22	1:F:49:ILE:HG12	1.65	0.78
1:L:39:VAL:HG22	1:L:49:ILE:HG12	1.66	0.78
1:B:122:LYS:HG2	1:B:429:LEU:HD21	1.64	0.78
1:D:420:ILE:HG12	1:D:448:GLU:HG2	1.66	0.78
1:B:233:MET:HB3	1:B:237:LEU:HD23	1.66	0.77
1:F:342:ILE:HG12	1:F:372:LEU:HD11	1.66	0.77
1:E:122:LYS:HG2	1:E:429:LEU:HD21	1.65	0.77
1:N:417:VAL:HG11	1:N:477:GLY:HA3	1.64	0.77
1:E:39:VAL:HG22	1:E:49:ILE:HG12	1.65	0.77
1:G:295:LEU:HA	1:G:342:ILE:HD11	1.65	0.77
1:H:224:ASP:HB3	1:H:302:SER:HB3	1.64	0.77
1:J:192:GLY:H	1:J:375:GLY:HA2	1.48	0.77
1:G:193:MET:HG2	1:G:295:LEU:HD22	1.66	0.77
1:L:192:GLY:H	1:L:375:GLY:HA2	1.49	0.77
2:P:65:VAL:HG12	2:P:94:ILE:HG22	1.64	0.77
1:E:169:VAL:HG22	1:E:173:GLY:HA3	1.67	0.77
1:A:115:ASP:OD1	1:A:432:GLN:NE2	2.16	0.77
1:B:393:LYS:NZ	1:B:397:GLU:OE2	2.17	0.77

Continued on next page...

For Manuscript Review

Continued from previous page...

Atom-1	Atom-2	Interatomic distance (Å)	Clash overlap (Å)
1:B:295:LEU:HA	1:B:342:ILE:HD11	1.67	0.77
1:N:352:GLN:OE1	1:N:368:ARG:NH2	2.14	0.77
2:T:69:ASP:HA	2:T:73:VAL:HG21	1.67	0.77
1:F:215:LEU:HD22	1:F:246:PRO:HB3	1.66	0.76
1:C:421:ARG:NH2	1:C:476:TYR:O	2.16	0.76
1:E:31:LEU:HB2	1:E:90:THR:HG21	1.67	0.76
2:P:69:ASP:HA	2:P:73:VAL:HG21	1.66	0.76
2:U:57:LEU:HD23	2:U:88:GLU:HB2	1.67	0.76
1:G:122:LYS:HG2	1:G:429:LEU:HD21	1.66	0.76
1:J:279:PRO:HG2	1:J:288:MET:HB3	1.67	0.76
1:A:122:LYS:HG2	1:A:429:LEU:HD21	1.67	0.76
1:G:223:ALA:HA	1:G:301:ILE:HB	1.68	0.76
1:L:232:GLU:HB3	1:L:309:LEU:HB2	1.68	0.76
1:G:240:VAL:HG21	1:G:247:LEU:HD12	1.65	0.76
1:K:349:ILE:HG21	1:K:368:ARG:HB2	1.68	0.76
1:M:333:ILE:HG23	1:M:376:VAL:HG21	1.68	0.76
1:C:122:LYS:HG2	1:C:429:LEU:HD21	1.66	0.76
1:N:349:ILE:HG21	1:N:368:ARG:HB2	1.68	0.76
1:B:421:ARG:NH2	1:B:476:TYR:O	2.16	0.76
2:T:57:LEU:HD23	2:T:88:GLU:HB2	1.68	0.76
1:D:215:LEU:HD22	1:D:246:PRO:HB3	1.68	0.75
1:C:281:PHE:H	1:C:284:ARG:HG3	1.52	0.75
1:N:85:ALA:HB1	1:N:499:VAL:HA	1.66	0.75
1:K:232:GLU:HB3	1:K:309:LEU:HB2	1.69	0.75
2:T:12:VAL:HG12	2:T:40:VAL:HA	1.69	0.75
1:G:31:LEU:HB2	1:G:90:THR:HG21	1.68	0.75
1:H:220:ILE:HD11	1:H:250:ILE:HD12	1.69	0.75
1:I:224:ASP:HB3	1:I:302:SER:HB3	1.65	0.75
1:F:180:GLY:N	1:F:381:VAL:O	2.20	0.75
1:C:295:LEU:HA	1:C:342:ILE:HD11	1.68	0.75
1:D:122:LYS:HG2	1:D:429:LEU:HD21	1.67	0.75
1:D:291:ASP:HB3	1:D:372:LEU:HD21	1.69	0.75
1:F:122:LYS:HG2	1:F:429:LEU:HD21	1.68	0.75
1:H:321:LYS:HB2	1:H:334:ASP:HB3	1.67	0.75
1:M:69:MET:HB2	1:N:47:PRO:HG2	1.69	0.74
2:Q:78:ILE:HG12	2:Q:83:VAL:HG21	1.69	0.74
1:F:51:LYS:NZ	3:F:601:ATP:O1A	2.20	0.74
1:J:232:GLU:HB3	1:J:309:LEU:HB2	1.70	0.74
1:C:169:VAL:HG22	1:C:173:GLY:HA3	1.69	0.74
1:N:274:ALA:HB1	1:N:325:ILE:HD13	1.69	0.74
1:C:469:VAL:HG13	1:C:477:GLY:HA2	1.70	0.74

Continued on next page...

For Manuscript Review

Continued from previous page...

Atom-1	Atom-2	Interatomic distance (Å)	Clash overlap (Å)
1:M:214:GLU:HG3	1:M:324:VAL:HG22	1.68	0.74
1:D:469:VAL:HG13	1:D:477:GLY:HA2	1.70	0.74
1:B:169:VAL:HG22	1:B:173:GLY:HA3	1.70	0.74
1:G:393:LYS:NZ	1:G:397:GLU:OE2	2.18	0.74
1:I:420:ILE:HD12	1:I:451:LEU:HD13	1.70	0.74
1:M:353:ILE:HG23	1:M:362:ARG:HH12	1.52	0.74
1:N:213:VAL:HB	1:N:325:ILE:HG12	1.69	0.74
2:T:57:LEU:O	2:T:60:LYS:NZ	2.19	0.74
1:E:421:ARG:NH2	1:E:476:TYR:O	2.19	0.74
1:M:289:LEU:HD23	1:M:300:VAL:HG13	1.68	0.74
1:E:248:LEU:HD22	1:E:323:VAL:HG11	1.68	0.74
1:J:295:LEU:HD23	1:J:342:ILE:HG12	1.70	0.73
1:J:224:ASP:HB3	1:J:302:SER:HB3	1.68	0.73
1:D:169:VAL:HB	1:D:377:ALA:HB2	1.70	0.73
1:E:197:ARG:O	1:E:330:THR:OG1	2.06	0.73
2:S:69:ASP:HA	2:S:73:VAL:HG21	1.69	0.73
1:G:233:MET:HB3	1:G:237:LEU:HG	1.71	0.73
1:I:77:VAL:HG21	1:I:510:VAL:HB	1.71	0.73
1:M:295:LEU:HD23	1:M:342:ILE:HG12	1.71	0.73
1:M:420:ILE:HD12	1:M:451:LEU:HD13	1.70	0.73
1:C:339:GLU:HA	1:C:342:ILE:HD12	1.71	0.73
1:H:225:LYS:HD3	1:H:303:GLU:HG3	1.71	0.73
1:J:77:VAL:HG21	1:J:510:VAL:HB	1.69	0.73
1:L:339:GLU:O	1:L:343:GLN:NE2	2.22	0.73
1:M:6:VAL:HG22	1:M:521:VAL:HG12	1.71	0.73
1:G:215:LEU:HD22	1:G:246:PRO:HB3	1.70	0.73
1:K:420:ILE:HD12	1:K:451:LEU:HD13	1.71	0.73
1:G:115:ASP:OD1	1:G:432:GLN:NE2	2.21	0.73
1:I:349:ILE:HG21	1:I:368:ARG:HB2	1.70	0.73
1:D:41:ASP:HA	1:D:47:PRO:HB3	1.71	0.73
1:N:295:LEU:HD23	1:N:342:ILE:HG12	1.71	0.73
1:G:186:GLU:HB3	1:G:380:LYS:HB2	1.71	0.72
1:M:479:ASN:O	1:M:483:GLU:N	2.22	0.72
1:C:197:ARG:O	1:C:330:THR:OG1	2.07	0.72
1:D:248:LEU:HD22	1:D:323:VAL:HG11	1.71	0.72
1:F:421:ARG:NH2	1:F:476:TYR:O	2.18	0.72
1:A:21:ASN:OD1	1:A:97:GLN:NE2	2.23	0.72
1:A:262:LEU:HD22	1:A:273:VAL:HG21	1.70	0.72
1:A:469:VAL:HG13	1:A:477:GLY:HA2	1.71	0.72
1:D:281:PHE:H	1:D:284:ARG:HG3	1.54	0.72
1:F:115:ASP:OD1	1:F:432:GLN:NE2	2.20	0.72

Continued on next page...

For Manuscript Review

Continued from previous page...

Atom-1	Atom-2	Interatomic distance (Å)	Clash overlap (Å)
1:J:352:GLN:HA	1:J:355:GLU:HG3	1.71	0.72
1:C:203:TYR:HB2	1:C:263:VAL:HB	1.71	0.72
1:D:169:VAL:HG22	1:D:173:GLY:HA3	1.72	0.72
1:F:20:VAL:HG13	1:F:74:VAL:HG21	1.71	0.72
1:J:431:GLY:N	1:J:437:ASN:OD1	2.23	0.72
1:K:139:SER:HA	1:K:171:LYS:HE3	1.72	0.72
1:K:289:LEU:HD23	1:K:300:VAL:HG13	1.72	0.72
2:T:10:VAL:N	2:T:86:MET:O	2.21	0.72
1:A:219:PHE:HD2	1:A:240:VAL:HG22	1.54	0.72
1:K:192:GLY:HA3	1:K:376:VAL:HG13	1.69	0.72
1:K:432:GLN:NE2	1:K:436:GLN:OE1	2.22	0.72
2:T:9:ARG:HB3	2:T:85:ILE:HD11	1.71	0.72
1:F:184:GLN:NE2	1:F:185:ASP:OD1	2.23	0.72
1:F:169:VAL:HG22	1:F:173:GLY:HA3	1.71	0.72
1:G:240:VAL:O	1:G:244:GLY:N	2.23	0.72
1:D:231:ARG:HA	1:D:234:LEU:HD23	1.72	0.71
1:I:10:ASN:HA	1:I:13:ARG:HB2	1.71	0.71
1:K:111:MET:HB2	1:K:116:LEU:HD11	1.71	0.71
1:N:479:ASN:ND2	1:N:491:MET:SD	2.63	0.71
2:R:12:VAL:HG12	2:R:40:VAL:HA	1.72	0.71
1:G:281:PHE:H	1:G:284:ARG:HG3	1.53	0.71
2:P:12:VAL:HG22	2:P:86:MET:HE1	1.72	0.71
2:Q:11:ILE:HD11	2:Q:83:VAL:HB	1.70	0.71
1:A:421:ARG:NH2	1:A:476:TYR:O	2.17	0.71
1:A:58:ARG:HA	1:A:75:LYS:HD3	1.72	0.71
1:F:231:ARG:HA	1:F:234:LEU:HD23	1.72	0.71
1:C:115:ASP:OD1	1:C:432:GLN:NE2	2.23	0.71
1:D:326:ASN:OD1	1:D:329:THR:N	2.23	0.71
1:F:240:VAL:O	1:F:244:GLY:N	2.24	0.71
1:H:47:PRO:HG2	1:N:69:MET:HB2	1.73	0.71
2:P:10:VAL:N	2:P:86:MET:O	2.22	0.71
1:E:420:ILE:HG12	1:E:448:GLU:HG2	1.71	0.71
1:F:393:LYS:NZ	1:F:397:GLU:OE2	2.23	0.71
1:H:349:ILE:HG21	1:H:368:ARG:HB2	1.72	0.71
1:J:339:GLU:O	1:J:343:GLN:NE2	2.24	0.71
1:L:322:ARG:HB2	1:L:333:ILE:HB	1.70	0.71
1:F:102:GLU:HG3	1:F:442:VAL:HG22	1.73	0.71
1:F:193:MET:HG2	1:F:295:LEU:HD22	1.73	0.71
1:F:491:MET:HE3	1:F:493:ILE:HD12	1.72	0.71
1:G:180:GLY:N	1:G:381:VAL:O	2.19	0.71
1:G:193:MET:HB2	1:G:332:ILE:HB	1.72	0.71

Continued on next page...

For Manuscript Review

Continued from previous page...

Atom-1	Atom-2	Interatomic distance (Å)	Clash overlap (Å)
1:J:349:ILE:HD13	1:J:368:ARG:HG2	1.71	0.71
1:K:352:GLN:O	1:K:362:ARG:NH2	2.23	0.71
1:L:224:ASP:HB3	1:L:302:SER:HB3	1.72	0.71
1:M:431:GLY:N	1:M:437:ASN:OD1	2.23	0.71
1:N:81:ALA:O	1:N:85:ALA:CB	2.38	0.71
1:E:223:ALA:HA	1:E:301:ILE:HB	1.71	0.71
2:S:11:ILE:O	2:S:41:LEU:N	2.24	0.71
1:A:240:VAL:O	1:A:244:GLY:N	2.24	0.71
1:I:40:LEU:HD22	1:I:59:GLU:HG2	1.71	0.71
1:A:295:LEU:HA	1:A:342:ILE:HD11	1.73	0.70
1:D:240:VAL:O	1:D:244:GLY:N	2.22	0.70
1:J:455:VAL:HG13	1:J:460:GLU:HB2	1.73	0.70
1:M:421:ARG:NH2	1:M:476:TYR:O	2.22	0.70
1:N:431:GLY:N	1:N:437:ASN:OD1	2.24	0.70
1:J:220:ILE:HD11	1:J:250:ILE:HD12	1.72	0.70
1:B:213:VAL:N	1:B:325:ILE:O	2.24	0.70
1:A:12:ALA:HA	1:A:520:MET:HE1	1.73	0.70
1:A:197:ARG:O	1:A:330:THR:OG1	2.10	0.70
1:B:326:ASN:OD1	1:B:329:THR:N	2.23	0.70
1:J:432:GLN:NE2	1:J:436:GLN:OE1	2.24	0.70
1:M:224:ASP:HB3	1:M:302:SER:HB3	1.74	0.70
1:N:31:LEU:O	1:N:457:ASN:ND2	2.23	0.70
2:S:59:VAL:HG11	2:S:91:ILE:HG21	1.71	0.70
1:D:186:GLU:HB3	1:D:380:LYS:HB2	1.72	0.70
1:E:215:LEU:HD22	1:E:246:PRO:HB3	1.71	0.70
1:E:469:VAL:HG13	1:E:477:GLY:HA2	1.72	0.70
1:G:184:GLN:NE2	1:G:185:ASP:OD1	2.25	0.70
1:H:362:ARG:HH21	1:H:366:GLN:HB2	1.54	0.70
2:R:69:ASP:HA	2:R:73:VAL:HG21	1.74	0.70
1:B:281:PHE:H	1:B:284:ARG:HG3	1.55	0.70
1:F:102:GLU:HB3	1:F:442:VAL:HG13	1.73	0.70
1:F:150:ILE:HG13	1:F:493:ILE:HA	1.73	0.70
1:K:224:ASP:HB3	1:K:302:SER:HB3	1.74	0.70
1:L:325:ILE:HG22	1:L:330:THR:HG23	1.73	0.70
1:B:469:VAL:HG13	1:B:477:GLY:HA2	1.73	0.70
1:G:219:PHE:HD2	1:G:240:VAL:HG22	1.57	0.70
1:G:365:LEU:HD13	1:G:368:ARG:HD3	1.73	0.70
1:M:194:GLN:HG3	1:M:331:THR:HB	1.74	0.70
1:N:348:GLN:O	1:N:351:GLN:NE2	2.24	0.70
2:O:10:VAL:N	2:O:86:MET:O	2.25	0.70
2:Q:12:VAL:HG12	2:Q:40:VAL:HA	1.72	0.70

Continued on next page...

For Manuscript Review

Continued from previous page...

Atom-1	Atom-2	Interatomic distance (Å)	Clash overlap (Å)
1:N:220:ILE:HD11	1:N:250:ILE:HD12	1.74	0.70
2:P:94:ILE:HG23	2:Q:6:LEU:HD11	1.74	0.70
2:R:67:PHE:HB3	2:R:91:ILE:HD13	1.73	0.70
1:B:240:VAL:O	1:B:244:GLY:N	2.23	0.70
1:G:356:ALA:O	1:G:362:ARG:NH2	2.24	0.70
1:K:233:MET:HG3	1:K:237:LEU:HG	1.74	0.70
1:K:431:GLY:N	1:K:437:ASN:OD1	2.25	0.70
1:A:169:VAL:HG22	1:A:173:GLY:HA3	1.72	0.70
1:C:169:VAL:HB	1:C:377:ALA:HB2	1.74	0.70
1:E:240:VAL:O	1:E:244:GLY:N	2.25	0.70
1:N:233:MET:HG3	1:N:237:LEU:HG	1.73	0.70
1:E:393:LYS:NZ	1:E:397:GLU:OE2	2.21	0.69
1:M:77:VAL:HG13	1:M:506:TYR:HB3	1.72	0.69
1:D:31:LEU:HB2	1:D:90:THR:HG21	1.73	0.69
1:F:279:PRO:HG2	1:F:288:MET:HB3	1.74	0.69
1:H:479:ASN:N	1:H:484:GLU:O	2.25	0.69
1:A:248:LEU:HD22	1:A:323:VAL:HG11	1.73	0.69
1:A:356:ALA:O	1:A:362:ARG:NH2	2.25	0.69
1:B:51:LYS:NZ	3:B:601:ATP:O1A	2.25	0.69
1:D:232:GLU:HA	1:D:310:GLU:HG3	1.75	0.69
1:G:248:LEU:HD22	1:G:323:VAL:HG11	1.72	0.69
1:H:31:LEU:O	1:H:457:ASN:ND2	2.24	0.69
1:I:193:MET:HG2	1:I:295:LEU:HD13	1.74	0.69
1:J:225:LYS:HD3	1:J:303:GLU:HG3	1.75	0.69
1:L:77:VAL:HG13	1:L:506:TYR:HB3	1.74	0.69
2:S:40:VAL:O	2:S:62:GLY:N	2.21	0.69
1:C:215:LEU:HD22	1:C:246:PRO:HB3	1.74	0.69
1:G:169:VAL:HG22	1:G:173:GLY:HA3	1.73	0.69
1:M:362:ARG:HH21	1:M:366:GLN:HG3	1.57	0.69
1:A:20:VAL:HG13	1:A:74:VAL:HG21	1.74	0.69
1:A:51:LYS:NZ	3:A:601:ATP:O1A	2.26	0.69
1:J:144:ILE:HD12	1:J:166:MET:HE3	1.74	0.69
1:F:263:VAL:O	1:F:266:THR:OG1	2.11	0.69
1:G:263:VAL:O	1:G:266:THR:OG1	2.11	0.69
1:M:520:MET:HG2	1:N:39:VAL:HB	1.75	0.69
1:N:77:VAL:HG21	1:N:510:VAL:HB	1.74	0.69
1:N:225:LYS:HD3	1:N:303:GLU:HG3	1.75	0.69
1:N:362:ARG:HH21	1:N:366:GLN:HB2	1.57	0.69
1:G:495:ASP:OD2	3:G:601:ATP:O2'	2.09	0.69
1:I:413:ALA:HB1	1:I:488:MET:HB2	1.75	0.69
1:K:307:MET:HG2	1:K:311:LYS:HZ2	1.58	0.69

Continued on next page...

For Manuscript Review

Continued from previous page...

Atom-1	Atom-2	Interatomic distance (Å)	Clash overlap (Å)
1:M:186:GLU:O	1:M:380:LYS:N	2.23	0.69
1:M:350:ARG:HD3	1:M:353:ILE:HD12	1.75	0.69
1:B:58:ARG:HA	1:B:75:LYS:HD3	1.73	0.69
1:C:114:MET:SD	7:D:2003:HOH:O	2.51	0.69
1:C:420:ILE:HG12	1:C:448:GLU:HG2	1.75	0.69
1:I:325:ILE:HG22	1:I:330:THR:HG23	1.74	0.69
1:J:192:GLY:HA3	1:J:376:VAL:HG13	1.75	0.69
1:M:128:VAL:HG13	1:M:501:ARG:HG3	1.75	0.69
1:M:232:GLU:HB3	1:M:309:LEU:HB2	1.73	0.69
1:B:20:VAL:HG13	1:B:74:VAL:HG21	1.73	0.69
1:C:252:GLU:OE2	1:C:285:ARG:NH1	2.25	0.69
1:G:197:ARG:O	1:G:330:THR:OG1	2.10	0.69
1:H:81:ALA:O	1:H:85:ALA:CB	2.41	0.69
1:L:284:ARG:CZ	1:L:364:LYS:HB3	2.23	0.69
1:L:295:LEU:HD23	1:L:342:ILE:HG12	1.74	0.69
1:A:39:VAL:HG22	1:A:49:ILE:HG12	1.75	0.69
1:D:308:GLU:H	1:D:311:LYS:HD3	1.57	0.69
1:E:279:PRO:HG2	1:E:288:MET:HB3	1.75	0.69
1:H:322:ARG:HB2	1:H:333:ILE:HB	1.75	0.69
1:K:520:MET:HG2	1:L:39:VAL:HB	1.73	0.69
1:H:15:LYS:HD3	1:H:18:ARG:HH21	1.56	0.68
1:G:130:GLU:HB2	1:G:422:VAL:HG13	1.73	0.68
1:I:289:LEU:HD23	1:I:300:VAL:HG13	1.75	0.68
1:M:174:VAL:HG23	1:M:376:VAL:HA	1.75	0.68
2:U:5:PRO:HG3	2:U:11:ILE:HG13	1.75	0.68
1:E:180:GLY:N	1:E:381:VAL:O	2.20	0.68
1:E:249:ILE:HB	1:E:275:ALA:HA	1.73	0.68
1:I:431:GLY:N	1:I:437:ASN:OD1	2.24	0.68
1:L:141:SER:HB3	1:L:163:ALA:HB1	1.76	0.68
1:B:220:ILE:N	1:B:318:GLY:O	2.23	0.68
1:M:40:LEU:HD13	1:M:59:GLU:HG3	1.75	0.68
1:M:325:ILE:HG22	1:M:330:THR:HG23	1.75	0.68
1:C:346:VAL:HB	1:C:369:VAL:HG13	1.76	0.68
1:F:185:ASP:HA	1:F:381:VAL:HA	1.75	0.68
1:F:226:LYS:HE2	1:F:253:ASP:HB3	1.76	0.68
1:A:193:MET:HG2	1:A:295:LEU:HD22	1.76	0.68
1:H:295:LEU:HA	1:H:342:ILE:HG12	1.75	0.68
1:L:420:ILE:HD12	1:L:451:LEU:HD13	1.74	0.68
1:L:421:ARG:NH2	1:L:476:TYR:O	2.24	0.68
1:F:281:PHE:H	1:F:284:ARG:HG3	1.58	0.68
2:S:13:LYS:HB2	2:S:41:LEU:HD11	1.74	0.68

Continued on next page...

For Manuscript Review

Continued from previous page...

Atom-1	Atom-2	Interatomic distance (Å)	Clash overlap (Å)
1:C:207:LYS:HZ1	1:C:214:GLU:HB2	1.58	0.68
1:C:479:ASN:N	1:C:484:GLU:O	2.26	0.68
1:I:519:CYS:HB3	1:J:38:VAL:HG22	1.76	0.68
1:K:84:ALA:O	1:K:498:LYS:NZ	2.23	0.68
1:L:81:ALA:O	1:L:85:ALA:CB	2.42	0.68
1:L:431:GLY:N	1:L:437:ASN:OD1	2.26	0.68
1:M:353:ILE:HG23	1:M:362:ARG:HH22	1.59	0.68
1:C:263:VAL:O	1:C:266:THR:OG1	2.11	0.68
1:L:346:VAL:HG13	1:L:350:ARG:NH1	2.09	0.68
2:P:11:ILE:HG22	2:P:41:LEU:HB2	1.73	0.68
2:T:13:LYS:HB2	2:T:41:LEU:HD11	1.74	0.68
1:B:353:ILE:HG23	1:B:362:ARG:HB2	1.75	0.68
1:J:325:ILE:HG22	1:J:330:THR:HG23	1.76	0.68
1:A:231:ARG:HH11	1:A:234:LEU:HD11	1.60	0.67
1:H:178:GLU:N	1:H:379:ILE:O	2.21	0.67
1:I:128:VAL:HG13	1:I:501:ARG:HG3	1.76	0.67
1:K:353:ILE:O	1:K:362:ARG:NH1	2.28	0.67
1:M:81:ALA:O	1:M:85:ALA:CB	2.42	0.67
2:T:59:VAL:HG21	2:T:91:ILE:HG21	1.76	0.67
1:A:66:PHE:HB3	1:A:520:MET:HE3	1.76	0.67
1:G:339:GLU:HA	1:G:342:ILE:HD12	1.75	0.67
1:L:104:LEU:HD21	1:L:514:MET:HG3	1.76	0.67
1:B:169:VAL:HB	1:B:377:ALA:HB2	1.77	0.67
1:H:194:GLN:HG3	1:H:331:THR:HB	1.76	0.67
1:J:81:ALA:O	1:J:85:ALA:CB	2.43	0.67
1:K:69:MET:HG2	1:K:520:MET:HE3	1.76	0.67
1:B:31:LEU:HB2	1:B:90:THR:HG21	1.75	0.67
1:D:51:LYS:NZ	3:D:601:ATP:O1A	2.26	0.67
1:G:231:ARG:HH11	2:U:31:ALA:HB1	1.60	0.67
1:I:455:VAL:HG13	1:I:460:GLU:HB2	1.74	0.67
1:J:291:ASP:OD1	1:J:345:ARG:NE	2.21	0.67
1:L:225:LYS:HD3	1:L:303:GLU:HG3	1.75	0.67
1:N:251:ALA:O	1:N:278:ALA:N	2.27	0.67
2:U:11:ILE:HD12	2:U:42:ALA:HB3	1.75	0.67
1:F:345:ARG:O	1:F:349:ILE:HG13	1.95	0.67
1:G:66:PHE:HB3	1:G:520:MET:HE3	1.75	0.67
1:I:427:ALA:HA	1:I:444:LEU:HD13	1.76	0.67
1:M:31:LEU:O	1:M:457:ASN:ND2	2.26	0.67
1:N:413:ALA:HB1	1:N:488:MET:HB2	1.75	0.67
1:D:219:PHE:HD2	1:D:240:VAL:HG22	1.59	0.67
1:D:365:LEU:HD13	1:D:368:ARG:HD3	1.77	0.67

Continued on next page...

For Manuscript Review

Continued from previous page...

Atom-1	Atom-2	Interatomic distance (Å)	Clash overlap (Å)
1:E:115:ASP:OD1	1:E:118:ARG:NH1	2.27	0.67
1:F:220:ILE:HG23	1:F:250:ILE:HD12	1.76	0.67
1:K:77:VAL:HG13	1:K:506:TYR:HB3	1.76	0.67
1:K:251:ALA:O	1:K:278:ALA:N	2.26	0.67
1:D:263:VAL:O	1:D:266:THR:OG1	2.13	0.67
1:E:213:VAL:N	1:E:325:ILE:O	2.27	0.67
1:L:7:LYS:HE3	1:L:15:LYS:HG3	1.76	0.67
1:M:200:LEU:HD13	1:M:254:VAL:H	1.59	0.67
2:Q:66:ILE:HD11	2:R:3:ILE:HD13	1.75	0.67
1:A:291:ASP:HB3	1:A:372:LEU:HD21	1.75	0.67
1:J:41:ASP:HA	1:J:47:PRO:HB3	1.75	0.67
1:J:177:VAL:O	1:J:393:LYS:NZ	2.28	0.67
1:K:31:LEU:O	1:K:457:ASN:ND2	2.25	0.67
1:K:419:LEU:HB3	1:K:447:MET:HB3	1.77	0.67
1:F:240:VAL:HG21	1:F:247:LEU:HD12	1.76	0.67
1:H:431:GLY:N	1:H:437:ASN:OD1	2.24	0.67
1:K:178:GLU:N	1:K:379:ILE:O	2.24	0.67
1:N:20:VAL:HG22	1:N:74:VAL:HG21	1.76	0.67
1:A:263:VAL:O	1:A:266:THR:OG1	2.11	0.67
1:C:15:LYS:HD3	1:C:18:ARG:HH21	1.60	0.67
1:D:115:ASP:OD1	1:D:118:ARG:NH1	2.26	0.67
1:D:295:LEU:HA	1:D:342:ILE:HD11	1.77	0.67
1:G:220:ILE:O	1:G:318:GLY:N	2.27	0.67
1:K:214:GLU:HG3	1:K:324:VAL:HG22	1.77	0.67
2:Q:65:VAL:HG12	2:Q:94:ILE:HG22	1.77	0.67
1:D:220:ILE:O	1:D:318:GLY:N	2.27	0.66
1:G:231:ARG:HA	1:G:234:LEU:HG	1.77	0.66
1:J:251:ALA:O	1:J:278:ALA:N	2.28	0.66
1:D:39:VAL:HG22	1:D:49:ILE:HG12	1.75	0.66
1:E:326:ASN:OD1	1:E:329:THR:N	2.22	0.66
1:G:115:ASP:OD1	1:G:118:ARG:NH1	2.26	0.66
1:J:349:ILE:HA	1:J:352:GLN:HG2	1.76	0.66
1:L:186:GLU:O	1:L:380:LYS:N	2.28	0.66
2:Q:65:VAL:HB	2:Q:91:ILE:HD12	1.78	0.66
2:R:95:VAL:HA	2:S:3:ILE:HG12	1.76	0.66
2:S:12:VAL:HG12	2:S:40:VAL:HA	1.76	0.66
1:D:415:GLY:HA2	3:D:601:ATP:H1’	1.77	0.66
1:E:51:LYS:NZ	3:E:601:ATP:O1A	2.29	0.66
1:E:339:GLU:HA	1:E:342:ILE:HD12	1.78	0.66
1:H:40:LEU:N	1:H:48:THR:O	2.28	0.66
1:H:41:ASP:HA	1:H:47:PRO:HB3	1.76	0.66

Continued on next page...

For Manuscript Review

Continued from previous page...

Atom-1	Atom-2	Interatomic distance (Å)	Clash overlap (Å)
1:I:225:LYS:HD3	1:I:303:GLU:HG3	1.75	0.66
1:K:7:LYS:HE3	1:K:15:LYS:HG3	1.76	0.66
1:K:356:ALA:O	1:K:362:ARG:NH1	2.28	0.66
1:M:251:ALA:O	1:M:278:ALA:N	2.28	0.66
1:B:220:ILE:HG23	1:B:250:ILE:HD12	1.76	0.66
1:F:175:ILE:HB	1:F:404:ARG:HH12	1.61	0.66
1:H:325:ILE:HG22	1:H:330:THR:HG23	1.77	0.66
1:H:419:LEU:HB3	1:H:447:MET:HB3	1.77	0.66
1:K:274:ALA:HB1	1:K:325:ILE:HD13	1.78	0.66
1:L:15:LYS:HD3	1:L:18:ARG:HH21	1.59	0.66
1:M:36:ARG:NH2	1:M:456:LEU:O	2.25	0.66
1:C:223:ALA:HA	1:C:301:ILE:HB	1.77	0.66
1:D:15:LYS:HD3	1:D:18:ARG:HH21	1.61	0.66
1:D:58:ARG:HA	1:D:75:LYS:HD3	1.78	0.66
1:K:128:VAL:HG13	1:K:501:ARG:HG3	1.76	0.66
1:E:223:ALA:HB1	1:E:225:LYS:HG2	1.76	0.66
1:F:248:LEU:HD22	1:F:323:VAL:HG11	1.76	0.66
1:N:249:ILE:O	1:N:276:VAL:N	2.25	0.66
2:S:73:VAL:HA	2:S:86:MET:HB3	1.77	0.66
1:A:228:SER:HA	1:A:255:GLU:HG3	1.78	0.66
1:C:115:ASP:OD1	1:C:118:ARG:NH1	2.28	0.66
1:C:326:ASN:OD1	1:C:329:THR:N	2.27	0.66
1:D:430:ARG:HH11	1:D:437:ASN:HB3	1.60	0.66
1:J:40:LEU:HD22	1:J:59:GLU:HG2	1.76	0.66
1:A:326:ASN:OD1	1:A:329:THR:N	2.28	0.66
1:E:226:LYS:HE2	1:E:253:ASP:HB3	1.77	0.66
1:G:265:ASN:OD1	2:U:26:VAL:N	2.26	0.66
1:K:104:LEU:HD21	1:K:514:MET:HG3	1.76	0.66
2:R:5:PRO:HG3	2:R:11:ILE:HG12	1.78	0.66
2:T:47:ARG:HH22	2:T:88:GLU:HB3	1.60	0.66
1:C:268:ARG:HG3	2:Q:26:VAL:HG21	1.76	0.66
1:D:265:ASN:OD1	2:R:26:VAL:N	2.27	0.66
1:G:85:ALA:HB1	1:G:499:VAL:HG22	1.78	0.66
1:G:225:LYS:HD3	1:G:309:LEU:HB2	1.78	0.66
1:G:431:GLY:HA3	1:G:436:GLN:HB3	1.77	0.66
1:I:31:LEU:O	1:I:457:ASN:ND2	2.26	0.66
2:O:78:ILE:HD11	2:O:83:VAL:HG11	1.78	0.66
2:U:67:PHE:HB3	2:U:91:ILE:HD13	1.77	0.66
1:C:172:GLU:O	1:C:366:GLN:NE2	2.28	0.66
1:C:346:VAL:HA	1:C:349:ILE:HD12	1.77	0.66
1:H:5:ASP:HB2	1:H:524:LEU:HD23	1.78	0.66

Continued on next page...

For Manuscript Review

Continued from previous page...

Atom-1	Atom-2	Interatomic distance (Å)	Clash overlap (Å)
1:H:128:VAL:HG13	1:H:501:ARG:HG3	1.78	0.66
1:J:166:MET:O	1:J:170:GLY:N	2.21	0.66
1:N:15:LYS:HD3	1:N:18:ARG:NH2	2.11	0.66
2:P:11:ILE:O	2:P:41:LEU:N	2.26	0.66
1:B:279:PRO:HG2	1:B:288:MET:HB3	1.77	0.65
1:B:479:ASN:ND2	1:B:491:MET:SD	2.69	0.65
1:F:193:MET:HB2	1:F:332:ILE:HB	1.78	0.65
1:I:81:ALA:O	1:I:85:ALA:CB	2.44	0.65
1:L:251:ALA:O	1:L:278:ALA:N	2.28	0.65
1:C:220:ILE:O	1:C:318:GLY:N	2.29	0.65
1:D:85:ALA:HB1	1:D:499:VAL:HG22	1.77	0.65
1:E:519:CYS:HB3	1:F:38:VAL:HG22	1.77	0.65
1:J:346:VAL:HG13	1:J:350:ARG:NH1	2.11	0.65
1:M:221:LEU:HB3	1:M:249:ILE:HA	1.78	0.65
1:I:20:VAL:HG22	1:I:74:VAL:HG21	1.78	0.65
2:P:47:ARG:N	2:P:55:LYS:O	2.29	0.65
2:U:73:VAL:HA	2:U:86:MET:HB3	1.78	0.65
1:B:216:GLU:OE2	1:B:322:ARG:NH1	2.28	0.65
1:G:113:PRO:HB2	1:G:516:THR:HA	1.78	0.65
1:M:455:VAL:HG13	1:M:460:GLU:HB2	1.78	0.65
1:M:517:THR:HA	1:N:37:ASN:HB2	1.78	0.65
2:Q:10:VAL:HG11	2:Q:40:VAL:HG12	1.79	0.65
2:T:17:VAL:HG22	2:T:34:LYS:HA	1.77	0.65
1:C:320:ALA:HA	1:C:336:VAL:H	1.62	0.65
1:H:427:ALA:HA	1:H:444:LEU:HD13	1.77	0.65
1:K:225:LYS:HD3	1:K:303:GLU:HG3	1.78	0.65
1:M:352:GLN:HA	1:M:355:GLU:HG3	1.78	0.65
1:B:215:LEU:HD22	1:B:246:PRO:HB3	1.78	0.65
1:G:58:ARG:HA	1:G:75:LYS:HD3	1.79	0.65
1:G:415:GLY:HA2	3:G:601:ATP:H1’	1.78	0.65
1:I:333:ILE:HG23	1:I:376:VAL:HG21	1.78	0.65
1:J:178:GLU:N	1:J:379:ILE:O	2.20	0.65
1:K:427:ALA:HA	1:K:444:LEU:HD13	1.77	0.65
1:L:139:SER:HA	1:L:171:LYS:HE3	1.78	0.65
1:A:215:LEU:HD22	1:A:246:PRO:HB3	1.79	0.65
1:C:431:GLY:HA3	1:C:436:GLN:HB3	1.79	0.65
1:E:231:ARG:HA	1:E:234:LEU:HG	1.79	0.65
1:E:349:ILE:HG23	1:E:365:LEU:HD12	1.78	0.65
1:F:213:VAL:N	1:F:325:ILE:O	2.27	0.65
1:L:197:ARG:NH2	1:L:280:GLY:O	2.30	0.65
1:L:420:ILE:HG12	1:L:448:GLU:HG2	1.77	0.65

Continued on next page...

For Manuscript Review

Continued from previous page...

Atom-1	Atom-2	Interatomic distance (Å)	Clash overlap (Å)
2:S:10:VAL:N	2:S:86:MET:O	2.15	0.65
2:T:95:VAL:HA	2:U:3:ILE:HG12	1.77	0.65
1:A:249:ILE:HB	1:A:275:ALA:HA	1.79	0.65
1:B:213:VAL:HB	1:B:325:ILE:HB	1.79	0.65
1:B:325:ILE:HG13	1:B:330:THR:HG23	1.78	0.65
1:C:356:ALA:O	1:C:362:ARG:NH2	2.30	0.65
1:K:215:LEU:HB3	1:K:246:PRO:HB2	1.78	0.65
1:K:325:ILE:HG22	1:K:330:THR:HG23	1.79	0.65
1:N:132:LYS:HE3	1:N:501:ARG:HD3	1.78	0.65
1:N:141:SER:HB3	1:N:163:ALA:HB1	1.78	0.65
1:H:250:ILE:HG12	1:H:276:VAL:HB	1.78	0.65
1:D:421:ARG:NH2	1:D:476:TYR:O	2.20	0.65
1:G:326:ASN:OD1	1:G:329:THR:N	2.23	0.65
1:H:251:ALA:O	1:H:278:ALA:N	2.29	0.65
1:L:31:LEU:O	1:L:457:ASN:ND2	2.23	0.65
1:N:81:ALA:O	1:N:85:ALA:HB2	1.96	0.65
1:N:479:ASN:N	1:N:484:GLU:O	2.29	0.65
2:O:8:ASP:HA	2:O:57:LEU:HD11	1.77	0.65
1:D:213:VAL:N	1:D:325:ILE:O	2.27	0.64
1:E:102:GLU:HB2	1:E:442:VAL:HG13	1.78	0.64
1:E:353:ILE:HG23	1:E:362:ARG:HB2	1.79	0.64
2:R:66:ILE:HD11	2:S:3:ILE:HD13	1.78	0.64
1:C:230:ILE:HA	1:C:233:MET:HE2	1.80	0.64
1:F:339:GLU:O	1:F:343:GLN:NE2	2.30	0.64
1:K:175:ILE:HA	1:K:377:ALA:HB3	1.78	0.64
1:K:220:ILE:HD11	1:K:250:ILE:HD12	1.77	0.64
2:Q:15:LYS:HE3	2:Q:64:ILE:HG23	1.79	0.64
2:U:11:ILE:O	2:U:41:LEU:N	2.21	0.64
1:B:268:ARG:HG3	2:P:26:VAL:HG21	1.78	0.64
1:D:71:ALA:HA	1:D:74:VAL:HG12	1.79	0.64
1:D:223:ALA:HA	1:D:301:ILE:HB	1.77	0.64
1:F:115:ASP:OD1	1:F:118:ARG:NH1	2.29	0.64
1:G:224:ASP:OD1	1:G:285:ARG:NH1	2.30	0.64
1:I:353:ILE:HA	1:I:362:ARG:HH22	1.61	0.64
1:I:421:ARG:NH2	1:I:476:TYR:O	2.29	0.64
1:J:420:ILE:HG12	1:J:448:GLU:HG2	1.79	0.64
1:L:349:ILE:HD13	1:L:368:ARG:HB3	1.79	0.64
1:N:419:LEU:HB3	1:N:447:MET:HB3	1.80	0.64
2:S:27:LEU:HB3	2:S:31:ALA:HB3	1.78	0.64
1:F:220:ILE:O	1:F:318:GLY:N	2.30	0.64
2:S:95:VAL:HA	2:T:3:ILE:HG12	1.79	0.64

Continued on next page...

For Manuscript Review

Continued from previous page...

Atom-1	Atom-2	Interatomic distance (Å)	Clash overlap (Å)
1:C:51:LYS:NZ	3:C:601:ATP:O1A	2.30	0.64
1:E:220:ILE:O	1:E:318:GLY:N	2.30	0.64
1:J:421:ARG:NH2	1:J:476:TYR:O	2.26	0.64
1:L:222:LEU:HD13	1:L:293:ALA:HA	1.79	0.64
1:M:18:ARG:NE	1:M:67:GLU:OE2	2.30	0.64
2:R:38:GLY:HA3	2:R:67:PHE:HE1	1.62	0.64
1:A:148:GLY:HA2	1:A:399:ALA:HB1	1.80	0.64
1:B:172:GLU:O	1:B:366:GLN:NE2	2.31	0.64
1:J:233:MET:HG3	1:J:237:LEU:HG	1.80	0.64
2:O:94:ILE:HG23	2:P:6:LEU:HD21	1.78	0.64
2:P:14:ARG:NH2	2:P:69:ASP:OD2	2.31	0.64
2:Q:38:GLY:HA3	2:Q:67:PHE:HE1	1.61	0.64
2:R:12:VAL:HG22	2:R:84:LEU:HB2	1.80	0.64
1:B:220:ILE:O	1:B:318:GLY:N	2.29	0.64
1:G:20:VAL:HG22	1:G:74:VAL:HB	1.77	0.64
1:L:291:ASP:OD1	1:L:345:ARG:NE	2.27	0.64
1:N:326:ASN:N	1:N:329:THR:O	2.23	0.64
2:O:73:VAL:HA	2:O:86:MET:HB3	1.78	0.64
1:A:279:PRO:HG2	1:A:288:MET:HB3	1.79	0.64
1:D:279:PRO:HG2	1:D:288:MET:HB3	1.80	0.64
1:E:421:ARG:HH12	1:E:470:LYS:HA	1.63	0.64
1:F:349:ILE:HG22	1:F:365:LEU:HB3	1.80	0.64
1:J:62:LEU:HB2	1:J:68:ASN:HB2	1.80	0.64
1:N:291:ASP:OD1	1:N:345:ARG:NE	2.31	0.64
1:D:270:ILE:HG21	2:R:25:ILE:HA	1.80	0.64
1:F:356:ALA:O	1:F:362:ARG:NH2	2.31	0.64
1:H:77:VAL:HG13	1:H:506:TYR:HB3	1.78	0.64
1:M:7:LYS:HE3	1:M:15:LYS:HG3	1.79	0.64
2:P:37:ARG:HH22	2:Q:78:ILE:HG22	1.62	0.64
2:P:59:VAL:HG21	2:P:91:ILE:HG21	1.80	0.64
1:E:263:VAL:O	1:E:266:THR:OG1	2.10	0.64
1:F:320:ALA:HA	1:F:336:VAL:H	1.63	0.64
1:F:431:GLY:HA3	1:F:436:GLN:HB3	1.80	0.64
1:K:421:ARG:NH2	1:K:476:TYR:O	2.25	0.64
1:L:324:VAL:HB	1:L:331:THR:HB	1.80	0.64
1:A:349:ILE:HG23	1:A:365:LEU:HD12	1.80	0.63
1:A:365:LEU:HD13	1:A:368:ARG:HD3	1.80	0.63
1:E:365:LEU:HD13	1:E:368:ARG:HD3	1.81	0.63
1:I:326:ASN:N	1:I:329:THR:O	2.30	0.63
1:I:393:LYS:NZ	1:I:397:GLU:OE2	2.31	0.63
1:J:249:ILE:O	1:J:276:VAL:N	2.27	0.63

Continued on next page...

For Manuscript Review

Continued from previous page...

Atom-1	Atom-2	Interatomic distance (Å)	Clash overlap (Å)
1:K:81:ALA:O	1:K:85:ALA:CB	2.45	0.63
1:L:322:ARG:O	1:L:333:ILE:N	2.30	0.63
1:B:263:VAL:O	1:B:266:THR:OG1	2.13	0.63
1:F:15:LYS:HB3	1:F:66:PHE:HB2	1.80	0.63
1:F:203:TYR:HB2	1:F:263:VAL:HB	1.80	0.63
1:G:261:THR:O	1:G:265:ASN:ND2	2.31	0.63
1:H:61:GLU:OE2	1:H:72:GLN:NE2	2.31	0.63
1:I:40:LEU:N	1:I:48:THR:O	2.30	0.63
1:I:251:ALA:O	1:I:278:ALA:N	2.29	0.63
1:K:213:VAL:HB	1:K:325:ILE:HG12	1.79	0.63
1:L:455:VAL:HG13	1:L:460:GLU:HB2	1.79	0.63
1:M:197:ARG:NH2	1:M:280:GLY:O	2.31	0.63
1:F:186:GLU:O	1:F:380:LYS:N	2.26	0.63
1:G:479:ASN:N	1:G:484:GLU:O	2.27	0.63
1:I:41:ASP:HA	1:I:47:PRO:HB3	1.80	0.63
1:J:455:VAL:HG21	1:J:465:VAL:HG11	1.79	0.63
1:N:200:LEU:HD13	1:N:254:VAL:H	1.64	0.63
2:S:8:ASP:HA	2:S:57:LEU:HD11	1.78	0.63
2:T:8:ASP:HB3	2:T:47:ARG:HG3	1.80	0.63
1:A:31:LEU:HB2	1:A:90:THR:HG21	1.81	0.63
1:A:115:ASP:OD1	1:A:118:ARG:NH1	2.29	0.63
1:I:205:ILE:HA	1:I:213:VAL:HG22	1.79	0.63
1:J:214:GLU:HG3	1:J:324:VAL:HG22	1.80	0.63
1:M:225:LYS:HD3	1:M:303:GLU:HG3	1.80	0.63
1:N:427:ALA:HA	1:N:444:LEU:HD13	1.81	0.63
1:D:261:THR:O	1:D:265:ASN:ND2	2.31	0.63
1:D:339:GLU:HA	1:D:342:ILE:HD12	1.81	0.63
1:F:31:LEU:HB2	1:F:90:THR:HG21	1.81	0.63
1:I:197:ARG:HE	1:I:279:PRO:HA	1.63	0.63
1:N:205:ILE:HA	1:N:213:VAL:HG22	1.80	0.63
2:O:6:LEU:HD11	2:U:94:ILE:HG13	1.81	0.63
1:J:240:VAL:HG21	1:J:247:LEU:HD13	1.79	0.63
1:L:479:ASN:N	1:L:484:GLU:O	2.31	0.63
1:M:291:ASP:OD1	1:M:345:ARG:NE	2.32	0.63
2:O:14:ARG:NH2	2:O:69:ASP:OD2	2.31	0.63
1:E:233:MET:HB3	1:E:237:LEU:HD23	1.81	0.63
1:F:326:ASN:OD1	1:F:329:THR:N	2.28	0.63
1:G:230:ILE:HA	1:G:233:MET:HE2	1.81	0.63
1:G:320:ALA:HA	1:G:336:VAL:H	1.63	0.63
1:I:214:GLU:HG3	1:I:324:VAL:HG22	1.81	0.63
1:K:64:ASP:HB3	1:K:67:GLU:HB2	1.80	0.63

Continued on next page...

For Manuscript Review

Continued from previous page...

Atom-1	Atom-2	Interatomic distance (Å)	Clash overlap (Å)
2:Q:10:VAL:N	2:Q:86:MET:O	2.29	0.63
1:D:325:ILE:HG13	1:D:330:THR:HG23	1.79	0.63
1:G:350:ARG:HA	1:G:353:ILE:HD12	1.81	0.63
1:I:200:LEU:HD21	1:I:277:LYS:HB2	1.79	0.63
1:I:501:ARG:NH1	1:I:505:GLN:OE1	2.32	0.63
1:J:266:THR:HG22	1:J:273:VAL:H	1.63	0.63
1:M:325:ILE:HA	1:M:330:THR:HA	1.80	0.63
1:B:130:GLU:HB2	1:B:422:VAL:HG13	1.79	0.62
1:B:231:ARG:HA	1:B:234:LEU:HG	1.81	0.62
1:E:342:ILE:HG12	1:E:372:LEU:HD11	1.79	0.62
1:H:7:LYS:HE3	1:H:15:LYS:HG3	1.80	0.62
2:P:65:VAL:HB	2:P:91:ILE:HG23	1.81	0.62
2:R:13:LYS:HB2	2:R:41:LEU:HD11	1.81	0.62
1:A:203:TYR:HB2	1:A:263:VAL:HB	1.79	0.62
1:H:20:VAL:HG22	1:H:74:VAL:HG21	1.80	0.62
1:I:166:MET:O	1:I:170:GLY:N	2.32	0.62
1:M:411:VAL:HA	1:M:496:PRO:HA	1.80	0.62
1:M:479:ASN:N	1:M:484:GLU:O	2.27	0.62
2:P:11:ILE:HD12	2:P:42:ALA:HB3	1.81	0.62
2:S:68:ASN:N	2:S:90:ASP:O	2.31	0.62
1:F:339:GLU:HA	1:F:342:ILE:HD12	1.80	0.62
1:A:222:LEU:O	1:A:301:ILE:N	2.20	0.62
1:B:190:VAL:O	1:B:376:VAL:N	2.24	0.62
1:G:213:VAL:N	1:G:325:ILE:O	2.29	0.62
1:H:166:MET:HB3	1:H:175:ILE:HD11	1.81	0.62
1:H:235:PRO:HG3	1:H:310:GLU:HA	1.81	0.62
1:I:222:LEU:HD13	1:I:293:ALA:HA	1.81	0.62
1:L:21:ASN:HA	1:L:97:GLN:HE21	1.64	0.62
1:D:421:ARG:HH12	1:D:470:LYS:HA	1.64	0.62
1:E:31:LEU:O	1:E:457:ASN:ND2	2.27	0.62
1:E:58:ARG:HA	1:E:75:LYS:HD3	1.80	0.62
1:H:139:SER:HA	1:H:171:LYS:HE3	1.82	0.62
1:I:477:GLY:N	1:I:486:GLY:O	2.31	0.62
1:K:36:ARG:NH2	1:K:456:LEU:O	2.31	0.62
1:L:205:ILE:HA	1:L:213:VAL:HG22	1.81	0.62
1:M:221:LEU:HD23	1:M:249:ILE:HG23	1.80	0.62
1:M:455:VAL:HG21	1:M:465:VAL:HG11	1.80	0.62
2:Q:15:LYS:HG3	2:Q:38:GLY:HA2	1.81	0.62
1:A:175:ILE:HB	1:A:404:ARG:HH12	1.65	0.62
1:C:27:VAL:HG12	1:C:90:THR:HG23	1.82	0.62
1:J:427:ALA:HA	1:J:444:LEU:HD13	1.81	0.62

Continued on next page...

For Manuscript Review

Continued from previous page...

Atom-1	Atom-2	Interatomic distance (Å)	Clash overlap (Å)
1:M:155:ASP:OD2	1:M:395:ARG:NH1	2.33	0.62
2:P:8:ASP:HA	2:P:57:LEU:HD11	1.80	0.62
2:U:47:ARG:N	2:U:55:LYS:O	2.32	0.62
1:A:220:ILE:O	1:A:318:GLY:N	2.32	0.62
1:A:397:GLU:O	1:A:401:HIS:ND1	2.32	0.62
1:B:240:VAL:HG21	1:B:247:LEU:HD12	1.82	0.62
1:E:265:ASN:OD1	2:S:26:VAL:N	2.30	0.62
1:G:240:VAL:HG11	1:G:247:LEU:HB2	1.80	0.62
1:G:325:ILE:HG13	1:G:330:THR:HG23	1.82	0.62
1:H:200:LEU:HD13	1:H:254:VAL:H	1.65	0.62
1:I:111:MET:HG3	1:I:116:LEU:HD11	1.82	0.62
1:M:65:LYS:O	1:M:69:MET:HG3	2.00	0.62
1:M:213:VAL:HB	1:M:325:ILE:HG12	1.81	0.62
1:N:365:LEU:HA	1:N:368:ARG:HG3	1.82	0.62
2:T:68:ASN:N	2:T:90:ASP:O	2.30	0.62
1:C:240:VAL:O	1:C:244:GLY:N	2.32	0.62
1:D:301:ILE:HD11	1:D:316:ASP:HB3	1.82	0.62
1:F:469:VAL:HG13	1:F:477:GLY:HA2	1.82	0.62
1:H:104:LEU:HD21	1:H:514:MET:HG3	1.81	0.62
1:I:197:ARG:NH2	1:I:280:GLY:O	2.32	0.62
1:K:117:LYS:NZ	1:K:121:ASP:OD2	2.32	0.62
1:M:501:ARG:NH1	1:M:505:GLN:OE1	2.33	0.62
1:F:213:VAL:HG11	1:F:274:ALA:HB2	1.82	0.62
1:J:322:ARG:O	1:J:333:ILE:N	2.29	0.62
1:N:82:ASN:HB2	1:N:89:THR:HG22	1.80	0.62
1:A:28:LYS:HE2	1:A:94:VAL:HG22	1.80	0.62
1:A:193:MET:HE1	1:A:372:LEU:HA	1.81	0.62
1:C:58:ARG:HA	1:C:75:LYS:HD3	1.80	0.62
1:C:213:VAL:N	1:C:325:ILE:O	2.27	0.62
1:F:249:ILE:HB	1:F:275:ALA:HA	1.81	0.62
1:G:186:GLU:O	1:G:380:LYS:N	2.30	0.62
1:I:240:VAL:HG21	1:I:247:LEU:HD13	1.80	0.62
1:K:291:ASP:OD1	1:K:345:ARG:NE	2.33	0.62
1:K:479:ASN:N	1:K:484:GLU:O	2.31	0.62
1:M:274:ALA:HB1	1:M:325:ILE:HD13	1.81	0.62
2:O:11:ILE:O	2:O:41:LEU:N	2.32	0.62
2:T:11:ILE:HG13	2:T:85:ILE:HD13	1.82	0.62
1:D:320:ALA:HA	1:D:336:VAL:H	1.65	0.61
1:G:205:ILE:HA	1:G:213:VAL:HG22	1.81	0.61
1:G:220:ILE:N	1:G:318:GLY:O	2.24	0.61
1:L:186:GLU:N	1:L:380:LYS:O	2.33	0.61

Continued on next page...

For Manuscript Review

Continued from previous page...

Atom-1	Atom-2	Interatomic distance (Å)	Clash overlap (Å)
1:L:200:LEU:HD13	1:L:254:VAL:H	1.64	0.61
1:L:325:ILE:HA	1:L:330:THR:HA	1.81	0.61
1:C:279:PRO:HG2	1:C:288:MET:HB3	1.82	0.61
1:D:20:VAL:HG13	1:D:74:VAL:HG21	1.81	0.61
1:J:479:ASN:O	1:J:483:GLU:N	2.34	0.61
1:N:195:PHE:HZ	1:N:250:ILE:HD13	1.66	0.61
1:N:215:LEU:HB3	1:N:246:PRO:HB2	1.81	0.61
1:N:333:ILE:HG12	1:N:376:VAL:HG11	1.81	0.61
2:U:49:LEU:HD12	2:U:53:GLU:HB2	1.82	0.61
1:C:177:VAL:HG23	1:C:400:LEU:HD22	1.82	0.61
1:E:325:ILE:HG13	1:E:330:THR:HG23	1.82	0.61
1:I:82:ASN:HB2	1:I:89:THR:HG22	1.81	0.61
1:J:15:LYS:NZ	1:J:64:ASP:OD2	2.27	0.61
1:J:31:LEU:O	1:J:457:ASN:ND2	2.24	0.61
1:K:197:ARG:NH2	1:K:280:GLY:O	2.33	0.61
1:M:197:ARG:HE	1:M:279:PRO:HA	1.65	0.61
1:M:200:LEU:HD21	1:M:277:LYS:HB2	1.82	0.61
1:N:197:ARG:NH2	1:N:280:GLY:O	2.33	0.61
2:S:66:ILE:HG21	2:T:76:GLU:HG2	1.82	0.61
1:A:220:ILE:N	1:A:318:GLY:O	2.23	0.61
1:H:185:ASP:OD1	1:H:382:GLY:N	2.31	0.61
1:I:81:ALA:HB1	1:I:503:ALA:HA	1.81	0.61
1:I:479:ASN:ND2	1:I:491:MET:HG3	2.14	0.61
1:K:322:ARG:HB2	1:K:333:ILE:HB	1.81	0.61
1:N:325:ILE:HG22	1:N:330:THR:HG23	1.83	0.61
1:N:455:VAL:HG13	1:N:460:GLU:HB2	1.83	0.61
2:S:37:ARG:HH22	2:T:78:ILE:HG22	1.65	0.61
1:F:58:ARG:HA	1:F:75:LYS:HD3	1.81	0.61
1:H:266:THR:HG22	1:H:273:VAL:H	1.65	0.61
1:J:40:LEU:N	1:J:48:THR:O	2.31	0.61
1:J:213:VAL:HB	1:J:325:ILE:HG12	1.83	0.61
1:K:200:LEU:HD13	1:K:254:VAL:H	1.65	0.61
1:C:124:VAL:HG21	1:C:508:ALA:HB2	1.81	0.61
1:C:495:ASP:OD2	3:C:601:ATP:O2'	2.15	0.61
1:D:393:LYS:NZ	1:D:397:GLU:OE2	2.30	0.61
1:I:266:THR:HG22	1:I:273:VAL:H	1.64	0.61
1:J:215:LEU:HB3	1:J:246:PRO:HB2	1.81	0.61
1:K:39:VAL:HG13	1:K:49:ILE:HG12	1.81	0.61
1:K:322:ARG:O	1:K:333:ILE:N	2.28	0.61
1:M:322:ARG:O	1:M:333:ILE:N	2.30	0.61
2:Q:40:VAL:HG23	2:Q:62:GLY:H	1.66	0.61

Continued on next page...

For Manuscript Review

Continued from previous page...

Atom-1	Atom-2	Interatomic distance (Å)	Clash overlap (Å)
2:Q:47:ARG:NH2	2:Q:88:GLU:HB3	2.16	0.61
1:A:144:ILE:HG23	1:A:403:THR:HG21	1.82	0.61
1:E:220:ILE:HG23	1:E:250:ILE:HD12	1.83	0.61
1:G:469:VAL:HG13	1:G:477:GLY:HA2	1.82	0.61
1:H:81:ALA:O	1:H:85:ALA:HB3	2.00	0.61
1:J:322:ARG:HB2	1:J:333:ILE:HB	1.81	0.61
1:M:322:ARG:HB2	1:M:333:ILE:HB	1.82	0.61
1:A:519:CYS:HB3	1:B:38:VAL:HG22	1.80	0.61
1:F:295:LEU:HA	1:F:342:ILE:HD11	1.83	0.61
1:C:220:ILE:HG23	1:C:250:ILE:HD12	1.81	0.61
1:C:248:LEU:HD22	1:C:323:VAL:HG11	1.83	0.61
1:D:227:ILE:HD12	1:D:254:VAL:HG22	1.80	0.61
1:E:415:GLY:HA2	3:E:601:ATP:H1’	1.81	0.61
1:G:169:VAL:HB	1:G:377:ALA:HB2	1.82	0.61
1:I:325:ILE:HA	1:I:330:THR:HA	1.81	0.61
1:K:455:VAL:HG13	1:K:460:GLU:HB2	1.81	0.61
1:N:155:ASP:OD2	1:N:395:ARG:NH1	2.34	0.61
1:A:251:ALA:O	1:A:278:ALA:N	2.33	0.61
1:A:431:GLY:HA3	1:A:436:GLN:HB3	1.82	0.61
1:C:231:ARG:HA	1:C:234:LEU:HG	1.83	0.61
1:H:38:VAL:HG22	1:N:519:CYS:HB3	1.82	0.61
1:H:339:GLU:HA	1:H:342:ILE:HD12	1.83	0.61
1:H:413:ALA:HB1	1:H:488:MET:HB2	1.83	0.61
1:I:218:PRO:HB3	1:I:246:PRO:HG2	1.83	0.61
1:J:122:LYS:NZ	1:J:432:GLN:OE1	2.34	0.61
1:K:32:GLY:HA3	1:K:454:ILE:HG23	1.83	0.61
1:L:223:ALA:HA	1:L:301:ILE:HB	1.82	0.61
1:M:38:VAL:O	1:M:50:THR:N	2.33	0.61
1:M:350:ARG:HA	1:M:353:ILE:HD12	1.83	0.61
2:U:15:LYS:HG3	2:U:38:GLY:HA2	1.83	0.61
1:B:27:VAL:HG12	1:B:90:THR:HG23	1.82	0.60
1:G:421:ARG:NH2	1:G:476:TYR:O	2.22	0.60
1:H:205:ILE:HA	1:H:213:VAL:HG22	1.81	0.60
1:I:174:VAL:HB	1:I:376:VAL:HG12	1.82	0.60
1:I:200:LEU:HD13	1:I:254:VAL:H	1.65	0.60
1:L:81:ALA:O	1:L:85:ALA:HB2	2.01	0.60
1:M:349:ILE:HG21	1:M:368:ARG:HB2	1.83	0.60
1:B:213:VAL:HG11	1:B:274:ALA:HB2	1.81	0.60
1:B:420:ILE:HG12	1:B:448:GLU:HG2	1.82	0.60
1:D:216:GLU:OE2	1:D:322:ARG:NH1	2.34	0.60
1:G:220:ILE:HG23	1:G:250:ILE:HD12	1.83	0.60

Continued on next page...

For Manuscript Review

Continued from previous page...

Atom-1	Atom-2	Interatomic distance (Å)	Clash overlap (Å)
1:H:174:VAL:HG11	1:H:376:VAL:HG12	1.82	0.60
1:H:291:ASP:OD1	1:H:345:ARG:NE	2.32	0.60
1:L:84:ALA:O	1:L:498:LYS:NZ	2.27	0.60
1:N:14:VAL:HB	1:N:18:ARG:HH12	1.65	0.60
2:O:11:ILE:HG22	2:O:41:LEU:HB2	1.83	0.60
1:A:230:ILE:HA	1:A:233:MET:HE2	1.83	0.60
1:C:519:CYS:HB3	1:D:38:VAL:HG22	1.82	0.60
1:K:205:ILE:HA	1:K:213:VAL:HG22	1.82	0.60
1:L:144:ILE:HG12	1:L:166:MET:HE3	1.83	0.60
2:P:12:VAL:HG12	2:P:40:VAL:HA	1.83	0.60
2:T:11:ILE:HD12	2:T:42:ALA:HB3	1.83	0.60
1:B:69:MET:HB2	1:C:47:PRO:HG2	1.83	0.60
1:C:20:VAL:HG22	1:C:74:VAL:HB	1.83	0.60
1:D:177:VAL:HG23	1:D:400:LEU:HD22	1.84	0.60
1:E:158:VAL:HG11	1:E:396:VAL:HA	1.84	0.60
1:F:213:VAL:HB	1:F:325:ILE:HB	1.84	0.60
1:L:406:ALA:HB2	1:L:496:PRO:HG3	1.83	0.60
1:N:76:GLU:HG2	1:N:80:LYS:HE3	1.83	0.60
1:B:320:ALA:HA	1:B:336:VAL:H	1.66	0.60
1:E:152:ALA:HB2	1:E:399:ALA:HB2	1.83	0.60
1:E:205:ILE:HA	1:E:213:VAL:HG22	1.84	0.60
1:H:81:ALA:HB1	1:H:503:ALA:HA	1.83	0.60
1:H:274:ALA:HB1	1:H:325:ILE:HD13	1.82	0.60
1:M:433:ASN:H	1:M:436:GLN:HB2	1.67	0.60
1:M:477:GLY:N	1:M:486:GLY:O	2.34	0.60
1:A:85:ALA:HB1	1:A:499:VAL:HG22	1.83	0.60
1:B:177:VAL:HG23	1:B:400:LEU:HD22	1.84	0.60
1:B:231:ARG:NH2	2:P:31:ALA:O	2.34	0.60
1:E:264:VAL:O	1:E:268:ARG:HG2	2.02	0.60
1:H:349:ILE:HD13	1:H:368:ARG:HB3	1.84	0.60
1:K:386:GLU:O	1:K:389:MET:HB2	2.01	0.60
1:L:501:ARG:NH1	1:L:505:GLN:OE1	2.34	0.60
2:O:12:VAL:O	2:O:84:LEU:N	2.34	0.60
2:T:73:VAL:HA	2:T:86:MET:HB3	1.83	0.60
1:A:186:GLU:HG3	1:A:380:LYS:HE2	1.84	0.60
1:B:232:GLU:HA	1:B:310:GLU:HG3	1.84	0.60
1:L:137:PRO:HA	1:L:410:GLY:HA2	1.84	0.60
1:M:427:ALA:HA	1:M:444:LEU:HD13	1.83	0.60
1:N:240:VAL:HG21	1:N:247:LEU:HD13	1.83	0.60
2:R:8:ASP:HA	2:R:57:LEU:HD11	1.83	0.60
1:A:27:VAL:HG12	1:A:90:THR:HG23	1.83	0.60

Continued on next page...

For Manuscript Review

Continued from previous page...

Atom-1	Atom-2	Interatomic distance (Å)	Clash overlap (Å)
1:B:85:ALA:HB1	1:B:499:VAL:HG22	1.82	0.60
1:B:346:VAL:HB	1:B:369:VAL:HG22	1.83	0.60
1:C:452:ARG:HH12	1:C:463:SER:HA	1.65	0.60
1:D:262:LEU:HD22	1:D:273:VAL:HG21	1.84	0.60
1:H:202:PRO:HG2	1:I:384:ALA:HA	1.83	0.60
1:J:179:ASP:HA	1:J:381:VAL:HG22	1.84	0.60
1:M:124:VAL:HG13	1:M:504:LEU:HG	1.84	0.60
1:N:421:ARG:NH2	1:N:476:TYR:O	2.26	0.60
1:A:209:GLU:HG2	1:A:210:THR:HG23	1.83	0.60
1:A:261:THR:O	1:A:265:ASN:ND2	2.33	0.60
1:A:346:VAL:HB	1:A:369:VAL:HG22	1.82	0.60
1:B:251:ALA:O	1:B:278:ALA:N	2.35	0.60
1:D:213:VAL:HB	1:D:325:ILE:HB	1.83	0.60
1:E:114:MET:SD	7:F:701:HOH:O	2.56	0.60
1:F:197:ARG:O	1:F:330:THR:OG1	2.12	0.60
1:F:350:ARG:HA	1:F:353:ILE:HD12	1.83	0.60
1:G:519:CYS:SG	1:G:520:MET:N	2.75	0.60
1:M:215:LEU:HB3	1:M:246:PRO:HB2	1.81	0.60
1:M:302:SER:HB2	1:M:304:GLU:HG2	1.83	0.60
2:R:46:GLY:HA2	2:R:57:LEU:HD12	1.84	0.60
1:A:213:VAL:N	1:A:325:ILE:O	2.29	0.60
1:A:393:LYS:NZ	1:A:397:GLU:OE2	2.26	0.60
1:B:230:ILE:HA	1:B:233:MET:HE2	1.83	0.60
1:C:393:LYS:NZ	1:C:397:GLU:OE2	2.30	0.60
1:E:232:GLU:HA	1:E:310:GLU:HG3	1.84	0.60
1:H:195:PHE:HZ	1:H:250:ILE:HD13	1.67	0.60
1:J:77:VAL:HG13	1:J:506:TYR:HB3	1.84	0.60
1:K:270:ILE:HG22	1:K:271:VAL:HG23	1.83	0.60
1:B:221:LEU:HB2	1:B:317:LEU:HD22	1.83	0.59
1:C:414:GLY:H	1:C:488:MET:HB3	1.67	0.59
1:D:346:VAL:HB	1:D:369:VAL:HG22	1.84	0.59
1:H:323:VAL:HG12	1:H:332:ILE:HG22	1.84	0.59
1:I:349:ILE:HD13	1:I:368:ARG:HB3	1.84	0.59
1:I:455:VAL:HG21	1:I:465:VAL:HG11	1.83	0.59
1:K:266:THR:HG22	1:K:273:VAL:H	1.66	0.59
1:K:295:LEU:HD23	1:K:342:ILE:HG12	1.82	0.59
1:K:325:ILE:HA	1:K:330:THR:HA	1.84	0.59
1:A:522:THR:HG22	1:B:41:ASP:HB2	1.83	0.59
1:C:421:ARG:HH12	1:C:470:LYS:HA	1.65	0.59
1:F:252:GLU:OE2	1:F:285:ARG:NH1	2.34	0.59
1:I:39:VAL:HG13	1:I:49:ILE:HG12	1.84	0.59

Continued on next page...

For Manuscript Review

Continued from previous page...

Atom-1	Atom-2	Interatomic distance (Å)	Clash overlap (Å)
1:J:200:LEU:HD13	1:J:254:VAL:H	1.66	0.59
1:J:420:ILE:HD12	1:J:451:LEU:HD13	1.83	0.59
1:A:264:VAL:O	1:A:268:ARG:HG2	2.03	0.59
1:A:287:ALA:HA	1:A:345:ARG:HH21	1.67	0.59
1:E:27:VAL:HG12	1:E:90:THR:HG23	1.83	0.59
1:G:177:VAL:HG23	1:G:400:LEU:HD22	1.82	0.59
1:H:215:LEU:HB3	1:H:246:PRO:HB2	1.84	0.59
1:H:365:LEU:HD23	1:H:368:ARG:HE	1.68	0.59
1:M:39:VAL:HA	1:M:49:ILE:HA	1.85	0.59
1:N:128:VAL:HG13	1:N:501:ARG:HG3	1.84	0.59
1:B:349:ILE:HG23	1:B:365:LEU:HB3	1.83	0.59
1:C:149:THR:OG1	1:C:156:GLU:HA	2.02	0.59
1:F:264:VAL:O	1:F:268:ARG:HG2	2.02	0.59
1:K:433:ASN:H	1:K:436:GLN:HB2	1.67	0.59
1:M:141:SER:HB3	1:M:163:ALA:HB1	1.83	0.59
1:N:325:ILE:HA	1:N:330:THR:HA	1.84	0.59
2:S:11:ILE:HG22	2:S:41:LEU:HB2	1.84	0.59
1:A:223:ALA:HB1	1:A:225:LYS:HG2	1.84	0.59
1:B:20:VAL:HG22	1:B:74:VAL:HB	1.83	0.59
1:C:144:ILE:HG23	1:C:403:THR:HB	1.84	0.59
1:D:197:ARG:O	1:D:330:THR:OG1	2.14	0.59
1:D:220:ILE:HG23	1:D:250:ILE:HD12	1.83	0.59
1:E:190:VAL:N	1:E:376:VAL:O	2.29	0.59
1:E:231:ARG:NH2	2:S:31:ALA:O	2.34	0.59
1:F:27:VAL:HG12	1:F:90:THR:HG23	1.85	0.59
1:F:193:MET:HE1	1:F:372:LEU:HA	1.83	0.59
1:G:262:LEU:HD22	1:G:273:VAL:HG21	1.84	0.59
1:H:197:ARG:NH2	1:H:280:GLY:O	2.35	0.59
1:I:419:LEU:HD22	1:I:447:MET:HG3	1.84	0.59
1:K:82:ASN:HB2	1:K:89:THR:HG22	1.83	0.59
1:M:240:VAL:HG21	1:M:247:LEU:HD13	1.83	0.59
1:N:194:GLN:HG3	1:N:331:THR:HB	1.83	0.59
2:P:46:GLY:HA2	2:P:57:LEU:HD12	1.83	0.59
2:T:46:GLY:HA2	2:T:57:LEU:HD12	1.83	0.59
2:T:65:VAL:HB	2:T:91:ILE:HG23	1.84	0.59
1:B:144:ILE:HG23	1:B:403:THR:HB	1.85	0.59
1:C:231:ARG:HH21	2:Q:31:ALA:HB1	1.67	0.59
1:D:27:VAL:HG12	1:D:90:THR:HG23	1.85	0.59
1:E:251:ALA:O	1:E:278:ALA:N	2.34	0.59
1:F:150:ILE:HD11	1:F:493:ILE:HG12	1.85	0.59
1:F:452:ARG:HH12	1:F:463:SER:HA	1.68	0.59

Continued on next page...

For Manuscript Review

Continued from previous page...

Atom-1	Atom-2	Interatomic distance (Å)	Clash overlap (Å)
1:I:420:ILE:HG12	1:I:448:GLU:HG2	1.84	0.59
1:M:138:CYS:HB3	1:M:406:ALA:HB1	1.84	0.59
1:M:193:MET:HG2	1:M:295:LEU:HD13	1.82	0.59
2:Q:40:VAL:HG13	2:Q:65:VAL:HG21	1.85	0.59
1:B:519:CYS:HB3	1:C:38:VAL:HG22	1.85	0.59
1:G:221:LEU:HB2	1:G:317:LEU:HD22	1.84	0.59
1:L:128:VAL:HG13	1:L:501:ARG:HG3	1.84	0.59
1:L:185:ASP:HA	1:L:381:VAL:HA	1.85	0.59
1:B:230:ILE:HD11	1:B:258:ALA:HA	1.85	0.59
1:G:102:GLU:HB2	1:G:442:VAL:HG13	1.84	0.59
1:H:82:ASN:HB2	1:H:89:THR:HG22	1.85	0.59
1:H:455:VAL:HG13	1:H:460:GLU:HB2	1.84	0.59
1:I:77:VAL:HG13	1:I:506:TYR:HB3	1.84	0.59
1:J:128:VAL:HG13	1:J:501:ARG:HG3	1.85	0.59
1:M:192:GLY:HA3	1:M:376:VAL:HG13	1.84	0.59
1:M:266:THR:HG22	1:M:273:VAL:H	1.68	0.59
1:E:124:VAL:HG21	1:E:508:ALA:HB2	1.84	0.59
1:E:346:VAL:HG13	1:E:372:LEU:HD23	1.83	0.59
1:I:295:LEU:HD23	1:I:342:ILE:HG12	1.84	0.59
1:K:196:ASP:HA	1:K:329:THR:HG22	1.85	0.59
1:M:291:ASP:HA	1:M:345:ARG:HG2	1.85	0.59
1:C:261:THR:HG21	2:Q:27:LEU:HD13	1.84	0.59
1:L:148:GLY:HA2	1:L:399:ALA:HB1	1.85	0.59
1:M:13:ARG:HD3	1:M:514:MET:HE3	1.85	0.59
2:Q:91:ILE:O	2:R:9:ARG:NH1	2.35	0.59
2:S:11:ILE:HD12	2:S:42:ALA:HB3	1.85	0.59
1:B:287:ALA:HA	1:B:345:ARG:HH21	1.68	0.58
1:C:12:ALA:HA	1:C:520:MET:HE3	1.84	0.58
1:I:322:ARG:O	1:I:333:ILE:N	2.30	0.58
1:K:168:LYS:HG2	1:K:189:VAL:HG13	1.85	0.58
1:L:82:ASN:HB2	1:L:89:THR:HG22	1.85	0.58
1:L:155:ASP:OD2	1:L:395:ARG:NH1	2.35	0.58
1:L:266:THR:HG22	1:L:273:VAL:H	1.68	0.58
2:O:46:GLY:HA2	2:O:57:LEU:HD12	1.84	0.58
2:Q:46:GLY:HA2	2:Q:57:LEU:HD12	1.84	0.58
1:C:391:GLU:OE1	1:C:395:ARG:NH1	2.36	0.58
1:F:205:ILE:HA	1:F:213:VAL:HG22	1.85	0.58
1:H:32:GLY:HA3	1:H:454:ILE:HG23	1.84	0.58
1:I:73:MET:SD	1:J:47:PRO:HD2	2.43	0.58
1:N:81:ALA:HB1	1:N:503:ALA:HA	1.85	0.58
2:T:43:VAL:HG13	2:T:57:LEU:HD22	1.84	0.58

Continued on next page...

For Manuscript Review

Continued from previous page...

Atom-1	Atom-2	Interatomic distance (Å)	Clash overlap (Å)
1:C:417:VAL:HG21	1:C:477:GLY:HA3	1.84	0.58
1:E:239:ALA:HA	1:E:242:LYS:HE2	1.86	0.58
1:G:28:LYS:HE2	1:G:94:VAL:HG22	1.85	0.58
1:G:149:THR:OG1	1:G:156:GLU:HA	2.03	0.58
1:G:452:ARG:HH12	1:G:463:SER:HA	1.68	0.58
1:J:205:ILE:HA	1:J:213:VAL:HG22	1.84	0.58
1:K:13:ARG:HD3	1:K:104:LEU:HD22	1.85	0.58
1:M:199:TYR:CE1	1:M:205:ILE:HD11	2.37	0.58
2:T:8:ASP:HA	2:T:57:LEU:HD11	1.84	0.58
1:B:190:VAL:N	1:B:376:VAL:O	2.27	0.58
1:G:203:TYR:HB2	1:G:263:VAL:CG2	2.33	0.58
1:G:222:LEU:O	1:G:301:ILE:N	2.28	0.58
1:J:185:ASP:OD1	1:J:382:GLY:N	2.34	0.58
1:K:326:ASN:N	1:K:329:THR:O	2.24	0.58
1:M:82:ASN:HB2	1:M:89:THR:HG22	1.84	0.58
1:M:346:VAL:HG22	1:M:372:LEU:HB3	1.84	0.58
1:M:353:ILE:HG23	1:M:362:ARG:NH1	2.17	0.58
2:P:94:ILE:HD11	2:Q:4:ARG:HE	1.68	0.58
1:A:169:VAL:HB	1:A:377:ALA:HB2	1.84	0.58
1:C:124:VAL:HG13	1:C:504:LEU:HG	1.84	0.58
1:C:148:GLY:O	1:C:152:ALA:N	2.35	0.58
1:D:214:GLU:HG3	1:D:324:VAL:HG22	1.83	0.58
1:E:381:VAL:HG12	1:E:389:MET:HE1	1.86	0.58
1:F:262:LEU:HD22	1:F:273:VAL:HG21	1.85	0.58
1:F:265:ASN:OD1	2:T:26:VAL:N	2.28	0.58
1:G:264:VAL:O	1:G:268:ARG:HG2	2.03	0.58
1:G:443:ALA:O	1:G:447:MET:HG2	2.03	0.58
1:H:345:ARG:O	1:H:349:ILE:HG13	2.04	0.58
1:I:359:ASP:OD1	1:I:360:TYR:N	2.35	0.58
1:J:81:ALA:O	1:J:85:ALA:HB3	2.02	0.58
1:L:111:MET:HG3	1:L:116:LEU:HD11	1.85	0.58
1:N:266:THR:HG22	1:N:273:VAL:H	1.69	0.58
1:N:498:LYS:HG3	1:N:501:ARG:NH2	2.18	0.58
2:R:73:VAL:HG22	2:R:86:MET:SD	2.42	0.58
1:A:320:ALA:HA	1:A:335:GLY:HA2	1.86	0.58
1:B:124:VAL:HG21	1:B:508:ALA:HB2	1.86	0.58
1:D:130:GLU:HB2	1:D:422:VAL:HG13	1.86	0.58
1:D:231:ARG:HH21	1:D:234:LEU:HD21	1.68	0.58
1:E:266:THR:O	1:E:272:LYS:NZ	2.26	0.58
1:E:381:VAL:HG13	1:E:392:LYS:HE3	1.86	0.58
1:F:232:GLU:HA	1:F:310:GLU:HG3	1.86	0.58

Continued on next page...

For Manuscript Review

Continued from previous page...

Atom-1	Atom-2	Interatomic distance (Å)	Clash overlap (Å)
1:H:190:VAL:O	1:H:376:VAL:N	2.34	0.58
1:I:202:PRO:HG2	1:J:384:ALA:HA	1.85	0.58
1:N:458:CYS:SG	1:N:480:ALA:HB1	2.43	0.58
2:O:3:ILE:HG13	2:O:78:ILE:HG21	1.85	0.58
2:O:11:ILE:HG13	2:O:85:ILE:HD13	1.85	0.58
2:T:66:ILE:HD11	2:U:3:ILE:HD13	1.85	0.58
1:B:12:ALA:HA	1:B:520:MET:CE	2.33	0.58
1:B:448:GLU:OE1	1:B:470:LYS:NZ	2.37	0.58
1:C:111:MET:HE1	1:C:438:VAL:HB	1.85	0.58
1:D:411:VAL:HG21	1:D:494:LEU:HD22	1.86	0.58
1:F:31:LEU:O	1:F:457:ASN:ND2	2.26	0.58
1:H:141:SER:HB3	1:H:163:ALA:HB1	1.84	0.58
2:P:11:ILE:HG23	2:P:83:VAL:HB	1.86	0.58
1:D:231:ARG:HH12	2:R:31:ALA:HB1	1.68	0.58
1:E:231:ARG:HH21	2:S:31:ALA:HB1	1.69	0.58
1:E:261:THR:O	1:E:265:ASN:ND2	2.35	0.58
1:F:169:VAL:HB	1:F:377:ALA:HB2	1.84	0.58
1:F:479:ASN:ND2	1:F:491:MET:SD	2.77	0.58
1:G:144:ILE:HG23	1:G:403:THR:HB	1.84	0.58
1:K:301:ILE:HG12	1:K:307:MET:HE1	1.84	0.58
1:M:5:ASP:N	1:M:522:THR:O	2.33	0.58
2:P:11:ILE:HG13	2:P:85:ILE:HD13	1.86	0.58
2:R:10:VAL:N	2:R:86:MET:O	2.29	0.58
2:R:15:LYS:HG2	2:R:38:GLY:HA2	1.85	0.58
1:C:251:ALA:O	1:C:278:ALA:N	2.37	0.58
1:F:35:GLY:O	7:F:701:HOH:O	2.16	0.58
1:H:166:MET:HB2	1:H:171:LYS:HA	1.85	0.58
1:H:421:ARG:NH2	1:H:476:TYR:O	2.27	0.58
1:L:81:ALA:HB1	1:L:503:ALA:HA	1.85	0.58
1:L:381:VAL:O	1:L:389:MET:HE1	2.04	0.58
1:E:342:ILE:HA	1:E:372:LEU:HD21	1.86	0.58
1:G:409:GLU:OE2	1:G:501:ARG:NH2	2.36	0.58
1:I:419:LEU:HB3	1:I:447:MET:HB3	1.84	0.58
1:K:455:VAL:HG21	1:K:465:VAL:HG11	1.85	0.58
1:L:495:ASP:OD2	6:L:601:ADP:O2'	2.19	0.58
2:R:65:VAL:HB	2:R:91:ILE:HG23	1.85	0.58
2:T:40:VAL:HG23	2:T:62:GLY:H	1.68	0.58
2:U:65:VAL:HB	2:U:91:ILE:HG23	1.86	0.58
1:F:240:VAL:HG11	1:F:247:LEU:HB2	1.86	0.57
1:G:5:ASP:HB3	1:G:522:THR:OG1	2.04	0.57
1:I:185:ASP:OD1	1:I:382:GLY:N	2.29	0.57

Continued on next page...

For Manuscript Review

Continued from previous page...

Atom-1	Atom-2	Interatomic distance (Å)	Clash overlap (Å)
1:J:82:ASN:HB2	1:J:89:THR:HG22	1.86	0.57
1:M:124:VAL:HG21	1:M:508:ALA:HB2	1.85	0.57
1:A:240:VAL:HG21	1:A:247:LEU:HD12	1.87	0.57
1:A:346:VAL:HA	1:A:349:ILE:HD12	1.85	0.57
1:B:415:GLY:HA2	3:B:601:ATP:H1'	1.85	0.57
1:D:66:PHE:HB3	1:D:520:MET:SD	2.44	0.57
1:D:158:VAL:HG11	1:D:396:VAL:HA	1.86	0.57
1:E:421:ARG:NH2	1:E:469:VAL:O	2.33	0.57
1:F:343:GLN:HA	1:F:346:VAL:HG22	1.84	0.57
1:H:326:ASN:N	1:H:329:THR:O	2.28	0.57
1:J:223:ALA:HA	1:J:301:ILE:HB	1.86	0.57
1:K:166:MET:HB3	1:K:171:LYS:HG2	1.86	0.57
2:P:14:ARG:HB3	2:P:67:PHE:HZ	1.69	0.57
1:A:443:ALA:O	1:A:447:MET:HG2	2.04	0.57
1:C:468:THR:HB	1:C:485:TYR:CE2	2.39	0.57
1:G:196:ASP:HA	1:G:329:THR:HA	1.85	0.57
1:J:177:VAL:HA	1:J:379:ILE:HB	1.86	0.57
1:J:479:ASN:N	1:J:484:GLU:O	2.37	0.57
2:P:10:VAL:HG22	2:P:43:VAL:HG22	1.84	0.57
2:U:10:VAL:HG22	2:U:43:VAL:HG22	1.86	0.57
1:A:325:ILE:HG13	1:A:330:THR:HG23	1.86	0.57
1:A:417:VAL:HG21	1:A:477:GLY:HA3	1.86	0.57
1:B:431:GLY:HA3	1:B:436:GLN:HB3	1.85	0.57
1:C:20:VAL:HG13	1:C:74:VAL:HG21	1.87	0.57
1:E:149:THR:OG1	1:E:156:GLU:HA	2.04	0.57
1:F:261:THR:O	1:F:265:ASN:ND2	2.37	0.57
1:J:89:THR:N	6:J:601:ADP:O3B	2.37	0.57
1:J:290:GLN:OE1	1:J:294:THR:OG1	2.22	0.57
1:J:381:VAL:HG21	1:J:393:LYS:HG2	1.86	0.57
2:R:64:ILE:O	2:R:95:VAL:N	2.37	0.57
2:S:65:VAL:HG12	2:S:94:ILE:HA	1.86	0.57
1:A:420:ILE:HG12	1:A:448:GLU:HG2	1.85	0.57
1:D:203:TYR:HB2	1:D:263:VAL:HB	1.85	0.57
1:F:148:GLY:HA2	1:F:399:ALA:HB1	1.87	0.57
1:G:346:VAL:HB	1:G:369:VAL:HG22	1.85	0.57
1:H:193:MET:HG2	1:H:371:LYS:HB3	1.87	0.57
1:K:68:ASN:O	1:K:72:GLN:HG2	2.05	0.57
1:K:406:ALA:HB2	1:K:496:PRO:HG3	1.87	0.57
1:A:108:ALA:HB1	1:H:109:ALA:HB1	1.87	0.57
1:D:31:LEU:O	1:D:457:ASN:ND2	2.23	0.57
1:E:189:VAL:HA	1:E:377:ALA:HA	1.86	0.57

Continued on next page...

For Manuscript Review

Continued from previous page...

Atom-1	Atom-2	Interatomic distance (Å)	Clash overlap (Å)
1:E:219:PHE:HD2	1:E:240:VAL:HG22	1.69	0.57
1:I:221:LEU:HB3	1:I:249:ILE:HA	1.87	0.57
2:P:67:PHE:HB3	2:P:91:ILE:HD13	1.84	0.57
2:U:8:ASP:HA	2:U:57:LEU:HD11	1.85	0.57
1:B:458:CYS:SG	1:B:480:ALA:HB1	2.44	0.57
1:E:323:VAL:HG22	1:E:332:ILE:HA	1.86	0.57
1:I:7:LYS:HE3	1:I:15:LYS:HG3	1.85	0.57
1:K:199:TYR:CE2	1:K:205:ILE:HD11	2.40	0.57
1:A:47:PRO:HG2	1:G:69:MET:HB3	1.86	0.57
1:B:239:ALA:HA	1:B:242:LYS:HE2	1.86	0.57
1:C:179:ASP:HA	1:C:381:VAL:HB	1.87	0.57
1:C:448:GLU:OE1	1:C:470:LYS:NZ	2.34	0.57
1:E:71:ALA:HA	1:E:74:VAL:HG12	1.86	0.57
1:H:411:VAL:HA	1:H:496:PRO:HA	1.87	0.57
1:L:343:GLN:HA	1:L:346:VAL:HB	1.85	0.57
1:L:350:ARG:NH1	1:L:369:VAL:HB	2.19	0.57
1:M:421:ARG:NH1	1:M:469:VAL:O	2.37	0.57
2:R:11:ILE:HG22	2:R:41:LEU:HB2	1.87	0.57
1:A:130:GLU:HB2	1:A:422:VAL:HG13	1.86	0.57
1:B:180:GLY:N	1:B:381:VAL:O	2.28	0.57
1:B:321:LYS:NZ	1:B:336:VAL:HG11	2.20	0.57
1:D:264:VAL:O	1:D:268:ARG:HG2	2.05	0.57
1:E:278:ALA:HB3	1:E:285:ARG:HE	1.70	0.57
1:E:452:ARG:HH12	1:E:463:SER:HA	1.70	0.57
1:H:240:VAL:HG21	1:H:247:LEU:HD13	1.87	0.57
1:H:519:CYS:HB3	1:I:38:VAL:HG22	1.86	0.57
1:N:455:VAL:HG22	1:N:478:TYR:CE2	2.40	0.57
1:A:339:GLU:HA	1:A:342:ILE:HD12	1.86	0.57
1:B:199:TYR:CD2	1:B:213:VAL:HG23	2.40	0.57
1:B:339:GLU:HA	1:B:342:ILE:HD12	1.86	0.57
1:D:124:VAL:HG21	1:D:508:ALA:HB2	1.87	0.57
1:E:231:ARG:HD3	1:E:234:LEU:HD11	1.87	0.57
1:F:420:ILE:HG12	1:F:448:GLU:HG2	1.86	0.57
1:H:169:VAL:HG21	1:H:377:ALA:HB2	1.87	0.57
1:H:199:TYR:CE2	1:H:205:ILE:HD11	2.39	0.57
1:I:33:PRO:HD3	6:I:601:ADP:C4	2.39	0.57
1:I:39:VAL:HA	1:I:49:ILE:HA	1.87	0.57
1:J:18:ARG:NE	1:J:67:GLU:OE2	2.34	0.57
1:J:274:ALA:HB1	1:J:325:ILE:HD13	1.87	0.57
1:A:231:ARG:NH2	2:O:31:ALA:O	2.37	0.56
1:B:150:ILE:HG23	3:B:601:ATP:C8	2.40	0.56

Continued on next page...

For Manuscript Review

Continued from previous page...

Atom-1	Atom-2	Interatomic distance (Å)	Clash overlap (Å)
1:C:214:GLU:OE2	1:C:322:ARG:NH1	2.38	0.56
1:C:231:ARG:HD3	1:C:234:LEU:HD11	1.87	0.56
1:D:200:LEU:HD12	1:D:275:ALA:HB1	1.86	0.56
1:D:350:ARG:HA	1:D:353:ILE:HD12	1.87	0.56
1:G:150:ILE:HG13	1:G:493:ILE:HA	1.87	0.56
1:H:501:ARG:NH1	1:H:505:GLN:OE1	2.38	0.56
1:I:479:ASN:O	1:I:483:GLU:N	2.38	0.56
1:K:235:PRO:HG3	1:K:310:GLU:HA	1.87	0.56
1:K:352:GLN:OE1	1:K:368:ARG:NH2	2.38	0.56
1:M:27:VAL:HG12	1:M:90:THR:HG23	1.87	0.56
1:M:519:CYS:HB3	1:N:38:VAL:HG22	1.85	0.56
1:N:501:ARG:NH1	1:N:505:GLN:OE1	2.38	0.56
2:O:11:ILE:HG23	2:O:83:VAL:HB	1.85	0.56
2:R:57:LEU:O	2:R:60:LYS:NZ	2.27	0.56
2:S:66:ILE:HD11	2:T:3:ILE:HD13	1.87	0.56
2:U:12:VAL:HA	2:U:40:VAL:HA	1.87	0.56
1:B:487:ASN:O	1:B:491:MET:HG2	2.04	0.56
1:C:209:GLU:HG2	1:C:210:THR:HG23	1.87	0.56
1:C:230:ILE:H	1:C:230:ILE:HD12	1.69	0.56
1:C:349:ILE:HG22	1:C:365:LEU:HB3	1.86	0.56
1:F:220:ILE:HG13	1:F:248:LEU:HD23	1.87	0.56
1:J:39:VAL:HG22	1:J:49:ILE:HG12	1.87	0.56
1:J:353:ILE:HD11	1:J:369:VAL:HG11	1.87	0.56
1:K:324:VAL:HB	1:K:331:THR:HG23	1.87	0.56
1:L:131:LEU:HD21	1:L:500:THR:HB	1.85	0.56
1:L:215:LEU:HB3	1:L:246:PRO:HB2	1.86	0.56
2:O:76:GLU:HB3	2:O:78:ILE:HG23	1.87	0.56
2:Q:47:ARG:HH22	2:Q:88:GLU:HB3	1.70	0.56
1:A:265:ASN:OD1	2:O:26:VAL:N	2.28	0.56
1:B:179:ASP:OD1	1:B:393:LYS:HD2	2.06	0.56
1:B:206:ASN:HD21	1:B:214:GLU:HB3	1.70	0.56
1:B:261:THR:O	1:B:265:ASN:ND2	2.37	0.56
1:B:343:GLN:HA	1:B:346:VAL:HG22	1.87	0.56
1:D:244:GLY:O	1:D:272:LYS:NZ	2.36	0.56
1:D:346:VAL:HA	1:D:349:ILE:HB	1.87	0.56
1:D:353:ILE:HG23	1:D:362:ARG:HB2	1.87	0.56
1:E:166:MET:HA	1:E:169:VAL:HG12	1.87	0.56
1:F:223:ALA:HB1	1:F:225:LYS:HG2	1.87	0.56
1:F:287:ALA:HA	1:F:345:ARG:HH21	1.70	0.56
1:G:124:VAL:HG21	1:G:508:ALA:HB2	1.86	0.56
1:H:27:VAL:HG12	1:H:90:THR:HG23	1.86	0.56

Continued on next page...

For Manuscript Review

Continued from previous page...

Atom-1	Atom-2	Interatomic distance (Å)	Clash overlap (Å)
1:H:73:MET:SD	1:I:47:PRO:HD2	2.46	0.56
1:H:137:PRO:HA	1:H:410:GLY:HA2	1.87	0.56
1:I:381:VAL:HG23	1:I:389:MET:SD	2.45	0.56
1:J:12:ALA:HA	1:J:520:MET:HE2	1.86	0.56
1:J:218:PRO:HB3	1:J:246:PRO:HG2	1.88	0.56
1:K:81:ALA:HB1	1:K:503:ALA:HA	1.86	0.56
1:K:194:GLN:HG3	1:K:331:THR:HB	1.86	0.56
1:K:501:ARG:NH1	1:K:505:GLN:OE1	2.38	0.56
1:N:65:LYS:O	1:N:69:MET:HG3	2.04	0.56
1:N:131:LEU:HG	1:N:497:THR:HG23	1.87	0.56
1:N:223:ALA:HA	1:N:301:ILE:HB	1.87	0.56
2:T:58:ASP:OD2	2:U:7:HIS:NE2	2.39	0.56
1:G:279:PRO:HG2	1:G:288:MET:HB3	1.86	0.56
1:G:429:LEU:O	1:G:430:ARG:NH1	2.38	0.56
1:H:233:MET:HG3	1:H:237:LEU:HG	1.86	0.56
1:J:325:ILE:HA	1:J:330:THR:HA	1.88	0.56
1:K:487:ASN:O	1:K:491:MET:HG2	2.04	0.56
1:L:166:MET:HB2	1:L:171:LYS:HA	1.86	0.56
1:M:33:PRO:HD3	6:M:601:ADP:C4	2.41	0.56
1:M:81:ALA:O	1:M:85:ALA:HB2	2.06	0.56
1:M:81:ALA:O	1:M:85:ALA:HB3	2.06	0.56
1:M:349:ILE:HD13	1:M:368:ARG:HB3	1.86	0.56
1:N:41:ASP:HA	1:N:47:PRO:HB3	1.87	0.56
2:S:10:VAL:HG13	2:S:40:VAL:HG13	1.87	0.56
1:A:177:VAL:HG23	1:A:400:LEU:HD22	1.88	0.56
1:B:197:ARG:O	1:B:330:THR:OG1	2.16	0.56
1:C:150:ILE:HG13	1:C:493:ILE:HA	1.87	0.56
1:C:166:MET:HA	1:C:169:VAL:HG12	1.86	0.56
1:D:287:ALA:HA	1:D:345:ARG:HH21	1.70	0.56
1:D:431:GLY:N	1:D:437:ASN:OD1	2.39	0.56
1:E:204:PHE:HD1	1:E:266:THR:HG21	1.70	0.56
1:G:27:VAL:HG12	1:G:90:THR:HG23	1.88	0.56
1:G:190:VAL:N	1:G:376:VAL:O	2.29	0.56
1:H:195:PHE:HB3	1:H:371:LYS:HE3	1.88	0.56
1:J:193:MET:HG2	1:J:295:LEU:HD13	1.87	0.56
2:U:46:GLY:HA2	2:U:57:LEU:HD12	1.86	0.56
1:A:65:LYS:O	1:A:69:MET:HG3	2.05	0.56
1:A:141:SER:HA	1:A:144:ILE:HD12	1.86	0.56
1:A:468:THR:HB	1:A:485:TYR:CE2	2.41	0.56
1:F:20:VAL:HG22	1:F:74:VAL:HB	1.85	0.56
1:G:199:TYR:CD2	1:G:213:VAL:HG23	2.41	0.56

Continued on next page...

For Manuscript Review

Continued from previous page...

Atom-1	Atom-2	Interatomic distance (Å)	Clash overlap (Å)
1:H:498:LYS:HG3	1:H:501:ARG:NH2	2.20	0.56
1:I:124:VAL:HG21	1:I:508:ALA:HB2	1.88	0.56
1:K:295:LEU:HA	1:K:342:ILE:HG12	1.88	0.56
1:M:205:ILE:HA	1:M:213:VAL:HG22	1.88	0.56
1:N:161:LEU:HG	1:N:187:LEU:HD23	1.87	0.56
2:O:66:ILE:HG21	2:P:76:GLU:HG2	1.88	0.56
2:P:5:PRO:HB3	2:P:85:ILE:HD11	1.87	0.56
1:A:262:LEU:HD13	1:A:273:VAL:HG11	1.87	0.56
1:C:178:GLU:HA	1:C:393:LYS:HE2	1.87	0.56
1:F:239:ALA:HA	1:F:242:LYS:HE2	1.87	0.56
1:G:151:SER:OG	1:G:399:ALA:HA	2.06	0.56
1:I:5:ASP:N	1:I:522:THR:O	2.38	0.56
1:I:165:ALA:HB2	1:I:187:LEU:HD22	1.88	0.56
1:L:27:VAL:HG12	1:L:90:THR:HG23	1.88	0.56
1:L:274:ALA:HB1	1:L:325:ILE:HD13	1.86	0.56
2:O:26:VAL:HG12	2:O:28:THR:HG23	1.87	0.56
2:P:77:LYS:HG3	2:P:80:ASN:HA	1.87	0.56
2:Q:95:VAL:HA	2:R:3:ILE:HG12	1.87	0.56
2:T:14:ARG:HA	2:T:38:GLY:HA2	1.87	0.56
1:A:41:ASP:HB2	1:G:522:THR:HG22	1.87	0.56
1:B:247:LEU:HB3	1:B:273:VAL:HG22	1.87	0.56
1:G:158:VAL:HG11	1:G:396:VAL:HA	1.87	0.56
1:H:461:GLU:HG3	1:H:464:VAL:H	1.71	0.56
1:I:262:LEU:HD22	1:I:273:VAL:HG21	1.88	0.56
1:I:274:ALA:HB1	1:I:325:ILE:HD13	1.88	0.56
1:L:222:LEU:HD23	1:L:250:ILE:HB	1.87	0.56
1:M:233:MET:HG3	1:M:237:LEU:HG	1.87	0.56
1:M:235:PRO:HG3	1:M:310:GLU:HA	1.87	0.56
1:N:301:ILE:HG12	1:N:307:MET:HE1	1.87	0.56
1:N:365:LEU:HD23	1:N:368:ARG:HE	1.71	0.56
2:O:5:PRO:HB3	2:O:85:ILE:HD11	1.88	0.56
2:P:20:LYS:NZ	2:P:23:GLY:O	2.38	0.56
1:C:232:GLU:HA	1:C:310:GLU:HG3	1.87	0.56
1:D:12:ALA:HA	1:D:520:MET:CE	2.36	0.56
1:E:69:MET:HB2	1:F:47:PRO:HG2	1.88	0.56
1:G:152:ALA:HB2	1:G:399:ALA:HB2	1.88	0.56
1:H:249:ILE:O	1:H:276:VAL:N	2.23	0.56
1:I:18:ARG:NE	1:I:67:GLU:OE2	2.38	0.56
1:I:81:ALA:O	1:I:85:ALA:HB2	2.06	0.56
1:K:479:ASN:HB2	1:K:491:MET:CE	2.35	0.56
1:L:218:PRO:HB3	1:L:246:PRO:HG2	1.88	0.56

Continued on next page...

For Manuscript Review

Continued from previous page...

Atom-1	Atom-2	Interatomic distance (Å)	Clash overlap (Å)
1:L:345:ARG:NH2	1:L:368:ARG:HH12	2.03	0.56
2:O:43:VAL:HG13	2:O:57:LEU:HD22	1.87	0.56
1:A:205:ILE:HA	1:A:213:VAL:HG22	1.88	0.56
1:D:219:PHE:CD2	1:D:240:VAL:HG22	2.40	0.56
1:I:345:ARG:NH2	1:I:368:ARG:HH12	2.04	0.56
1:K:333:ILE:HG12	1:K:376:VAL:HG11	1.88	0.56
1:L:124:VAL:HG21	1:L:508:ALA:HB2	1.88	0.56
1:M:177:VAL:HA	1:M:379:ILE:HB	1.87	0.56
2:R:11:ILE:N	2:R:42:ALA:O	2.33	0.56
1:B:149:THR:OG1	1:B:156:GLU:HA	2.06	0.55
1:B:200:LEU:HD12	1:B:275:ALA:HB1	1.87	0.55
1:B:322:ARG:O	1:B:333:ILE:N	2.35	0.55
1:B:468:THR:HB	1:B:485:TYR:CE2	2.41	0.55
1:C:186:GLU:HB3	1:C:380:LYS:HB2	1.88	0.55
1:C:409:GLU:OE2	1:C:501:ARG:NH2	2.38	0.55
1:D:5:ASP:N	1:D:522:THR:O	2.27	0.55
1:E:143:ALA:O	1:E:147:VAL:HG23	2.06	0.55
1:E:443:ALA:O	1:E:447:MET:HG2	2.05	0.55
1:F:130:GLU:HB2	1:F:422:VAL:HG13	1.87	0.55
1:G:214:GLU:HG3	1:G:324:VAL:HG22	1.88	0.55
1:H:384:ALA:HA	1:N:202:PRO:HG2	1.87	0.55
1:H:433:ASN:H	1:H:436:GLN:HB2	1.71	0.55
1:I:132:LYS:NZ	1:I:409:GLU:OE2	2.37	0.55
1:J:15:LYS:HB3	1:J:66:PHE:HB2	1.88	0.55
1:J:221:LEU:HD23	1:J:249:ILE:HG23	1.87	0.55
1:K:421:ARG:NH1	1:K:469:VAL:O	2.39	0.55
1:K:458:CYS:SG	1:K:480:ALA:HB1	2.47	0.55
1:K:477:GLY:N	1:K:486:GLY:O	2.39	0.55
1:M:498:LYS:HG3	1:M:501:ARG:NH2	2.21	0.55
2:T:64:ILE:O	2:T:95:VAL:N	2.39	0.55
2:U:38:GLY:HA3	2:U:67:PHE:HE1	1.71	0.55
1:C:308:GLU:H	1:C:311:LYS:HD3	1.70	0.55
1:D:431:GLY:HA3	1:D:436:GLN:HB3	1.88	0.55
1:G:213:VAL:HG11	1:G:274:ALA:HB2	1.88	0.55
1:H:219:PHE:CE2	1:H:314:LEU:HD22	2.40	0.55
1:H:351:GLN:HA	1:H:354:GLU:CD	2.25	0.55
1:I:155:ASP:OD2	1:I:395:ARG:HD2	2.06	0.55
1:I:178:GLU:N	1:I:379:ILE:O	2.25	0.55
1:L:233:MET:HG3	1:L:237:LEU:HG	1.86	0.55
1:L:339:GLU:HB3	1:L:343:GLN:HE22	1.71	0.55
1:L:433:ASN:H	1:L:436:GLN:HB2	1.70	0.55

Continued on next page...

For Manuscript Review

Continued from previous page...

Atom-1	Atom-2	Interatomic distance (Å)	Clash overlap (Å)
1:M:263:VAL:O	1:M:266:THR:OG1	2.22	0.55
1:B:365:LEU:HD13	1:B:368:ARG:HD3	1.87	0.55
1:B:452:ARG:HH12	1:B:463:SER:HA	1.71	0.55
1:C:302:SER:OG	1:C:304:GLU:OE1	2.23	0.55
1:D:150:ILE:HG23	3:D:601:ATP:C8	2.41	0.55
1:F:488:MET:HA	1:F:491:MET:HE2	1.88	0.55
1:G:349:ILE:HG23	1:G:365:LEU:HD12	1.87	0.55
1:G:468:THR:HB	1:G:485:TYR:CE2	2.40	0.55
1:H:47:PRO:HD2	1:N:73:MET:SD	2.47	0.55
1:L:202:PRO:HG2	1:M:384:ALA:HA	1.88	0.55
1:M:461:GLU:HG3	1:M:464:VAL:H	1.71	0.55
1:N:346:VAL:HG22	1:N:372:LEU:HB3	1.87	0.55
2:P:40:VAL:HG23	2:P:62:GLY:H	1.71	0.55
2:P:95:VAL:HA	2:Q:3:ILE:HG22	1.87	0.55
1:B:158:VAL:HG11	1:B:396:VAL:HA	1.87	0.55
1:B:193:MET:HB2	1:B:332:ILE:HB	1.89	0.55
1:D:204:PHE:HD1	1:D:266:THR:HG21	1.71	0.55
1:E:16:MET:HE3	1:E:69:MET:SD	2.46	0.55
1:H:177:VAL:HA	1:H:379:ILE:HB	1.89	0.55
1:I:233:MET:HG3	1:I:237:LEU:HG	1.88	0.55
1:K:349:ILE:HD13	1:K:368:ARG:HB3	1.89	0.55
1:N:29:VAL:O	1:N:36:ARG:N	2.34	0.55
1:A:204:PHE:HD1	1:A:266:THR:HG21	1.72	0.55
1:A:343:GLN:HA	1:A:346:VAL:HG22	1.88	0.55
1:C:240:VAL:HG11	1:C:247:LEU:HB2	1.87	0.55
1:H:39:VAL:HG13	1:H:49:ILE:HG12	1.89	0.55
1:H:168:LYS:HG2	1:H:189:VAL:HG13	1.87	0.55
1:I:295:LEU:HA	1:I:342:ILE:HG12	1.89	0.55
1:I:381:VAL:HG11	1:I:393:LYS:HA	1.88	0.55
1:L:432:GLN:OE1	1:L:436:GLN:NE2	2.31	0.55
1:M:218:PRO:HB3	1:M:246:PRO:HG2	1.89	0.55
1:M:368:ARG:O	1:M:372:LEU:HD23	2.07	0.55
1:N:14:VAL:HB	1:N:18:ARG:NH1	2.21	0.55
1:B:184:GLN:NE2	1:B:185:ASP:OD1	2.40	0.55
1:D:20:VAL:HG22	1:D:74:VAL:HB	1.88	0.55
1:E:431:GLY:HA3	1:E:436:GLN:HB3	1.89	0.55
1:E:468:THR:HB	1:E:485:TYR:CE2	2.42	0.55
1:F:166:MET:HA	1:F:169:VAL:HG12	1.89	0.55
1:G:39:VAL:HG22	1:G:49:ILE:HG12	1.88	0.55
1:H:155:ASP:OD2	1:H:395:ARG:HD2	2.06	0.55
1:I:302:SER:HB2	1:I:304:GLU:HG2	1.86	0.55

Continued on next page...

For Manuscript Review

Continued from previous page...

Atom-1	Atom-2	Interatomic distance (Å)	Clash overlap (Å)
1:K:221:LEU:HB3	1:K:249:ILE:HA	1.87	0.55
1:L:427:ALA:HA	1:L:444:LEU:HD13	1.88	0.55
1:M:89:THR:N	6:M:601:ADP:O3B	2.39	0.55
1:N:89:THR:N	6:N:601:ADP:O3B	2.38	0.55
2:P:43:VAL:HG13	2:P:57:LEU:HD22	1.88	0.55
1:A:20:VAL:HG12	1:A:97:GLN:OE1	2.07	0.55
1:B:71:ALA:HA	1:B:74:VAL:HG12	1.89	0.55
1:B:230:ILE:O	1:B:234:LEU:N	2.40	0.55
1:E:150:ILE:HG23	3:E:601:ATP:C8	2.42	0.55
1:H:230:ILE:O	1:H:234:LEU:N	2.39	0.55
1:J:20:VAL:HG22	1:J:74:VAL:HG21	1.88	0.55
1:L:13:ARG:HD3	1:L:514:MET:HE3	1.86	0.55
1:L:472:GLY:HA3	1:L:476:TYR:CD2	2.42	0.55
1:N:137:PRO:HA	1:N:410:GLY:HA2	1.89	0.55
1:B:356:ALA:O	1:B:362:ARG:NH2	2.39	0.55
1:C:240:VAL:HG21	1:C:247:LEU:HD13	1.89	0.55
1:C:414:GLY:HA3	1:C:493:ILE:HG22	1.89	0.55
1:E:65:LYS:O	1:E:69:MET:HG3	2.06	0.55
1:F:149:THR:OG1	1:F:156:GLU:HA	2.07	0.55
1:G:150:ILE:HG23	3:G:601:ATP:C8	2.42	0.55
1:I:346:VAL:HG22	1:I:372:LEU:HB3	1.88	0.55
1:J:279:PRO:O	1:J:285:ARG:HA	2.06	0.55
1:L:203:TYR:HB2	1:L:263:VAL:HG13	1.89	0.55
1:L:263:VAL:HG12	1:L:267:MET:HE1	1.89	0.55
1:M:117:LYS:HB2	1:M:515:ILE:HG21	1.89	0.55
1:A:452:ARG:HH12	1:A:463:SER:HA	1.71	0.55
1:B:102:GLU:HB2	1:B:442:VAL:HG13	1.88	0.55
1:B:221:LEU:HD23	1:B:249:ILE:HG12	1.88	0.55
1:D:349:ILE:HG23	1:D:365:LEU:HD12	1.89	0.55
1:E:28:LYS:HE2	1:E:94:VAL:HG22	1.89	0.55
1:E:124:VAL:HG13	1:E:504:LEU:HG	1.88	0.55
1:E:432:GLN:HB2	1:E:436:GLN:NE2	2.22	0.55
1:H:89:THR:N	6:H:601:ADP:O3B	2.39	0.55
1:H:291:ASP:HB3	1:H:372:LEU:HD21	1.87	0.55
1:H:301:ILE:HG21	1:H:309:LEU:HD23	1.89	0.55
1:I:322:ARG:HB2	1:I:333:ILE:HB	1.87	0.55
1:J:68:ASN:O	1:J:72:GLN:HG2	2.07	0.55
1:L:290:GLN:OE1	1:L:294:THR:OG1	2.24	0.55
1:B:166:MET:HA	1:B:169:VAL:HG12	1.89	0.55
1:E:20:VAL:HG13	1:E:74:VAL:HG21	1.89	0.55
1:E:20:VAL:HG22	1:E:74:VAL:HB	1.89	0.55

Continued on next page...

For Manuscript Review

Continued from previous page...

Atom-1	Atom-2	Interatomic distance (Å)	Clash overlap (Å)
1:F:199:TYR:CD2	1:F:213:VAL:HG23	2.42	0.55
1:I:68:ASN:O	1:I:72:GLN:HG2	2.07	0.55
1:J:85:ALA:HB2	1:J:502:SER:HB2	1.89	0.55
1:K:124:VAL:HG13	1:K:504:LEU:HG	1.88	0.55
1:K:197:ARG:HE	1:K:279:PRO:HA	1.72	0.55
1:K:414:GLY:HA3	1:K:493:ILE:HG22	1.89	0.55
1:L:461:GLU:HG3	1:L:464:VAL:H	1.71	0.55
2:Q:47:ARG:N	2:Q:55:LYS:O	2.40	0.55
2:S:65:VAL:HG21	2:S:91:ILE:HD12	1.88	0.55
1:A:220:ILE:HG23	1:A:250:ILE:HD12	1.88	0.54
1:B:219:PHE:CZ	1:B:245:LYS:HE2	2.42	0.54
1:C:193:MET:HB2	1:C:332:ILE:HB	1.89	0.54
1:E:221:LEU:HB2	1:E:317:LEU:HD22	1.89	0.54
1:H:197:ARG:HE	1:H:279:PRO:HA	1.72	0.54
1:J:199:TYR:CE1	1:J:205:ILE:HD11	2.42	0.54
1:J:411:VAL:HA	1:J:496:PRO:HA	1.89	0.54
1:L:180:GLY:H	1:L:389:MET:HE2	1.72	0.54
1:L:199:TYR:CE2	1:L:205:ILE:HD11	2.42	0.54
1:M:353:ILE:HG23	1:M:362:ARG:NH2	2.22	0.54
1:N:433:ASN:H	1:N:436:GLN:HB2	1.72	0.54
2:T:11:ILE:HG22	2:T:41:LEU:HB2	1.88	0.54
1:A:220:ILE:HG13	1:A:248:LEU:HD23	1.88	0.54
1:A:414:GLY:HA3	1:A:493:ILE:HG22	1.89	0.54
1:B:323:VAL:HG22	1:B:332:ILE:HA	1.88	0.54
1:B:356:ALA:HB1	1:B:361:ASP:HB2	1.90	0.54
1:D:292:ILE:O	1:D:296:THR:OG1	2.17	0.54
1:D:323:VAL:HG22	1:D:332:ILE:HA	1.89	0.54
1:F:200:LEU:HD12	1:F:275:ALA:HB1	1.89	0.54
1:I:13:ARG:HG3	1:I:104:LEU:HD22	1.89	0.54
1:K:185:ASP:OD2	1:K:392:LYS:HE3	2.07	0.54
2:T:15:LYS:HZ1	2:T:64:ILE:HG12	1.72	0.54
1:B:66:PHE:HB3	1:B:520:MET:SD	2.48	0.54
1:B:135:SER:HB3	1:B:497:THR:HG21	1.89	0.54
1:C:510:VAL:HG23	1:D:385:THR:HG21	1.90	0.54
1:E:200:LEU:HD12	1:E:275:ALA:HB1	1.89	0.54
1:E:250:ILE:HG23	1:E:278:ALA:HA	1.89	0.54
1:F:69:MET:HE1	1:F:520:MET:HB3	1.89	0.54
1:F:144:ILE:HG23	1:F:403:THR:HG21	1.89	0.54
1:G:220:ILE:HG13	1:G:248:LEU:HD23	1.89	0.54
1:H:352:GLN:HA	1:H:355:GLU:HG3	1.89	0.54
1:J:81:ALA:HB1	1:J:503:ALA:HA	1.90	0.54

Continued on next page...

For Manuscript Review

Continued from previous page...

Atom-1	Atom-2	Interatomic distance (Å)	Clash overlap (Å)
1:K:27:VAL:HG12	1:K:90:THR:HG23	1.89	0.54
1:K:284:ARG:NE	1:K:364:LYS:HB3	2.23	0.54
1:N:345:ARG:NH2	1:N:368:ARG:HH12	2.05	0.54
1:A:510:VAL:HG23	1:B:385:THR:HG21	1.89	0.54
1:D:205:ILE:HA	1:D:213:VAL:HG22	1.90	0.54
1:D:278:ALA:HB3	1:D:285:ARG:HH11	1.72	0.54
1:D:417:VAL:HG21	1:D:477:GLY:HA3	1.89	0.54
1:E:177:VAL:HG23	1:E:400:LEU:HD22	1.89	0.54
1:H:419:LEU:HD22	1:H:447:MET:HG3	1.89	0.54
1:J:235:PRO:HG3	1:J:310:GLU:HA	1.89	0.54
1:K:31:LEU:HD13	1:K:90:THR:HB	1.88	0.54
1:K:249:ILE:O	1:K:276:VAL:N	2.34	0.54
1:K:346:VAL:HG22	1:K:372:LEU:HB3	1.89	0.54
2:P:12:VAL:HG22	2:P:84:LEU:HB2	1.90	0.54
1:A:421:ARG:HH12	1:A:470:LYS:HA	1.72	0.54
1:B:115:ASP:OD1	1:B:118:ARG:NH1	2.37	0.54
1:C:176:THR:O	1:C:379:ILE:N	2.28	0.54
1:C:343:GLN:HA	1:C:346:VAL:HG22	1.89	0.54
1:D:397:GLU:O	1:D:401:HIS:ND1	2.41	0.54
1:E:152:ALA:O	1:E:395:ARG:NH1	2.41	0.54
1:E:200:LEU:HD21	1:E:277:LYS:HG3	1.89	0.54
1:F:204:PHE:HD1	1:F:266:THR:HG21	1.71	0.54
1:F:230:ILE:H	1:F:230:ILE:HD12	1.70	0.54
1:H:455:VAL:HG21	1:H:465:VAL:HG11	1.89	0.54
1:J:155:ASP:OD2	1:J:395:ARG:HD2	2.07	0.54
1:J:162:ILE:HD11	1:J:396:VAL:HG13	1.89	0.54
1:J:356:ALA:HB2	1:J:365:LEU:HD12	1.88	0.54
1:K:81:ALA:O	1:K:85:ALA:HB2	2.08	0.54
2:R:11:ILE:HB	2:R:42:ALA:HB3	1.90	0.54
2:U:68:ASN:N	2:U:90:ASP:O	2.33	0.54
1:A:154:SER:N	7:A:2019:HOH:O	2.40	0.54
1:A:458:CYS:SG	1:A:480:ALA:HB1	2.47	0.54
1:C:452:ARG:HH21	1:C:470:LYS:HZ1	1.54	0.54
1:D:122:LYS:HD3	1:D:440:ILE:HD11	1.88	0.54
1:D:353:ILE:HD13	1:D:366:GLN:HG3	1.90	0.54
1:E:7:LYS:HE2	1:E:11:ASP:HB3	1.90	0.54
1:F:152:ALA:HB2	1:F:399:ALA:HB2	1.90	0.54
1:G:51:LYS:NZ	3:G:601:ATP:O1A	2.41	0.54
1:G:200:LEU:N	1:G:275:ALA:O	2.40	0.54
1:J:16:MET:O	1:J:20:VAL:HG23	2.07	0.54
1:K:365:LEU:HA	1:K:368:ARG:HG3	1.90	0.54

Continued on next page...

For Manuscript Review

Continued from previous page...

Atom-1	Atom-2	Interatomic distance (Å)	Clash overlap (Å)
1:L:240:VAL:HG21	1:L:247:LEU:HD13	1.90	0.54
1:N:199:TYR:CE2	1:N:205:ILE:HD11	2.41	0.54
1:B:114:MET:HE1	1:C:34:LYS:HG2	1.88	0.54
1:C:12:ALA:HB1	1:C:16:MET:HE3	1.89	0.54
1:I:414:GLY:HA3	1:I:493:ILE:HG22	1.90	0.54
1:K:89:THR:N	6:K:601:ADP:O3B	2.38	0.54
1:M:233:MET:HG2	1:M:262:LEU:HD21	1.89	0.54
1:B:150:ILE:HG13	1:B:493:ILE:HA	1.90	0.54
1:J:222:LEU:HD23	1:J:250:ILE:HB	1.89	0.54
1:L:177:VAL:HA	1:L:379:ILE:HB	1.89	0.54
1:M:81:ALA:HB1	1:M:503:ALA:HA	1.90	0.54
1:N:40:LEU:N	1:N:48:THR:O	2.37	0.54
2:S:43:VAL:HG13	2:S:57:LEU:HD22	1.89	0.54
2:U:13:LYS:HB2	2:U:41:LEU:HD11	1.89	0.54
1:B:421:ARG:O	1:B:425:LYS:HG3	2.08	0.54
1:B:452:ARG:HH21	1:B:470:LYS:HZ1	1.56	0.54
1:B:479:ASN:N	1:B:484:GLU:O	2.40	0.54
1:C:204:PHE:HD1	1:C:266:THR:HG21	1.73	0.54
1:E:140:ASP:OD1	1:E:140:ASP:N	2.41	0.54
1:E:175:ILE:HB	1:E:404:ARG:HH12	1.72	0.54
1:H:295:LEU:HD13	1:H:332:ILE:HD11	1.88	0.54
1:I:479:ASN:N	1:I:484:GLU:O	2.37	0.54
1:J:498:LYS:HG3	1:J:501:ARG:NH2	2.23	0.54
1:L:250:ILE:HG23	1:L:278:ALA:HA	1.90	0.54
1:N:85:ALA:HB2	1:N:502:SER:HB2	1.89	0.54
1:N:166:MET:HB3	1:N:175:ILE:HD11	1.90	0.54
1:N:356:ALA:HB2	1:N:365:LEU:HD12	1.90	0.54
2:O:8:ASP:OD2	2:O:87:SER:OG	2.25	0.54
1:A:144:ILE:HG23	1:A:403:THR:CG2	2.36	0.54
1:A:200:LEU:HD12	1:A:275:ALA:HB1	1.90	0.54
1:C:190:VAL:N	1:C:376:VAL:O	2.27	0.54
1:C:364:LYS:O	1:C:368:ARG:HG3	2.07	0.54
1:E:214:GLU:HG3	1:E:324:VAL:HG22	1.89	0.54
1:G:64:ASP:O	1:G:68:ASN:N	2.35	0.54
1:G:420:ILE:HG12	1:G:448:GLU:HG2	1.89	0.54
1:H:39:VAL:HA	1:H:49:ILE:HA	1.90	0.54
1:H:324:VAL:HB	1:H:331:THR:HG22	1.90	0.54
1:H:349:ILE:O	1:H:353:ILE:HG13	2.07	0.54
1:I:177:VAL:HA	1:I:379:ILE:HB	1.90	0.54
1:I:324:VAL:N	1:I:331:THR:O	2.35	0.54
1:J:197:ARG:NH2	1:J:280:GLY:O	2.41	0.54

Continued on next page...

For Manuscript Review

Continued from previous page...

Atom-1	Atom-2	Interatomic distance (Å)	Clash overlap (Å)
1:J:302:SER:HB2	1:J:304:GLU:HG2	1.90	0.54
1:K:10:ASN:HA	1:K:13:ARG:HB2	1.89	0.54
1:N:195:PHE:CZ	1:N:250:ILE:HD13	2.43	0.54
1:N:218:PRO:HG3	1:N:323:VAL:HG22	1.90	0.54
1:B:196:ASP:HA	1:B:329:THR:HA	1.88	0.53
1:D:346:VAL:HA	1:D:349:ILE:HD12	1.90	0.53
1:F:197:ARG:HD2	1:F:277:LYS:HB2	1.90	0.53
1:G:155:ASP:OD2	1:G:395:ARG:NH1	2.41	0.53
1:I:62:LEU:HB2	1:I:68:ASN:HB2	1.90	0.53
1:I:475:ASN:HB2	1:I:487:ASN:ND2	2.23	0.53
1:J:262:LEU:HD22	1:J:273:VAL:HG21	1.90	0.53
1:N:122:LYS:HD3	1:N:440:ILE:HD11	1.90	0.53
1:A:124:VAL:HG21	1:A:508:ALA:HB2	1.89	0.53
1:D:149:THR:OG1	1:D:156:GLU:HA	2.08	0.53
1:D:221:LEU:HD21	1:D:309:LEU:HD11	1.90	0.53
1:D:222:LEU:O	1:D:301:ILE:N	2.31	0.53
1:D:468:THR:HB	1:D:485:TYR:CE2	2.43	0.53
1:E:130:GLU:HB2	1:E:422:VAL:HG13	1.89	0.53
1:G:411:VAL:HG21	1:G:494:LEU:HD22	1.88	0.53
1:H:13:ARG:HD3	1:H:514:MET:HE3	1.88	0.53
1:J:163:ALA:HA	1:J:166:MET:HE2	1.90	0.53
1:K:461:GLU:HG3	1:K:464:VAL:H	1.71	0.53
1:M:284:ARG:CZ	1:M:364:LYS:HD2	2.38	0.53
1:N:230:ILE:O	1:N:234:LEU:N	2.41	0.53
2:Q:57:LEU:HD23	2:Q:88:GLU:HB2	1.90	0.53
1:A:230:ILE:O	1:A:234:LEU:N	2.42	0.53
1:A:452:ARG:HH21	1:A:470:LYS:HZ1	1.56	0.53
1:C:397:GLU:O	1:C:401:HIS:ND1	2.42	0.53
1:D:199:TYR:CD2	1:D:213:VAL:HG23	2.44	0.53
1:D:343:GLN:HA	1:D:346:VAL:HG22	1.90	0.53
1:F:429:LEU:HB3	1:F:440:ILE:HG21	1.91	0.53
1:G:193:MET:HE1	1:G:372:LEU:HA	1.89	0.53
1:G:287:ALA:HA	1:G:345:ARG:HH21	1.72	0.53
1:G:346:VAL:HA	1:G:349:ILE:HD12	1.90	0.53
1:I:199:TYR:CE1	1:I:205:ILE:HD11	2.43	0.53
1:I:348:GLN:O	1:I:351:GLN:NE2	2.41	0.53
1:K:155:ASP:OD2	1:K:395:ARG:HD2	2.08	0.53
1:K:177:VAL:HA	1:K:379:ILE:HB	1.89	0.53
1:M:20:VAL:HG22	1:M:74:VAL:HG21	1.90	0.53
1:M:82:ASN:O	1:M:86:GLY:N	2.30	0.53
2:O:13:LYS:HG2	2:O:41:LEU:HD21	1.91	0.53

Continued on next page...

For Manuscript Review

Continued from previous page...

Atom-1	Atom-2	Interatomic distance (Å)	Clash overlap (Å)
1:A:193:MET:HB2	1:A:332:ILE:HB	1.90	0.53
1:C:29:VAL:O	1:C:36:ARG:N	2.39	0.53
1:C:431:GLY:N	1:C:437:ASN:OD1	2.41	0.53
1:C:458:CYS:SG	1:C:480:ALA:HB1	2.48	0.53
1:F:30:THR:HB	1:F:51:LYS:HG2	1.89	0.53
1:F:223:ALA:HA	1:F:301:ILE:HB	1.89	0.53
1:F:411:VAL:HG21	1:F:494:LEU:HD22	1.90	0.53
1:F:458:CYS:SG	1:F:480:ALA:HB1	2.49	0.53
1:F:468:THR:HB	1:F:485:TYR:CE1	2.43	0.53
1:G:287:ALA:HB1	1:G:368:ARG:CZ	2.39	0.53
1:I:230:ILE:O	1:I:234:LEU:N	2.41	0.53
1:I:417:VAL:HG21	1:I:488:MET:HG2	1.89	0.53
1:J:5:ASP:HB2	1:J:524:LEU:HD23	1.89	0.53
1:K:12:ALA:O	1:K:16:MET:HG2	2.08	0.53
1:L:131:LEU:HG	1:L:497:THR:HG23	1.89	0.53
1:A:479:ASN:ND2	1:A:491:MET:HG3	2.23	0.53
1:C:415:GLY:HA2	3:C:601:ATP:H1’	1.90	0.53
1:E:458:CYS:SG	1:E:480:ALA:HB1	2.48	0.53
1:H:31:LEU:HD13	1:H:90:THR:HB	1.90	0.53
1:I:291:ASP:OD1	1:I:345:ARG:NE	2.41	0.53
1:J:203:TYR:HE2	1:K:181:THR:HA	1.73	0.53
1:K:323:VAL:HA	1:K:332:ILE:HA	1.89	0.53
1:K:479:ASN:O	1:K:483:GLU:N	2.42	0.53
1:N:218:PRO:HB3	1:N:246:PRO:HG2	1.90	0.53
2:O:65:VAL:HG23	2:O:67:PHE:HD1	1.72	0.53
2:R:55:LYS:HE3	2:S:48:ILE:HG21	1.91	0.53
1:D:140:ASP:N	1:D:140:ASP:OD1	2.40	0.53
1:G:20:VAL:HG13	1:G:74:VAL:HG21	1.89	0.53
1:K:218:PRO:HB3	1:K:246:PRO:HG2	1.91	0.53
1:M:165:ALA:HB2	1:M:187:LEU:HD22	1.90	0.53
1:N:247:LEU:HG	1:N:249:ILE:HD11	1.90	0.53
1:A:206:ASN:HD21	1:A:214:GLU:HB3	1.73	0.53
1:B:205:ILE:HA	1:B:213:VAL:HG22	1.91	0.53
1:F:190:VAL:O	1:F:376:VAL:N	2.39	0.53
1:F:364:LYS:O	1:F:368:ARG:HG3	2.08	0.53
1:H:350:ARG:HD3	1:H:353:ILE:HD12	1.91	0.53
1:I:122:LYS:HZ3	1:I:431:GLY:HA2	1.73	0.53
1:J:161:LEU:HG	1:J:187:LEU:HD23	1.90	0.53
1:M:149:THR:OG1	1:M:156:GLU:HA	2.09	0.53
1:M:161:LEU:HG	1:M:187:LEU:HD23	1.90	0.53
1:M:365:LEU:HD23	1:M:368:ARG:HE	1.72	0.53

Continued on next page...

For Manuscript Review

Continued from previous page...

Atom-1	Atom-2	Interatomic distance (Å)	Clash overlap (Å)
2:T:47:ARG:O	2:T:55:LYS:N	2.35	0.53
1:A:158:VAL:HG11	1:A:396:VAL:HA	1.90	0.53
1:D:479:ASN:O	1:D:483:GLU:N	2.42	0.53
1:G:228:SER:O	1:G:258:ALA:HB2	2.09	0.53
1:G:230:ILE:HD12	1:G:230:ILE:H	1.73	0.53
1:I:16:MET:O	1:I:20:VAL:HG23	2.09	0.53
1:J:124:VAL:HG21	1:J:508:ALA:HB2	1.90	0.53
1:K:284:ARG:HB3	1:K:284:ARG:CZ	2.39	0.53
1:N:33:PRO:HD3	6:N:601:ADP:C4	2.44	0.53
1:N:178:GLU:N	1:N:379:ILE:O	2.21	0.53
2:P:66:ILE:HD11	2:Q:3:ILE:HG21	1.91	0.53
1:B:322:ARG:HB3	1:B:333:ILE:HB	1.91	0.53
1:D:65:LYS:O	1:D:69:MET:HG3	2.08	0.53
1:E:219:PHE:O	1:E:248:LEU:N	2.39	0.53
1:E:219:PHE:CZ	1:E:245:LYS:HE2	2.44	0.53
1:F:5:ASP:N	1:F:522:THR:O	2.26	0.53
1:F:226:LYS:HZ2	1:F:255:GLU:HG3	1.74	0.53
1:L:89:THR:N	6:L:601:ADP:O3B	2.41	0.53
1:L:324:VAL:N	1:L:331:THR:O	2.38	0.53
1:N:81:ALA:O	1:N:85:ALA:HB3	2.09	0.53
1:N:193:MET:HG2	1:N:295:LEU:HD13	1.89	0.53
2:O:47:ARG:N	2:O:55:LYS:O	2.42	0.53
2:O:64:ILE:O	2:O:95:VAL:N	2.35	0.53
2:T:47:ARG:NH2	2:T:88:GLU:HB3	2.24	0.53
1:A:197:ARG:HD2	1:A:277:LYS:HB2	1.91	0.53
1:A:429:LEU:HB3	1:A:440:ILE:HG21	1.90	0.53
1:B:189:VAL:HA	1:B:377:ALA:HA	1.90	0.53
1:C:205:ILE:HA	1:C:213:VAL:HG22	1.91	0.53
1:D:239:ALA:HA	1:D:242:LYS:HE2	1.91	0.53
1:E:479:ASN:HB3	1:E:484:GLU:HG2	1.90	0.53
1:G:421:ARG:HH12	1:G:470:LYS:HA	1.74	0.53
1:G:431:GLY:N	1:G:437:ASN:OD1	2.40	0.53
1:H:124:VAL:HG21	1:H:508:ALA:HB2	1.91	0.53
1:I:262:LEU:HD13	1:I:273:VAL:HG11	1.90	0.53
1:J:39:VAL:HA	1:J:49:ILE:HA	1.90	0.53
1:J:195:PHE:HB3	1:J:371:LYS:HE3	1.91	0.53
1:K:215:LEU:HB2	1:K:323:VAL:HG22	1.90	0.53
1:L:455:VAL:HG22	1:L:478:TYR:CE2	2.44	0.53
1:M:85:ALA:HB2	1:M:502:SER:HB2	1.91	0.53
1:M:116:LEU:HG	1:M:435:ASP:OD1	2.09	0.53
1:N:349:ILE:HG12	1:N:368:ARG:NH2	2.24	0.53

Continued on next page...

For Manuscript Review

Continued from previous page...

Atom-1	Atom-2	Interatomic distance (Å)	Clash overlap (Å)
1:N:351:GLN:NE2	1:N:352:GLN:HG3	2.24	0.53
2:Q:10:VAL:HG22	2:Q:43:VAL:HG12	1.90	0.53
1:A:230:ILE:H	1:A:230:ILE:HD12	1.73	0.52
1:B:409:GLU:OE2	1:B:501:ARG:NE	2.40	0.52
1:B:429:LEU:O	1:B:430:ARG:NH1	2.37	0.52
1:C:352:GLN:HB3	1:C:365:LEU:HD13	1.92	0.52
1:D:232:GLU:HB3	1:D:309:LEU:HD23	1.91	0.52
1:D:452:ARG:HG3	1:D:462:PRO:HB2	1.91	0.52
1:F:176:THR:O	1:F:379:ILE:N	2.31	0.52
1:G:153:ASN:HD22	1:G:395:ARG:HD3	1.74	0.52
1:G:221:LEU:HD23	1:G:249:ILE:HG12	1.92	0.52
1:I:489:ILE:HA	1:I:494:LEU:HD21	1.90	0.52
1:L:345:ARG:O	1:L:349:ILE:HG13	2.09	0.52
1:N:190:VAL:O	1:N:376:VAL:N	2.42	0.52
1:N:352:GLN:HA	1:N:355:GLU:HG3	1.91	0.52
1:A:180:GLY:N	1:A:381:VAL:O	2.35	0.52
1:A:232:GLU:HA	1:A:310:GLU:HG3	1.90	0.52
1:D:364:LYS:O	1:D:368:ARG:HG3	2.10	0.52
1:D:455:VAL:HG13	1:D:460:GLU:HB2	1.90	0.52
1:E:154:SER:N	7:E:720:HOH:O	2.42	0.52
1:F:427:ALA:O	1:F:441:LYS:NZ	2.42	0.52
1:F:479:ASN:HB3	1:F:484:GLU:HG2	1.92	0.52
1:G:343:GLN:HA	1:G:346:VAL:HG22	1.89	0.52
1:H:262:LEU:HD22	1:H:273:VAL:HG11	1.91	0.52
1:I:433:ASN:H	1:I:436:GLN:HB2	1.73	0.52
1:L:61:GLU:OE2	1:L:72:GLN:NE2	2.43	0.52
1:L:221:LEU:HB3	1:L:249:ILE:HA	1.90	0.52
1:N:149:THR:OG1	1:N:156:GLU:HA	2.08	0.52
2:O:76:GLU:HG3	2:U:66:ILE:HG21	1.91	0.52
2:R:68:ASN:N	2:R:90:ASP:O	2.35	0.52
2:T:59:VAL:HG22	2:T:94:ILE:HD11	1.90	0.52
1:C:102:GLU:OE1	1:C:445:ARG:NE	2.28	0.52
1:C:323:VAL:HG22	1:C:332:ILE:HA	1.90	0.52
1:D:349:ILE:HG23	1:D:365:LEU:HB3	1.91	0.52
1:E:128:VAL:HG13	1:E:501:ARG:HG3	1.90	0.52
1:E:193:MET:HG2	1:E:295:LEU:HD22	1.89	0.52
1:E:213:VAL:HG11	1:E:274:ALA:HB2	1.91	0.52
1:E:417:VAL:HG21	1:E:477:GLY:HA3	1.91	0.52
1:H:421:ARG:NH1	1:H:469:VAL:O	2.40	0.52
1:I:89:THR:N	6:I:601:ADP:O3B	2.41	0.52
1:I:102:GLU:HB2	1:I:442:VAL:HG13	1.92	0.52

Continued on next page...

For Manuscript Review

Continued from previous page...

Atom-1	Atom-2	Interatomic distance (Å)	Clash overlap (Å)
1:J:215:LEU:HB2	1:J:323:VAL:HG22	1.90	0.52
1:J:501:ARG:NH1	1:J:505:GLN:OE1	2.41	0.52
1:K:345:ARG:NH2	1:K:368:ARG:HH12	2.07	0.52
1:K:368:ARG:O	1:K:372:LEU:HD23	2.09	0.52
1:L:324:VAL:O	1:L:331:THR:N	2.25	0.52
1:L:411:VAL:HA	1:L:496:PRO:HA	1.90	0.52
1:N:166:MET:HB2	1:N:171:LYS:HA	1.90	0.52
1:N:219:PHE:CE2	1:N:314:LEU:HD22	2.44	0.52
2:O:10:VAL:HG22	2:O:43:VAL:HG22	1.91	0.52
2:O:69:ASP:HA	2:O:73:VAL:HG21	1.90	0.52
1:A:278:ALA:HB3	1:A:285:ARG:HE	1.74	0.52
1:A:356:ALA:HB1	1:A:361:ASP:HB2	1.91	0.52
1:B:31:LEU:O	1:B:457:ASN:ND2	2.26	0.52
1:C:381:VAL:HG21	1:C:393:LYS:HA	1.90	0.52
1:F:124:VAL:HG21	1:F:508:ALA:HB2	1.91	0.52
1:G:397:GLU:O	1:G:401:HIS:ND1	2.43	0.52
1:H:345:ARG:NH2	1:H:368:ARG:HH12	2.07	0.52
1:J:433:ASN:H	1:J:436:GLN:HB2	1.73	0.52
1:K:419:LEU:HD22	1:K:447:MET:HG3	1.90	0.52
1:L:301:ILE:HG12	1:L:307:MET:HE1	1.91	0.52
1:N:31:LEU:HD13	1:N:90:THR:HB	1.91	0.52
1:N:224:ASP:OD2	1:N:286:LYS:HG2	2.09	0.52
1:N:411:VAL:HA	1:N:496:PRO:HA	1.92	0.52
1:B:397:GLU:O	1:B:401:HIS:ND1	2.43	0.52
1:D:421:ARG:O	1:D:425:LYS:HG3	2.09	0.52
1:E:163:ALA:HA	1:E:166:MET:HE3	1.92	0.52
1:E:295:LEU:HA	1:E:342:ILE:HD11	1.89	0.52
1:F:158:VAL:HG11	1:F:396:VAL:HA	1.91	0.52
1:G:124:VAL:HG13	1:G:504:LEU:HG	1.90	0.52
1:G:209:GLU:HG2	1:G:210:THR:HG23	1.91	0.52
1:H:68:ASN:O	1:H:72:GLN:HG2	2.10	0.52
1:J:221:LEU:HB3	1:J:249:ILE:HA	1.90	0.52
1:K:240:VAL:HG21	1:K:247:LEU:HD13	1.90	0.52
1:M:218:PRO:HG3	1:M:323:VAL:HG22	1.91	0.52
1:M:284:ARG:CZ	1:M:284:ARG:HB3	2.39	0.52
2:O:59:VAL:HG21	2:O:91:ILE:HG21	1.91	0.52
1:A:219:PHE:CZ	1:A:245:LYS:HE2	2.44	0.52
1:C:222:LEU:O	1:C:301:ILE:N	2.25	0.52
1:D:135:SER:HB3	1:D:497:THR:HG21	1.91	0.52
1:E:414:GLY:HA3	1:E:493:ILE:HG22	1.91	0.52
1:F:16:MET:HE1	1:G:39:VAL:HG11	1.92	0.52

Continued on next page...

For Manuscript Review

Continued from previous page...

Atom-1	Atom-2	Interatomic distance (Å)	Clash overlap (Å)
1:H:144:ILE:HG23	1:H:403:THR:HB	1.92	0.52
1:H:186:GLU:O	1:H:380:LYS:N	2.30	0.52
1:I:215:LEU:HB3	1:I:246:PRO:HB2	1.91	0.52
1:I:235:PRO:HG3	1:I:310:GLU:HA	1.92	0.52
1:J:32:GLY:HA3	1:J:454:ILE:HG23	1.92	0.52
1:J:190:VAL:O	1:J:376:VAL:N	2.42	0.52
1:K:124:VAL:HG21	1:K:508:ALA:HB2	1.92	0.52
1:L:69:MET:HG2	1:L:520:MET:CE	2.40	0.52
1:L:192:GLY:HA3	1:L:376:VAL:HG13	1.91	0.52
1:L:349:ILE:HG21	1:L:368:ARG:HB2	1.90	0.52
2:O:20:LYS:NZ	2:O:24:GLY:HA2	2.25	0.52
2:U:43:VAL:HG13	2:U:57:LEU:HD22	1.92	0.52
2:U:59:VAL:HG22	2:U:94:ILE:HD11	1.92	0.52
1:B:219:PHE:HD2	1:B:240:VAL:HG22	1.73	0.52
1:C:128:VAL:HG13	1:C:501:ARG:HG3	1.92	0.52
1:C:175:ILE:HB	1:C:404:ARG:HH12	1.74	0.52
1:C:193:MET:HG2	1:C:295:LEU:HD22	1.91	0.52
1:C:230:ILE:HD11	1:C:258:ALA:HA	1.91	0.52
1:F:31:LEU:HB2	1:F:90:THR:CG2	2.39	0.52
1:G:5:ASP:N	1:G:522:THR:O	2.36	0.52
1:G:140:ASP:OD1	1:G:140:ASP:N	2.42	0.52
1:H:241:ALA:HB2	1:H:271:VAL:HG22	1.91	0.52
1:I:345:ARG:O	1:I:349:ILE:HG13	2.10	0.52
1:I:461:GLU:HG3	1:I:464:VAL:H	1.75	0.52
1:K:122:LYS:NZ	1:K:432:GLN:OE1	2.42	0.52
1:K:345:ARG:O	1:K:349:ILE:HG13	2.09	0.52
1:K:420:ILE:HG12	1:K:448:GLU:HG2	1.91	0.52
1:M:349:ILE:O	1:M:353:ILE:HG13	2.10	0.52
1:N:215:LEU:HB2	1:N:323:VAL:HG22	1.91	0.52
1:N:429:LEU:HD23	1:N:440:ILE:HG12	1.92	0.52
1:A:239:ALA:HA	1:A:242:LYS:HE2	1.91	0.52
1:B:151:SER:HB2	1:B:399:ALA:HA	1.92	0.52
1:B:186:GLU:HG2	1:B:380:LYS:HB2	1.92	0.52
1:D:213:VAL:HG11	1:D:274:ALA:HB2	1.91	0.52
1:E:250:ILE:HD13	1:E:292:ILE:HD13	1.92	0.52
1:E:343:GLN:HA	1:E:346:VAL:HG22	1.91	0.52
1:F:190:VAL:N	1:F:376:VAL:O	2.33	0.52
1:F:325:ILE:HG13	1:F:330:THR:HG23	1.90	0.52
1:G:261:THR:OG1	2:U:28:THR:O	2.24	0.52
1:G:452:ARG:HH21	1:G:470:LYS:HZ1	1.58	0.52
1:H:76:GLU:HG2	1:H:80:LYS:HE3	1.92	0.52

Continued on next page...

For Manuscript Review

Continued from previous page...

Atom-1	Atom-2	Interatomic distance (Å)	Clash overlap (Å)
1:H:440:ILE:O	1:H:444:LEU:HG	2.10	0.52
1:J:195:PHE:HZ	1:J:250:ILE:HD13	1.74	0.52
1:K:348:GLN:O	1:K:352:GLN:HG3	2.10	0.52
1:M:326:ASN:N	1:M:329:THR:O	2.24	0.52
1:N:77:VAL:HG13	1:N:506:TYR:HB3	1.91	0.52
1:N:322:ARG:HB2	1:N:333:ILE:HB	1.92	0.52
2:Q:37:ARG:HH12	2:R:3:ILE:HD11	1.75	0.52
1:A:39:VAL:O	1:G:520:MET:HA	2.10	0.52
1:A:135:SER:HB3	1:A:497:THR:HG21	1.92	0.52
1:D:31:LEU:HB2	1:D:90:THR:CG2	2.38	0.52
1:D:458:CYS:SG	1:D:480:ALA:HB1	2.50	0.52
1:F:214:GLU:HG3	1:F:324:VAL:HG22	1.91	0.52
1:G:239:ALA:HA	1:G:242:LYS:HE2	1.92	0.52
1:I:204:PHE:HE2	1:I:275:ALA:HB3	1.75	0.52
1:J:31:LEU:HD13	1:J:90:THR:HB	1.92	0.52
1:K:222:LEU:HD23	1:K:250:ILE:HB	1.91	0.52
1:K:433:ASN:OD1	1:K:434:GLU:N	2.43	0.52
1:L:132:LYS:NZ	1:L:409:GLU:OE2	2.39	0.52
1:N:115:ASP:O	1:N:436:GLN:HG2	2.10	0.52
2:S:11:ILE:HG12	2:S:85:ILE:HG12	1.91	0.52
2:U:26:VAL:HG12	2:U:28:THR:HG23	1.92	0.52
2:U:64:ILE:O	2:U:95:VAL:N	2.41	0.52
1:A:205:ILE:HD13	1:A:211:GLY:HA2	1.91	0.52
1:B:163:ALA:HA	1:B:166:MET:HE3	1.92	0.52
1:B:194:GLN:HG3	1:B:331:THR:HG22	1.92	0.52
1:G:31:LEU:HB2	1:G:90:THR:CG2	2.40	0.52
1:G:214:GLU:OE2	1:G:322:ARG:NH1	2.43	0.52
1:G:364:LYS:O	1:G:368:ARG:HG3	2.09	0.52
1:G:414:GLY:HA3	1:G:493:ILE:HG22	1.92	0.52
1:H:477:GLY:N	1:H:486:GLY:O	2.39	0.52
1:I:85:ALA:HB2	1:I:502:SER:HB2	1.92	0.52
1:I:115:ASP:O	1:I:436:GLN:HG2	2.10	0.52
1:L:81:ALA:O	1:L:85:ALA:HB3	2.10	0.52
1:L:475:ASN:HB2	1:L:487:ASN:ND2	2.25	0.52
1:M:31:LEU:HD13	1:M:90:THR:HB	1.92	0.52
1:N:66:PHE:HB3	1:N:520:MET:HE1	1.92	0.52
1:N:349:ILE:HD13	1:N:368:ARG:HB3	1.91	0.52
1:N:414:GLY:HA3	1:N:493:ILE:HG22	1.92	0.52
1:N:479:ASN:O	1:N:483:GLU:N	2.43	0.52
1:A:149:THR:OG1	1:A:156:GLU:HA	2.09	0.51
1:B:386:GLU:O	1:B:390:LYS:HG3	2.11	0.51

Continued on next page...

For Manuscript Review

Continued from previous page...

Atom-1	Atom-2	Interatomic distance (Å)	Clash overlap (Å)
1:C:68:ASN:O	1:C:72:GLN:HG2	2.11	0.51
1:E:479:ASN:ND2	1:E:491:MET:HG3	2.24	0.51
1:F:230:ILE:HA	1:F:233:MET:HE2	1.92	0.51
1:F:448:GLU:OE1	1:F:470:LYS:NZ	2.41	0.51
1:G:421:ARG:O	1:G:425:LYS:HG3	2.10	0.51
1:H:115:ASP:O	1:H:436:GLN:HG2	2.09	0.51
1:H:455:VAL:HG22	1:H:478:TYR:CE2	2.45	0.51
1:I:221:LEU:N	1:I:248:LEU:O	2.28	0.51
1:L:85:ALA:HB2	1:L:502:SER:HB2	1.91	0.51
1:N:101:THR:HG22	1:N:105:LYS:HE2	1.91	0.51
2:R:77:LYS:HD2	2:R:80:ASN:HA	1.91	0.51
1:B:261:THR:HG21	2:P:27:LEU:HD13	1.92	0.51
1:B:417:VAL:HG21	1:B:477:GLY:HA3	1.92	0.51
1:C:283:ASP:OD1	1:C:284:ARG:N	2.43	0.51
1:C:313:THR:O	1:C:317:LEU:HG	2.09	0.51
1:C:314:LEU:HA	1:C:317:LEU:HD12	1.92	0.51
1:E:169:VAL:HB	1:E:377:ALA:HB2	1.92	0.51
1:F:68:ASN:O	1:F:72:GLN:HG2	2.09	0.51
1:G:465:VAL:HA	1:G:485:TYR:OH	2.10	0.51
1:I:368:ARG:O	1:I:372:LEU:HD23	2.10	0.51
1:J:350:ARG:HA	1:J:353:ILE:HD12	1.92	0.51
1:M:34:LYS:HB2	1:M:458:CYS:SG	2.51	0.51
1:M:351:GLN:HA	1:M:354:GLU:CD	2.31	0.51
1:M:498:LYS:HG3	1:M:501:ARG:HH21	1.74	0.51
1:A:71:ALA:HA	1:A:74:VAL:HG12	1.92	0.51
1:A:421:ARG:O	1:A:425:LYS:HG3	2.10	0.51
1:C:31:LEU:HB2	1:C:90:THR:CG2	2.38	0.51
1:G:472:GLY:HA3	1:G:476:TYR:CD2	2.44	0.51
1:H:197:ARG:NH1	1:H:277:LYS:HD3	2.26	0.51
1:J:247:LEU:HG	1:J:249:ILE:HD11	1.92	0.51
1:L:30:THR:HA	1:L:35:GLY:HA3	1.93	0.51
1:M:345:ARG:O	1:M:349:ILE:HG13	2.10	0.51
1:N:279:PRO:O	1:N:285:ARG:HA	2.10	0.51
1:N:437:ASN:O	1:N:441:LYS:HG2	2.10	0.51
2:R:12:VAL:O	2:R:84:LEU:N	2.37	0.51
2:T:65:VAL:HG21	2:T:91:ILE:HD12	1.92	0.51
1:A:178:GLU:N	1:A:379:ILE:O	2.32	0.51
1:C:102:GLU:HB2	1:C:442:VAL:HG13	1.92	0.51
1:E:199:TYR:CD1	1:E:213:VAL:HG23	2.45	0.51
1:G:320:ALA:HA	1:G:335:GLY:HA2	1.93	0.51
1:H:342:ILE:HG23	1:H:372:LEU:HD12	1.92	0.51

Continued on next page...

For Manuscript Review

Continued from previous page...

Atom-1	Atom-2	Interatomic distance (Å)	Clash overlap (Å)
1:J:475:ASN:HB2	1:J:487:ASN:ND2	2.25	0.51
1:K:174:VAL:HG11	1:K:376:VAL:HG12	1.91	0.51
1:K:219:PHE:CE2	1:K:314:LEU:HD22	2.45	0.51
1:K:247:LEU:HG	1:K:249:ILE:HD11	1.92	0.51
1:M:39:VAL:HG22	1:M:49:ILE:HG23	1.92	0.51
1:M:95:LEU:O	1:M:99:ILE:HG13	2.11	0.51
1:M:204:PHE:HD1	1:M:266:THR:HG21	1.76	0.51
2:O:9:ARG:HB3	2:O:85:ILE:HD11	1.93	0.51
2:R:47:ARG:N	2:R:55:LYS:O	2.43	0.51
2:S:46:GLY:HA2	2:S:57:LEU:HD12	1.92	0.51
2:S:47:ARG:N	2:S:55:LYS:O	2.43	0.51
1:A:452:ARG:NH1	7:A:2013:HOH:O	2.29	0.51
1:B:230:ILE:HD12	1:B:230:ILE:H	1.75	0.51
1:C:150:ILE:HG23	3:C:601:ATP:C8	2.45	0.51
1:C:215:LEU:HD12	1:C:248:LEU:HB2	1.93	0.51
1:D:215:LEU:HD12	1:D:248:LEU:HB2	1.92	0.51
1:F:186:GLU:N	1:F:380:LYS:O	2.42	0.51
1:F:291:ASP:OD1	1:F:372:LEU:HD13	2.10	0.51
1:H:279:PRO:O	1:H:285:ARG:HA	2.11	0.51
1:H:472:GLY:HA3	1:H:476:TYR:CD2	2.45	0.51
1:I:27:VAL:HG12	1:I:90:THR:HG23	1.92	0.51
1:I:356:ALA:HB3	1:I:362:ARG:HH21	1.76	0.51
1:I:438:VAL:O	1:I:442:VAL:HG23	2.11	0.51
1:I:514:MET:HG3	1:I:514:MET:O	2.11	0.51
1:K:137:PRO:HA	1:K:410:GLY:HA2	1.92	0.51
1:K:472:GLY:HA3	1:K:476:TYR:CD2	2.45	0.51
1:M:122:LYS:HZ2	1:M:431:GLY:HA2	1.75	0.51
1:N:32:GLY:HA3	1:N:454:ILE:HG23	1.91	0.51
1:N:39:VAL:HA	1:N:49:ILE:HA	1.92	0.51
1:N:131:LEU:HD21	1:N:500:THR:HB	1.93	0.51
1:D:240:VAL:HG11	1:D:247:LEU:HB2	1.92	0.51
1:D:352:GLN:O	1:D:356:ALA:N	2.44	0.51
3:D:601:ATP:O1G	7:D:2001:HOH:O	2.19	0.51
1:F:71:ALA:HA	1:F:74:VAL:HG12	1.92	0.51
1:F:417:VAL:HG21	1:F:477:GLY:HA3	1.92	0.51
1:G:11:ASP:O	1:G:15:LYS:HG2	2.11	0.51
1:H:81:ALA:O	1:H:85:ALA:HB2	2.10	0.51
1:H:262:LEU:HB3	1:H:273:VAL:HG11	1.93	0.51
1:J:64:ASP:HB3	1:J:67:GLU:HB2	1.93	0.51
1:J:325:ILE:O	1:J:325:ILE:HG13	2.09	0.51
1:K:440:ILE:O	1:K:444:LEU:HG	2.11	0.51

Continued on next page...

For Manuscript Review

Continued from previous page...

Atom-1	Atom-2	Interatomic distance (Å)	Clash overlap (Å)
1:N:144:ILE:HD13	1:N:166:MET:SD	2.50	0.51
1:N:406:ALA:HB2	1:N:496:PRO:HG3	1.92	0.51
1:B:124:VAL:HG13	1:B:504:LEU:HG	1.91	0.51
1:B:200:LEU:HD21	1:B:277:LYS:HG3	1.92	0.51
1:B:250:ILE:HG23	1:B:278:ALA:HA	1.93	0.51
1:B:498:LYS:HG3	1:B:501:ARG:HH21	1.75	0.51
1:F:196:ASP:HA	1:F:329:THR:HA	1.91	0.51
1:F:519:CYS:HB3	1:G:38:VAL:HG22	1.92	0.51
1:G:175:ILE:HB	1:G:404:ARG:HH12	1.75	0.51
1:G:414:GLY:H	1:G:488:MET:HB3	1.76	0.51
1:H:85:ALA:HB2	1:H:502:SER:HB2	1.91	0.51
1:K:40:LEU:N	1:K:48:THR:O	2.44	0.51
1:M:32:GLY:HA3	1:M:454:ILE:HG23	1.92	0.51
1:N:345:ARG:O	1:N:349:ILE:HG13	2.10	0.51
1:A:31:LEU:HB2	1:A:90:THR:CG2	2.40	0.51
1:A:150:ILE:HG23	3:A:601:ATP:C8	2.46	0.51
1:A:231:ARG:HA	1:A:234:LEU:HG	1.93	0.51
1:B:5:ASP:N	1:B:522:THR:O	2.29	0.51
1:C:200:LEU:HD12	1:C:275:ALA:HB1	1.91	0.51
1:D:144:ILE:HG23	1:D:403:THR:HB	1.93	0.51
1:D:148:GLY:HA2	1:D:399:ALA:HB1	1.93	0.51
1:E:147:VAL:HG12	1:E:402:ALA:HB1	1.93	0.51
1:F:230:ILE:HG22	1:F:234:LEU:HD22	1.93	0.51
1:F:511:ALA:O	1:F:515:ILE:HG12	2.11	0.51
1:G:16:MET:HE1	1:G:517:THR:HG21	1.93	0.51
1:G:417:VAL:HG21	1:G:477:GLY:HA3	1.93	0.51
1:H:14:VAL:O	1:H:18:ARG:HG3	2.10	0.51
1:H:57:ALA:HA	1:H:60:ILE:HD12	1.93	0.51
1:H:213:VAL:HB	1:H:325:ILE:HG12	1.91	0.51
1:I:279:PRO:O	1:I:285:ARG:HA	2.11	0.51
1:J:301:ILE:HG21	1:J:309:LEU:HD23	1.93	0.51
1:L:40:LEU:N	1:L:48:THR:O	2.42	0.51
1:L:57:ALA:HA	1:L:60:ILE:HD12	1.91	0.51
2:P:10:VAL:HG11	2:P:40:VAL:HG12	1.93	0.51
1:A:150:ILE:HG13	1:A:493:ILE:HA	1.92	0.51
1:C:158:VAL:HG11	1:C:396:VAL:HA	1.92	0.51
1:C:517:THR:HG21	1:D:39:VAL:HG23	1.93	0.51
1:D:206:ASN:HD21	1:D:214:GLU:HB3	1.76	0.51
1:E:190:VAL:O	1:E:376:VAL:N	2.38	0.51
1:F:397:GLU:O	1:F:401:HIS:ND1	2.44	0.51
1:J:429:LEU:HG	1:J:440:ILE:HD13	1.93	0.51

Continued on next page...

For Manuscript Review

Continued from previous page...

Atom-1	Atom-2	Interatomic distance (Å)	Clash overlap (Å)
1:L:224:ASP:OD2	1:L:286:LYS:HG2	2.10	0.51
1:M:69:MET:HE1	1:N:39:VAL:HG11	1.92	0.51
1:M:158:VAL:HG13	1:M:396:VAL:HA	1.93	0.51
1:N:84:ALA:O	1:N:502:SER:OG	2.28	0.51
2:U:59:VAL:HG21	2:U:91:ILE:HG21	1.93	0.51
1:A:207:LYS:HZ3	1:A:214:GLU:HB2	1.75	0.51
1:B:264:VAL:O	1:B:268:ARG:HG2	2.11	0.51
1:B:352:GLN:O	1:B:356:ALA:N	2.44	0.51
1:C:196:ASP:HA	1:C:329:THR:HA	1.93	0.51
1:H:351:GLN:NE2	1:H:352:GLN:HG3	2.26	0.51
1:I:81:ALA:O	1:I:85:ALA:HB3	2.10	0.51
1:K:56:VAL:HG12	1:K:60:ILE:HD11	1.92	0.51
1:L:429:LEU:HB3	1:L:440:ILE:HG21	1.93	0.51
1:M:178:GLU:N	1:M:379:ILE:O	2.29	0.51
1:N:346:VAL:O	1:N:350:ARG:HG2	2.11	0.51
1:N:368:ARG:O	1:N:372:LEU:HD23	2.11	0.51
1:A:283:ASP:OD1	1:A:284:ARG:N	2.44	0.50
1:B:364:LYS:O	1:B:368:ARG:HG3	2.10	0.50
1:B:498:LYS:HG3	1:B:501:ARG:NH2	2.26	0.50
1:C:220:ILE:HG13	1:C:248:LEU:HD23	1.92	0.50
1:H:70:GLY:HA2	1:H:73:MET:HE1	1.94	0.50
1:I:284:ARG:NE	1:I:364:LYS:HB3	2.26	0.50
1:K:81:ALA:O	1:K:85:ALA:HB3	2.10	0.50
1:M:465:VAL:HA	1:M:485:TYR:OH	2.11	0.50
1:N:124:VAL:HG11	1:N:508:ALA:HB2	1.93	0.50
1:N:290:GLN:OE1	1:N:294:THR:OG1	2.30	0.50
1:A:200:LEU:N	1:A:275:ALA:O	2.37	0.50
1:C:190:VAL:O	1:C:376:VAL:N	2.36	0.50
1:E:213:VAL:HB	1:E:325:ILE:HB	1.94	0.50
1:E:219:PHE:CD2	1:E:240:VAL:HG22	2.46	0.50
1:E:240:VAL:HG21	1:E:247:LEU:HD12	1.92	0.50
1:G:136:VAL:HG23	1:G:411:VAL:HG23	1.94	0.50
1:G:458:CYS:SG	1:G:480:ALA:HB1	2.51	0.50
1:H:16:MET:O	1:H:20:VAL:HG23	2.11	0.50
1:I:6:VAL:HG13	1:I:521:VAL:HG22	1.92	0.50
1:J:27:VAL:HG12	1:J:90:THR:HG23	1.94	0.50
1:J:293:ALA:O	1:J:298:GLY:N	2.45	0.50
1:J:320:ALA:HA	1:J:336:VAL:H	1.75	0.50
1:J:460:GLU:O	1:J:462:PRO:HD3	2.11	0.50
1:L:186:GLU:HB3	1:L:380:LYS:HB2	1.93	0.50
1:N:38:VAL:O	1:N:50:THR:N	2.34	0.50

Continued on next page...

For Manuscript Review

Continued from previous page...

Atom-1	Atom-2	Interatomic distance (Å)	Clash overlap (Å)
1:N:345:ARG:HH21	1:N:368:ARG:HH12	1.57	0.50
2:P:86:MET:HG2	2:P:87:SER:O	2.11	0.50
2:U:40:VAL:HG23	2:U:62:GLY:H	1.76	0.50
1:C:71:ALA:HA	1:C:74:VAL:HG12	1.93	0.50
1:D:287:ALA:HB1	1:D:368:ARG:CZ	2.41	0.50
1:E:386:GLU:O	1:E:390:LYS:HG3	2.11	0.50
1:E:522:THR:HG22	1:F:41:ASP:HB2	1.93	0.50
1:G:322:ARG:HB3	1:G:333:ILE:HB	1.93	0.50
1:I:472:GLY:HA3	1:I:476:TYR:CD2	2.47	0.50
1:J:414:GLY:HA3	1:J:493:ILE:HG22	1.94	0.50
1:J:468:THR:HB	1:J:485:TYR:CE2	2.46	0.50
1:L:368:ARG:O	1:L:372:LEU:HD23	2.12	0.50
1:A:352:GLN:O	1:A:356:ALA:N	2.45	0.50
1:B:193:MET:HG2	1:B:295:LEU:HD22	1.93	0.50
1:B:308:GLU:H	1:B:311:LYS:HD3	1.77	0.50
1:D:206:ASN:ND2	1:D:214:GLU:O	2.44	0.50
1:E:308:GLU:H	1:E:311:LYS:HD3	1.75	0.50
1:I:190:VAL:O	1:I:376:VAL:N	2.44	0.50
1:L:204:PHE:HE2	1:L:275:ALA:HB3	1.76	0.50
1:N:98:ALA:O	1:N:102:GLU:HG2	2.10	0.50
1:N:301:ILE:HG21	1:N:309:LEU:HD23	1.94	0.50
1:B:31:LEU:HB2	1:B:90:THR:CG2	2.39	0.50
1:B:228:SER:O	1:B:258:ALA:HB2	2.12	0.50
1:E:150:ILE:HG13	1:E:494:LEU:H	1.77	0.50
1:J:472:GLY:HA3	1:J:476:TYR:CD2	2.47	0.50
1:K:279:PRO:C	1:K:288:MET:HG3	2.31	0.50
1:L:200:LEU:HD21	1:L:277:LYS:HB2	1.92	0.50
1:N:197:ARG:HE	1:N:279:PRO:HA	1.76	0.50
1:N:262:LEU:HD22	1:N:273:VAL:HG21	1.93	0.50
1:N:322:ARG:O	1:N:333:ILE:N	2.31	0.50
1:A:219:PHE:CD2	1:A:240:VAL:HG22	2.41	0.50
1:A:430:ARG:HH22	1:A:441:LYS:HE2	1.75	0.50
1:A:511:ALA:O	1:A:515:ILE:HG12	2.11	0.50
1:B:204:PHE:HD1	1:B:266:THR:HG21	1.77	0.50
1:B:287:ALA:HB1	1:B:368:ARG:CZ	2.42	0.50
1:C:399:ALA:O	1:C:403:THR:HG23	2.12	0.50
1:E:421:ARG:O	1:E:425:LYS:HG3	2.12	0.50
1:F:28:LYS:HE2	1:F:94:VAL:HG22	1.93	0.50
1:G:453:GLN:NE2	1:G:457:ASN:OD1	2.45	0.50
1:I:356:ALA:O	1:I:362:ARG:NH2	2.45	0.50
1:J:197:ARG:NH1	1:J:277:LYS:HD3	2.26	0.50

Continued on next page...

For Manuscript Review

Continued from previous page...

Atom-1	Atom-2	Interatomic distance (Å)	Clash overlap (Å)
1:M:204:PHE:CD1	1:M:266:THR:HG21	2.47	0.50
2:Q:12:VAL:O	2:Q:84:LEU:N	2.45	0.50
1:A:179:ASP:OD1	1:A:393:LYS:HD2	2.11	0.50
1:A:223:ALA:HA	1:A:301:ILE:HB	1.93	0.50
1:B:102:GLU:OE1	1:B:445:ARG:NE	2.32	0.50
1:B:511:ALA:O	1:B:515:ILE:HG12	2.12	0.50
1:C:421:ARG:NH2	1:C:469:VAL:O	2.31	0.50
1:D:356:ALA:HB1	1:D:361:ASP:HB2	1.94	0.50
1:F:287:ALA:HA	1:F:345:ARG:NH2	2.27	0.50
1:G:429:LEU:HB3	1:G:440:ILE:HG21	1.93	0.50
1:I:15:LYS:O	1:I:67:GLU:HG2	2.11	0.50
1:L:163:ALA:O	1:L:167:ASP:HB2	2.12	0.50
1:M:204:PHE:HE2	1:M:275:ALA:HB3	1.77	0.50
1:N:188:ASP:OD1	1:N:188:ASP:N	2.45	0.50
1:N:197:ARG:NH1	1:N:277:LYS:HD3	2.26	0.50
2:R:43:VAL:HG13	2:R:57:LEU:HD22	1.94	0.50
1:C:213:VAL:HB	1:C:325:ILE:HB	1.92	0.50
1:D:291:ASP:O	1:D:294:THR:OG1	2.27	0.50
1:D:420:ILE:HG21	1:D:470:LYS:HG2	1.94	0.50
1:E:264:VAL:HG21	2:S:28:THR:HG21	1.93	0.50
1:G:185:ASP:HA	1:G:381:VAL:HA	1.94	0.50
1:G:232:GLU:HA	1:G:310:GLU:HG3	1.94	0.50
1:H:37:ASN:ND2	1:H:51:LYS:HE2	2.26	0.50
1:H:406:ALA:HB2	1:H:496:PRO:HG3	1.94	0.50
1:I:104:LEU:HD21	1:I:514:MET:HG2	1.93	0.50
1:I:192:GLY:HA3	1:I:376:VAL:HG22	1.93	0.50
1:I:417:VAL:CG1	1:I:477:GLY:HA3	2.41	0.50
1:J:17:LEU:HB2	1:J:104:LEU:HD12	1.94	0.50
1:J:451:LEU:HD21	1:J:465:VAL:HG12	1.94	0.50
1:K:98:ALA:O	1:K:102:GLU:HG2	2.12	0.50
1:K:144:ILE:HD12	1:K:166:MET:HE3	1.93	0.50
1:L:325:ILE:HG13	1:L:325:ILE:O	2.10	0.50
1:M:204:PHE:CE2	1:M:275:ALA:HB3	2.47	0.50
1:N:295:LEU:HA	1:N:342:ILE:HG12	1.94	0.50
1:B:32:GLY:HA2	3:B:601:ATP:O4’	2.11	0.50
1:B:87:ASP:OD1	1:B:88:GLY:N	2.41	0.50
1:D:386:GLU:O	1:D:390:LYS:HG3	2.11	0.50
1:F:138:CYS:HB2	1:F:411:VAL:HG13	1.94	0.50
1:H:248:LEU:HD22	1:H:323:VAL:HG21	1.94	0.50
1:I:150:ILE:HD12	6:I:601:ADP:N7	2.26	0.50
1:J:241:ALA:HB2	1:J:271:VAL:HG22	1.94	0.50

Continued on next page...

For Manuscript Review

Continued from previous page...

Atom-1	Atom-2	Interatomic distance (Å)	Clash overlap (Å)
1:J:421:ARG:NH1	1:J:469:VAL:O	2.44	0.50
1:K:144:ILE:HB	1:K:166:MET:HE3	1.93	0.50
1:L:106:ALA:O	1:L:111:MET:HG2	2.12	0.50
1:L:223:ALA:O	1:L:251:ALA:HA	2.12	0.50
1:L:235:PRO:HG3	1:L:310:GLU:HA	1.93	0.50
1:L:323:VAL:HG12	1:L:332:ILE:HG22	1.93	0.50
1:M:57:ALA:HA	1:M:60:ILE:HD12	1.93	0.50
1:N:204:PHE:HE2	1:N:275:ALA:HB3	1.76	0.50
2:R:66:ILE:HG21	2:S:76:GLU:HG2	1.93	0.50
2:U:13:LYS:N	2:U:39:GLU:O	2.43	0.50
1:A:151:SER:HB2	1:A:399:ALA:HA	1.94	0.49
1:D:259:LEU:O	1:D:263:VAL:HG13	2.12	0.49
1:D:448:GLU:HB3	1:D:452:ARG:NH1	2.27	0.49
1:H:176:THR:O	1:H:379:ILE:N	2.43	0.49
1:H:200:LEU:HD21	1:H:277:LYS:HB2	1.94	0.49
1:J:81:ALA:O	1:J:85:ALA:HB2	2.11	0.49
1:J:122:LYS:NZ	1:J:431:GLY:HA2	2.27	0.49
1:L:279:PRO:C	1:L:288:MET:HG3	2.32	0.49
1:L:423:ALA:HB2	1:L:447:MET:HB2	1.94	0.49
1:M:350:ARG:O	1:M:353:ILE:HB	2.11	0.49
1:B:321:LYS:HZ2	1:B:336:VAL:HG11	1.78	0.49
1:C:226:LYS:HZ3	1:C:253:ASP:HB3	1.77	0.49
1:F:214:GLU:OE2	1:F:322:ARG:NH1	2.46	0.49
1:H:47:PRO:CG	1:N:69:MET:HB2	2.41	0.49
1:I:31:LEU:HD23	1:I:453:GLN:HB3	1.94	0.49
1:I:197:ARG:NH1	1:I:277:LYS:HD3	2.27	0.49
2:O:40:VAL:HG23	2:O:62:GLY:H	1.77	0.49
2:T:2:ASN:OD1	2:T:3:ILE:N	2.45	0.49
1:A:128:VAL:HG13	1:A:501:ARG:HG3	1.93	0.49
1:A:431:GLY:N	1:A:437:ASN:OD1	2.44	0.49
1:B:399:ALA:O	1:B:403:THR:HG23	2.13	0.49
1:C:186:GLU:O	1:C:380:LYS:N	2.33	0.49
1:C:458:CYS:HB3	1:C:483:GLU:OE2	2.13	0.49
1:D:12:ALA:HA	1:D:520:MET:HE1	1.93	0.49
1:D:196:ASP:HA	1:D:329:THR:HA	1.94	0.49
1:D:438:VAL:O	1:D:442:VAL:HG23	2.12	0.49
1:J:265:ASN:O	1:J:269:GLY:N	2.45	0.49
1:L:37:ASN:ND2	1:L:51:LYS:HE2	2.27	0.49
1:N:100:ILE:HD11	1:N:510:VAL:HG22	1.95	0.49
1:N:177:VAL:HA	1:N:379:ILE:HB	1.93	0.49
1:N:284:ARG:HB3	1:N:284:ARG:CZ	2.41	0.49

Continued on next page...

For Manuscript Review

Continued from previous page...

Atom-1	Atom-2	Interatomic distance (Å)	Clash overlap (Å)
1:B:111:MET:HE1	1:B:116:LEU:HD21	1.93	0.49
1:B:175:ILE:HB	1:B:404:ARG:HH12	1.77	0.49
1:D:262:LEU:HD13	1:D:273:VAL:HG11	1.94	0.49
1:E:144:ILE:O	1:E:403:THR:HG22	2.12	0.49
1:E:409:GLU:OE2	1:E:501:ARG:NH2	2.44	0.49
1:H:356:ALA:HB2	1:H:365:LEU:HD12	1.94	0.49
1:I:325:ILE:HG13	1:I:325:ILE:O	2.12	0.49
1:I:349:ILE:HG12	1:I:368:ARG:NH2	2.28	0.49
1:K:432:GLN:HB2	1:K:436:GLN:NE2	2.27	0.49
1:K:460:GLU:HG3	1:K:478:TYR:OH	2.12	0.49
1:L:291:ASP:OD2	1:L:368:ARG:HD2	2.12	0.49
1:L:414:GLY:HA3	1:L:493:ILE:HG22	1.94	0.49
1:M:122:LYS:HZ2	1:M:440:ILE:HD11	1.76	0.49
1:N:124:VAL:HG13	1:N:504:LEU:HG	1.94	0.49
1:N:124:VAL:HG21	1:N:508:ALA:HB2	1.94	0.49
2:O:2:ASN:OD1	2:O:3:ILE:N	2.45	0.49
2:R:47:ARG:NH2	2:R:88:GLU:HB3	2.27	0.49
2:S:17:VAL:HG22	2:S:34:LYS:HA	1.95	0.49
2:U:20:LYS:HE3	2:U:24:GLY:HA2	1.93	0.49
1:A:33:PRO:HD3	3:A:601:ATP:C4	2.48	0.49
1:C:265:ASN:HA	1:C:268:ARG:HB2	1.95	0.49
1:D:220:ILE:HG13	1:D:248:LEU:HD23	1.95	0.49
1:E:194:GLN:HG3	1:E:331:THR:HG22	1.94	0.49
1:E:498:LYS:HG3	1:E:501:ARG:NH2	2.28	0.49
1:G:438:VAL:O	1:G:442:VAL:HG23	2.13	0.49
1:H:115:ASP:CG	1:H:118:ARG:HH21	2.16	0.49
1:H:218:PRO:HG3	1:H:323:VAL:HG22	1.94	0.49
1:I:204:PHE:CE2	1:I:275:ALA:HB3	2.47	0.49
1:J:186:GLU:O	1:J:380:LYS:N	2.26	0.49
1:J:224:ASP:OD2	1:J:286:LYS:HG2	2.12	0.49
1:K:219:PHE:HD2	1:K:240:VAL:HG22	1.76	0.49
1:K:349:ILE:HG12	1:K:368:ARG:CZ	2.42	0.49
1:K:356:ALA:HB3	1:K:362:ARG:NH2	2.26	0.49
1:L:174:VAL:HG11	1:L:376:VAL:HG12	1.93	0.49
1:L:323:VAL:HA	1:L:332:ILE:HA	1.94	0.49
1:M:230:ILE:O	1:M:234:LEU:N	2.45	0.49
1:B:225:LYS:N	1:B:252:GLU:OE1	2.46	0.49
1:B:411:VAL:HG21	1:B:494:LEU:HD22	1.94	0.49
1:C:143:ALA:O	1:C:147:VAL:HG23	2.12	0.49
1:C:498:LYS:HG3	1:C:501:ARG:NH2	2.27	0.49
1:D:498:LYS:HG3	1:D:501:ARG:NH2	2.28	0.49

Continued on next page...

For Manuscript Review

Continued from previous page...

Atom-1	Atom-2	Interatomic distance (Å)	Clash overlap (Å)
1:E:178:GLU:O	1:E:381:VAL:N	2.45	0.49
1:F:66:PHE:HB3	1:F:520:MET:SD	2.51	0.49
1:F:199:TYR:HE2	1:F:212:ALA:HA	1.75	0.49
1:G:111:MET:HE1	1:G:438:VAL:HB	1.95	0.49
1:H:433:ASN:OD1	1:H:434:GLU:N	2.46	0.49
1:I:31:LEU:HD13	1:I:90:THR:HB	1.94	0.49
1:L:352:GLN:HA	1:L:355:GLU:HG3	1.94	0.49
1:L:479:ASN:HB2	1:L:491:MET:HE3	1.93	0.49
1:M:262:LEU:O	1:M:266:THR:HG23	2.12	0.49
1:M:264:VAL:HA	1:M:267:MET:HE1	1.92	0.49
2:Q:11:ILE:HG13	2:Q:41:LEU:HD12	1.95	0.49
1:A:169:VAL:HG11	1:A:175:ILE:HG13	1.95	0.49
1:A:438:VAL:O	1:A:442:VAL:HG23	2.13	0.49
1:C:199:TYR:CD2	1:C:213:VAL:HG23	2.48	0.49
1:C:205:ILE:HD13	1:C:211:GLY:HA2	1.94	0.49
1:C:264:VAL:O	1:C:268:ARG:HG2	2.13	0.49
1:D:136:VAL:HG23	1:D:411:VAL:HG23	1.94	0.49
1:G:143:ALA:O	1:G:147:VAL:HG23	2.12	0.49
1:H:38:VAL:O	1:H:50:THR:N	2.42	0.49
1:H:224:ASP:OD2	1:H:286:LYS:HG2	2.13	0.49
1:M:76:GLU:HG2	1:M:80:LYS:HE3	1.93	0.49
1:M:148:GLY:HA2	1:M:399:ALA:HB1	1.95	0.49
1:M:345:ARG:NH2	1:M:368:ARG:HH12	2.11	0.49
1:M:353:ILE:CG2	1:M:362:ARG:HH22	2.23	0.49
2:P:86:MET:HG3	2:P:90:ASP:HB2	1.95	0.49
1:A:141:SER:HB2	1:A:163:ALA:HB1	1.95	0.49
1:B:136:VAL:HG23	1:B:411:VAL:HG23	1.95	0.49
1:B:199:TYR:HE2	1:B:212:ALA:HA	1.76	0.49
1:D:16:MET:SD	1:D:514:MET:HE1	2.52	0.49
1:E:287:ALA:HA	1:E:345:ARG:NH2	2.28	0.49
1:F:177:VAL:HG23	1:F:400:LEU:HD22	1.95	0.49
1:G:179:ASP:OD1	1:G:393:LYS:HD2	2.12	0.49
1:G:194:GLN:O	1:G:371:LYS:NZ	2.40	0.49
1:I:491:MET:HG2	1:I:493:ILE:HG13	1.93	0.49
1:J:37:ASN:ND2	1:J:51:LYS:HE2	2.27	0.49
1:J:206:ASN:ND2	1:J:214:GLU:O	2.45	0.49
1:K:130:GLU:OE1	1:K:426:LEU:HG	2.12	0.49
1:K:460:GLU:O	1:K:462:PRO:HD3	2.13	0.49
1:L:417:VAL:O	1:L:421:ARG:HG2	2.13	0.49
1:M:346:VAL:O	1:M:350:ARG:HG2	2.12	0.49
1:C:215:LEU:HB3	1:C:246:PRO:HB2	1.93	0.49

Continued on next page...

For Manuscript Review

Continued from previous page...

Atom-1	Atom-2	Interatomic distance (Å)	Clash overlap (Å)
1:C:386:GLU:O	1:C:389:MET:HB2	2.13	0.49
1:D:342:ILE:HG23	1:D:372:LEU:HB3	1.95	0.49
1:D:427:ALA:HA	1:D:444:LEU:HD13	1.94	0.49
1:E:283:ASP:OD1	1:E:284:ARG:N	2.46	0.49
1:E:291:ASP:OD1	1:E:372:LEU:HD22	2.11	0.49
1:F:154:SER:N	7:F:719:HOH:O	2.44	0.49
1:I:177:VAL:HG13	1:I:393:LYS:NZ	2.28	0.49
1:I:265:ASN:O	1:I:269:GLY:N	2.45	0.49
1:K:455:VAL:HG22	1:K:478:TYR:CE2	2.48	0.49
1:L:12:ALA:O	1:L:16:MET:HG2	2.11	0.49
1:L:32:GLY:HA3	1:L:454:ILE:HG23	1.95	0.49
1:M:171:LYS:HD2	1:M:407:VAL:HG22	1.93	0.49
1:M:468:THR:HB	1:M:485:TYR:CE2	2.48	0.49
1:N:16:MET:O	1:N:20:VAL:HG23	2.13	0.49
1:N:37:ASN:ND2	1:N:51:LYS:HE2	2.28	0.49
1:N:241:ALA:HB2	1:N:271:VAL:HG22	1.95	0.49
1:N:320:ALA:HA	1:N:336:VAL:H	1.78	0.49
1:B:421:ARG:HH12	1:B:470:LYS:HA	1.77	0.49
1:C:102:GLU:CB	1:C:442:VAL:HG13	2.43	0.49
1:D:41:ASP:OD1	1:D:47:PRO:HG3	2.13	0.49
1:E:179:ASP:OD1	1:E:393:LYS:HD2	2.13	0.49
1:F:116:LEU:HD21	1:F:438:VAL:HG12	1.95	0.49
1:F:209:GLU:HG2	1:F:210:THR:HG23	1.94	0.49
1:F:251:ALA:O	1:F:278:ALA:N	2.43	0.49
1:F:313:THR:O	1:F:317:LEU:HG	2.13	0.49
1:I:460:GLU:O	1:I:462:PRO:HD3	2.13	0.49
1:J:301:ILE:HG12	1:J:307:MET:HE1	1.94	0.49
1:J:477:GLY:N	1:J:486:GLY:O	2.43	0.49
1:K:262:LEU:HD22	1:K:273:VAL:HG11	1.95	0.49
1:N:440:ILE:O	1:N:444:LEU:HG	2.13	0.49
1:A:189:VAL:HA	1:A:377:ALA:HA	1.94	0.48
1:A:421:ARG:NH2	1:A:469:VAL:O	2.37	0.48
1:B:33:PRO:HD3	3:B:601:ATP:C4	2.48	0.48
1:B:349:ILE:HG23	1:B:365:LEU:HD12	1.95	0.48
1:E:364:LYS:O	1:E:368:ARG:HG3	2.12	0.48
1:G:283:ASP:OD1	1:G:284:ARG:N	2.46	0.48
1:G:458:CYS:HB3	1:G:483:GLU:OE2	2.13	0.48
1:I:284:ARG:HE	1:I:364:LYS:HB3	1.78	0.48
1:J:66:PHE:CD1	1:J:520:MET:HE3	2.47	0.48
1:J:122:LYS:HZ3	1:J:431:GLY:HA2	1.77	0.48
1:J:124:VAL:HG11	1:J:508:ALA:HB2	1.95	0.48

Continued on next page...

For Manuscript Review

Continued from previous page...

Atom-1	Atom-2	Interatomic distance (Å)	Clash overlap (Å)
1:J:218:PRO:HG3	1:J:323:VAL:HG22	1.95	0.48
1:K:202:PRO:HG2	1:L:384:ALA:HA	1.94	0.48
1:K:415:GLY:HA2	6:K:601:ADP:N3	2.28	0.48
1:L:311:LYS:HD2	1:L:311:LYS:O	2.13	0.48
1:N:498:LYS:HG3	1:N:501:ARG:HH21	1.77	0.48
2:T:94:ILE:HG13	2:U:6:LEU:HD11	1.95	0.48
1:A:213:VAL:HB	1:A:325:ILE:HB	1.95	0.48
1:B:201:SER:HB2	1:B:259:LEU:HD21	1.95	0.48
1:B:472:GLY:HA3	1:B:476:TYR:CD2	2.48	0.48
1:E:32:GLY:HA2	3:E:601:ATP:O4’	2.13	0.48
1:E:301:ILE:HD11	1:E:316:ASP:HB3	1.95	0.48
1:F:140:ASP:N	1:F:140:ASP:OD1	2.46	0.48
1:F:472:GLY:HA3	1:F:476:TYR:CD2	2.48	0.48
1:H:175:ILE:HA	1:H:377:ALA:HB3	1.94	0.48
1:H:204:PHE:HE2	1:H:275:ALA:HB3	1.77	0.48
1:I:311:LYS:HD2	1:I:311:LYS:O	2.14	0.48
1:I:323:VAL:HG12	1:I:332:ILE:HG22	1.93	0.48
1:I:468:THR:HB	1:I:485:TYR:CE2	2.48	0.48
1:M:220:ILE:HG12	1:M:222:LEU:HD21	1.94	0.48
1:M:262:LEU:HB3	1:M:273:VAL:HG11	1.94	0.48
1:M:477:GLY:O	1:M:486:GLY:N	2.46	0.48
1:A:102:GLU:HB2	1:A:442:VAL:HG13	1.95	0.48
1:A:199:TYR:CD2	1:A:213:VAL:HG23	2.48	0.48
1:C:219:PHE:CZ	1:C:245:LYS:HD2	2.48	0.48
1:C:261:THR:O	1:C:265:ASN:ND2	2.44	0.48
1:C:387:VAL:HA	1:C:390:LYS:HE2	1.94	0.48
1:D:320:ALA:HA	1:D:335:GLY:HA2	1.93	0.48
1:G:150:ILE:O	7:G:701:HOH:O	2.20	0.48
1:I:37:ASN:ND2	1:I:51:LYS:HE2	2.27	0.48
1:I:301:ILE:HG21	1:I:309:LEU:HD23	1.94	0.48
1:J:204:PHE:HE2	1:J:275:ALA:HB3	1.77	0.48
1:K:262:LEU:HD22	1:K:273:VAL:HG21	1.95	0.48
1:L:6:VAL:HG22	1:L:521:VAL:HG22	1.95	0.48
1:L:460:GLU:O	1:L:462:PRO:HD3	2.14	0.48
1:L:520:MET:HE2	1:M:39:VAL:HB	1.94	0.48
1:M:39:VAL:HG13	1:M:49:ILE:HG12	1.95	0.48
1:N:214:GLU:HG3	1:N:324:VAL:HG22	1.94	0.48
1:N:222:LEU:HD23	1:N:250:ILE:HB	1.96	0.48
1:N:383:ALA:HB1	1:N:388:GLU:HB3	1.95	0.48
1:N:472:GLY:HA3	1:N:476:TYR:CD2	2.48	0.48
2:S:65:VAL:HB	2:S:91:ILE:HG23	1.96	0.48

Continued on next page...

For Manuscript Review

Continued from previous page...

Atom-1	Atom-2	Interatomic distance (Å)	Clash overlap (Å)
1:A:134:LEU:HD23	1:A:418:ALA:HB1	1.95	0.48
1:A:287:ALA:HA	1:A:345:ARG:NH2	2.27	0.48
1:A:427:ALA:HA	1:A:444:LEU:HD13	1.95	0.48
1:A:448:GLU:OE1	1:A:470:LYS:NZ	2.42	0.48
1:B:166:MET:HB3	1:B:175:ILE:HD11	1.94	0.48
1:B:240:VAL:HG11	1:B:247:LEU:HB2	1.95	0.48
1:C:325:ILE:HG13	1:C:330:THR:HG23	1.94	0.48
1:E:206:ASN:HD21	1:E:214:GLU:HB3	1.78	0.48
1:G:323:VAL:HG22	1:G:332:ILE:HA	1.94	0.48
1:I:186:GLU:O	1:I:380:LYS:N	2.26	0.48
1:I:429:LEU:HG	1:I:440:ILE:HD13	1.93	0.48
1:J:36:ARG:NH2	1:J:456:LEU:O	2.34	0.48
1:J:299:THR:N	1:J:316:ASP:O	2.43	0.48
1:L:149:THR:OG1	1:L:156:GLU:HA	2.13	0.48
1:L:193:MET:HG2	1:L:295:LEU:HD13	1.94	0.48
1:M:36:ARG:HE	1:M:457:ASN:HA	1.79	0.48
2:O:11:ILE:HD12	2:O:42:ALA:HB3	1.94	0.48
2:P:64:ILE:O	2:P:95:VAL:N	2.38	0.48
2:T:26:VAL:HG12	2:T:28:THR:HG23	1.95	0.48
1:B:20:VAL:HA	1:B:74:VAL:HG11	1.95	0.48
1:B:346:VAL:HA	1:B:349:ILE:HD12	1.95	0.48
1:B:452:ARG:NH1	7:B:2009:HOH:O	2.30	0.48
1:C:247:LEU:HB3	1:C:273:VAL:HG22	1.95	0.48
1:D:246:PRO:HG3	1:D:272:LYS:HE2	1.93	0.48
1:E:231:ARG:HH22	2:S:27:LEU:HD13	1.79	0.48
1:F:151:SER:HB2	1:F:399:ALA:HA	1.96	0.48
1:F:352:GLN:HB3	1:F:365:LEU:HD13	1.96	0.48
1:G:61:GLU:HG2	1:G:72:GLN:OE1	2.13	0.48
1:H:325:ILE:HA	1:H:330:THR:HA	1.96	0.48
1:I:144:ILE:HD12	1:I:166:MET:HE3	1.94	0.48
1:J:25:ASP:OD1	1:J:97:GLN:NE2	2.45	0.48
1:K:165:ALA:HB2	1:K:187:LEU:HD22	1.96	0.48
1:K:166:MET:HB3	1:K:171:LYS:HA	1.96	0.48
1:K:381:VAL:HG21	1:K:393:LYS:HB2	1.96	0.48
1:L:40:LEU:HD13	1:L:59:GLU:HG3	1.95	0.48
1:L:107:VAL:HG13	1:L:113:PRO:HG3	1.96	0.48
1:L:231:ARG:O	1:L:231:ARG:NH1	2.39	0.48
1:N:13:ARG:HG2	1:N:514:MET:HE3	1.94	0.48
1:N:16:MET:HE3	1:N:69:MET:SD	2.53	0.48
1:N:479:ASN:HB2	1:N:491:MET:SD	2.54	0.48
2:U:65:VAL:HG23	2:U:67:PHE:HD1	1.78	0.48

Continued on next page...

For Manuscript Review

Continued from previous page...

Atom-1	Atom-2	Interatomic distance (Å)	Clash overlap (Å)
1:B:278:ALA:HB3	1:B:285:ARG:HH11	1.77	0.48
1:B:479:ASN:HB3	1:B:484:GLU:HG2	1.95	0.48
1:C:32:GLY:HA2	3:C:601:ATP:O4'	2.14	0.48
1:D:152:ALA:HB2	1:D:399:ALA:HB2	1.96	0.48
1:D:199:TYR:HE2	1:D:212:ALA:HA	1.78	0.48
1:G:166:MET:HA	1:G:169:VAL:HG12	1.94	0.48
1:G:189:VAL:HA	1:G:377:ALA:HA	1.96	0.48
1:I:440:ILE:O	1:I:444:LEU:HG	2.13	0.48
1:J:194:GLN:HG3	1:J:331:THR:HB	1.95	0.48
1:J:284:ARG:HB3	1:J:284:ARG:CZ	2.44	0.48
1:M:279:PRO:O	1:M:285:ARG:HA	2.13	0.48
1:N:82:ASN:O	1:N:86:GLY:N	2.31	0.48
2:Q:14:ARG:NH1	2:Q:69:ASP:OD1	2.46	0.48
1:B:148:GLY:HA2	1:B:399:ALA:HB1	1.96	0.48
1:C:356:ALA:HB1	1:C:361:ASP:HB2	1.95	0.48
1:E:222:LEU:O	1:E:301:ILE:N	2.25	0.48
1:E:427:ALA:O	1:E:441:LYS:NZ	2.46	0.48
1:F:392:LYS:O	1:F:396:VAL:HG23	2.14	0.48
1:H:187:LEU:HB3	1:H:379:ILE:HG12	1.95	0.48
1:J:342:ILE:O	1:J:346:VAL:HG23	2.13	0.48
1:K:221:LEU:HD23	1:K:249:ILE:HG23	1.96	0.48
1:L:5:ASP:N	1:L:522:THR:O	2.44	0.48
1:M:472:GLY:HA3	1:M:476:TYR:CD2	2.48	0.48
1:N:199:TYR:CE2	1:N:327:LYS:HA	2.49	0.48
1:N:311:LYS:HD2	1:N:311:LYS:O	2.13	0.48
1:N:421:ARG:NH1	1:N:469:VAL:O	2.46	0.48
1:N:495:ASP:OD2	6:N:601:ADP:O2'	2.23	0.48
1:A:100:ILE:HA	1:A:515:ILE:HD11	1.95	0.48
1:A:176:THR:O	1:A:379:ILE:N	2.33	0.48
1:A:287:ALA:HB1	1:A:368:ARG:CZ	2.44	0.48
1:B:114:MET:CE	1:C:34:LYS:HG2	2.44	0.48
1:D:230:ILE:O	1:D:233:MET:N	2.41	0.48
1:E:6:VAL:HG22	1:E:521:VAL:HG13	1.96	0.48
1:F:207:LYS:HE2	1:F:212:ALA:HB3	1.95	0.48
1:I:161:LEU:HG	1:I:187:LEU:HD23	1.96	0.48
1:I:194:GLN:HA	1:I:330:THR:O	2.14	0.48
1:I:323:VAL:HA	1:I:332:ILE:HA	1.95	0.48
1:I:359:ASP:O	1:I:362:ARG:HB2	2.14	0.48
1:I:433:ASN:OD1	1:I:434:GLU:N	2.47	0.48
1:K:195:PHE:CE2	1:K:197:ARG:HB2	2.49	0.48
1:K:204:PHE:HE2	1:K:275:ALA:HB3	1.78	0.48

Continued on next page...

For Manuscript Review

Continued from previous page...

Atom-1	Atom-2	Interatomic distance (Å)	Clash overlap (Å)
1:L:16:MET:O	1:L:20:VAL:HG23	2.14	0.48
1:M:301:ILE:HG12	1:M:307:MET:HE1	1.96	0.48
1:M:311:LYS:O	1:M:311:LYS:HD2	2.14	0.48
2:O:37:ARG:HH22	2:P:78:ILE:HG22	1.78	0.48
1:A:219:PHE:HB3	1:A:317:LEU:HD13	1.95	0.48
1:A:364:LYS:O	1:A:368:ARG:HG3	2.13	0.48
1:B:427:ALA:O	1:B:441:LYS:NZ	2.46	0.48
1:D:124:VAL:HG22	1:D:504:LEU:HD11	1.95	0.48
1:F:122:LYS:NZ	1:F:430:ARG:O	2.34	0.48
1:F:141:SER:HB2	1:F:163:ALA:HB1	1.96	0.48
1:F:144:ILE:HG23	1:F:403:THR:CG2	2.44	0.48
1:F:220:ILE:N	1:F:318:GLY:O	2.33	0.48
1:G:102:GLU:CB	1:G:442:VAL:HG13	2.44	0.48
1:H:124:VAL:HG13	1:H:504:LEU:HG	1.95	0.48
1:H:320:ALA:HA	1:H:336:VAL:H	1.79	0.48
1:J:284:ARG:HE	1:J:364:LYS:HB3	1.78	0.48
1:L:220:ILE:HG13	1:L:248:LEU:HD22	1.94	0.48
1:L:259:LEU:O	1:L:263:VAL:HG23	2.13	0.48
1:L:265:ASN:O	1:L:269:GLY:N	2.46	0.48
1:M:222:LEU:HD13	1:M:293:ALA:HA	1.96	0.48
2:P:40:VAL:HG23	2:P:62:GLY:N	2.29	0.48
1:A:415:GLY:HA2	3:A:601:ATP:H1'	1.96	0.48
1:A:475:ASN:HB2	1:A:487:ASN:ND2	2.29	0.48
1:B:226:LYS:HZ3	1:B:253:ASP:HB3	1.79	0.48
1:D:479:ASN:N	1:D:484:GLU:O	2.43	0.48
1:E:268:ARG:NH1	2:S:26:VAL:HG11	2.29	0.48
1:F:320:ALA:HA	1:F:335:GLY:HA2	1.95	0.48
1:F:430:ARG:HH12	1:F:441:LYS:HE2	1.79	0.48
1:I:477:GLY:O	1:I:486:GLY:N	2.47	0.48
1:L:69:MET:HE3	1:M:47:PRO:HD2	1.95	0.48
1:L:262:LEU:HD22	1:L:273:VAL:HG21	1.95	0.48
1:L:421:ARG:NH1	1:L:469:VAL:O	2.46	0.48
1:M:140:ASP:O	1:M:144:ILE:HG12	2.14	0.48
1:M:429:LEU:HG	1:M:440:ILE:HD13	1.95	0.48
2:P:12:VAL:CG2	2:P:84:LEU:HB2	2.44	0.48
2:S:5:PRO:HD3	2:S:42:ALA:HB1	1.96	0.48
2:S:67:PHE:CE2	2:S:69:ASP:HB3	2.49	0.48
1:A:472:GLY:HA3	1:A:476:TYR:CD2	2.48	0.47
1:B:214:GLU:HG3	1:B:324:VAL:HG22	1.95	0.47
1:D:128:VAL:HG13	1:D:501:ARG:HG3	1.96	0.47
1:E:31:LEU:HB2	1:E:90:THR:CG2	2.40	0.47

Continued on next page...

For Manuscript Review

Continued from previous page...

Atom-1	Atom-2	Interatomic distance (Å)	Clash overlap (Å)
1:E:33:PRO:HD3	3:E:601:ATP:C4	2.49	0.47
1:F:221:LEU:HD11	1:F:309:LEU:HD11	1.96	0.47
1:F:262:LEU:O	1:F:266:THR:HG23	2.14	0.47
1:F:422:VAL:HA	1:F:425:LYS:HE2	1.96	0.47
1:G:178:GLU:HA	1:G:393:LYS:HE2	1.95	0.47
1:G:399:ALA:O	1:G:403:THR:HG23	2.13	0.47
1:I:70:GLY:HA2	1:I:73:MET:HE1	1.95	0.47
1:J:419:LEU:HD22	1:J:447:MET:SD	2.54	0.47
1:K:324:VAL:O	1:K:331:THR:N	2.43	0.47
1:M:479:ASN:HB2	1:M:491:MET:SD	2.54	0.47
2:R:27:LEU:HB3	2:R:31:ALA:HB3	1.95	0.47
1:A:12:ALA:CA	1:A:520:MET:HE1	2.43	0.47
1:A:138:CYS:HB2	1:A:411:VAL:HG13	1.96	0.47
1:B:68:ASN:O	1:B:72:GLN:HG2	2.14	0.47
1:C:31:LEU:O	1:C:457:ASN:ND2	2.32	0.47
1:D:32:GLY:HA2	3:D:601:ATP:O4’	2.14	0.47
1:E:178:GLU:HA	1:E:393:LYS:HE2	1.95	0.47
1:F:134:LEU:HD23	1:F:418:ALA:HB1	1.95	0.47
1:G:206:ASN:HD21	1:G:214:GLU:HB3	1.79	0.47
3:G:601:ATP:H5’2	3:G:601:ATP:H8	1.79	0.47
1:H:319:GLN:HB2	1:H:336:VAL:HB	1.97	0.47
1:H:414:GLY:HA3	1:H:493:ILE:HG22	1.95	0.47
1:I:365:LEU:HA	1:I:368:ARG:HG3	1.95	0.47
1:J:458:CYS:SG	1:J:480:ALA:HB1	2.54	0.47
1:J:465:VAL:HA	1:J:485:TYR:OH	2.14	0.47
1:K:218:PRO:HG3	1:K:323:VAL:HG22	1.96	0.47
1:K:519:CYS:HB3	1:L:38:VAL:HG22	1.96	0.47
1:L:12:ALA:HB1	1:L:520:MET:SD	2.55	0.47
1:M:37:ASN:ND2	1:M:51:LYS:HE2	2.28	0.47
1:N:64:ASP:HB3	1:N:67:GLU:HB2	1.96	0.47
1:N:250:ILE:HG12	1:N:276:VAL:HB	1.94	0.47
2:R:65:VAL:HG23	2:R:67:PHE:HD1	1.79	0.47
2:S:74:LYS:O	2:S:85:ILE:N	2.46	0.47
1:A:11:ASP:O	1:A:15:LYS:HG2	2.13	0.47
1:A:458:CYS:HB3	1:A:483:GLU:OE2	2.14	0.47
1:D:146:GLN:HB2	1:D:494:LEU:HD12	1.97	0.47
1:D:513:LEU:HD11	1:E:388:GLU:HA	1.95	0.47
1:F:427:ALA:HA	1:F:444:LEU:HD13	1.97	0.47
1:G:32:GLY:HA2	3:G:601:ATP:O4’	2.14	0.47
1:H:165:ALA:HB2	1:H:187:LEU:HD22	1.96	0.47
1:J:65:LYS:O	1:J:69:MET:HB2	2.14	0.47

Continued on next page...

For Manuscript Review

Continued from previous page...

Atom-1	Atom-2	Interatomic distance (Å)	Clash overlap (Å)
1:J:440:ILE:O	1:J:444:LEU:HG	2.14	0.47
1:K:190:VAL:O	1:K:376:VAL:N	2.47	0.47
1:K:230:ILE:HD13	1:K:261:THR:HB	1.95	0.47
1:K:411:VAL:HG12	1:K:496:PRO:HA	1.97	0.47
1:L:194:GLN:HA	1:L:330:THR:O	2.14	0.47
1:M:14:VAL:O	1:M:18:ARG:HG3	2.12	0.47
1:M:323:VAL:HA	1:M:332:ILE:HA	1.97	0.47
1:M:324:VAL:HB	1:M:331:THR:HG23	1.96	0.47
1:N:292:ILE:HA	1:N:295:LEU:HD12	1.95	0.47
1:N:460:GLU:O	1:N:462:PRO:HD3	2.15	0.47
2:O:4:ARG:NH1	2:U:94:ILE:HD12	2.30	0.47
2:O:38:GLY:HA3	2:O:67:PHE:HE1	1.79	0.47
2:Q:59:VAL:HG11	2:Q:91:ILE:HG21	1.95	0.47
2:R:73:VAL:HA	2:R:86:MET:HB3	1.96	0.47
2:R:94:ILE:HG13	2:S:6:LEU:HD11	1.96	0.47
1:A:227:ILE:HG23	1:A:233:MET:SD	2.54	0.47
1:A:346:VAL:HA	1:A:349:ILE:HB	1.96	0.47
1:C:20:VAL:HA	1:C:74:VAL:HG11	1.96	0.47
1:C:430:ARG:HD2	1:C:437:ASN:HB3	1.96	0.47
1:D:231:ARG:NH2	2:R:21:SER:OG	2.47	0.47
1:F:20:VAL:HG12	1:F:97:GLN:OE1	2.15	0.47
1:F:22:VAL:HG11	1:F:62:LEU:HD21	1.95	0.47
1:F:69:MET:HB3	1:G:47:PRO:HG2	1.96	0.47
1:G:128:VAL:HG13	1:G:501:ARG:HG3	1.96	0.47
1:I:353:ILE:HG23	1:I:362:ARG:NH1	2.30	0.47
1:I:460:GLU:HG3	1:I:478:TYR:OH	2.14	0.47
1:K:417:VAL:O	1:K:421:ARG:HG2	2.14	0.47
1:L:148:GLY:CA	1:L:399:ALA:HB1	2.45	0.47
1:M:190:VAL:O	1:M:376:VAL:N	2.48	0.47
1:M:319:GLN:HB2	1:M:336:VAL:HB	1.96	0.47
1:M:339:GLU:HA	1:M:342:ILE:HD12	1.95	0.47
1:N:140:ASP:O	1:N:144:ILE:HG13	2.15	0.47
1:N:417:VAL:O	1:N:421:ARG:HG2	2.14	0.47
1:N:420:ILE:HG12	1:N:448:GLU:HG2	1.97	0.47
1:N:477:GLY:N	1:N:486:GLY:O	2.48	0.47
2:Q:12:VAL:CG2	2:Q:84:LEU:HB2	2.45	0.47
1:A:5:ASP:N	1:A:522:THR:O	2.29	0.47
1:A:264:VAL:HG11	2:O:28:THR:HG21	1.95	0.47
1:B:259:LEU:O	1:B:263:VAL:HG13	2.15	0.47
1:C:422:VAL:HA	1:C:425:LYS:HE2	1.97	0.47
1:C:430:ARG:HD3	1:C:430:ARG:HA	1.74	0.47

Continued on next page...

For Manuscript Review

Continued from previous page...

Atom-1	Atom-2	Interatomic distance (Å)	Clash overlap (Å)
1:E:479:ASN:N	1:E:484:GLU:O	2.47	0.47
1:F:451:LEU:HD21	1:F:469:VAL:HG21	1.96	0.47
1:G:262:LEU:O	1:G:266:THR:HG23	2.15	0.47
1:G:402:ALA:O	1:G:496:PRO:HG3	2.15	0.47
1:H:301:ILE:HG12	1:H:307:MET:HE1	1.95	0.47
1:H:438:VAL:O	1:H:442:VAL:HG23	2.15	0.47
1:I:301:ILE:HG12	1:I:307:MET:HE1	1.95	0.47
1:I:352:GLN:HA	1:I:355:GLU:HG3	1.95	0.47
1:I:353:ILE:HG23	1:I:362:ARG:HH12	1.79	0.47
1:J:339:GLU:HA	1:J:342:ILE:HD12	1.97	0.47
1:K:85:ALA:HB2	1:K:502:SER:HB2	1.95	0.47
1:K:124:VAL:HG11	1:K:508:ALA:HB2	1.95	0.47
1:L:76:GLU:HG2	1:L:80:LYS:HE3	1.95	0.47
1:L:204:PHE:CE2	1:L:275:ALA:HB3	2.49	0.47
1:L:455:VAL:HG21	1:L:465:VAL:HG11	1.96	0.47
1:M:290:GLN:OE1	1:M:294:THR:OG1	2.32	0.47
1:M:421:ARG:HH12	1:M:469:VAL:C	2.18	0.47
1:N:17:LEU:HD11	1:N:101:THR:HG23	1.97	0.47
1:N:95:LEU:O	1:N:99:ILE:HG13	2.13	0.47
2:T:47:ARG:N	2:T:55:LYS:O	2.47	0.47
1:A:152:ALA:HB2	1:A:399:ALA:HB2	1.97	0.47
1:A:165:ALA:HB2	1:A:379:ILE:HD11	1.97	0.47
1:A:353:ILE:HG23	1:A:362:ARG:HB2	1.95	0.47
1:B:12:ALA:HA	1:B:520:MET:HE2	1.96	0.47
1:B:12:ALA:HA	1:B:520:MET:HE3	1.96	0.47
1:B:455:VAL:HG13	1:B:460:GLU:HB2	1.97	0.47
1:C:112:ASN:ND2	1:C:115:ASP:OD2	2.42	0.47
1:C:487:ASN:O	1:C:491:MET:HG2	2.14	0.47
1:H:460:GLU:HG3	1:H:478:TYR:OH	2.15	0.47
1:I:115:ASP:OD2	1:I:433:ASN:ND2	2.38	0.47
1:J:12:ALA:HA	1:J:520:MET:CE	2.43	0.47
1:L:169:VAL:HG21	1:L:377:ALA:HB2	1.96	0.47
1:M:348:GLN:O	1:M:351:GLN:HG3	2.15	0.47
1:N:174:VAL:HG11	1:N:376:VAL:HG12	1.95	0.47
1:N:284:ARG:CZ	1:N:364:LYS:HD2	2.45	0.47
2:Q:13:LYS:HB2	2:Q:41:LEU:HD11	1.95	0.47
2:T:11:ILE:O	2:T:41:LEU:N	2.34	0.47
1:A:138:CYS:O	1:A:407:VAL:HA	2.14	0.47
1:A:215:LEU:HD12	1:A:248:LEU:HB2	1.96	0.47
1:A:259:LEU:O	1:A:263:VAL:HG13	2.15	0.47
1:A:386:GLU:O	1:A:390:LYS:HG3	2.15	0.47

Continued on next page...

For Manuscript Review

Continued from previous page...

Atom-1	Atom-2	Interatomic distance (Å)	Clash overlap (Å)
1:B:69:MET:HB2	1:C:47:PRO:CG	2.45	0.47
1:C:66:PHE:HB3	1:C:520:MET:SD	2.54	0.47
1:C:218:PRO:HG2	1:C:323:VAL:HG23	1.97	0.47
1:C:230:ILE:HG22	1:C:234:LEU:HD23	1.96	0.47
1:C:519:CYS:SG	1:C:520:MET:N	2.88	0.47
1:D:190:VAL:N	1:D:376:VAL:O	2.41	0.47
1:D:207:LYS:NZ	1:D:214:GLU:HB2	2.30	0.47
1:D:231:ARG:NH2	1:D:234:LEU:HD21	2.29	0.47
1:D:511:ALA:O	1:D:515:ILE:HG12	2.13	0.47
1:E:113:PRO:HB3	1:E:515:ILE:HG22	1.95	0.47
1:E:265:ASN:HA	1:E:268:ARG:HB2	1.97	0.47
1:G:20:VAL:HA	1:G:74:VAL:HG11	1.97	0.47
1:G:301:ILE:HD11	1:G:316:ASP:HB3	1.97	0.47
1:H:5:ASP:N	1:H:522:THR:O	2.45	0.47
1:H:95:LEU:O	1:H:99:ILE:HG13	2.14	0.47
1:H:195:PHE:CD2	1:H:279:PRO:HB3	2.49	0.47
1:H:429:LEU:O	1:H:441:LYS:NZ	2.34	0.47
1:H:479:ASN:O	1:H:483:GLU:N	2.47	0.47
1:I:350:ARG:O	1:I:354:GLU:HG2	2.15	0.47
1:I:411:VAL:HA	1:I:496:PRO:HA	1.96	0.47
1:J:76:GLU:HG2	1:J:80:LYS:HE3	1.97	0.47
1:J:368:ARG:O	1:J:372:LEU:HD23	2.15	0.47
1:J:433:ASN:OD1	1:J:434:GLU:N	2.48	0.47
1:K:115:ASP:CG	1:K:118:ARG:HH21	2.16	0.47
1:K:348:GLN:O	1:K:351:GLN:HG2	2.15	0.47
1:L:32:GLY:HA2	6:L:601:ADP:O4'	2.15	0.47
1:L:34:LYS:HE2	1:L:481:ALA:HA	1.96	0.47
1:L:138:CYS:O	1:L:407:VAL:HG22	2.15	0.47
1:M:45:GLY:O	1:M:47:PRO:HD3	2.15	0.47
1:M:475:ASN:HB3	1:M:489:ILE:HG12	1.96	0.47
1:N:13:ARG:HA	1:N:514:MET:HE1	1.97	0.47
1:N:27:VAL:HG12	1:N:90:THR:HG23	1.97	0.47
1:N:141:SER:HA	1:N:144:ILE:HD12	1.95	0.47
1:N:168:LYS:HG2	1:N:189:VAL:HG13	1.96	0.47
1:N:219:PHE:HD2	1:N:240:VAL:HG22	1.80	0.47
2:P:92:LEU:HD21	2:Q:74:LYS:HG3	1.96	0.47
2:Q:64:ILE:O	2:Q:95:VAL:N	2.40	0.47
1:E:113:PRO:HA	1:E:116:LEU:HD12	1.96	0.47
1:E:392:LYS:O	1:E:396:VAL:HG23	2.14	0.47
1:H:12:ALA:O	1:H:16:MET:HG2	2.15	0.47
1:H:115:ASP:HB3	1:H:435:ASP:HB2	1.97	0.47

Continued on next page...

For Manuscript Review

Continued from previous page...

Atom-1	Atom-2	Interatomic distance (Å)	Clash overlap (Å)
1:H:460:GLU:O	1:H:462:PRO:HD3	2.13	0.47
1:I:137:PRO:HA	1:I:410:GLY:HA2	1.96	0.47
1:I:465:VAL:HA	1:I:485:TYR:OH	2.14	0.47
1:J:438:VAL:O	1:J:442:VAL:HG23	2.15	0.47
1:K:177:VAL:HG13	1:K:393:LYS:HE2	1.97	0.47
1:N:123:ALA:HB2	1:N:440:ILE:HG12	1.96	0.47
1:N:248:LEU:HD21	1:N:250:ILE:HD11	1.97	0.47
1:A:7:LYS:HG2	1:A:66:PHE:CE1	2.49	0.47
1:A:40:LEU:HD13	1:A:59:GLU:HG3	1.97	0.47
1:A:427:ALA:O	1:A:441:LYS:NZ	2.48	0.47
1:C:166:MET:HB3	1:C:175:ILE:HD11	1.96	0.47
1:C:270:ILE:HG21	2:Q:25:ILE:HA	1.97	0.47
1:C:427:ALA:HA	1:C:444:LEU:HD13	1.97	0.47
1:C:447:MET:HE2	1:C:447:MET:HA	1.97	0.47
1:D:15:LYS:HD2	1:D:67:GLU:HG3	1.97	0.47
1:D:153:ASN:ND2	7:D:2003:HOH:O	2.46	0.47
1:D:222:LEU:HD21	1:D:292:ILE:HG22	1.96	0.47
1:D:261:THR:HG21	2:R:27:LEU:HD13	1.95	0.47
1:D:361:ASP:O	1:D:365:LEU:HD23	2.15	0.47
1:F:346:VAL:HB	1:F:369:VAL:HG13	1.97	0.47
1:G:361:ASP:O	1:G:365:LEU:HD23	2.15	0.47
1:H:417:VAL:O	1:H:421:ARG:HG2	2.15	0.47
1:K:132:LYS:NZ	1:K:409:GLU:OE2	2.46	0.47
1:K:479:ASN:CG	1:K:493:ILE:HD11	2.35	0.47
1:L:345:ARG:HD2	1:L:348:GLN:OE1	2.14	0.47
1:M:351:GLN:NE2	1:M:352:GLN:HG3	2.30	0.47
2:P:38:GLY:HA3	2:P:67:PHE:HE1	1.80	0.47
2:T:12:VAL:HG12	2:T:40:VAL:HG12	1.97	0.47
1:A:140:ASP:N	1:A:140:ASP:OD1	2.48	0.47
1:B:128:VAL:HG13	1:B:501:ARG:HG3	1.96	0.47
1:B:431:GLY:N	1:B:437:ASN:OD1	2.48	0.47
1:C:69:MET:HG3	1:D:47:PRO:HG2	1.97	0.47
1:D:217:SER:HA	1:D:320:ALA:O	2.15	0.47
1:D:392:LYS:O	1:D:396:VAL:HG23	2.15	0.47
1:E:339:GLU:O	1:E:342:ILE:HB	2.15	0.47
1:G:114:MET:HG3	1:G:118:ARG:NH1	2.30	0.47
1:G:247:LEU:HD21	1:G:249:ILE:HG13	1.96	0.47
1:H:284:ARG:CZ	1:H:364:LYS:HB3	2.45	0.47
1:H:333:ILE:HG23	1:H:376:VAL:HG21	1.96	0.47
1:H:349:ILE:HG12	1:H:368:ARG:CZ	2.45	0.47
1:I:289:LEU:HG	1:I:300:VAL:HG22	1.97	0.47

Continued on next page...

For Manuscript Review

Continued from previous page...

Atom-1	Atom-2	Interatomic distance (Å)	Clash overlap (Å)
1:I:351:GLN:NE2	1:I:352:GLN:HG3	2.29	0.47
1:J:195:PHE:CZ	1:J:250:ILE:HD13	2.50	0.47
1:L:295:LEU:HD21	1:L:372:LEU:HD13	1.96	0.47
1:M:460:GLU:O	1:M:462:PRO:HD3	2.15	0.47
2:Q:11:ILE:HB	2:Q:85:ILE:HD13	1.96	0.47
2:R:59:VAL:HG11	2:R:91:ILE:HG21	1.97	0.47
1:D:227:ILE:HB	1:D:254:VAL:HA	1.98	0.46
1:G:199:TYR:HE2	1:G:212:ALA:HA	1.79	0.46
1:G:392:LYS:O	1:G:396:VAL:HG23	2.14	0.46
1:G:427:ALA:O	1:G:441:LYS:NZ	2.47	0.46
1:H:195:PHE:HE2	1:H:197:ARG:HB2	1.80	0.46
1:H:346:VAL:O	1:H:350:ARG:HG2	2.15	0.46
1:I:339:GLU:HA	1:I:342:ILE:HB	1.96	0.46
1:K:95:LEU:O	1:K:99:ILE:HG13	2.15	0.46
1:K:158:VAL:HG13	1:K:396:VAL:HG22	1.97	0.46
1:M:444:LEU:O	1:M:447:MET:HG2	2.15	0.46
1:N:284:ARG:NE	1:N:364:LYS:HB3	2.29	0.46
1:A:420:ILE:CG2	1:A:470:LYS:HG2	2.45	0.46
1:B:222:LEU:O	1:B:301:ILE:N	2.29	0.46
1:C:136:VAL:HG23	1:C:411:VAL:HG23	1.97	0.46
1:C:243:ALA:HB2	1:C:314:LEU:HD21	1.97	0.46
1:C:294:THR:HG22	1:C:341:ALA:HB1	1.97	0.46
1:D:230:ILE:HG22	1:D:234:LEU:HD22	1.97	0.46
1:E:219:PHE:HB3	1:E:317:LEU:HD13	1.97	0.46
1:F:92:ALA:HB2	1:F:503:ALA:HB1	1.98	0.46
1:F:498:LYS:HG3	1:F:501:ARG:NH2	2.31	0.46
1:H:348:GLN:O	1:H:351:GLN:NE2	2.48	0.46
1:H:511:ALA:O	1:H:515:ILE:HG13	2.16	0.46
1:J:7:LYS:HE3	1:J:15:LYS:HE3	1.96	0.46
1:J:100:ILE:HG23	1:J:514:MET:HE1	1.97	0.46
1:J:137:PRO:HA	1:J:410:GLY:HA2	1.97	0.46
1:J:174:VAL:HG11	1:J:376:VAL:HG12	1.97	0.46
1:J:262:LEU:HD22	1:J:273:VAL:HG11	1.98	0.46
1:L:39:VAL:HA	1:L:49:ILE:HA	1.96	0.46
1:L:45:GLY:O	1:L:47:PRO:HD3	2.16	0.46
1:L:158:VAL:HG13	1:L:396:VAL:HA	1.97	0.46
1:M:250:ILE:HG23	1:M:278:ALA:HA	1.97	0.46
1:M:279:PRO:C	1:M:288:MET:HG3	2.36	0.46
1:N:221:LEU:HD23	1:N:249:ILE:HD12	1.96	0.46
2:P:25:ILE:H	2:P:25:ILE:HD12	1.80	0.46
2:T:37:ARG:HH22	2:U:78:ILE:HG22	1.80	0.46

Continued on next page...

For Manuscript Review

Continued from previous page...

Atom-1	Atom-2	Interatomic distance (Å)	Clash overlap (Å)
1:A:292:ILE:O	1:A:296:THR:OG1	2.22	0.46
1:B:227:ILE:HG23	1:B:233:MET:SD	2.56	0.46
1:C:359:ASP:HA	1:C:362:ARG:HE	1.80	0.46
1:D:178:GLU:N	1:D:379:ILE:O	2.41	0.46
1:D:387:VAL:HA	1:D:390:LYS:HE2	1.96	0.46
1:F:149:THR:HA	1:F:155:ASP:O	2.16	0.46
1:F:215:LEU:HD12	1:F:248:LEU:HB2	1.97	0.46
1:G:205:ILE:HD13	1:G:211:GLY:HA2	1.96	0.46
1:H:195:PHE:CZ	1:H:250:ILE:HD13	2.48	0.46
1:H:197:ARG:HD2	1:H:277:LYS:HB3	1.96	0.46
1:J:489:ILE:HA	1:J:494:LEU:HD21	1.97	0.46
1:K:32:GLY:HA2	6:K:601:ADP:O4'	2.15	0.46
1:K:224:ASP:OD2	1:K:286:LYS:HG2	2.16	0.46
1:K:339:GLU:HA	1:K:342:ILE:HD12	1.97	0.46
1:L:414:GLY:O	1:L:417:VAL:HG22	2.15	0.46
1:M:230:ILE:O	1:M:234:LEU:HG	2.15	0.46
1:N:195:PHE:CE2	1:N:197:ARG:HB2	2.50	0.46
1:N:287:ALA:HB1	1:N:368:ARG:NH1	2.31	0.46
1:A:136:VAL:HG23	1:A:411:VAL:HG23	1.97	0.46
1:A:186:GLU:HG2	1:A:380:LYS:HB2	1.96	0.46
1:A:190:VAL:N	1:A:376:VAL:O	2.32	0.46
1:A:237:LEU:HD12	1:A:271:VAL:HG21	1.96	0.46
1:B:143:ALA:O	1:B:147:VAL:HG23	2.16	0.46
1:B:214:GLU:HG2	1:B:322:ARG:HH11	1.81	0.46
1:B:370:ALA:HB1	1:B:375:GLY:O	2.15	0.46
1:D:223:ALA:HB1	1:D:225:LYS:HG2	1.98	0.46
1:F:150:ILE:HG23	3:F:601:ATP:C8	2.50	0.46
1:F:356:ALA:HB1	1:F:361:ASP:HB2	1.97	0.46
1:G:200:LEU:HD12	1:G:275:ALA:HB1	1.97	0.46
1:H:220:ILE:HG21	1:H:296:THR:HG21	1.96	0.46
1:I:223:ALA:O	1:I:251:ALA:HA	2.16	0.46
1:J:419:LEU:HD12	1:J:450:PRO:HG2	1.96	0.46
1:L:15:LYS:HB3	1:L:66:PHE:HB2	1.97	0.46
1:L:230:ILE:O	1:L:234:LEU:N	2.49	0.46
1:L:333:ILE:HG12	1:L:376:VAL:HG11	1.96	0.46
1:M:455:VAL:CG1	1:M:460:GLU:HB2	2.45	0.46
1:N:185:ASP:OD2	1:N:392:LYS:HE3	2.15	0.46
1:N:342:ILE:O	1:N:346:VAL:HG23	2.16	0.46
1:N:433:ASN:OD1	1:N:434:GLU:N	2.48	0.46
2:O:57:LEU:O	2:O:60:LYS:NZ	2.32	0.46
2:P:37:ARG:NH2	2:Q:77:LYS:O	2.48	0.46

Continued on next page...

For Manuscript Review

Continued from previous page...

Atom-1	Atom-2	Interatomic distance (Å)	Clash overlap (Å)
2:S:17:VAL:HG21	2:S:34:LYS:HD2	1.98	0.46
1:A:6:VAL:HG22	1:A:521:VAL:HG13	1.98	0.46
1:A:32:GLY:HA2	3:A:601:ATP:O4'	2.15	0.46
1:A:102:GLU:OE1	1:A:445:ARG:NE	2.41	0.46
1:A:495:ASP:OD2	3:A:601:ATP:O2'	2.24	0.46
1:B:28:LYS:HE2	1:B:94:VAL:HG22	1.98	0.46
1:B:31:LEU:HD13	1:B:90:THR:HB	1.97	0.46
1:F:200:LEU:N	1:F:275:ALA:O	2.40	0.46
1:F:206:ASN:HD21	1:F:214:GLU:HB3	1.81	0.46
1:F:413:ALA:HB3	1:F:418:ALA:HB2	1.97	0.46
1:G:420:ILE:CG2	1:G:470:LYS:HG2	2.46	0.46
1:G:498:LYS:HG3	1:G:501:ARG:NH2	2.30	0.46
1:I:149:THR:OG1	1:I:156:GLU:HA	2.14	0.46
1:K:262:LEU:HB3	1:K:273:VAL:HG11	1.97	0.46
1:L:339:GLU:HG2	1:L:342:ILE:HD12	1.98	0.46
1:M:352:GLN:HA	1:M:355:GLU:CG	2.45	0.46
2:O:78:ILE:HA	2:U:37:ARG:HH22	1.81	0.46
2:S:9:ARG:HA	2:S:87:SER:HA	1.97	0.46
2:S:12:VAL:HG22	2:S:84:LEU:HB2	1.96	0.46
1:A:131:LEU:HD21	1:A:419:LEU:HD23	1.98	0.46
1:A:314:LEU:HA	1:A:317:LEU:HD12	1.97	0.46
1:B:7:LYS:HE2	1:B:11:ASP:HB3	1.98	0.46
1:B:287:ALA:HA	1:B:345:ARG:NH2	2.31	0.46
1:C:62:LEU:HB2	1:C:68:ASN:HB2	1.97	0.46
1:C:350:ARG:O	1:C:354:GLU:HG2	2.15	0.46
1:C:475:ASN:CG	1:C:489:ILE:HG12	2.36	0.46
1:E:77:VAL:HG12	1:E:92:ALA:HB1	1.97	0.46
1:F:206:ASN:ND2	1:F:214:GLU:O	2.48	0.46
1:F:493:ILE:HD13	3:F:601:ATP:N1	2.31	0.46
1:H:124:VAL:HG11	1:H:508:ALA:HB2	1.98	0.46
1:H:326:ASN:ND2	1:H:329:THR:OG1	2.43	0.46
1:I:95:LEU:O	1:I:99:ILE:HG13	2.15	0.46
1:I:287:ALA:HB1	1:I:368:ARG:CZ	2.46	0.46
1:I:339:GLU:HA	1:I:342:ILE:HD12	1.98	0.46
1:J:115:ASP:O	1:J:436:GLN:HG2	2.16	0.46
1:J:124:VAL:HG11	1:J:508:ALA:CB	2.46	0.46
1:J:274:ALA:HB1	1:J:325:ILE:CD1	2.45	0.46
1:K:115:ASP:O	1:K:436:GLN:HG2	2.16	0.46
1:M:16:MET:HE1	1:M:69:MET:SD	2.55	0.46
1:M:69:MET:HA	1:M:72:GLN:HG2	1.97	0.46
2:T:46:GLY:HA2	2:T:57:LEU:CD1	2.45	0.46

Continued on next page...

For Manuscript Review

Continued from previous page...

Atom-1	Atom-2	Interatomic distance (Å)	Clash overlap (Å)
1:B:453:GLN:NE2	1:B:457:ASN:OD1	2.48	0.46
1:C:77:VAL:HG11	1:C:96:ALA:HB2	1.97	0.46
1:C:152:ALA:HA	1:C:395:ARG:HG2	1.98	0.46
1:H:174:VAL:HB	1:H:376:VAL:HA	1.97	0.46
1:H:274:ALA:HB1	1:H:325:ILE:CD1	2.46	0.46
1:H:325:ILE:O	1:H:325:ILE:HG13	2.15	0.46
1:I:417:VAL:O	1:I:421:ARG:HG2	2.15	0.46
1:K:7:LYS:HD3	1:K:12:ALA:HA	1.97	0.46
1:K:130:GLU:HB3	1:K:422:VAL:HG22	1.98	0.46
1:N:262:LEU:HD22	1:N:273:VAL:HG11	1.97	0.46
2:O:67:PHE:HB3	2:O:91:ILE:HD13	1.97	0.46
1:A:217:SER:HA	1:A:320:ALA:O	2.16	0.46
1:B:346:VAL:HA	1:B:349:ILE:HB	1.98	0.46
1:D:370:ALA:HB1	1:D:375:GLY:O	2.16	0.46
1:H:15:LYS:NZ	1:H:64:ASP:OD2	2.33	0.46
1:H:64:ASP:HB3	1:H:67:GLU:HB2	1.98	0.46
1:H:227:ILE:HG12	1:H:309:LEU:HD11	1.96	0.46
1:J:230:ILE:O	1:J:234:LEU:N	2.49	0.46
1:J:295:LEU:HA	1:J:342:ILE:HG12	1.97	0.46
1:K:5:ASP:N	1:K:522:THR:O	2.40	0.46
1:K:19:GLY:HA3	1:K:67:GLU:O	2.16	0.46
1:K:42:LYS:HD3	1:K:46:ALA:O	2.15	0.46
1:L:115:ASP:HB3	1:L:435:ASP:HB2	1.97	0.46
1:M:195:PHE:CD2	1:M:279:PRO:HB3	2.51	0.46
1:N:475:ASN:HB2	1:N:487:ASN:ND2	2.31	0.46
2:Q:14:ARG:HG3	2:Q:67:PHE:HZ	1.81	0.46
1:C:204:PHE:CD1	1:C:266:THR:HG21	2.50	0.46
1:C:262:LEU:HD22	1:C:273:VAL:HG21	1.97	0.46
1:D:261:THR:HG23	2:R:27:LEU:HA	1.96	0.46
1:D:523:ASP:OD1	1:D:524:LEU:N	2.48	0.46
1:E:231:ARG:HH11	1:E:234:LEU:HD11	1.81	0.46
1:E:294:THR:HG22	1:E:341:ALA:HB1	1.97	0.46
1:E:411:VAL:HG21	1:E:494:LEU:HD22	1.98	0.46
1:F:162:ILE:HG12	1:F:400:LEU:HD13	1.97	0.46
1:J:19:GLY:HA3	1:J:67:GLU:O	2.16	0.46
1:J:166:MET:HA	1:J:175:ILE:HD11	1.98	0.46
1:J:252:GLU:O	1:J:277:LYS:HG3	2.16	0.46
1:J:411:VAL:HG12	1:J:496:PRO:HA	1.98	0.46
1:J:432:GLN:HB2	1:J:436:GLN:NE2	2.30	0.46
1:K:279:PRO:O	1:K:285:ARG:HA	2.15	0.46
1:L:31:LEU:HD13	1:L:90:THR:HB	1.98	0.46

Continued on next page...

For Manuscript Review

Continued from previous page...

Atom-1	Atom-2	Interatomic distance (Å)	Clash overlap (Å)
1:M:166:MET:O	1:M:169:VAL:N	2.49	0.46
2:O:25:ILE:H	2:O:25:ILE:HD12	1.81	0.46
2:T:11:ILE:HB	2:T:42:ALA:HB3	1.98	0.46
1:A:465:VAL:HA	1:A:485:TYR:OH	2.16	0.46
1:B:392:LYS:O	1:B:396:VAL:HG23	2.16	0.46
1:C:250:ILE:HG23	1:C:278:ALA:HA	1.98	0.46
1:C:345:ARG:O	1:C:349:ILE:HG13	2.15	0.46
1:D:169:VAL:HG13	1:D:170:GLY:O	2.16	0.46
1:E:11:ASP:O	1:E:15:LYS:HG2	2.16	0.46
1:E:259:LEU:O	1:E:263:VAL:HG13	2.16	0.46
1:F:15:LYS:HD3	1:F:18:ARG:HH21	1.80	0.46
1:G:204:PHE:CD1	1:G:266:THR:HG21	2.51	0.46
1:I:76:GLU:HG2	1:I:80:LYS:HE2	1.97	0.46
1:I:421:ARG:NH1	1:I:469:VAL:O	2.47	0.46
1:N:419:LEU:HD12	1:N:450:PRO:HG2	1.98	0.46
2:O:68:ASN:N	2:O:90:ASP:O	2.34	0.46
2:O:76:GLU:OE1	2:O:85:ILE:HG22	2.16	0.46
2:R:8:ASP:O	2:R:57:LEU:HD21	2.16	0.46
2:U:11:ILE:HB	2:U:42:ALA:H	1.80	0.46
1:A:313:THR:O	1:A:317:LEU:HG	2.16	0.45
1:A:498:LYS:HG3	1:A:501:ARG:NH2	2.31	0.45
1:B:219:PHE:CD2	1:B:240:VAL:HG22	2.50	0.45
1:C:15:LYS:HB2	1:C:520:MET:HE3	1.98	0.45
1:C:455:VAL:HG13	1:C:460:GLU:HB2	1.99	0.45
1:D:124:VAL:HG13	1:D:504:LEU:HG	1.98	0.45
1:D:165:ALA:O	1:D:169:VAL:HG12	2.15	0.45
1:D:465:VAL:HA	1:D:485:TYR:OH	2.16	0.45
1:E:193:MET:HE1	1:E:371:LYS:O	2.15	0.45
1:E:209:GLU:HG2	1:E:210:THR:HG23	1.98	0.45
1:F:126:ALA:O	1:F:130:GLU:HG2	2.16	0.45
1:F:259:LEU:O	1:F:263:VAL:HG13	2.15	0.45
1:G:339:GLU:O	1:G:342:ILE:HB	2.16	0.45
1:H:221:LEU:HB3	1:H:249:ILE:HA	1.98	0.45
1:H:458:CYS:SG	1:H:480:ALA:HB1	2.56	0.45
1:I:12:ALA:O	1:I:16:MET:HG2	2.16	0.45
1:I:115:ASP:CG	1:I:118:ARG:HH21	2.20	0.45
1:I:488:MET:HE3	1:I:493:ILE:HD12	1.98	0.45
1:K:475:ASN:HB2	1:K:487:ASN:ND2	2.31	0.45
1:K:479:ASN:HB2	1:K:491:MET:HE3	1.97	0.45
1:L:190:VAL:O	1:L:376:VAL:N	2.49	0.45
1:M:222:LEU:HD23	1:M:250:ILE:HB	1.98	0.45

Continued on next page...

For Manuscript Review

Continued from previous page...

Atom-1	Atom-2	Interatomic distance (Å)	Clash overlap (Å)
1:N:107:VAL:HG13	1:N:113:PRO:HG3	1.98	0.45
1:N:420:ILE:HD12	1:N:451:LEU:HD13	1.96	0.45
1:A:381:VAL:HG21	1:A:393:LYS:HA	1.98	0.45
1:C:287:ALA:HB1	1:C:368:ARG:CZ	2.46	0.45
1:C:453:GLN:NE2	1:C:457:ASN:OD1	2.49	0.45
1:D:69:MET:HB2	1:E:47:PRO:HG3	1.97	0.45
1:E:352:GLN:O	1:E:356:ALA:N	2.49	0.45
1:E:356:ALA:HB1	1:E:361:ASP:HB2	1.98	0.45
1:F:124:VAL:HG13	1:F:504:LEU:HG	1.97	0.45
1:F:179:ASP:OD1	1:F:393:LYS:HD2	2.15	0.45
1:G:51:LYS:HD3	1:G:153:ASN:ND2	2.31	0.45
1:G:207:LYS:HE2	1:G:212:ALA:HB3	1.98	0.45
1:G:523:ASP:OD1	1:G:524:LEU:N	2.48	0.45
1:H:123:ALA:HB3	1:H:443:ALA:HB3	1.98	0.45
1:H:141:SER:HA	1:H:144:ILE:HD13	1.97	0.45
1:K:195:PHE:HE2	1:K:197:ARG:HB2	1.81	0.45
1:K:438:VAL:O	1:K:442:VAL:HG23	2.15	0.45
1:L:498:LYS:HG3	1:L:501:ARG:NH2	2.31	0.45
1:M:40:LEU:N	1:M:48:THR:O	2.49	0.45
1:M:193:MET:SD	1:M:295:LEU:HD22	2.57	0.45
1:N:195:PHE:HE2	1:N:197:ARG:HB2	1.81	0.45
2:S:57:LEU:HD23	2:S:88:GLU:HB2	1.97	0.45
2:U:10:VAL:N	2:U:86:MET:O	2.23	0.45
2:U:25:ILE:HD12	2:U:25:ILE:H	1.81	0.45
1:B:204:PHE:CD1	1:B:266:THR:HG21	2.51	0.45
1:B:270:ILE:HG21	2:P:25:ILE:HA	1.99	0.45
1:D:287:ALA:HA	1:D:345:ARG:NH2	2.31	0.45
1:E:465:VAL:HA	1:E:485:TYR:OH	2.15	0.45
1:F:230:ILE:O	1:F:233:MET:N	2.39	0.45
1:F:386:GLU:O	1:F:390:LYS:HD3	2.15	0.45
1:G:205:ILE:HG23	1:G:212:ALA:O	2.16	0.45
1:G:223:ALA:HB1	1:G:225:LYS:HG2	1.98	0.45
1:H:149:THR:OG1	1:H:156:GLU:HA	2.16	0.45
1:H:206:ASN:ND2	1:H:214:GLU:O	2.49	0.45
1:H:381:VAL:HG11	1:H:393:LYS:HA	1.98	0.45
1:I:76:GLU:O	1:I:80:LYS:HG3	2.17	0.45
1:J:102:GLU:HB2	1:J:442:VAL:HG13	1.98	0.45
1:K:116:LEU:HG	1:K:435:ASP:HB3	1.97	0.45
1:M:76:GLU:O	1:M:80:LYS:HG3	2.15	0.45
1:N:195:PHE:CD2	1:N:279:PRO:HB3	2.51	0.45
2:O:37:ARG:HG2	2:O:66:ILE:HG12	1.98	0.45

Continued on next page...

For Manuscript Review

Continued from previous page...

Atom-1	Atom-2	Interatomic distance (Å)	Clash overlap (Å)
2:P:75:SER:HA	2:P:83:VAL:O	2.17	0.45
2:Q:2:ASN:OD1	2:Q:3:ILE:N	2.50	0.45
1:B:169:VAL:HG13	1:B:170:GLY:O	2.16	0.45
1:B:226:LYS:NZ	1:B:253:ASP:HB3	2.31	0.45
1:B:429:LEU:HB3	1:B:440:ILE:HG21	1.99	0.45
1:C:130:GLU:HB2	1:C:422:VAL:HG13	1.98	0.45
1:D:69:MET:HB2	1:E:47:PRO:CG	2.46	0.45
1:E:122:LYS:NZ	1:E:430:ARG:O	2.37	0.45
1:E:346:VAL:HB	1:E:369:VAL:HG22	1.99	0.45
1:F:414:GLY:H	1:F:488:MET:HB3	1.81	0.45
3:F:601:ATP:H5'2	3:F:601:ATP:H8	1.81	0.45
1:G:291:ASP:HB3	1:G:372:LEU:HD13	1.98	0.45
1:H:246:PRO:HA	1:H:272:LYS:HB2	1.98	0.45
1:I:224:ASP:OD2	1:I:286:LYS:HG2	2.16	0.45
1:J:13:ARG:HB3	1:J:104:LEU:HD22	1.98	0.45
1:J:149:THR:OG1	1:J:156:GLU:HA	2.15	0.45
1:K:386:GLU:HA	1:K:389:MET:HG2	1.98	0.45
1:L:381:VAL:HG11	1:L:396:VAL:HG21	1.99	0.45
1:M:356:ALA:HB2	1:M:365:LEU:HD12	1.97	0.45
1:M:460:GLU:HG3	1:M:478:TYR:OH	2.17	0.45
1:N:199:TYR:CZ	1:N:327:LYS:HA	2.52	0.45
2:P:73:VAL:HA	2:P:86:MET:HB3	1.98	0.45
1:A:226:LYS:HE3	1:A:255:GLU:HG2	1.97	0.45
1:A:361:ASP:O	1:A:365:LEU:HD23	2.16	0.45
1:B:217:SER:HA	1:B:320:ALA:O	2.17	0.45
1:B:475:ASN:HB2	1:B:487:ASN:ND2	2.32	0.45
1:C:5:ASP:N	1:C:522:THR:O	2.29	0.45
1:C:259:LEU:O	1:C:263:VAL:HG13	2.17	0.45
1:F:353:ILE:HD13	1:F:366:GLN:HG3	1.98	0.45
1:F:430:ARG:HH22	1:F:441:LYS:HE2	1.81	0.45
1:G:321:LYS:HB3	1:G:334:ASP:HB3	1.98	0.45
1:G:479:ASN:HB2	1:G:491:MET:HE3	1.97	0.45
1:I:520:MET:HG3	1:J:39:VAL:O	2.16	0.45
1:J:262:LEU:HB3	1:J:273:VAL:HG11	1.98	0.45
1:J:349:ILE:O	1:J:353:ILE:HG13	2.16	0.45
1:J:417:VAL:O	1:J:421:ARG:HG2	2.16	0.45
1:K:417:VAL:HG12	1:K:451:LEU:HD12	1.98	0.45
1:L:149:THR:HG22	1:L:154:SER:HA	1.97	0.45
1:N:111:MET:HB2	1:N:116:LEU:HD11	1.98	0.45
1:N:364:LYS:HD3	1:N:364:LYS:HA	1.74	0.45
1:N:419:LEU:HD22	1:N:447:MET:HG3	1.99	0.45

Continued on next page...

For Manuscript Review

Continued from previous page...

Atom-1	Atom-2	Interatomic distance (Å)	Clash overlap (Å)
2:S:95:VAL:HG13	2:T:3:ILE:HD11	1.98	0.45
2:T:95:VAL:HG13	2:U:3:ILE:HD11	1.97	0.45
1:B:198:GLY:N	1:B:328:ASP:O	2.50	0.45
1:C:198:GLY:N	1:C:328:ASP:O	2.50	0.45
1:C:224:ASP:N	1:C:301:ILE:O	2.48	0.45
1:C:248:LEU:HD22	1:C:323:VAL:HG21	1.98	0.45
1:C:402:ALA:O	1:C:496:PRO:HG3	2.16	0.45
1:D:204:PHE:CD1	1:D:266:THR:HG21	2.52	0.45
1:E:199:TYR:OH	1:E:211:GLY:O	2.23	0.45
1:E:431:GLY:N	1:E:437:ASN:OD1	2.49	0.45
1:F:268:ARG:NH1	2:T:26:VAL:HG11	2.32	0.45
1:F:381:VAL:HG13	1:F:392:LYS:HE3	1.99	0.45
1:G:222:LEU:HD22	1:G:293:ALA:HB2	1.98	0.45
1:G:429:LEU:HD23	1:G:440:ILE:HG12	1.98	0.45
1:H:465:VAL:HA	1:H:485:TYR:OH	2.17	0.45
1:I:203:TYR:HE2	1:J:181:THR:HA	1.81	0.45
1:I:346:VAL:O	1:I:350:ARG:HG2	2.16	0.45
1:I:487:ASN:HB3	1:I:490:ASP:OD2	2.17	0.45
1:J:134:LEU:HD21	1:J:425:LYS:NZ	2.32	0.45
1:J:140:ASP:O	1:J:144:ILE:HG12	2.16	0.45
1:J:423:ALA:HB2	1:J:447:MET:HB2	1.98	0.45
1:L:15:LYS:HD3	1:L:18:ARG:NH2	2.30	0.45
1:L:197:ARG:HE	1:L:279:PRO:HA	1.81	0.45
1:M:351:GLN:HA	1:M:354:GLU:OE1	2.17	0.45
1:M:417:VAL:HG21	1:M:488:MET:HG3	1.97	0.45
1:N:216:GLU:HG2	1:N:322:ARG:HD2	1.98	0.45
1:N:415:GLY:HA2	6:N:601:ADP:N3	2.32	0.45
2:O:12:VAL:CG2	2:O:84:LEU:HB2	2.47	0.45
2:P:88:GLU:OE1	2:Q:7:HIS:NE2	2.47	0.45
1:A:218:PRO:O	1:A:319:GLN:HA	2.17	0.45
1:A:308:GLU:HB2	1:A:311:LYS:HG3	1.99	0.45
1:A:342:ILE:HG23	1:A:372:LEU:HB3	1.98	0.45
1:B:265:ASN:HA	1:B:268:ARG:HB2	1.99	0.45
1:C:152:ALA:HB2	1:C:399:ALA:HB2	1.99	0.45
1:D:102:GLU:CB	1:D:442:VAL:HG13	2.46	0.45
1:D:219:PHE:CE2	1:D:314:LEU:HD23	2.52	0.45
1:F:452:ARG:HH21	1:F:470:LYS:NZ	2.15	0.45
1:G:322:ARG:O	1:G:333:ILE:N	2.45	0.45
1:H:252:GLU:O	1:H:277:LYS:HG3	2.17	0.45
1:I:192:GLY:HA3	1:I:376:VAL:HG13	1.98	0.45
1:J:102:GLU:HB3	1:J:442:VAL:HG22	1.98	0.45

Continued on next page...

For Manuscript Review

Continued from previous page...

Atom-1	Atom-2	Interatomic distance (Å)	Clash overlap (Å)
1:K:193:MET:HG2	1:K:295:LEU:HD13	1.98	0.45
1:L:16:MET:HE2	1:L:66:PHE:O	2.17	0.45
1:N:122:LYS:NZ	1:N:431:GLY:HA2	2.32	0.45
1:A:31:LEU:HD13	1:A:90:THR:HB	1.99	0.45
1:A:102:GLU:CB	1:A:442:VAL:HG13	2.47	0.45
1:A:106:ALA:O	1:A:111:MET:HG2	2.17	0.45
1:B:205:ILE:HG23	1:B:212:ALA:O	2.17	0.45
1:B:361:ASP:O	1:B:365:LEU:HD23	2.15	0.45
1:B:381:VAL:HG13	1:B:392:LYS:HE3	1.99	0.45
1:C:262:LEU:O	1:C:266:THR:HG23	2.16	0.45
1:C:320:ALA:HA	1:C:335:GLY:HA2	1.97	0.45
1:D:220:ILE:HG22	1:D:222:LEU:HG	1.99	0.45
1:D:510:VAL:HG23	1:E:385:THR:HG21	1.98	0.45
1:E:141:SER:HB2	1:E:163:ALA:HB1	1.99	0.45
1:F:66:PHE:HA	1:F:69:MET:HE3	1.98	0.45
1:F:199:TYR:OH	1:F:211:GLY:O	2.17	0.45
1:H:76:GLU:O	1:H:80:LYS:HG3	2.17	0.45
1:H:219:PHE:HD2	1:H:240:VAL:HG22	1.80	0.45
1:H:349:ILE:HG12	1:H:368:ARG:NH2	2.31	0.45
1:K:342:ILE:O	1:K:346:VAL:HG23	2.17	0.45
1:L:66:PHE:O	1:L:69:MET:HB3	2.17	0.45
1:L:339:GLU:HA	1:L:342:ILE:HB	1.99	0.45
1:M:179:ASP:OD1	1:M:180:GLY:N	2.50	0.45
1:M:223:ALA:O	1:M:251:ALA:HA	2.17	0.45
2:T:40:VAL:HG22	2:T:63:ASP:O	2.17	0.45
1:A:68:ASN:O	1:A:72:GLN:HG2	2.16	0.45
1:A:114:MET:SD	1:A:118:ARG:NH2	2.88	0.45
1:C:87:ASP:OD1	1:C:88:GLY:N	2.50	0.45
1:F:136:VAL:HG23	1:F:411:VAL:HG23	1.99	0.45
1:F:138:CYS:O	1:F:407:VAL:HA	2.17	0.45
1:F:168:LYS:HD3	1:F:168:LYS:HA	1.70	0.45
1:F:226:LYS:NZ	1:F:255:GLU:HG3	2.31	0.45
1:H:32:GLY:HA2	6:H:601:ADP:O4’	2.17	0.45
1:H:199:TYR:CE2	1:H:327:LYS:HA	2.52	0.45
1:I:114:MET:SD	1:I:516:THR:HG22	2.56	0.45
1:L:230:ILE:HD13	1:L:261:THR:HB	1.98	0.45
1:L:419:LEU:HD22	1:L:447:MET:SD	2.56	0.45
1:M:98:ALA:O	1:M:102:GLU:HG2	2.17	0.45
1:M:185:ASP:HA	1:M:381:VAL:HA	1.98	0.45
1:N:429:LEU:HB3	1:N:440:ILE:HG21	1.98	0.45
2:O:78:ILE:HG22	2:U:37:ARG:HH22	1.82	0.45

Continued on next page...

For Manuscript Review

Continued from previous page...

Atom-1	Atom-2	Interatomic distance (Å)	Clash overlap (Å)
2:O:94:ILE:HD11	2:P:4:ARG:HH11	1.81	0.45
2:Q:10:VAL:O	2:Q:86:MET:N	2.47	0.45
2:Q:69:ASP:HA	2:Q:73:VAL:HG21	1.99	0.45
2:T:11:ILE:HG23	2:T:83:VAL:HB	1.99	0.45
1:D:381:VAL:O	1:D:389:MET:HE1	2.17	0.45
1:E:149:THR:HG21	1:E:156:GLU:OE2	2.16	0.45
1:G:201:SER:HB3	1:G:204:PHE:CE2	2.51	0.45
1:I:122:LYS:NZ	1:I:431:GLY:HA2	2.30	0.45
1:K:82:ASN:O	1:K:86:GLY:N	2.31	0.45
1:K:265:ASN:O	1:K:269:GLY:N	2.50	0.45
1:K:366:GLN:HA	1:K:369:VAL:HG22	1.99	0.45
1:B:165:ALA:HB2	1:B:379:ILE:HD11	1.99	0.44
1:B:223:ALA:HB3	1:B:251:ALA:HB2	1.99	0.44
1:C:287:ALA:HA	1:C:345:ARG:HH21	1.81	0.44
1:D:230:ILE:H	1:D:230:ILE:HD12	1.81	0.44
1:E:128:VAL:HG21	1:E:505:GLN:HG3	1.98	0.44
1:E:475:ASN:HB2	1:E:487:ASN:ND2	2.31	0.44
1:F:77:VAL:HG12	1:F:92:ALA:HB1	1.98	0.44
1:F:361:ASP:O	1:F:365:LEU:HG	2.17	0.44
1:G:124:VAL:HG11	1:G:508:ALA:CB	2.47	0.44
1:G:197:ARG:HD2	1:G:277:LYS:HB2	1.98	0.44
1:J:36:ARG:HE	1:J:457:ASN:HA	1.82	0.44
1:J:76:GLU:O	1:J:80:LYS:HG3	2.16	0.44
1:J:141:SER:HA	1:J:144:ILE:HD11	1.99	0.44
1:J:195:PHE:CE2	1:J:197:ARG:HB2	2.52	0.44
1:L:64:ASP:HB3	1:L:67:GLU:HB2	2.00	0.44
1:L:130:GLU:HG2	1:L:422:VAL:HG22	1.99	0.44
1:L:130:GLU:OE1	1:L:426:LEU:HG	2.17	0.44
1:L:433:ASN:OD1	1:L:434:GLU:N	2.50	0.44
1:L:458:CYS:SG	1:L:480:ALA:HB1	2.57	0.44
1:M:174:VAL:HG11	1:M:194:GLN:HB2	1.99	0.44
1:M:429:LEU:HB3	1:M:440:ILE:HG21	1.98	0.44
2:S:10:VAL:HG11	2:S:91:ILE:HD11	1.99	0.44
1:C:180:GLY:N	1:C:381:VAL:O	2.31	0.44
1:C:219:PHE:CE1	1:C:245:LYS:HD2	2.53	0.44
1:C:361:ASP:O	1:C:365:LEU:HG	2.16	0.44
1:D:414:GLY:HA3	1:D:493:ILE:HG22	1.99	0.44
1:E:383:ALA:HB1	1:E:388:GLU:HG2	2.00	0.44
1:E:429:LEU:HB3	1:E:440:ILE:HG21	1.99	0.44
1:E:448:GLU:OE1	1:E:470:LYS:NZ	2.42	0.44
1:F:69:MET:O	1:F:73:MET:HG2	2.16	0.44

Continued on next page...

For Manuscript Review

Continued from previous page...

Atom-1	Atom-2	Interatomic distance (Å)	Clash overlap (Å)
1:F:346:VAL:HA	1:F:349:ILE:HD12	2.00	0.44
1:F:352:GLN:O	1:F:356:ALA:N	2.50	0.44
1:G:102:GLU:OE1	1:G:445:ARG:NE	2.38	0.44
1:G:215:LEU:HD12	1:G:248:LEU:HB2	1.98	0.44
1:H:142:LYS:NZ	1:H:146:GLN:HG3	2.32	0.44
1:H:222:LEU:HD23	1:H:250:ILE:HB	1.99	0.44
1:H:287:ALA:HB1	1:H:368:ARG:CZ	2.48	0.44
1:H:294:THR:HG21	1:H:345:ARG:HB2	1.98	0.44
1:K:5:ASP:HB3	1:K:522:THR:HG22	1.98	0.44
1:K:15:LYS:HD3	1:K:18:ARG:NH2	2.32	0.44
1:L:95:LEU:O	1:L:99:ILE:HG13	2.17	0.44
1:L:141:SER:HA	1:L:144:ILE:HD13	1.99	0.44
1:N:230:ILE:O	1:N:234:LEU:HG	2.17	0.44
1:N:252:GLU:O	1:N:277:LYS:HG3	2.17	0.44
2:S:11:ILE:HG23	2:S:83:VAL:HB	2.00	0.44
1:C:126:ALA:O	1:C:130:GLU:HG2	2.18	0.44
1:D:134:LEU:HD23	1:D:418:ALA:HB1	1.99	0.44
1:D:479:ASN:HB3	1:D:484:GLU:HG2	1.99	0.44
1:E:16:MET:SD	1:E:514:MET:HE1	2.58	0.44
1:E:69:MET:HB2	1:F:47:PRO:CG	2.46	0.44
1:F:353:ILE:HG23	1:F:362:ARG:HB2	1.99	0.44
1:F:420:ILE:CG2	1:F:470:LYS:HG2	2.47	0.44
1:G:251:ALA:O	1:G:277:LYS:HA	2.18	0.44
1:H:230:ILE:O	1:H:234:LEU:HG	2.18	0.44
1:I:262:LEU:HD22	1:I:273:VAL:HG11	1.98	0.44
1:J:32:GLY:HA2	6:J:601:ADP:O4'	2.17	0.44
1:J:168:LYS:HG2	1:J:189:VAL:HG13	2.00	0.44
1:J:195:PHE:CD2	1:J:279:PRO:HB3	2.53	0.44
1:K:37:ASN:ND2	1:K:51:LYS:HE2	2.32	0.44
1:K:345:ARG:HD2	1:K:348:GLN:OE1	2.17	0.44
1:L:124:VAL:HG11	1:L:508:ALA:CB	2.47	0.44
1:L:193:MET:HE3	1:L:295:LEU:HD13	2.00	0.44
1:L:263:VAL:O	1:L:266:THR:OG1	2.27	0.44
1:L:465:VAL:HA	1:L:485:TYR:OH	2.17	0.44
1:M:112:ASN:OD1	1:M:114:MET:N	2.50	0.44
1:M:345:ARG:HD2	1:M:348:GLN:OE1	2.18	0.44
1:M:349:ILE:HG12	1:M:368:ARG:NH2	2.32	0.44
1:A:268:ARG:NH1	2:O:26:VAL:HG11	2.32	0.44
1:B:197:ARG:HD2	1:B:277:LYS:HB2	1.99	0.44
1:B:215:LEU:HD12	1:B:248:LEU:HB2	2.00	0.44
1:B:232:GLU:HG2	1:B:310:GLU:OE2	2.17	0.44

Continued on next page...

For Manuscript Review

Continued from previous page...

Atom-1	Atom-2	Interatomic distance (Å)	Clash overlap (Å)
1:C:200:LEU:HD21	1:C:277:LYS:HG3	1.98	0.44
1:D:283:ASP:OD1	1:D:284:ARG:N	2.50	0.44
3:D:601:ATP:H5'2	3:D:601:ATP:H8	1.81	0.44
1:E:6:VAL:HG13	1:E:521:VAL:HG22	2.00	0.44
1:E:313:THR:O	1:E:317:LEU:HG	2.17	0.44
1:F:224:ASP:OD1	1:F:285:ARG:NH1	2.51	0.44
1:F:231:ARG:NH2	2:T:31:ALA:O	2.40	0.44
1:G:219:PHE:CD2	1:G:240:VAL:HG22	2.44	0.44
1:G:265:ASN:O	1:G:269:GLY:N	2.51	0.44
1:K:85:ALA:CB	1:K:499:VAL:HA	2.41	0.44
1:K:358:SER:O	1:K:362:ARG:HG2	2.17	0.44
1:L:145:ALA:HA	1:L:159:GLY:C	2.38	0.44
1:L:221:LEU:HD23	1:L:249:ILE:HD12	1.99	0.44
1:M:130:GLU:OE1	1:M:426:LEU:HG	2.18	0.44
1:M:364:LYS:HD3	1:M:364:LYS:HA	1.76	0.44
2:Q:8:ASP:HA	2:Q:57:LEU:HD11	1.99	0.44
2:S:11:ILE:HB	2:S:42:ALA:HB3	1.99	0.44
1:A:440:ILE:O	1:A:444:LEU:HG	2.18	0.44
1:B:134:LEU:HD23	1:B:418:ALA:HB1	2.00	0.44
1:D:145:ALA:HA	1:D:159:GLY:C	2.37	0.44
1:E:236:VAL:HG21	1:E:317:LEU:HD21	1.99	0.44
1:E:479:ASN:O	1:E:483:GLU:N	2.51	0.44
1:F:40:LEU:HD13	1:F:59:GLU:HG3	2.00	0.44
1:F:294:THR:HG22	1:F:341:ALA:HB1	1.99	0.44
1:G:349:ILE:HG23	1:G:365:LEU:CD1	2.47	0.44
1:H:215:LEU:HB2	1:H:323:VAL:HG22	1.99	0.44
1:I:218:PRO:HG3	1:I:323:VAL:HG22	1.99	0.44
1:I:294:THR:HG21	1:I:345:ARG:HB2	2.00	0.44
1:M:115:ASP:OD2	1:M:433:ASN:ND2	2.35	0.44
1:M:130:GLU:HB2	1:M:422:VAL:HG13	1.99	0.44
1:M:287:ALA:HB1	1:M:368:ARG:CZ	2.47	0.44
1:M:295:LEU:HA	1:M:342:ILE:HG12	1.99	0.44
2:Q:43:VAL:HB	2:Q:57:LEU:HD22	1.99	0.44
1:A:270:ILE:HG21	2:O:25:ILE:HA	1.99	0.44
1:B:381:VAL:HG12	1:B:389:MET:HE1	2.00	0.44
1:B:420:ILE:CG2	1:B:470:LYS:HG2	2.47	0.44
1:B:479:ASN:O	1:B:483:GLU:N	2.50	0.44
1:D:234:LEU:HD12	1:D:238:GLU:OE2	2.18	0.44
1:D:381:VAL:HG13	1:D:392:LYS:HE3	2.00	0.44
1:D:417:VAL:O	1:D:421:ARG:HG2	2.18	0.44
1:F:222:LEU:O	1:F:301:ILE:N	2.32	0.44

Continued on next page...

For Manuscript Review

Continued from previous page...

Atom-1	Atom-2	Interatomic distance (Å)	Clash overlap (Å)
1:F:510:VAL:O	1:F:514:MET:HG2	2.18	0.44
1:G:112:ASN:ND2	1:G:115:ASP:OD2	2.43	0.44
1:G:313:THR:O	1:G:317:LEU:HG	2.17	0.44
1:G:346:VAL:HA	1:G:349:ILE:HB	2.00	0.44
1:H:195:PHE:CE2	1:H:197:ARG:HB2	2.53	0.44
1:I:29:VAL:O	1:I:36:ARG:N	2.26	0.44
1:I:193:MET:SD	1:I:295:LEU:HD22	2.58	0.44
1:I:216:GLU:HG2	1:I:322:ARG:HD2	1.99	0.44
1:I:475:ASN:HB2	1:I:487:ASN:HD21	1.82	0.44
1:J:31:LEU:HD23	1:J:453:GLN:HB3	2.00	0.44
1:J:177:VAL:C	1:J:393:LYS:HZ2	2.21	0.44
1:K:149:THR:OG1	1:K:156:GLU:HA	2.18	0.44
1:M:148:GLY:CA	1:M:399:ALA:HB1	2.47	0.44
1:M:219:PHE:CD2	1:M:245:LYS:HD2	2.52	0.44
1:M:230:ILE:HD13	1:M:261:THR:HB	1.99	0.44
1:M:458:CYS:SG	1:M:480:ALA:HB1	2.57	0.44
1:N:5:ASP:N	1:N:522:THR:O	2.44	0.44
1:N:7:LYS:HE3	1:N:15:LYS:HG3	1.98	0.44
2:R:11:ILE:O	2:R:41:LEU:N	2.40	0.44
1:A:158:VAL:HG22	1:A:396:VAL:HG22	1.99	0.44
1:A:203:TYR:CE1	1:G:305:ILE:HG12	2.52	0.44
1:A:222:LEU:HD21	1:A:292:ILE:HG22	1.99	0.44
1:B:207:LYS:NZ	1:B:214:GLU:HB2	2.32	0.44
1:B:222:LEU:HB2	1:B:300:VAL:HA	2.00	0.44
1:C:77:VAL:HG12	1:C:92:ALA:HB1	1.99	0.44
1:C:134:LEU:HD23	1:C:418:ALA:HB1	2.00	0.44
1:C:230:ILE:O	1:C:234:LEU:N	2.51	0.44
1:C:239:ALA:HA	1:C:242:LYS:HE2	1.98	0.44
1:E:12:ALA:O	1:E:16:MET:HG2	2.18	0.44
1:E:511:ALA:O	1:E:515:ILE:HG12	2.17	0.44
1:F:152:ALA:O	1:F:395:ARG:HD2	2.18	0.44
1:F:169:VAL:HB	1:F:377:ALA:CB	2.47	0.44
1:G:178:GLU:N	1:G:379:ILE:O	2.47	0.44
1:J:115:ASP:CG	1:J:118:ARG:HH21	2.21	0.44
1:J:123:ALA:HB3	1:J:443:ALA:HB3	2.00	0.44
1:J:284:ARG:O	1:J:288:MET:HG2	2.18	0.44
1:J:319:GLN:HB2	1:J:336:VAL:HB	2.00	0.44
1:J:359:ASP:HA	1:J:362:ARG:HB3	1.99	0.44
1:K:323:VAL:HB	1:K:332:ILE:HG22	2.00	0.44
1:L:124:VAL:HG11	1:L:508:ALA:HB2	1.99	0.44
1:L:299:THR:N	1:L:316:ASP:O	2.44	0.44

Continued on next page...

For Manuscript Review

Continued from previous page...

Atom-1	Atom-2	Interatomic distance (Å)	Clash overlap (Å)
1:M:24:ALA:HB3	1:M:97:GLN:HG3	2.00	0.44
1:M:349:ILE:HG12	1:M:368:ARG:CZ	2.48	0.44
2:P:6:LEU:HB3	2:P:7:HIS:CD2	2.52	0.44
1:A:350:ARG:NH2	1:A:369:VAL:HG11	2.32	0.44
1:B:128:VAL:HG21	1:B:505:GLN:HG3	2.00	0.44
1:D:462:PRO:O	1:D:466:ALA:CB	2.66	0.44
1:E:523:ASP:OD1	1:E:524:LEU:N	2.51	0.44
1:F:15:LYS:HB2	1:F:520:MET:HE3	1.99	0.44
1:F:431:GLY:N	1:F:437:ASN:OD1	2.43	0.44
1:G:77:VAL:HG12	1:G:92:ALA:HB1	1.99	0.44
1:G:455:VAL:CG1	1:G:460:GLU:HB2	2.47	0.44
1:H:34:LYS:HB2	1:H:458:CYS:SG	2.58	0.44
1:H:217:SER:O	1:H:245:LYS:HD3	2.17	0.44
1:H:350:ARG:HA	1:H:353:ILE:HD12	1.99	0.44
1:H:495:ASP:OD2	6:H:601:ADP:O2'	2.33	0.44
1:J:230:ILE:O	1:J:234:LEU:HG	2.18	0.44
1:K:498:LYS:HG3	1:K:501:ARG:NH2	2.33	0.44
1:L:69:MET:HG2	1:L:520:MET:HE3	2.00	0.44
1:L:220:ILE:HD11	1:L:250:ILE:HD12	2.00	0.44
1:M:178:GLU:HB2	1:M:380:LYS:HD3	1.99	0.44
1:N:130:GLU:HB2	1:N:422:VAL:HG13	2.00	0.44
2:R:75:SER:HB3	2:R:82:GLU:OE1	2.18	0.44
1:A:239:ALA:HB1	1:A:314:LEU:HD11	2.00	0.44
1:A:419:LEU:HD23	1:A:419:LEU:HA	1.87	0.44
1:C:16:MET:HE3	1:C:520:MET:HE2	2.00	0.44
1:C:232:GLU:HB3	1:C:309:LEU:HD23	2.00	0.44
1:C:279:PRO:O	1:C:285:ARG:HA	2.18	0.44
1:D:198:GLY:N	1:D:328:ASP:O	2.51	0.44
1:E:82:ASN:HB2	1:E:89:THR:HG21	2.00	0.44
1:E:452:ARG:HH21	1:E:470:LYS:NZ	2.16	0.44
1:F:250:ILE:HD13	1:F:292:ILE:HD13	2.00	0.44
1:H:283:ASP:OD1	1:H:284:ARG:N	2.51	0.44
1:I:452:ARG:HH12	1:I:463:SER:HA	1.83	0.44
1:J:176:THR:O	1:J:379:ILE:N	2.45	0.44
1:J:246:PRO:HA	1:J:272:LYS:HB2	2.00	0.44
1:K:34:LYS:HB2	1:K:458:CYS:SG	2.58	0.44
1:M:417:VAL:O	1:M:421:ARG:HG2	2.17	0.44
1:N:115:ASP:CG	1:N:118:ARG:HH21	2.21	0.44
2:P:9:ARG:HA	2:P:87:SER:HA	1.99	0.44
2:Q:5:PRO:O	2:Q:44:GLY:HA2	2.18	0.44
2:Q:94:ILE:HG23	2:R:6:LEU:HD11	1.99	0.44

Continued on next page...

For Manuscript Review

Continued from previous page...

Atom-1	Atom-2	Interatomic distance (Å)	Clash overlap (Å)
1:A:34:LYS:HG2	1:G:114:MET:HE2	2.00	0.43
1:A:162:ILE:HG12	1:A:400:LEU:HD13	1.99	0.43
1:A:349:ILE:HG23	1:A:365:LEU:HB3	1.99	0.43
1:B:220:ILE:HG13	1:B:248:LEU:HD23	2.00	0.43
1:B:421:ARG:NH2	1:B:469:VAL:O	2.44	0.43
1:C:221:LEU:HD21	1:C:309:LEU:HD11	1.99	0.43
1:C:443:ALA:O	1:C:447:MET:HG2	2.18	0.43
1:D:184:GLN:N	1:D:382:GLY:HA3	2.32	0.43
1:D:251:ALA:O	1:D:278:ALA:N	2.51	0.43
1:D:264:VAL:HA	1:D:267:MET:SD	2.57	0.43
1:D:305:ILE:O	1:E:264:VAL:HG22	2.18	0.43
1:D:420:ILE:CG2	1:D:470:LYS:HG2	2.48	0.43
1:E:458:CYS:HB3	1:E:483:GLU:OE2	2.17	0.43
1:F:417:VAL:O	1:F:421:ARG:HG2	2.18	0.43
1:G:217:SER:HA	1:G:320:ALA:O	2.18	0.43
1:G:270:ILE:HG21	2:U:25:ILE:HA	2.00	0.43
1:H:193:MET:H	1:H:332:ILE:HG13	1.83	0.43
1:I:221:LEU:HD23	1:I:249:ILE:HD12	1.99	0.43
1:J:69:MET:HG2	1:K:47:PRO:CG	2.48	0.43
1:J:383:ALA:HB1	1:J:388:GLU:HB3	2.00	0.43
1:K:252:GLU:O	1:K:277:LYS:HG3	2.18	0.43
1:K:349:ILE:O	1:K:353:ILE:HG13	2.18	0.43
1:K:414:GLY:O	1:K:417:VAL:HG22	2.18	0.43
1:A:479:ASN:HB3	1:A:484:GLU:HG2	2.00	0.43
1:E:220:ILE:HG22	1:E:222:LEU:HG	2.00	0.43
1:F:219:PHE:CZ	1:F:314:LEU:HD23	2.54	0.43
1:F:359:ASP:HA	1:F:362:ARG:HE	1.81	0.43
1:G:239:ALA:HB1	1:G:314:LEU:HD11	1.99	0.43
1:H:82:ASN:O	1:H:86:GLY:N	2.32	0.43
1:H:364:LYS:HA	1:H:364:LYS:HD3	1.81	0.43
1:I:342:ILE:O	1:I:346:VAL:HG23	2.18	0.43
1:J:56:VAL:HG12	1:J:60:ILE:HD11	1.99	0.43
1:J:104:LEU:HD23	1:J:104:LEU:HA	1.60	0.43
1:L:262:LEU:HB3	1:L:273:VAL:HG11	2.00	0.43
1:M:386:GLU:O	1:M:389:MET:HB3	2.18	0.43
1:N:34:LYS:HB2	1:N:458:CYS:SG	2.58	0.43
1:N:262:LEU:HB3	1:N:273:VAL:HG11	2.00	0.43
1:N:349:ILE:HG21	1:N:368:ARG:CB	2.45	0.43
2:Q:9:ARG:HA	2:Q:87:SER:HA	2.00	0.43
1:C:339:GLU:O	1:C:342:ILE:HB	2.18	0.43
1:E:6:VAL:HG22	1:E:521:VAL:HG22	2.00	0.43

Continued on next page...

For Manuscript Review

Continued from previous page...

Atom-1	Atom-2	Interatomic distance (Å)	Clash overlap (Å)
1:E:12:ALA:HA	1:E:520:MET:HE2	2.00	0.43
1:E:207:LYS:NZ	1:E:214:GLU:HB2	2.33	0.43
1:F:440:ILE:O	1:F:444:LEU:HG	2.17	0.43
1:G:138:CYS:HB3	1:G:406:ALA:HB1	2.01	0.43
1:G:226:LYS:HZ1	1:G:255:GLU:N	2.17	0.43
1:G:268:ARG:NH1	2:U:26:VAL:HG11	2.33	0.43
1:G:452:ARG:NH1	7:G:714:HOH:O	2.30	0.43
1:H:179:ASP:HA	1:H:381:VAL:HG22	2.00	0.43
1:H:265:ASN:O	1:H:269:GLY:N	2.51	0.43
1:H:475:ASN:HB2	1:H:487:ASN:ND2	2.33	0.43
1:I:390:LYS:NZ	1:I:393:LYS:HG2	2.33	0.43
1:J:20:VAL:HG22	1:J:74:VAL:CG2	2.47	0.43
1:K:4:LYS:HB3	1:K:521:VAL:HG13	2.00	0.43
1:K:195:PHE:CZ	1:K:250:ILE:HD13	2.53	0.43
1:K:468:THR:HB	1:K:485:TYR:CE2	2.53	0.43
1:L:140:ASP:O	1:L:144:ILE:HD12	2.18	0.43
1:L:218:PRO:HG3	1:L:323:VAL:HG22	2.00	0.43
1:L:349:ILE:HG21	1:L:368:ARG:CB	2.49	0.43
1:M:230:ILE:HG12	1:M:261:THR:HG21	2.00	0.43
1:N:219:PHE:CD2	1:N:314:LEU:HD22	2.52	0.43
2:R:12:VAL:HG21	2:R:86:MET:HE1	1.99	0.43
1:B:178:GLU:HG3	1:B:380:LYS:HG3	2.01	0.43
1:D:162:ILE:HG23	1:D:400:LEU:HD12	2.00	0.43
1:D:226:LYS:NZ	1:D:253:ASP:HB3	2.33	0.43
1:E:108:ALA:HB1	1:K:109:ALA:HB1	1.99	0.43
1:F:221:LEU:HD12	1:F:236:VAL:HG11	2.00	0.43
1:G:227:ILE:HG23	1:G:233:MET:SD	2.58	0.43
1:H:69:MET:SD	1:H:520:MET:HE3	2.58	0.43
1:H:142:LYS:HD2	1:H:145:ALA:HB3	2.01	0.43
1:H:405:ALA:HB1	1:H:498:LYS:HB3	2.00	0.43
1:H:433:ASN:HB3	1:H:436:GLN:HG3	2.00	0.43
1:I:124:VAL:HG13	1:I:504:LEU:HG	2.01	0.43
1:J:15:LYS:HD3	1:J:18:ARG:NH2	2.34	0.43
1:J:127:ALA:HB3	1:J:504:LEU:HD21	2.00	0.43
1:K:102:GLU:HG3	1:K:445:ARG:NH1	2.33	0.43
1:K:199:TYR:CE2	1:K:327:LYS:HA	2.53	0.43
1:K:219:PHE:CD2	1:K:314:LEU:HD22	2.54	0.43
1:L:31:LEU:HB3	1:L:453:GLN:HG3	1.99	0.43
1:L:262:LEU:HD13	1:L:273:VAL:HG11	2.00	0.43
1:M:224:ASP:OD2	1:M:286:LYS:HG2	2.18	0.43
1:M:381:VAL:HG23	1:M:389:MET:SD	2.58	0.43

Continued on next page...

For Manuscript Review

Continued from previous page...

Atom-1	Atom-2	Interatomic distance (Å)	Clash overlap (Å)
2:T:75:SER:HA	2:T:83:VAL:O	2.17	0.43
1:A:124:VAL:HG22	1:A:504:LEU:HD11	2.00	0.43
1:A:243:ALA:HB2	1:A:314:LEU:HD21	2.01	0.43
1:A:392:LYS:O	1:A:396:VAL:HG23	2.19	0.43
1:C:106:ALA:O	1:C:111:MET:HG2	2.19	0.43
1:D:260:ALA:HA	1:D:263:VAL:HG22	2.00	0.43
1:E:239:ALA:HB1	1:E:314:LEU:HD11	2.00	0.43
1:E:240:VAL:HG21	1:E:247:LEU:CD1	2.48	0.43
1:F:20:VAL:HA	1:F:74:VAL:HG11	2.00	0.43
1:F:127:ALA:N	1:F:426:LEU:HD11	2.34	0.43
1:F:178:GLU:N	1:F:379:ILE:O	2.42	0.43
1:F:205:ILE:HG23	1:F:212:ALA:O	2.19	0.43
1:G:113:PRO:CB	1:G:516:THR:HA	2.45	0.43
1:G:219:PHE:CE2	1:G:314:LEU:HD23	2.53	0.43
1:G:268:ARG:HG3	2:U:26:VAL:HG21	2.00	0.43
1:H:30:THR:HB	1:H:51:LYS:C	2.38	0.43
1:H:152:ALA:HB3	1:H:155:ASP:HB2	2.01	0.43
1:H:221:LEU:N	1:H:248:LEU:O	2.28	0.43
1:I:411:VAL:HG12	1:I:496:PRO:HA	2.00	0.43
1:J:276:VAL:HG12	1:J:277:LYS:O	2.17	0.43
1:J:455:VAL:CG1	1:J:460:GLU:HB2	2.45	0.43
1:K:115:ASP:OD2	1:K:433:ASN:ND2	2.35	0.43
1:K:168:LYS:HE2	1:K:168:LYS:HB2	1.87	0.43
1:L:221:LEU:HD23	1:L:249:ILE:HG23	2.01	0.43
1:M:16:MET:O	1:M:20:VAL:HG23	2.18	0.43
1:M:42:LYS:N	1:M:47:PRO:HB3	2.33	0.43
1:M:197:ARG:NH1	1:M:277:LYS:HD3	2.33	0.43
2:P:13:LYS:HG2	2:P:41:LEU:HD21	2.00	0.43
2:T:14:ARG:NH1	2:T:34:LYS:HZ2	2.16	0.43
2:U:10:VAL:O	2:U:86:MET:HG3	2.18	0.43
1:B:200:LEU:N	1:B:275:ALA:O	2.35	0.43
1:B:440:ILE:O	1:B:444:LEU:HG	2.18	0.43
1:C:34:LYS:O	1:C:36:ARG:NH1	2.52	0.43
1:C:219:PHE:HB3	1:C:317:LEU:HB3	2.01	0.43
1:D:452:ARG:HH22	1:D:470:LYS:HE2	1.83	0.43
1:E:62:LEU:O	1:E:68:ASN:HB2	2.18	0.43
1:E:270:ILE:HG21	2:S:25:ILE:HA	2.00	0.43
1:E:349:ILE:HG23	1:E:365:LEU:CD1	2.48	0.43
1:E:479:ASN:CG	1:E:493:ILE:HD11	2.39	0.43
1:H:235:PRO:CG	1:H:310:GLU:HA	2.47	0.43
1:H:432:GLN:HB2	1:H:436:GLN:NE2	2.34	0.43

Continued on next page...

For Manuscript Review

Continued from previous page...

Atom-1	Atom-2	Interatomic distance (Å)	Clash overlap (Å)
1:H:468:THR:HB	1:H:485:TYR:CE2	2.54	0.43
1:I:324:VAL:HB	1:I:331:THR:HB	2.00	0.43
1:I:365:LEU:HD23	1:I:368:ARG:HE	1.83	0.43
1:L:477:GLY:N	1:L:486:GLY:O	2.51	0.43
1:L:511:ALA:O	1:L:515:ILE:HG13	2.19	0.43
1:N:123:ALA:HB3	1:N:443:ALA:HB3	2.01	0.43
1:N:222:LEU:HD11	1:N:292:ILE:HG22	1.99	0.43
2:P:15:LYS:HE2	2:P:64:ILE:HG23	2.01	0.43
2:R:63:ASP:HB3	2:R:94:ILE:HG23	2.00	0.43
2:U:74:LYS:HE3	2:U:74:LYS:HB3	1.72	0.43
1:A:16:MET:SD	1:A:514:MET:HE1	2.58	0.43
1:A:152:ALA:O	1:A:395:ARG:HD2	2.18	0.43
1:A:218:PRO:HG2	1:A:323:VAL:HG23	2.00	0.43
1:B:339:GLU:HG3	1:B:343:GLN:OE1	2.18	0.43
1:B:465:VAL:HA	1:B:485:TYR:OH	2.17	0.43
1:C:429:LEU:HB3	1:C:440:ILE:HG21	2.01	0.43
1:D:207:LYS:HE2	1:D:212:ALA:HB3	2.00	0.43
1:F:149:THR:HG22	1:F:154:SER:HA	1.99	0.43
1:F:287:ALA:HB1	1:F:368:ARG:CZ	2.49	0.43
1:F:415:GLY:HA2	3:F:601:ATP:H1'	2.01	0.43
1:F:469:VAL:HG22	1:F:477:GLY:C	2.39	0.43
1:G:115:ASP:CG	1:G:433:ASN:HD21	2.22	0.43
1:H:102:GLU:HB3	1:H:442:VAL:HG22	2.01	0.43
1:H:345:ARG:HD2	1:H:348:GLN:OE1	2.19	0.43
1:I:345:ARG:HD2	1:I:348:GLN:OE1	2.18	0.43
1:J:9:GLY:O	1:J:13:ARG:HG2	2.19	0.43
1:J:162:ILE:HG22	1:J:166:MET:HE1	2.00	0.43
1:J:277:LYS:NZ	1:J:285:ARG:HH21	2.17	0.43
1:J:516:THR:O	1:K:37:ASN:HB2	2.19	0.43
1:K:197:ARG:NH1	1:K:277:LYS:HD3	2.33	0.43
1:K:230:ILE:O	1:K:234:LEU:HG	2.18	0.43
1:L:115:ASP:O	1:L:436:GLN:HG2	2.18	0.43
1:N:68:ASN:O	1:N:72:GLN:HG2	2.17	0.43
1:N:352:GLN:O	1:N:356:ALA:N	2.52	0.43
2:R:9:ARG:HA	2:R:87:SER:HA	2.00	0.43
1:A:455:VAL:HG13	1:A:460:GLU:HB2	2.00	0.43
1:B:102:GLU:CB	1:B:442:VAL:HG13	2.49	0.43
1:B:223:ALA:O	1:B:251:ALA:HA	2.19	0.43
1:B:252:GLU:HA	1:B:285:ARG:NH1	2.34	0.43
1:B:427:ALA:HA	1:B:444:LEU:HD13	2.00	0.43
1:C:239:ALA:HB1	1:C:314:LEU:HD12	1.99	0.43

Continued on next page...

For Manuscript Review

Continued from previous page...

Atom-1	Atom-2	Interatomic distance (Å)	Clash overlap (Å)
1:C:277:LYS:HB3	1:C:277:LYS:HE3	1.88	0.43
1:E:77:VAL:HG11	1:E:96:ALA:HB2	2.01	0.43
1:E:135:SER:HB3	1:E:497:THR:HG21	2.00	0.43
1:E:230:ILE:H	1:E:230:ILE:HD12	1.84	0.43
1:E:262:LEU:O	1:E:266:THR:HG23	2.19	0.43
1:E:295:LEU:HD12	1:E:342:ILE:HD11	2.00	0.43
1:F:222:LEU:HD22	1:F:293:ALA:HB2	2.01	0.43
1:F:225:LYS:HE2	1:F:225:LYS:HB2	1.83	0.43
1:G:452:ARG:HH21	1:G:470:LYS:NZ	2.16	0.43
1:H:190:VAL:O	1:H:376:VAL:HG22	2.19	0.43
1:K:185:ASP:HA	1:K:380:LYS:O	2.19	0.43
1:K:187:LEU:HB3	1:K:379:ILE:HG12	2.01	0.43
1:K:274:ALA:HB1	1:K:325:ILE:CD1	2.48	0.43
1:K:291:ASP:HB3	1:K:372:LEU:HD21	2.00	0.43
1:L:349:ILE:O	1:L:353:ILE:HG13	2.19	0.43
1:M:33:PRO:HD2	1:M:454:ILE:HG23	2.00	0.43
1:M:168:LYS:HE2	1:M:168:LYS:HB2	1.83	0.43
1:M:199:TYR:CE1	1:M:327:LYS:HA	2.54	0.43
1:N:81:ALA:HA	1:N:506:TYR:CD2	2.54	0.43
1:N:465:VAL:HA	1:N:485:TYR:OH	2.19	0.43
1:A:387:VAL:HA	1:A:390:LYS:HE2	2.01	0.43
1:A:452:ARG:HH21	1:A:470:LYS:NZ	2.17	0.43
1:B:69:MET:O	1:B:73:MET:HG2	2.18	0.43
1:B:270:ILE:HG22	1:B:271:VAL:HG13	2.00	0.43
1:B:339:GLU:O	1:B:342:ILE:HB	2.18	0.43
1:C:111:MET:HE1	1:C:116:LEU:HD21	2.00	0.43
1:C:227:ILE:HG23	1:C:233:MET:SD	2.59	0.43
1:C:239:ALA:HB1	1:C:314:LEU:CD1	2.49	0.43
1:D:209:GLU:HG2	1:D:210:THR:HG23	2.01	0.43
1:E:136:VAL:HG23	1:E:411:VAL:HG23	2.01	0.43
1:F:207:LYS:NZ	1:F:214:GLU:HB2	2.34	0.43
1:F:265:ASN:HA	1:F:268:ARG:HB2	2.01	0.43
1:F:308:GLU:HB2	1:F:311:LYS:HG3	2.01	0.43
1:F:421:ARG:O	1:F:425:LYS:HG3	2.19	0.43
1:F:429:LEU:O	1:F:430:ARG:NH1	2.52	0.43
1:F:453:GLN:NE2	1:F:457:ASN:OD1	2.52	0.43
1:G:207:LYS:NZ	1:G:214:GLU:HB2	2.34	0.43
1:H:290:GLN:HG3	1:H:345:ARG:NE	2.33	0.43
1:I:147:VAL:HG11	1:I:406:ALA:HB2	2.00	0.43
1:I:217:SER:HA	1:I:320:ALA:O	2.19	0.43
1:I:513:LEU:HA	1:I:513:LEU:HD23	1.87	0.43

Continued on next page...

For Manuscript Review

Continued from previous page...

Atom-1	Atom-2	Interatomic distance (Å)	Clash overlap (Å)
1:J:364:LYS:HD3	1:J:364:LYS:HA	1.67	0.43
1:J:390:LYS:HA	1:J:393:LYS:HB2	2.01	0.43
1:J:460:GLU:HG3	1:J:478:TYR:OH	2.18	0.43
1:L:69:MET:HE2	1:L:73:MET:HG3	2.00	0.43
1:L:104:LEU:HD23	1:L:104:LEU:HA	1.82	0.43
1:L:195:PHE:CE2	1:L:197:ARG:HB2	2.54	0.43
1:L:319:GLN:HB2	1:L:336:VAL:HB	2.01	0.43
1:M:15:LYS:HD3	1:M:18:ARG:NH2	2.34	0.43
1:M:150:ILE:HG13	1:M:494:LEU:H	1.84	0.43
1:M:301:ILE:HG21	1:M:309:LEU:HD23	2.00	0.43
1:M:333:ILE:HG12	1:M:376:VAL:HG11	2.00	0.43
1:N:287:ALA:HB1	1:N:368:ARG:CZ	2.48	0.43
1:N:513:LEU:HA	1:N:513:LEU:HD23	1.75	0.43
2:O:8:ASP:O	2:O:57:LEU:HD21	2.18	0.43
2:R:20:LYS:HE3	2:R:24:GLY:HA2	2.01	0.43
2:U:14:ARG:NH2	2:U:69:ASP:OD1	2.50	0.43
2:U:65:VAL:HG12	2:U:94:ILE:HG12	2.01	0.43
1:B:417:VAL:O	1:B:421:ARG:HG2	2.19	0.43
1:C:350:ARG:HA	1:C:353:ILE:HD12	2.01	0.43
1:C:452:ARG:NH1	7:C:707:HOH:O	2.30	0.43
1:E:10:ASN:O	1:E:14:VAL:HG23	2.19	0.43
1:E:361:ASP:O	1:E:365:LEU:HD23	2.19	0.43
1:G:417:VAL:O	1:G:421:ARG:HG2	2.19	0.43
1:H:289:LEU:HD22	1:H:300:VAL:HG13	2.00	0.43
1:I:39:VAL:HG22	1:I:49:ILE:HG23	2.01	0.43
1:I:124:VAL:HG11	1:I:508:ALA:HB2	2.01	0.43
1:I:252:GLU:O	1:I:277:LYS:HG3	2.19	0.43
1:J:130:GLU:OE1	1:J:426:LEU:HG	2.18	0.43
1:J:174:VAL:CG1	1:J:376:VAL:HG12	2.49	0.43
1:K:193:MET:SD	1:K:295:LEU:HD22	2.58	0.43
1:K:223:ALA:HA	1:K:301:ILE:HB	2.00	0.43
1:K:421:ARG:HH12	1:K:469:VAL:C	2.22	0.43
1:L:124:VAL:HG22	1:L:504:LEU:HD11	2.01	0.43
1:M:73:MET:SD	1:N:47:PRO:HD2	2.59	0.43
1:M:139:SER:O	1:M:171:LYS:HD3	2.18	0.43
1:N:149:THR:HG22	1:N:154:SER:HA	2.00	0.43
1:N:235:PRO:HG3	1:N:310:GLU:HA	2.01	0.43
2:P:20:LYS:HZ1	2:P:24:GLY:HA2	1.83	0.43
2:Q:86:MET:HE2	2:Q:90:ASP:HB2	2.01	0.43
2:R:88:GLU:HG2	2:R:91:ILE:HG13	2.01	0.43
2:S:67:PHE:HE2	2:S:69:ASP:HB3	1.83	0.43

Continued on next page...

For Manuscript Review

Continued from previous page...

Atom-1	Atom-2	Interatomic distance (Å)	Clash overlap (Å)
2:S:69:ASP:OD1	2:S:69:ASP:N	2.51	0.43
2:S:73:VAL:HG22	2:S:86:MET:SD	2.59	0.43
1:A:194:GLN:HA	1:A:331:THR:HA	2.01	0.42
1:A:232:GLU:HG2	1:A:310:GLU:OE2	2.19	0.42
1:B:131:LEU:HD21	1:B:419:LEU:HD23	2.01	0.42
1:B:186:GLU:HG3	1:B:380:LYS:HE2	2.01	0.42
1:B:291:ASP:OD1	1:B:345:ARG:HG2	2.19	0.42
1:C:35:GLY:O	1:C:51:LYS:HE2	2.19	0.42
1:C:54:VAL:HG11	1:C:82:ASN:HB2	2.01	0.42
1:D:207:LYS:HZ1	1:D:214:GLU:HB2	1.83	0.42
1:D:214:GLU:OE2	1:D:322:ARG:NH1	2.52	0.42
1:D:399:ALA:O	1:D:403:THR:HG23	2.18	0.42
1:E:203:TYR:HB2	1:E:263:VAL:HB	2.01	0.42
1:E:277:LYS:HE3	1:E:285:ARG:HH22	1.84	0.42
1:F:158:VAL:HG22	1:F:396:VAL:HG22	1.99	0.42
1:F:198:GLY:N	1:F:328:ASP:O	2.53	0.42
1:H:161:LEU:HG	1:H:187:LEU:HD23	2.00	0.42
1:H:169:VAL:CG2	1:H:377:ALA:HB2	2.48	0.42
1:J:124:VAL:HG22	1:J:504:LEU:HD11	2.01	0.42
1:J:262:LEU:HD13	1:J:273:VAL:HG11	2.01	0.42
1:K:339:GLU:HA	1:K:342:ILE:HB	2.01	0.42
1:L:115:ASP:CG	1:L:118:ARG:HH21	2.22	0.42
1:N:20:VAL:HG22	1:N:74:VAL:CG2	2.45	0.42
1:N:69:MET:HA	1:N:72:GLN:HG2	2.01	0.42
1:N:339:GLU:HA	1:N:342:ILE:HD12	2.00	0.42
1:N:514:MET:HA	1:N:517:THR:OG1	2.19	0.42
2:R:37:ARG:HH12	2:S:3:ILE:HD11	1.84	0.42
2:R:78:ILE:HD11	2:R:83:VAL:HG11	2.01	0.42
1:A:207:LYS:HD2	1:A:212:ALA:HB3	2.01	0.42
1:B:194:GLN:HA	1:B:331:THR:HA	2.00	0.42
1:C:169:VAL:HG13	1:C:170:GLY:O	2.18	0.42
1:E:205:ILE:HD13	1:E:211:GLY:HA2	2.02	0.42
1:E:220:ILE:HG13	1:E:248:LEU:HD23	2.01	0.42
1:F:308:GLU:H	1:F:311:LYS:HD3	1.84	0.42
1:G:62:LEU:O	1:G:68:ASN:HB2	2.18	0.42
1:G:162:ILE:HG23	1:G:400:LEU:HD12	2.01	0.42
1:G:455:VAL:HG13	1:G:460:GLU:HB2	2.00	0.42
1:H:138:CYS:O	1:H:407:VAL:HG22	2.19	0.42
1:H:199:TYR:CZ	1:H:327:LYS:HA	2.54	0.42
1:I:279:PRO:C	1:I:288:MET:HG3	2.40	0.42
1:I:299:THR:N	1:I:316:ASP:O	2.47	0.42

Continued on next page...

For Manuscript Review

Continued from previous page...

Atom-1	Atom-2	Interatomic distance (Å)	Clash overlap (Å)
1:I:327:LYS:HE2	1:I:327:LYS:HB3	1.82	0.42
1:K:246:PRO:HA	1:K:272:LYS:HB2	2.00	0.42
1:L:76:GLU:O	1:L:80:LYS:HG3	2.19	0.42
1:L:162:ILE:HG23	1:L:400:LEU:HD12	2.01	0.42
1:L:177:VAL:HG22	1:L:393:LYS:HE2	2.01	0.42
1:L:320:ALA:HA	1:L:336:VAL:H	1.85	0.42
1:M:415:GLY:HA3	1:M:488:MET:HE2	2.01	0.42
1:N:13:ARG:HA	1:N:514:MET:CE	2.49	0.42
1:N:32:GLY:HA2	6:N:601:ADP:O4'	2.19	0.42
1:N:40:LEU:HD22	1:N:59:GLU:HB3	2.02	0.42
1:N:451:LEU:O	1:N:455:VAL:HG23	2.19	0.42
2:P:3:ILE:HD13	2:P:78:ILE:HG21	2.00	0.42
2:T:5:PRO:HD3	2:T:42:ALA:HB1	2.01	0.42
1:B:82:ASN:HB2	1:B:89:THR:HG21	2.01	0.42
1:B:115:ASP:OD2	1:B:433:ASN:ND2	2.32	0.42
1:B:338:GLU:HG2	1:B:338:GLU:O	2.19	0.42
1:B:441:LYS:HB3	1:B:445:ARG:NH1	2.34	0.42
1:C:242:LYS:HE2	1:C:242:LYS:HB2	1.89	0.42
1:C:250:ILE:HD11	1:C:332:ILE:HD11	2.02	0.42
1:C:353:ILE:HG23	1:C:362:ARG:HB2	2.01	0.42
1:D:197:ARG:HD2	1:D:277:LYS:HB2	2.00	0.42
1:D:313:THR:O	1:D:317:LEU:HG	2.20	0.42
1:E:48:THR:HG22	1:E:390:LYS:NZ	2.35	0.42
1:E:199:TYR:HE1	1:E:212:ALA:HA	1.83	0.42
1:E:199:TYR:HA	1:E:276:VAL:HG12	2.01	0.42
1:E:207:LYS:HE2	1:E:212:ALA:HB3	2.00	0.42
1:F:283:ASP:OD1	1:F:284:ARG:N	2.52	0.42
1:G:122:LYS:NZ	1:G:430:ARG:O	2.41	0.42
1:J:158:VAL:HG13	1:J:396:VAL:HG22	1.99	0.42
1:K:30:THR:HA	1:K:35:GLY:HA3	2.02	0.42
1:K:429:LEU:HB3	1:K:440:ILE:HG21	2.01	0.42
1:L:339:GLU:HA	1:L:342:ILE:HD12	2.00	0.42
1:M:433:ASN:OD1	1:M:434:GLU:N	2.52	0.42
1:N:35:GLY:HA2	1:N:457:ASN:HB3	2.00	0.42
1:A:169:VAL:HG13	1:A:170:GLY:O	2.19	0.42
1:A:253:ASP:OD1	1:A:254:VAL:N	2.50	0.42
1:A:336:VAL:HG23	1:A:336:VAL:O	2.20	0.42
1:B:205:ILE:HD13	1:B:211:GLY:HA2	2.01	0.42
1:B:276:VAL:HG11	1:B:330:THR:OG1	2.19	0.42
1:B:313:THR:O	1:B:317:LEU:HG	2.19	0.42
3:B:601:ATP:O1G	7:B:2001:HOH:O	2.21	0.42

Continued on next page...

For Manuscript Review

Continued from previous page...

Atom-1	Atom-2	Interatomic distance (Å)	Clash overlap (Å)
1:C:135:SER:HB3	1:C:497:THR:HG21	2.00	0.42
1:C:260:ALA:HA	1:C:263:VAL:HG22	2.02	0.42
1:D:448:GLU:HB3	1:D:452:ARG:CZ	2.50	0.42
1:G:77:VAL:HG11	1:G:96:ALA:HB2	2.02	0.42
1:G:277:LYS:HB3	1:G:277:LYS:HE3	1.90	0.42
1:H:35:GLY:HA2	1:H:457:ASN:HB3	2.00	0.42
1:H:295:LEU:HD23	1:H:342:ILE:HD13	2.02	0.42
1:H:421:ARG:HH12	1:H:469:VAL:C	2.23	0.42
1:I:15:LYS:NZ	1:I:64:ASP:OD2	2.42	0.42
1:I:215:LEU:O	1:I:323:VAL:HG22	2.20	0.42
1:I:219:PHE:CE2	1:I:245:LYS:HD2	2.55	0.42
1:I:349:ILE:HG12	1:I:368:ARG:CZ	2.50	0.42
1:I:447:MET:O	1:I:450:PRO:HD2	2.19	0.42
1:J:12:ALA:O	1:J:16:MET:HG2	2.19	0.42
1:J:14:VAL:O	1:J:18:ARG:HG3	2.20	0.42
1:J:31:LEU:HB3	1:J:453:GLN:HG3	2.01	0.42
1:J:406:ALA:HB2	1:J:496:PRO:HG3	2.01	0.42
1:K:46:ALA:HA	1:K:47:PRO:HD3	1.78	0.42
1:K:320:ALA:HA	1:K:336:VAL:H	1.84	0.42
1:L:279:PRO:O	1:L:285:ARG:HA	2.19	0.42
1:L:390:LYS:HA	1:L:390:LYS:HD2	1.74	0.42
1:L:452:ARG:HH12	1:L:463:SER:HA	1.83	0.42
1:L:520:MET:HE2	1:L:520:MET:HB3	1.80	0.42
1:M:62:LEU:HB2	1:M:68:ASN:HB2	2.02	0.42
1:M:168:LYS:HG2	1:M:189:VAL:HG13	2.02	0.42
1:M:185:ASP:OD2	1:M:392:LYS:HE3	2.19	0.42
1:N:319:GLN:HB2	1:N:336:VAL:HB	2.01	0.42
2:O:47:ARG:NH2	2:O:88:GLU:HB3	2.35	0.42
2:P:8:ASP:O	2:P:57:LEU:HD21	2.19	0.42
2:R:4:ARG:HH12	2:R:6:LEU:HD23	1.85	0.42
1:A:513:LEU:HB3	1:B:49:ILE:HD13	2.01	0.42
1:B:124:VAL:HG22	1:B:504:LEU:HD11	2.01	0.42
1:B:250:ILE:HD11	1:B:332:ILE:HD11	2.00	0.42
1:D:455:VAL:CG1	1:D:460:GLU:HB2	2.49	0.42
1:E:440:ILE:O	1:E:444:LEU:HG	2.20	0.42
1:G:448:GLU:OE1	1:G:470:LYS:NZ	2.53	0.42
1:I:220:ILE:HG12	1:I:222:LEU:HD21	2.02	0.42
1:I:356:ALA:HB3	1:I:362:ARG:NH2	2.35	0.42
1:J:46:ALA:HA	1:J:47:PRO:HD3	1.90	0.42
1:K:9:GLY:N	1:K:518:GLU:O	2.39	0.42
1:K:220:ILE:HG12	1:K:222:LEU:HD21	2.01	0.42

Continued on next page...

For Manuscript Review

Continued from previous page...

Atom-1	Atom-2	Interatomic distance (Å)	Clash overlap (Å)
1:K:230:ILE:O	1:K:234:LEU:N	2.52	0.42
1:K:451:LEU:HD11	1:K:469:VAL:HG21	2.02	0.42
1:L:175:ILE:HG21	1:L:400:LEU:HD11	2.01	0.42
1:L:230:ILE:O	1:L:234:LEU:HG	2.18	0.42
1:M:102:GLU:HB3	1:M:442:VAL:HG22	2.02	0.42
1:M:452:ARG:HH12	1:M:463:SER:HA	1.84	0.42
1:N:117:LYS:HD2	1:N:512:GLY:O	2.19	0.42
1:N:174:VAL:CG1	1:N:376:VAL:HG12	2.50	0.42
1:N:262:LEU:HD13	1:N:273:VAL:HG11	2.02	0.42
1:N:276:VAL:HG12	1:N:277:LYS:O	2.19	0.42
2:O:27:LEU:HB3	2:O:31:ALA:HB3	2.01	0.42
1:B:178:GLU:O	1:B:381:VAL:N	2.51	0.42
1:B:349:ILE:HG21	1:B:369:VAL:HG23	2.02	0.42
1:D:443:ALA:O	1:D:447:MET:HG2	2.20	0.42
1:F:323:VAL:HG22	1:F:332:ILE:HA	2.01	0.42
1:I:353:ILE:HA	1:I:362:ARG:HH12	1.84	0.42
1:J:34:LYS:HB2	1:J:458:CYS:SG	2.59	0.42
1:J:52:ASP:O	1:J:56:VAL:HG23	2.20	0.42
1:J:132:LYS:NZ	1:J:409:GLU:OE2	2.42	0.42
1:J:360:TYR:CE1	1:J:364:LYS:HE3	2.55	0.42
1:K:14:VAL:O	1:K:18:ARG:HG3	2.19	0.42
1:K:140:ASP:OD1	1:K:141:SER:N	2.52	0.42
1:K:146:GLN:HB2	1:K:494:LEU:HD12	2.01	0.42
1:L:195:PHE:CD2	1:L:279:PRO:HB3	2.54	0.42
1:L:204:PHE:CD1	1:L:266:THR:HG21	2.55	0.42
1:L:219:PHE:CD2	1:L:245:LYS:HD2	2.55	0.42
1:M:150:ILE:HD12	6:M:601:ADP:N7	2.35	0.42
1:N:438:VAL:O	1:N:442:VAL:HG23	2.20	0.42
1:N:460:GLU:HG3	1:N:478:TYR:OH	2.19	0.42
2:R:59:VAL:HG22	2:R:88:GLU:HG3	2.01	0.42
1:B:23:LEU:HD22	1:B:74:VAL:HG13	2.02	0.42
1:C:262:LEU:HD13	1:C:273:VAL:HG11	2.01	0.42
1:C:386:GLU:O	1:C:390:LYS:HG3	2.20	0.42
1:D:227:ILE:HG22	1:D:255:GLU:OE1	2.19	0.42
1:D:277:LYS:HB3	1:D:277:LYS:HE3	1.90	0.42
1:E:112:ASN:ND2	7:E:722:HOH:O	2.49	0.42
1:E:455:VAL:HG13	1:E:460:GLU:HB2	2.02	0.42
1:F:465:VAL:HA	1:F:485:TYR:OH	2.20	0.42
1:G:264:VAL:HA	1:G:267:MET:SD	2.60	0.42
1:H:248:LEU:HD21	1:H:250:ILE:HD11	2.02	0.42
1:J:104:LEU:HG	1:J:514:MET:HE1	2.02	0.42

Continued on next page...

For Manuscript Review

Continued from previous page...

Atom-1	Atom-2	Interatomic distance (Å)	Clash overlap (Å)
1:J:417:VAL:HG12	1:J:451:LEU:HD12	2.02	0.42
1:K:319:GLN:HB2	1:K:336:VAL:HB	2.02	0.42
1:K:346:VAL:O	1:K:350:ARG:HG2	2.20	0.42
1:L:102:GLU:HB3	1:L:442:VAL:HG22	2.02	0.42
1:M:13:ARG:HA	1:M:514:MET:CE	2.50	0.42
1:M:420:ILE:HG23	1:M:470:LYS:HG2	2.00	0.42
1:M:434:GLU:HA	1:M:437:ASN:ND2	2.34	0.42
1:N:150:ILE:HD12	6:N:601:ADP:N7	2.35	0.42
1:N:217:SER:HA	1:N:320:ALA:O	2.19	0.42
1:N:220:ILE:HA	1:N:248:LEU:HB3	2.01	0.42
1:N:511:ALA:O	1:N:515:ILE:HG13	2.19	0.42
2:S:73:VAL:HG11	2:S:84:LEU:HD23	2.02	0.42
1:A:95:LEU:O	1:A:99:ILE:HG13	2.20	0.42
1:A:441:LYS:HB3	1:A:445:ARG:NH1	2.34	0.42
1:B:33:PRO:HG3	3:B:601:ATP:C6	2.55	0.42
1:B:207:LYS:HE2	1:B:212:ALA:HB3	2.02	0.42
1:B:283:ASP:OD1	1:B:284:ARG:N	2.53	0.42
1:D:115:ASP:O	1:D:436:GLN:HG2	2.20	0.42
1:E:134:LEU:HD23	1:E:418:ALA:HB1	2.01	0.42
1:E:230:ILE:HD11	1:E:258:ALA:HA	2.02	0.42
3:E:601:ATP:H8	3:E:601:ATP:H5'2	1.85	0.42
1:F:218:PRO:HG2	1:F:323:VAL:HG23	2.02	0.42
1:G:232:GLU:HG2	1:G:310:GLU:OE2	2.20	0.42
1:H:33:PRO:HD3	6:H:601:ADP:N9	2.35	0.42
1:H:368:ARG:O	1:H:372:LEU:HD23	2.19	0.42
1:I:319:GLN:HB2	1:I:336:VAL:HB	2.01	0.42
1:I:364:LYS:HD3	1:I:364:LYS:HA	1.69	0.42
1:J:214:GLU:OE1	1:J:214:GLU:N	2.52	0.42
1:J:345:ARG:O	1:J:349:ILE:HG13	2.20	0.42
1:J:476:TYR:HA	1:J:487:ASN:HA	2.01	0.42
1:L:68:ASN:O	1:L:72:GLN:HG2	2.20	0.42
1:L:168:LYS:HG2	1:L:189:VAL:HG13	2.02	0.42
1:L:443:ALA:O	1:L:447:MET:HG2	2.20	0.42
1:M:39:VAL:HG22	1:M:49:ILE:HG12	2.01	0.42
1:M:137:PRO:HA	1:M:410:GLY:HA2	2.00	0.42
1:M:144:ILE:HG23	1:M:403:THR:HB	2.02	0.42
1:M:489:ILE:HG23	1:M:494:LEU:HD21	2.00	0.42
1:N:124:VAL:HG11	1:N:508:ALA:CB	2.49	0.42
1:N:452:ARG:HH12	1:N:463:SER:HA	1.85	0.42
2:O:40:VAL:HG22	2:O:63:ASP:O	2.20	0.42
2:R:92:LEU:HB3	2:S:85:ILE:HG21	2.02	0.42

Continued on next page...

For Manuscript Review

Continued from previous page...

Atom-1	Atom-2	Interatomic distance (Å)	Clash overlap (Å)
1:A:69:MET:HB2	1:B:47:PRO:CG	2.50	0.42
1:A:175:ILE:HB	1:A:404:ARG:NH1	2.33	0.42
1:A:196:ASP:HA	1:A:329:THR:HA	2.01	0.42
1:B:243:ALA:HB2	1:B:314:LEU:HD21	2.02	0.42
1:B:447:MET:HA	1:B:447:MET:HE2	2.02	0.42
1:B:523:ASP:OD1	1:B:524:LEU:N	2.53	0.42
1:C:128:VAL:HG21	1:C:505:GLN:HG3	2.02	0.42
1:C:452:ARG:HH21	1:C:470:LYS:NZ	2.18	0.42
1:D:205:ILE:HG23	1:D:212:ALA:O	2.20	0.42
1:E:387:VAL:HA	1:E:390:LYS:HE2	2.02	0.42
1:F:452:ARG:HH21	1:F:470:LYS:HZ1	1.66	0.42
1:G:194:GLN:HA	1:G:331:THR:HA	2.02	0.42
1:G:199:TYR:CZ	1:G:205:ILE:HD11	2.55	0.42
1:I:432:GLN:HB2	1:I:436:GLN:NE2	2.35	0.42
1:J:233:MET:HE1	1:J:247:LEU:HD21	2.02	0.42
1:K:33:PRO:HD3	6:K:601:ADP:C4	2.55	0.42
1:K:417:VAL:HG12	1:K:451:LEU:CD1	2.50	0.42
1:K:478:TYR:HA	1:K:485:TYR:HA	2.00	0.42
1:L:432:GLN:HB2	1:L:436:GLN:OE1	2.20	0.42
1:M:15:LYS:NZ	1:M:64:ASP:OD2	2.35	0.42
1:M:216:GLU:HG2	1:M:322:ARG:HD2	2.01	0.42
1:M:320:ALA:HA	1:M:336:VAL:H	1.85	0.42
1:N:248:LEU:HD22	1:N:323:VAL:HG21	2.01	0.42
2:O:12:VAL:HG22	2:O:84:LEU:HB2	2.02	0.42
2:O:40:VAL:HG11	2:O:59:VAL:HG11	2.02	0.42
2:Q:25:ILE:H	2:Q:25:ILE:HD12	1.83	0.42
2:U:12:VAL:O	2:U:84:LEU:HB2	2.20	0.42
1:A:31:LEU:O	1:A:457:ASN:ND2	2.24	0.42
1:A:215:LEU:HB3	1:A:246:PRO:HB2	2.02	0.42
1:C:33:PRO:HD3	3:C:601:ATP:C8	2.54	0.42
1:C:162:ILE:HG23	1:C:400:LEU:HD12	2.01	0.42
1:D:48:THR:HG22	1:D:390:LYS:NZ	2.35	0.42
1:F:168:LYS:HB3	1:F:189:VAL:HB	2.02	0.42
1:F:250:ILE:HG23	1:F:278:ALA:HA	2.02	0.42
1:G:261:THR:HA	2:U:28:THR:OG1	2.20	0.42
1:G:524:LEU:HD23	1:G:524:LEU:HA	1.92	0.42
1:H:217:SER:HA	1:H:320:ALA:O	2.20	0.42
1:I:32:GLY:HA3	1:I:454:ILE:HG23	2.01	0.42
1:I:127:ALA:HB3	1:I:504:LEU:HD21	2.02	0.42
1:J:217:SER:HA	1:J:320:ALA:O	2.19	0.42
1:J:452:ARG:HH12	1:J:463:SER:HA	1.85	0.42

Continued on next page...

For Manuscript Review

Continued from previous page...

Atom-1	Atom-2	Interatomic distance (Å)	Clash overlap (Å)
1:K:221:LEU:N	1:K:248:LEU:O	2.31	0.42
1:K:223:ALA:O	1:K:251:ALA:HA	2.19	0.42
1:M:85:ALA:CB	1:M:499:VAL:HA	2.40	0.42
1:M:353:ILE:HD11	1:M:369:VAL:HG11	2.02	0.42
1:N:16:MET:HE1	1:N:66:PHE:O	2.20	0.42
2:R:59:VAL:HG12	2:R:94:ILE:HD11	2.01	0.42
1:A:240:VAL:HG21	1:A:247:LEU:CD1	2.49	0.41
1:A:261:THR:HG23	2:O:26:VAL:O	2.20	0.41
1:B:162:ILE:HG12	1:B:400:LEU:HD13	2.01	0.41
1:B:231:ARG:HH11	1:B:234:LEU:HD11	1.85	0.41
1:C:34:LYS:HA	1:C:36:ARG:HH22	1.84	0.41
1:D:143:ALA:O	1:D:147:VAL:HG23	2.20	0.41
1:D:186:GLU:O	1:D:380:LYS:N	2.30	0.41
1:D:448:GLU:O	1:D:452:ARG:HD3	2.20	0.41
1:D:479:ASN:CG	1:D:493:ILE:HD11	2.40	0.41
1:E:158:VAL:HG13	1:E:396:VAL:HG13	2.02	0.41
1:E:197:ARG:HD2	1:E:277:LYS:HB2	2.02	0.41
1:E:206:ASN:ND2	1:E:214:GLU:O	2.53	0.41
1:F:82:ASN:HB2	1:F:89:THR:HG21	2.02	0.41
1:F:222:LEU:HD21	1:F:292:ILE:HG22	2.00	0.41
1:F:455:VAL:HG13	1:F:460:GLU:HB2	2.01	0.41
1:F:455:VAL:CG1	1:F:460:GLU:HB2	2.50	0.41
1:G:441:LYS:HB3	1:G:445:ARG:NH1	2.35	0.41
1:G:455:VAL:HG21	1:G:465:VAL:HG11	2.02	0.41
1:H:2:ALA:O	1:H:4:LYS:HG2	2.20	0.41
1:H:100:ILE:HG12	1:H:511:ALA:HA	2.02	0.41
1:H:132:LYS:NZ	1:H:409:GLU:OE2	2.43	0.41
1:H:250:ILE:HA	1:H:276:VAL:O	2.20	0.41
1:I:15:LYS:HD3	1:I:18:ARG:NH2	2.35	0.41
1:I:406:ALA:HB2	1:I:496:PRO:HG3	2.02	0.41
1:J:34:LYS:HE3	1:J:483:GLU:OE1	2.20	0.41
1:J:199:TYR:CE1	1:J:327:LYS:HA	2.54	0.41
1:J:343:GLN:HA	1:J:346:VAL:HB	2.02	0.41
1:K:195:PHE:CD2	1:K:279:PRO:HB3	2.55	0.41
1:K:199:TYR:CZ	1:K:327:LYS:HA	2.55	0.41
1:K:287:ALA:HB1	1:K:368:ARG:CZ	2.50	0.41
1:K:447:MET:O	1:K:450:PRO:HD2	2.21	0.41
1:L:179:ASP:OD1	1:L:389:MET:HE2	2.20	0.41
1:L:301:ILE:HG21	1:L:309:LEU:HD23	2.02	0.41
1:M:16:MET:HG3	1:M:514:MET:SD	2.60	0.41
1:M:20:VAL:HG22	1:M:74:VAL:CG2	2.50	0.41

Continued on next page...

For Manuscript Review

Continued from previous page...

Atom-1	Atom-2	Interatomic distance (Å)	Clash overlap (Å)
1:M:162:ILE:HG23	1:M:400:LEU:HD12	2.01	0.41
1:M:217:SER:HA	1:M:320:ALA:O	2.20	0.41
1:M:231:ARG:O	1:M:231:ARG:NH1	2.43	0.41
1:M:432:GLN:HB2	1:M:436:GLN:NE2	2.34	0.41
2:S:39:GLU:N	2:S:39:GLU:OE1	2.53	0.41
2:T:25:ILE:H	2:T:25:ILE:HD12	1.85	0.41
1:A:20:VAL:HG22	1:A:74:VAL:HB	2.02	0.41
1:A:295:LEU:HD21	1:A:335:GLY:H	1.86	0.41
1:A:323:VAL:HG22	1:A:332:ILE:HA	2.02	0.41
1:B:149:THR:HG22	1:B:154:SER:HA	2.01	0.41
1:B:323:VAL:HG22	1:B:332:ILE:HG12	2.01	0.41
1:C:36:ARG:NE	1:C:36:ARG:HA	2.35	0.41
1:C:207:LYS:HD2	1:C:212:ALA:HB3	2.01	0.41
1:C:217:SER:HA	1:C:320:ALA:O	2.20	0.41
1:C:465:VAL:HA	1:C:485:TYR:OH	2.20	0.41
1:E:205:ILE:HG23	1:E:212:ALA:O	2.20	0.41
1:E:217:SER:HA	1:E:320:ALA:O	2.20	0.41
1:F:150:ILE:CG1	1:F:493:ILE:HA	2.47	0.41
1:F:276:VAL:HG11	1:F:330:THR:OG1	2.20	0.41
1:F:458:CYS:HB3	1:F:483:GLU:OE2	2.20	0.41
1:F:519:CYS:SG	1:F:520:MET:N	2.93	0.41
1:H:279:PRO:C	1:H:288:MET:HG3	2.39	0.41
1:I:106:ALA:O	1:I:111:MET:HG2	2.19	0.41
1:J:479:ASN:HB2	1:J:491:MET:CE	2.50	0.41
1:K:16:MET:HE1	1:K:69:MET:HB3	2.02	0.41
1:K:248:LEU:HB2	1:K:323:VAL:HG21	2.03	0.41
1:M:7:LYS:HE3	1:M:15:LYS:HE3	2.02	0.41
1:N:289:LEU:HD22	1:N:300:VAL:HG13	2.01	0.41
1:N:381:VAL:HG23	1:N:389:MET:SD	2.60	0.41
2:S:25:ILE:H	2:S:25:ILE:HD12	1.85	0.41
1:A:305:ILE:O	1:B:264:VAL:HG22	2.20	0.41
1:A:349:ILE:HG23	1:A:365:LEU:CD1	2.47	0.41
1:B:452:ARG:HH21	1:B:470:LYS:NZ	2.17	0.41
1:D:123:ALA:HB2	1:D:440:ILE:HG23	2.02	0.41
1:D:239:ALA:HB1	1:D:314:LEU:HD11	2.02	0.41
1:E:345:ARG:O	1:E:349:ILE:HG13	2.20	0.41
1:E:466:ALA:O	1:E:470:LYS:HG3	2.20	0.41
1:F:108:ALA:HB1	1:J:109:ALA:HB1	2.03	0.41
1:F:219:PHE:CD2	1:F:240:VAL:HG22	2.55	0.41
1:F:339:GLU:HB3	1:F:343:GLN:HE22	1.84	0.41
1:F:421:ARG:HH12	1:F:470:LYS:HA	1.84	0.41

Continued on next page...

For Manuscript Review

Continued from previous page...

Atom-1	Atom-2	Interatomic distance (Å)	Clash overlap (Å)
1:G:31:LEU:HD23	1:G:453:GLN:HG3	2.02	0.41
1:H:20:VAL:HG22	1:H:74:VAL:CG2	2.48	0.41
1:I:117:LYS:HD2	1:I:512:GLY:O	2.20	0.41
1:J:414:GLY:O	1:J:417:VAL:HG22	2.20	0.41
1:K:217:SER:HA	1:K:320:ALA:O	2.21	0.41
1:L:31:LEU:HD23	1:L:453:GLN:HB3	2.03	0.41
1:L:230:ILE:HG12	1:L:261:THR:HG21	2.02	0.41
1:M:149:THR:HG22	1:M:154:SER:HA	2.02	0.41
1:M:284:ARG:NE	1:M:364:LYS:HB3	2.35	0.41
1:M:339:GLU:HA	1:M:342:ILE:HB	2.01	0.41
1:N:85:ALA:CB	1:N:499:VAL:HA	2.43	0.41
1:N:187:LEU:HB3	1:N:379:ILE:HG12	2.02	0.41
1:A:455:VAL:CG1	1:A:460:GLU:HB2	2.50	0.41
1:B:178:GLU:HA	1:B:393:LYS:HE2	2.01	0.41
1:B:443:ALA:O	1:B:447:MET:HG2	2.21	0.41
1:C:194:GLN:HA	1:C:331:THR:HA	2.03	0.41
1:C:511:ALA:O	1:C:515:ILE:HG12	2.20	0.41
1:D:222:LEU:HD22	1:D:293:ALA:HB2	2.02	0.41
1:F:28:LYS:HD3	1:F:453:GLN:OE1	2.21	0.41
1:F:194:GLN:HA	1:F:331:THR:HA	2.02	0.41
1:F:277:LYS:HB3	1:F:277:LYS:HE3	1.90	0.41
1:F:399:ALA:O	1:F:403:THR:OG1	2.28	0.41
1:F:510:VAL:HG23	1:G:385:THR:HG21	2.01	0.41
1:G:381:VAL:HG13	1:G:392:LYS:HE3	2.03	0.41
1:H:116:LEU:HD23	1:H:435:ASP:O	2.21	0.41
1:I:38:VAL:O	1:I:50:THR:N	2.47	0.41
1:J:150:ILE:HD12	6:J:601:ADP:N7	2.34	0.41
1:J:339:GLU:HA	1:J:342:ILE:HB	2.02	0.41
1:K:124:VAL:HG11	1:K:508:ALA:CB	2.50	0.41
1:L:451:LEU:O	1:L:455:VAL:HG23	2.20	0.41
1:M:186:GLU:N	1:M:380:LYS:O	2.53	0.41
2:P:66:ILE:HG21	2:Q:76:GLU:HG2	2.03	0.41
2:P:77:LYS:HE2	2:P:77:LYS:HB2	1.89	0.41
2:R:12:VAL:HG12	2:R:40:VAL:HG12	2.01	0.41
2:T:73:VAL:HG22	2:T:86:MET:SD	2.61	0.41
1:A:350:ARG:O	1:A:354:GLU:HG2	2.20	0.41
1:C:472:GLY:HA3	1:C:476:TYR:CD2	2.56	0.41
1:D:102:GLU:HB2	1:D:442:VAL:HG13	2.02	0.41
1:D:149:THR:HG22	1:D:154:SER:HA	2.01	0.41
1:E:15:LYS:HE2	1:E:67:GLU:HG3	2.01	0.41
1:E:54:VAL:HG11	1:E:82:ASN:HB2	2.02	0.41

Continued on next page...

For Manuscript Review

Continued from previous page...

Atom-1	Atom-2	Interatomic distance (Å)	Clash overlap (Å)
1:E:178:GLU:N	1:E:379:ILE:O	2.45	0.41
1:E:219:PHE:HB2	1:E:247:LEU:HA	2.02	0.41
1:E:338:GLU:H	1:E:338:GLU:CD	2.23	0.41
1:F:475:ASN:O	1:F:488:MET:N	2.52	0.41
1:G:13:ARG:HD2	1:G:104:LEU:HD22	2.01	0.41
1:H:17:LEU:HD12	1:H:17:LEU:HA	1.83	0.41
1:H:215:LEU:HB3	1:H:218:PRO:HB3	2.02	0.41
1:H:216:GLU:HG2	1:H:322:ARG:HD2	2.01	0.41
1:H:220:ILE:HA	1:H:248:LEU:HB3	2.03	0.41
1:J:169:VAL:CG2	1:J:377:ALA:HB2	2.51	0.41
1:J:443:ALA:O	1:J:447:MET:HG2	2.20	0.41
1:J:493:ILE:HG12	6:J:601:ADP:N6	2.35	0.41
1:J:516:THR:OG1	1:K:37:ASN:OD1	2.18	0.41
1:K:150:ILE:HD12	6:K:601:ADP:N7	2.36	0.41
1:K:294:THR:HG21	1:K:345:ARG:HB2	2.01	0.41
1:K:465:VAL:HA	1:K:485:TYR:OH	2.20	0.41
1:K:522:THR:HA	1:L:41:ASP:HB3	2.02	0.41
1:L:289:LEU:HD22	1:L:300:VAL:HG13	2.03	0.41
1:M:104:LEU:HD21	1:M:514:MET:HG2	2.01	0.41
1:N:15:LYS:HB3	1:N:66:PHE:HB2	2.03	0.41
2:O:6:LEU:HB3	2:O:7:HIS:CD2	2.55	0.41
2:U:37:ARG:HA	2:U:65:VAL:O	2.20	0.41
1:A:92:ALA:HB2	1:A:503:ALA:HB1	2.03	0.41
1:B:206:ASN:ND2	1:B:214:GLU:O	2.53	0.41
1:C:34:LYS:HB2	1:C:458:CYS:SG	2.60	0.41
1:D:158:VAL:HG22	1:D:396:VAL:HG22	2.01	0.41
1:D:421:ARG:NH2	1:D:469:VAL:O	2.44	0.41
1:E:455:VAL:CG1	1:E:460:GLU:HB2	2.51	0.41
1:E:472:GLY:HA3	1:E:476:TYR:CD2	2.56	0.41
1:G:134:LEU:HD23	1:G:418:ALA:HB1	2.01	0.41
1:H:301:ILE:HA	1:H:307:MET:HE3	2.01	0.41
1:I:124:VAL:HG11	1:I:508:ALA:CB	2.51	0.41
1:I:152:ALA:HB3	1:I:155:ASP:HB2	2.03	0.41
1:I:421:ARG:HH12	1:I:469:VAL:C	2.24	0.41
1:J:105:LYS:HA	1:J:105:LYS:HD2	1.69	0.41
1:K:489:ILE:HA	1:K:494:LEU:HD21	2.03	0.41
1:N:144:ILE:HG23	1:N:403:THR:HB	2.02	0.41
1:N:185:ASP:HA	1:N:380:LYS:O	2.21	0.41
1:N:284:ARG:NH1	1:N:364:LYS:HD2	2.36	0.41
1:N:455:VAL:HG21	1:N:465:VAL:HG11	2.01	0.41
2:R:95:VAL:HG13	2:S:3:ILE:HD11	2.02	0.41

Continued on next page...

For Manuscript Review

Continued from previous page...

Atom-1	Atom-2	Interatomic distance (Å)	Clash overlap (Å)
1:A:420:ILE:HG21	1:A:470:LYS:HG2	2.01	0.41
1:C:257:GLU:HG3	2:Q:30:SER:H	1.85	0.41
1:C:440:ILE:O	1:C:444:LEU:HG	2.21	0.41
1:D:138:CYS:HB3	1:D:406:ALA:HB1	2.03	0.41
1:D:262:LEU:O	1:D:266:THR:HG23	2.20	0.41
1:E:33:PRO:HG3	3:E:601:ATP:C6	2.55	0.41
1:F:32:GLY:HA2	3:F:601:ATP:O4'	2.20	0.41
1:F:452:ARG:NH1	7:F:708:HOH:O	2.29	0.41
1:G:33:PRO:HD3	3:G:601:ATP:C8	2.56	0.41
1:G:106:ALA:O	1:G:111:MET:HG2	2.21	0.41
1:G:287:ALA:HA	1:G:345:ARG:NH2	2.34	0.41
1:G:348:GLN:HA	1:G:351:GLN:OE1	2.20	0.41
1:G:370:ALA:HB1	1:G:375:GLY:O	2.21	0.41
1:H:140:ASP:O	1:H:144:ILE:HD12	2.21	0.41
1:H:351:GLN:HA	1:H:354:GLU:OE2	2.20	0.41
1:I:415:GLY:HA2	6:I:601:ADP:N3	2.35	0.41
1:J:124:VAL:HG13	1:J:504:LEU:HG	2.03	0.41
1:J:250:ILE:HG12	1:J:276:VAL:HB	2.03	0.41
1:J:480:ALA:H	6:J:601:ADP:H2	1.69	0.41
1:J:526:LYS:HA	1:J:526:LYS:HD2	1.78	0.41
1:K:123:ALA:HB3	1:K:443:ALA:HB3	2.01	0.41
1:L:199:TYR:CZ	1:L:327:LYS:HA	2.56	0.41
1:L:213:VAL:HB	1:L:325:ILE:HG12	2.03	0.41
1:M:31:LEU:HD23	1:M:453:GLN:HB3	2.03	0.41
1:M:68:ASN:O	1:M:72:GLN:HG2	2.20	0.41
1:M:433:ASN:HB3	1:M:436:GLN:HG3	2.02	0.41
1:N:15:LYS:NZ	1:N:64:ASP:OD2	2.35	0.41
1:N:345:ARG:HD2	1:N:348:GLN:OE1	2.20	0.41
2:P:5:PRO:HD3	2:P:42:ALA:HB1	2.03	0.41
2:R:12:VAL:CG2	2:R:84:LEU:HB2	2.49	0.41
2:R:37:ARG:NH2	2:S:78:ILE:HG22	2.35	0.41
2:R:38:GLY:HA3	2:R:67:PHE:CE1	2.50	0.41
2:T:15:LYS:HE2	2:T:39:GLU:HB2	2.03	0.41
2:T:17:VAL:HG13	2:T:33:ALA:O	2.20	0.41
2:T:74:LYS:O	2:T:85:ILE:N	2.54	0.41
2:U:8:ASP:C	2:U:57:LEU:HD21	2.41	0.41
2:U:57:LEU:O	2:U:60:LYS:NZ	2.36	0.41
1:A:34:LYS:HB2	1:A:458:CYS:SG	2.61	0.41
1:A:136:VAL:HA	1:A:137:PRO:HD3	1.95	0.41
1:A:213:VAL:O	1:A:325:ILE:N	2.32	0.41
1:A:248:LEU:HD12	1:A:274:ALA:O	2.21	0.41

Continued on next page...

For Manuscript Review

Continued from previous page...

Atom-1	Atom-2	Interatomic distance (Å)	Clash overlap (Å)
1:A:277:LYS:HB3	1:A:277:LYS:HE3	1.92	0.41
1:A:453:GLN:NE2	1:A:457:ASN:OD1	2.53	0.41
1:C:194:GLN:HG3	1:C:331:THR:HG22	2.02	0.41
1:C:219:PHE:HB3	1:C:317:LEU:HD13	2.02	0.41
1:C:323:VAL:HG22	1:C:332:ILE:HG12	2.03	0.41
1:C:517:THR:CG2	1:D:39:VAL:HG23	2.50	0.41
1:D:7:LYS:HB2	1:D:520:MET:HE2	2.02	0.41
1:D:230:ILE:HA	1:D:233:MET:HE2	2.03	0.41
1:D:239:ALA:HB1	1:D:314:LEU:CD1	2.50	0.41
1:E:308:GLU:HB2	1:E:311:LYS:HG3	2.03	0.41
1:E:397:GLU:O	1:E:401:HIS:ND1	2.54	0.41
1:F:270:ILE:HG21	2:T:25:ILE:HA	2.02	0.41
1:G:250:ILE:HG23	1:G:278:ALA:HA	2.02	0.41
1:H:124:VAL:HG11	1:H:508:ALA:CB	2.51	0.41
1:I:149:THR:HG21	1:I:156:GLU:OE2	2.20	0.41
1:J:219:PHE:CD2	1:J:245:LYS:HD2	2.56	0.41
1:J:347:ALA:O	1:J:351:GLN:HG3	2.20	0.41
1:J:519:CYS:HB3	1:K:38:VAL:HG22	2.02	0.41
1:K:149:THR:HG21	1:K:156:GLU:OE2	2.21	0.41
1:L:217:SER:HA	1:L:320:ALA:O	2.21	0.41
1:L:233:MET:HG2	1:L:262:LEU:HD21	2.02	0.41
1:L:263:VAL:HG12	1:L:267:MET:CE	2.51	0.41
1:L:274:ALA:HB1	1:L:325:ILE:CD1	2.51	0.41
1:L:431:GLY:HA3	1:L:436:GLN:HB3	2.02	0.41
1:L:447:MET:HE1	1:L:504:LEU:HD13	2.03	0.41
1:M:176:THR:O	1:M:379:ILE:N	2.45	0.41
1:M:289:LEU:HD12	1:M:289:LEU:HA	1.87	0.41
2:Q:10:VAL:HB	2:Q:86:MET:SD	2.61	0.41
2:S:75:SER:HA	2:S:83:VAL:O	2.21	0.41
2:U:40:VAL:HG22	2:U:63:ASP:O	2.20	0.41
1:A:54:VAL:HG11	1:A:82:ASN:HB2	2.02	0.41
1:A:144:ILE:HG21	1:A:163:ALA:HA	2.02	0.41
1:A:168:LYS:HB3	1:A:189:VAL:HG11	2.03	0.41
1:A:226:LYS:NZ	1:A:253:ASP:HB3	2.36	0.41
1:A:319:GLN:C	1:A:336:VAL:HG22	2.41	0.41
1:A:320:ALA:HA	1:A:336:VAL:H	1.86	0.41
1:C:111:MET:CE	1:C:435:ASP:HA	2.51	0.41
1:C:123:ALA:HB2	1:C:440:ILE:HG23	2.02	0.41
1:C:138:CYS:HB3	1:C:406:ALA:HB1	2.03	0.41
1:D:360:TYR:O	1:D:364:LYS:HG2	2.20	0.41
1:D:460:GLU:HB3	1:D:465:VAL:HG21	2.03	0.41

Continued on next page...

For Manuscript Review

Continued from previous page...

Atom-1	Atom-2	Interatomic distance (Å)	Clash overlap (Å)
1:E:115:ASP:CG	1:E:433:ASN:HD21	2.23	0.41
1:E:230:ILE:HA	1:E:233:MET:HE2	2.03	0.41
1:E:287:ALA:HA	1:E:345:ARG:HH21	1.86	0.41
1:F:102:GLU:CD	1:F:445:ARG:HE	2.24	0.41
1:F:112:ASN:ND2	1:F:115:ASP:OD2	2.46	0.41
1:F:261:THR:HG23	2:T:27:LEU:HA	2.03	0.41
1:F:264:VAL:HG11	2:T:28:THR:HG21	2.02	0.41
1:F:430:ARG:HH22	1:F:441:LYS:CE	2.34	0.41
1:G:82:ASN:HB2	1:G:89:THR:HG21	2.03	0.41
1:G:217:SER:N	1:G:218:PRO:HD3	2.36	0.41
1:G:323:VAL:HG12	1:G:325:ILE:HD11	2.02	0.41
1:G:421:ARG:NH2	1:G:469:VAL:O	2.48	0.41
1:H:18:ARG:HB2	1:H:67:GLU:HG2	2.01	0.41
1:H:149:THR:HG22	1:H:154:SER:HA	2.03	0.41
1:H:150:ILE:HD12	6:H:601:ADP:N7	2.36	0.41
1:H:429:LEU:HB3	1:H:440:ILE:HG21	2.02	0.41
1:I:4:LYS:HE2	1:J:59:GLU:OE2	2.21	0.41
1:I:33:PRO:HD2	1:I:454:ILE:HG23	2.01	0.41
1:I:141:SER:HB3	1:I:163:ALA:HB1	2.03	0.41
1:I:455:VAL:CG1	1:I:460:GLU:HB2	2.48	0.41
1:J:70:GLY:HA2	1:J:73:MET:CE	2.50	0.41
1:J:479:ASN:CG	1:J:493:ILE:HD11	2.42	0.41
1:K:5:ASP:HB3	1:K:522:THR:CG2	2.51	0.41
1:K:149:THR:HG22	1:K:154:SER:HA	2.03	0.41
1:K:511:ALA:O	1:K:515:ILE:HG13	2.20	0.41
1:L:180:GLY:H	1:L:389:MET:CE	2.33	0.41
1:L:262:LEU:HD22	1:L:273:VAL:HG11	2.03	0.41
1:L:291:ASP:HA	1:L:345:ARG:HG2	2.03	0.41
1:M:13:ARG:HA	1:M:514:MET:HE1	2.03	0.41
1:M:220:ILE:HG13	1:M:248:LEU:HD22	2.03	0.41
1:M:262:LEU:HD13	1:M:273:VAL:HG11	2.03	0.41
1:M:271:VAL:O	1:M:273:VAL:HG23	2.21	0.41
1:M:324:VAL:HB	1:M:331:THR:CG2	2.51	0.41
1:M:325:ILE:O	1:M:325:ILE:HG13	2.21	0.41
1:M:342:ILE:O	1:M:346:VAL:HG23	2.20	0.41
1:M:411:VAL:HG12	1:M:496:PRO:HA	2.02	0.41
1:M:414:GLY:HA3	1:M:493:ILE:HG22	2.02	0.41
2:O:39:GLU:OE1	2:O:64:ILE:HG13	2.20	0.41
2:P:12:VAL:CG2	2:P:86:MET:HE1	2.48	0.41
2:P:49:LEU:HD12	2:P:53:GLU:HG2	2.03	0.41
2:P:94:ILE:HD11	2:Q:4:ARG:NE	2.35	0.41

Continued on next page...

For Manuscript Review

Continued from previous page...

Atom-1	Atom-2	Interatomic distance (Å)	Clash overlap (Å)
2:Q:14:ARG:HG3	2:Q:67:PHE:CZ	2.55	0.41
2:U:5:PRO:HB3	2:U:9:ARG:HB2	2.02	0.41
1:A:28:LYS:HD3	1:A:453:GLN:OE1	2.21	0.41
1:A:342:ILE:HG23	1:A:372:LEU:HD12	2.03	0.41
1:B:136:VAL:HA	1:B:137:PRO:HD3	1.94	0.41
1:B:152:ALA:O	1:B:395:ARG:HD2	2.20	0.41
1:B:305:ILE:HD12	1:B:305:ILE:HA	1.97	0.41
1:C:149:THR:HG21	1:C:156:GLU:OE2	2.20	0.41
1:C:264:VAL:HG21	2:Q:28:THR:HG21	2.03	0.41
1:D:28:LYS:O	1:D:453:GLN:NE2	2.54	0.41
1:D:178:GLU:OE2	1:D:380:LYS:HD2	2.21	0.41
1:D:234:LEU:N	1:D:235:PRO:HD2	2.36	0.41
1:E:100:ILE:HA	1:E:515:ILE:HD11	2.03	0.41
1:E:360:TYR:O	1:E:364:LYS:HG2	2.21	0.41
1:F:189:VAL:HA	1:F:377:ALA:HA	2.02	0.41
1:F:429:LEU:HD23	1:F:440:ILE:HG12	2.03	0.41
1:G:420:ILE:HG23	1:G:470:LYS:HG2	2.03	0.41
1:H:2:ALA:O	1:I:61:GLU:HB2	2.21	0.41
1:H:220:ILE:HG12	1:H:222:LEU:HG	2.02	0.41
1:I:519:CYS:O	1:J:38:VAL:HA	2.21	0.41
1:K:513:LEU:HD23	1:K:513:LEU:HA	1.93	0.41
1:K:526:LYS:HD2	1:K:526:LYS:HA	1.79	0.41
1:M:292:ILE:HA	1:M:295:LEU:HD12	2.03	0.41
1:N:221:LEU:N	1:N:248:LEU:O	2.29	0.41
2:R:25:ILE:HD12	2:R:25:ILE:H	1.86	0.41
1:A:10:ASN:O	1:A:14:VAL:HG23	2.21	0.40
1:A:236:VAL:O	1:A:240:VAL:HG23	2.21	0.40
1:A:250:ILE:HG23	1:A:278:ALA:HA	2.03	0.40
1:A:432:GLN:HG2	1:A:436:GLN:HG3	2.02	0.40
1:B:149:THR:HA	1:B:155:ASP:O	2.21	0.40
1:B:158:VAL:HG22	1:B:396:VAL:HG22	2.03	0.40
1:C:225:LYS:N	1:C:252:GLU:OE1	2.53	0.40
1:C:228:SER:O	1:C:255:GLU:HB2	2.21	0.40
1:C:308:GLU:N	1:C:311:LYS:HD3	2.36	0.40
1:D:146:GLN:CD	1:D:492:GLY:HA2	2.41	0.40
1:D:338:GLU:CD	1:D:338:GLU:H	2.24	0.40
1:E:193:MET:HB2	1:E:332:ILE:HB	2.02	0.40
1:E:213:VAL:O	1:E:325:ILE:N	2.35	0.40
1:E:223:ALA:HB3	1:E:251:ALA:HB2	2.03	0.40
1:E:243:ALA:HB2	1:E:314:LEU:HD21	2.03	0.40
1:F:41:ASP:HA	1:F:47:PRO:HB3	2.02	0.40

Continued on next page...

For Manuscript Review

Continued from previous page...

Atom-1	Atom-2	Interatomic distance (Å)	Clash overlap (Å)
1:F:169:VAL:HG11	1:F:175:ILE:HG13	2.02	0.40
1:F:234:LEU:HD12	1:F:238:GLU:OE2	2.21	0.40
1:G:286:LYS:HA	1:G:286:LYS:HD3	1.81	0.40
1:G:479:ASN:HB3	1:G:482:THR:HB	2.02	0.40
1:H:352:GLN:HA	1:H:355:GLU:CG	2.51	0.40
1:J:81:ALA:HA	1:J:506:TYR:CD2	2.56	0.40
1:J:289:LEU:HD23	1:J:300:VAL:HG22	2.02	0.40
1:J:360:TYR:O	1:J:364:LYS:HG2	2.21	0.40
1:K:122:LYS:HZ3	1:K:431:GLY:HA2	1.86	0.40
1:K:194:GLN:HA	1:K:331:THR:HA	2.03	0.40
1:K:252:GLU:HG3	1:K:285:ARG:CZ	2.52	0.40
1:L:353:ILE:HG23	1:L:362:ARG:HD2	2.04	0.40
1:M:219:PHE:CE2	1:M:245:LYS:HD2	2.56	0.40
1:N:192:GLY:N	1:N:375:GLY:HA2	2.24	0.40
1:N:493:ILE:HD13	6:N:601:ADP:N1	2.36	0.40
2:P:40:VAL:HG11	2:P:59:VAL:HG11	2.04	0.40
2:T:5:PRO:HB3	2:T:85:ILE:HD11	2.03	0.40
2:U:4:ARG:NH2	2:U:45:ASN:HB3	2.36	0.40
2:U:49:LEU:N	2:U:53:GLU:O	2.35	0.40
1:A:116:LEU:HD21	1:A:438:VAL:HG12	2.03	0.40
1:A:383:ALA:HB1	1:A:388:GLU:HB3	2.03	0.40
1:A:479:ASN:O	1:A:483:GLU:N	2.54	0.40
1:B:138:CYS:HB3	1:B:406:ALA:HB1	2.04	0.40
1:B:419:LEU:HD23	1:B:419:LEU:HA	1.94	0.40
1:C:197:ARG:HD2	1:C:277:LYS:HB2	2.03	0.40
1:C:304:GLU:HG3	1:D:203:TYR:CE2	2.56	0.40
1:D:12:ALA:O	1:D:16:MET:HG2	2.21	0.40
1:D:33:PRO:HB2	1:D:481:ALA:HB2	2.03	0.40
1:D:255:GLU:HG2	1:D:257:GLU:H	1.86	0.40
1:D:452:ARG:NH1	1:D:466:ALA:HB1	2.36	0.40
1:E:430:ARG:HH12	1:E:441:LYS:HE2	1.86	0.40
1:G:115:ASP:O	1:G:436:GLN:HG2	2.20	0.40
1:H:289:LEU:HD23	1:H:300:VAL:HG22	2.04	0.40
1:I:42:LYS:H	1:I:47:PRO:HB3	1.85	0.40
1:I:140:ASP:O	1:I:144:ILE:HG13	2.20	0.40
1:I:247:LEU:O	1:I:273:VAL:HA	2.21	0.40
1:I:252:GLU:HG3	1:I:285:ARG:CZ	2.50	0.40
1:K:127:ALA:HB3	1:K:504:LEU:HD21	2.03	0.40
1:K:162:ILE:HD11	1:K:396:VAL:HG13	2.02	0.40
1:K:284:ARG:CZ	1:K:364:LYS:HD2	2.51	0.40
1:K:324:VAL:HB	1:K:331:THR:CG2	2.50	0.40

Continued on next page...

For Manuscript Review

Continued from previous page...

Atom-1	Atom-2	Interatomic distance (Å)	Clash overlap (Å)
1:K:349:ILE:HG12	1:K:368:ARG:NH2	2.36	0.40
1:L:33:PRO:HD3	6:L:601:ADP:N9	2.37	0.40
1:L:295:LEU:HA	1:L:342:ILE:HG12	2.03	0.40
1:N:197:ARG:HD2	1:N:277:LYS:HB3	2.03	0.40
1:N:421:ARG:HH12	1:N:469:VAL:C	2.25	0.40
2:P:37:ARG:HA	2:P:65:VAL:O	2.22	0.40
2:R:67:PHE:HA	2:R:91:ILE:HA	2.02	0.40
1:B:114:MET:HB3	1:B:118:ARG:CZ	2.51	0.40
1:B:292:ILE:O	1:B:296:THR:OG1	2.28	0.40
1:B:323:VAL:HA	1:B:331:THR:O	2.21	0.40
3:B:601:ATP:H8	3:B:601:ATP:H5'2	1.85	0.40
1:C:82:ASN:HB2	1:C:89:THR:HG21	2.03	0.40
1:C:420:ILE:CG2	1:C:470:LYS:HG2	2.51	0.40
1:D:20:VAL:HA	1:D:74:VAL:HG11	2.03	0.40
1:E:20:VAL:HA	1:E:74:VAL:HG11	2.03	0.40
1:E:31:LEU:HD23	1:E:453:GLN:HG3	2.04	0.40
1:E:194:GLN:HE21	1:E:329:THR:HG21	1.85	0.40
1:E:197:ARG:CZ	1:E:279:PRO:HA	2.51	0.40
1:E:203:TYR:CB	1:E:263:VAL:HB	2.51	0.40
1:E:495:ASP:OD2	3:E:601:ATP:O2'	2.33	0.40
1:E:513:LEU:HD11	1:F:388:GLU:HA	2.02	0.40
1:F:200:LEU:HD21	1:F:277:LYS:HG3	2.03	0.40
1:F:260:ALA:HA	1:F:263:VAL:HG22	2.02	0.40
1:I:30:THR:HA	1:I:35:GLY:HA3	2.03	0.40
1:I:81:ALA:HA	1:I:506:TYR:CD2	2.56	0.40
1:I:98:ALA:O	1:I:102:GLU:HG2	2.21	0.40
1:I:320:ALA:HA	1:I:335:GLY:HA2	2.04	0.40
1:J:248:LEU:HD21	1:J:250:ILE:HD11	2.03	0.40
1:K:479:ASN:ND2	1:K:493:ILE:HD11	2.37	0.40
1:L:348:GLN:HA	1:L:351:GLN:OE1	2.20	0.40
1:M:526:LYS:HD2	1:M:526:LYS:HA	1.80	0.40
1:N:7:LYS:HE3	1:N:15:LYS:HE3	2.03	0.40
1:N:30:THR:HA	1:N:35:GLY:HA3	2.04	0.40
1:N:102:GLU:HG3	1:N:445:ARG:NH1	2.36	0.40
1:N:246:PRO:HA	1:N:272:LYS:HB2	2.04	0.40
2:Q:40:VAL:HG22	2:Q:63:ASP:O	2.22	0.40
2:T:27:LEU:HB3	2:T:31:ALA:HB3	2.02	0.40
1:A:16:MET:HE3	1:A:69:MET:SD	2.61	0.40
1:A:136:VAL:O	1:A:411:VAL:N	2.32	0.40
1:A:479:ASN:N	1:A:484:GLU:O	2.53	0.40
1:B:115:ASP:O	1:B:436:GLN:HG2	2.21	0.40

Continued on next page...

For Manuscript Review

Continued from previous page...

Atom-1	Atom-2	Interatomic distance (Å)	Clash overlap (Å)
1:B:455:VAL:HG21	1:B:465:VAL:HG11	2.03	0.40
1:C:163:ALA:HA	1:C:166:MET:HE3	2.03	0.40
1:C:183:LEU:HA	1:C:383:ALA:N	2.37	0.40
1:C:392:LYS:O	1:C:396:VAL:HG23	2.21	0.40
1:C:417:VAL:O	1:C:421:ARG:HG2	2.21	0.40
1:D:82:ASN:HB2	1:D:89:THR:HG21	2.03	0.40
1:D:114:MET:CE	1:E:34:LYS:HG2	2.51	0.40
1:D:462:PRO:O	1:D:466:ALA:HB3	2.21	0.40
1:E:218:PRO:HG2	1:E:323:VAL:HG23	2.02	0.40
1:E:233:MET:O	1:E:237:LEU:HB2	2.21	0.40
1:E:420:ILE:CG2	1:E:470:LYS:HG2	2.51	0.40
1:F:161:LEU:HD12	1:F:161:LEU:HA	1.88	0.40
1:F:223:ALA:HB1	1:F:225:LYS:CG	2.51	0.40
1:F:413:ALA:HB1	1:F:488:MET:HG3	2.04	0.40
1:H:214:GLU:OE1	1:H:324:VAL:HG22	2.22	0.40
1:I:414:GLY:O	1:I:488:MET:HG3	2.21	0.40
1:J:116:LEU:HD23	1:J:435:ASP:O	2.21	0.40
1:K:230:ILE:HG12	1:K:261:THR:HG21	2.03	0.40
1:K:325:ILE:O	1:K:325:ILE:HG13	2.21	0.40
1:L:81:ALA:HA	1:L:506:TYR:CD2	2.56	0.40
1:L:468:THR:HB	1:L:485:TYR:CE2	2.56	0.40
1:M:351:GLN:HA	1:M:354:GLU:OE2	2.21	0.40
1:N:227:ILE:HG12	1:N:309:LEU:HD11	2.03	0.40
1:N:447:MET:O	1:N:450:PRO:HD2	2.21	0.40
2:O:10:VAL:HG11	2:O:40:VAL:HG12	2.03	0.40
2:P:47:ARG:NH2	2:P:88:GLU:HB3	2.36	0.40
2:Q:6:LEU:O	2:Q:9:ARG:HG3	2.22	0.40
2:T:11:ILE:N	2:T:42:ALA:O	2.44	0.40
1:A:147:VAL:HG22	1:A:494:LEU:HB2	2.03	0.40
1:A:513:LEU:HD12	1:B:387:VAL:HG23	2.03	0.40
1:B:145:ALA:HA	1:B:159:GLY:C	2.41	0.40
1:B:455:VAL:CG1	1:B:460:GLU:HB2	2.51	0.40
1:C:31:LEU:HD13	1:C:90:THR:HB	2.04	0.40
1:C:220:ILE:HG22	1:C:222:LEU:HG	2.03	0.40
1:C:455:VAL:CG1	1:C:460:GLU:HB2	2.51	0.40
1:D:154:SER:N	7:D:2018:HOH:O	2.54	0.40
1:D:235:PRO:HG3	1:D:310:GLU:O	2.22	0.40
1:D:249:ILE:HB	1:D:275:ALA:HA	2.04	0.40
1:D:297:GLY:HA2	1:D:338:GLU:OE2	2.21	0.40
1:F:342:ILE:HG23	1:F:372:LEU:CG	2.41	0.40
1:G:294:THR:HG22	1:G:341:ALA:HB1	2.04	0.40

Continued on next page...

For Manuscript Review

Continued from previous page...

Atom-1	Atom-2	Interatomic distance (Å)	Clash overlap (Å)
1:G:313:THR:OG1	1:G:315:GLU:OE1	2.35	0.40
1:H:39:VAL:HG22	1:H:49:ILE:HG12	2.04	0.40
1:H:102:GLU:HB2	1:H:442:VAL:HG13	2.04	0.40
1:H:262:LEU:HD22	1:H:273:VAL:HG21	2.03	0.40
1:I:287:ALA:HB1	1:I:368:ARG:NH1	2.37	0.40
1:J:230:ILE:HD13	1:J:261:THR:HB	2.03	0.40
1:K:204:PHE:CE2	1:K:275:ALA:HB3	2.56	0.40
1:L:20:VAL:HG13	1:L:74:VAL:HG21	2.04	0.40
1:L:519:CYS:O	1:M:38:VAL:HA	2.21	0.40
1:M:33:PRO:HG3	6:M:601:ADP:C6	2.56	0.40
1:M:115:ASP:CG	1:M:118:ARG:HH21	2.24	0.40
1:N:291:ASP:HA	1:N:345:ARG:HG2	2.02	0.40
1:N:295:LEU:HD21	1:N:372:LEU:HD13	2.04	0.40
2:Q:67:PHE:HA	2:Q:91:ILE:HA	2.03	0.40
2:S:14:ARG:HH21	2:S:67:PHE:HE2	1.69	0.40
2:S:60:LYS:N	2:S:63:ASP:OD2	2.35	0.40
2:T:25:ILE:HG22	2:T:27:LEU:HD22	2.04	0.40

There are no symmetry-related clashes.

5.3 Torsion angles (i)

5.3.1 Protein backbone (i)

In the following table, the Percentiles column shows the percent Ramachandran outliers of the chain as a percentile score with respect to all PDB entries followed by that with respect to all EM entries.

The Analysed column shows the number of residues for which the backbone conformation was analysed, and the total number of residues.

Mol	Chain	Analysed	Favoured	Allowed	Outliers	Percentiles	
1	A	522/547 (95%)	504 (97%)	18 (3%)	0	100	100
1	B	522/547 (95%)	509 (98%)	13 (2%)	0	100	100
1	C	522/547 (95%)	506 (97%)	16 (3%)	0	100	100
1	D	522/547 (95%)	510 (98%)	12 (2%)	0	100	100
1	E	522/547 (95%)	507 (97%)	15 (3%)	0	100	100
1	F	522/547 (95%)	507 (97%)	15 (3%)	0	100	100
1	G	522/547 (95%)	510 (98%)	12 (2%)	0	100	100

Continued on next page...

For Manuscript Review

Continued from previous page...

Mol	Chain	Analysed	Favoured	Allowed	Outliers	Percentiles	
1	H	523/547 (96%)	500 (96%)	23 (4%)	0	100	100
1	I	523/547 (96%)	500 (96%)	23 (4%)	0	100	100
1	J	523/547 (96%)	500 (96%)	23 (4%)	0	100	100
1	K	523/547 (96%)	500 (96%)	23 (4%)	0	100	100
1	L	523/547 (96%)	499 (95%)	24 (5%)	0	100	100
1	M	523/547 (96%)	504 (96%)	19 (4%)	0	100	100
1	N	523/547 (96%)	502 (96%)	21 (4%)	0	100	100
2	O	93/97 (96%)	87 (94%)	6 (6%)	0	100	100
2	P	93/97 (96%)	88 (95%)	5 (5%)	0	100	100
2	Q	93/97 (96%)	89 (96%)	4 (4%)	0	100	100
2	R	93/97 (96%)	87 (94%)	6 (6%)	0	100	100
2	S	93/97 (96%)	85 (91%)	8 (9%)	0	100	100
2	T	93/97 (96%)	90 (97%)	3 (3%)	0	100	100
2	U	93/97 (96%)	89 (96%)	4 (4%)	0	100	100
All	All	7966/8337 (96%)	7673 (96%)	293 (4%)	0	100	100

There are no Ramachandran outliers to report.

5.3.2 Protein sidechains (i)

In the following table, the Percentiles column shows the percent sidechain outliers of the chain as a percentile score with respect to all PDB entries followed by that with respect to all EM entries.

The Analysed column shows the number of residues for which the sidechain conformation was analysed, and the total number of residues.

Mol	Chain	Analysed	Rotameric	Outliers	Percentiles	
1	A	403/414 (97%)	403 (100%)	0	100	100
1	B	403/414 (97%)	403 (100%)	0	100	100
1	C	403/414 (97%)	403 (100%)	0	100	100
1	D	403/414 (97%)	403 (100%)	0	100	100
1	E	403/414 (97%)	403 (100%)	0	100	100
1	F	403/414 (97%)	403 (100%)	0	100	100
1	G	403/414 (97%)	403 (100%)	0	100	100

Continued on next page...

For Manuscript Review

Continued from previous page...

Mol	Chain	Analysed	Rotameric	Outliers	Percentiles	
1	H	405/414 (98%)	404 (100%)	1 (0%)	93	96
1	I	405/414 (98%)	403 (100%)	2 (0%)	88	93
1	J	405/414 (98%)	404 (100%)	1 (0%)	93	96
1	K	405/414 (98%)	404 (100%)	1 (0%)	93	96
1	L	405/414 (98%)	403 (100%)	2 (0%)	88	93
1	M	405/414 (98%)	403 (100%)	2 (0%)	88	93
1	N	405/414 (98%)	403 (100%)	2 (0%)	88	93
2	O	73/80 (91%)	73 (100%)	0	100	100
2	P	73/80 (91%)	73 (100%)	0	100	100
2	Q	73/80 (91%)	73 (100%)	0	100	100
2	R	73/80 (91%)	73 (100%)	0	100	100
2	S	73/80 (91%)	73 (100%)	0	100	100
2	T	73/80 (91%)	73 (100%)	0	100	100
2	U	73/80 (91%)	73 (100%)	0	100	100
All	All	6167/6356 (97%)	6156 (100%)	11 (0%)	93	96

All (11) residues with a non-rotameric sidechain are listed below:

Mol	Chain	Res	Type
1	H	231	ARG
1	I	231	ARG
1	I	311	LYS
1	J	231	ARG
1	K	231	ARG
1	L	231	ARG
1	L	311	LYS
1	M	231	ARG
1	M	311	LYS
1	N	231	ARG
1	N	311	LYS

Sometimes sidechains can be flipped to improve hydrogen bonding and reduce clashes. All (14) such sidechains are listed below:

Mol	Chain	Res	Type
1	A	453	GLN
1	B	453	GLN

Continued on next page...

For Manuscript Review

Continued from previous page...

Mol	Chain	Res	Type
1	C	453	GLN
1	D	194	GLN
1	D	453	GLN
1	E	112	ASN
1	E	453	GLN
1	F	343	GLN
1	F	453	GLN
1	G	453	GLN
1	J	343	GLN
1	L	21	ASN
1	L	97	GLN
1	L	343	GLN

5.3.3 RNA (i)

There are no RNA molecules in this entry.

5.4 Non-standard residues in protein, DNA, RNA chains (i)

There are no non-standard protein/DNA/RNA residues in this entry.

5.5 Carbohydrates (i)

There are no monosaccharides in this entry.

5.6 Ligand geometry (i)

Of 42 ligands modelled in this entry, 28 are monoatomic - leaving 14 for Mogul analysis.

In the following table, the Counts columns list the number of bonds (or angles) for which Mogul statistics could be retrieved, the number of bonds (or angles) that are observed in the model and the number of bonds (or angles) that are defined in the Chemical Component Dictionary. The Link column lists molecule types, if any, to which the group is linked. The Z score for a bond length (or angle) is the number of standard deviations the observed value is removed from the expected value. A bond length (or angle) with $|Z| > 2$ is considered an outlier worth inspection. RMSZ is the root-mean-square of all Z scores of the bond lengths (or angles).

Mol	Type	Chain	Res	Link	Bond lengths			Bond angles		
					Counts	RMSZ	$\#\|Z\| > 2$	Counts	RMSZ	$\#\|Z\| > 2$
3	ATP	D	601	4,5	26,33,33	0.60	0	31,52,52	0.76	2 (6%)

For Manuscript Review

Mol	Type	Chain	Res	Link	Bond lengths			Bond angles		
					Counts	RMSZ	#|Z| > 2	Counts	RMSZ	#|Z| > 2
6	ADP	L	601	4,5	24,29,29	0.90	1 (4%)	29,45,45	1.53	4 (13%)
3	ATP	G	601	4,5	26,33,33	0.60	0	31,52,52	0.74	2 (6%)
6	ADP	N	601	4,5	24,29,29	0.91	1 (4%)	29,45,45	1.49	4 (13%)
3	ATP	F	601	4,5	26,33,33	0.61	0	31,52,52	0.75	2 (6%)
6	ADP	I	601	4,5	24,29,29	0.91	1 (4%)	29,45,45	1.50	4 (13%)
6	ADP	J	601	4,5	24,29,29	0.92	1 (4%)	29,45,45	1.48	4 (13%)
6	ADP	K	601	4,5	24,29,29	0.91	1 (4%)	29,45,45	1.48	4 (13%)
6	ADP	M	601	4,5	24,29,29	0.93	1 (4%)	29,45,45	1.50	4 (13%)
3	ATP	E	601	4,5	26,33,33	0.60	0	31,52,52	0.75	2 (6%)
6	ADP	H	601	4,5	24,29,29	0.91	1 (4%)	29,45,45	1.50	4 (13%)
3	ATP	A	601	4,5	26,33,33	0.60	0	31,52,52	0.75	2 (6%)
3	ATP	C	601	5	26,33,33	0.61	0	31,52,52	0.74	2 (6%)
3	ATP	B	601	4,5	26,33,33	0.60	0	31,52,52	0.75	2 (6%)

In the following table, the Chirals column lists the number of chiral outliers, the number of chiral centers analysed, the number of these observed in the model and the number defined in the Chemical Component Dictionary. Similar counts are reported in the Torsion and Rings columns. '-' means no outliers of that kind were identified.

Mol	Type	Chain	Res	Link	Chirals	Torsions	Rings
3	ATP	D	601	4,5	-	6/18/38/38	0/3/3/3
6	ADP	L	601	4,5	-	5/12/32/32	0/3/3/3
3	ATP	G	601	4,5	-	6/18/38/38	0/3/3/3
6	ADP	N	601	4,5	-	5/12/32/32	0/3/3/3
3	ATP	F	601	4,5	-	4/18/38/38	0/3/3/3
6	ADP	I	601	4,5	-	6/12/32/32	0/3/3/3
6	ADP	J	601	4,5	-	6/12/32/32	0/3/3/3
6	ADP	K	601	4,5	-	6/12/32/32	0/3/3/3
6	ADP	M	601	4,5	-	5/12/32/32	0/3/3/3
3	ATP	E	601	4,5	-	3/18/38/38	0/3/3/3
6	ADP	H	601	4,5	-	5/12/32/32	0/3/3/3
3	ATP	A	601	4,5	-	2/18/38/38	0/3/3/3
3	ATP	C	601	5	-	4/18/38/38	0/3/3/3
3	ATP	B	601	4,5	-	5/18/38/38	0/3/3/3

All (7) bond length outliers are listed below:

For Manuscript Review

Mol	Chain	Res	Type	Atoms	Z	Observed(Å)	Ideal(Å)
6	J	601	ADP	C5-C4	2.41	1.47	1.40
6	L	601	ADP	C5-C4	2.39	1.47	1.40
6	M	601	ADP	C5-C4	2.38	1.47	1.40
6	N	601	ADP	C5-C4	2.38	1.47	1.40
6	I	601	ADP	C5-C4	2.37	1.47	1.40
6	H	601	ADP	C5-C4	2.37	1.47	1.40
6	K	601	ADP	C5-C4	2.37	1.47	1.40

All (42) bond angle outliers are listed below:

Mol	Chain	Res	Type	Atoms	Z	Observed(^{o})	Ideal(^{o})
6	K	601	ADP	PA-O3A-PB	-3.79	119.84	132.83
6	I	601	ADP	PA-O3A-PB	-3.77	119.91	132.83
6	L	601	ADP	PA-O3A-PB	-3.74	119.99	132.83
6	H	601	ADP	PA-O3A-PB	-3.74	120.01	132.83
6	J	601	ADP	PA-O3A-PB	-3.72	120.07	132.83
6	M	601	ADP	PA-O3A-PB	-3.70	120.12	132.83
6	N	601	ADP	PA-O3A-PB	-3.69	120.17	132.83
6	L	601	ADP	N3-C2-N1	-3.43	123.31	128.68
6	J	601	ADP	C3'-C2'-C1'	3.36	106.03	100.98
6	I	601	ADP	C3'-C2'-C1'	3.33	106.00	100.98
6	H	601	ADP	C3'-C2'-C1'	3.29	105.93	100.98
6	M	601	ADP	C3'-C2'-C1'	3.27	105.90	100.98
6	M	601	ADP	N3-C2-N1	-3.26	123.58	128.68
6	K	601	ADP	C3'-C2'-C1'	3.25	105.87	100.98
6	H	601	ADP	N3-C2-N1	-3.24	123.61	128.68
6	N	601	ADP	N3-C2-N1	-3.24	123.62	128.68
6	N	601	ADP	C3'-C2'-C1'	3.22	105.83	100.98
6	L	601	ADP	C3'-C2'-C1'	3.22	105.82	100.98
6	I	601	ADP	N3-C2-N1	-3.22	123.65	128.68
6	J	601	ADP	N3-C2-N1	-3.11	123.82	128.68
6	K	601	ADP	N3-C2-N1	-3.08	123.86	128.68
6	H	601	ADP	C4-C5-N7	-2.93	106.35	109.40
6	L	601	ADP	C4-C5-N7	-2.87	106.41	109.40
6	K	601	ADP	C4-C5-N7	-2.76	106.52	109.40
6	N	601	ADP	C4-C5-N7	-2.75	106.53	109.40
6	J	601	ADP	C4-C5-N7	-2.75	106.54	109.40
6	I	601	ADP	C4-C5-N7	-2.38	106.92	109.40
3	D	601	ATP	C5-C6-N6	2.32	123.88	120.35
3	A	601	ATP	C5-C6-N6	2.31	123.86	120.35
6	M	601	ADP	C4-C5-N7	-2.29	107.01	109.40
3	B	601	ATP	C5-C6-N6	2.29	123.83	120.35
3	F	601	ATP	C5-C6-N6	2.26	123.79	120.35

Continued on next page...

For Manuscript Review

Continued from previous page...

Mol	Chain	Res	Type	Atoms	Z	Observed(^{o})	Ideal(^{o})
3	E	601	ATP	C5-C6-N6	2.26	123.79	120.35
3	G	601	ATP	C5-C6-N6	2.25	123.78	120.35
3	C	601	ATP	C5-C6-N6	2.25	123.77	120.35
3	F	601	ATP	PB-O3B-PG	2.07	139.94	132.83
3	C	601	ATP	PB-O3B-PG	2.06	139.90	132.83
3	D	601	ATP	PB-O3B-PG	2.06	139.90	132.83
3	A	601	ATP	PB-O3B-PG	2.06	139.88	132.83
3	B	601	ATP	PB-O3B-PG	2.05	139.87	132.83
3	E	601	ATP	PB-O3B-PG	2.05	139.85	132.83
3	G	601	ATP	PB-O3B-PG	2.04	139.83	132.83

There are no chirality outliers.

All (68) torsion outliers are listed below:

Mol	Chain	Res	Type	Atoms
3	B	601	ATP	C3'-C4'-C5'-O5'
3	D	601	ATP	C5'-O5'-PA-O2A
3	D	601	ATP	C3'-C4'-C5'-O5'
3	E	601	ATP	C3'-C4'-C5'-O5'
3	G	601	ATP	C3'-C4'-C5'-O5'
6	H	601	ADP	C5'-O5'-PA-O3A
6	I	601	ADP	C5'-O5'-PA-O1A
6	I	601	ADP	C5'-O5'-PA-O3A
6	J	601	ADP	C5'-O5'-PA-O1A
6	J	601	ADP	C5'-O5'-PA-O3A
6	J	601	ADP	O4'-C4'-C5'-O5'
6	K	601	ADP	C5'-O5'-PA-O1A
6	K	601	ADP	C5'-O5'-PA-O3A
6	L	601	ADP	C5'-O5'-PA-O1A
6	L	601	ADP	O4'-C4'-C5'-O5'
6	M	601	ADP	C5'-O5'-PA-O1A
6	M	601	ADP	C5'-O5'-PA-O3A
6	N	601	ADP	C5'-O5'-PA-O1A
6	N	601	ADP	C5'-O5'-PA-O3A
6	H	601	ADP	O4'-C4'-C5'-O5'
6	H	601	ADP	C3'-C4'-C5'-O5'
6	I	601	ADP	O4'-C4'-C5'-O5'
6	J	601	ADP	C3'-C4'-C5'-O5'
6	K	601	ADP	O4'-C4'-C5'-O5'
6	M	601	ADP	O4'-C4'-C5'-O5'
6	N	601	ADP	O4'-C4'-C5'-O5'
3	D	601	ATP	O4'-C4'-C5'-O5'

Continued on next page...

For Manuscript Review

Continued from previous page...

Mol	Chain	Res	Type	Atoms
6	K	601	ADP	C3'-C4'-C5'-O5'
6	L	601	ADP	C3'-C4'-C5'-O5'
6	M	601	ADP	C3'-C4'-C5'-O5'
6	I	601	ADP	C3'-C4'-C5'-O5'
6	N	601	ADP	C3'-C4'-C5'-O5'
3	A	601	ATP	C3'-C4'-C5'-O5'
3	B	601	ATP	O4'-C4'-C5'-O5'
3	E	601	ATP	O4'-C4'-C5'-O5'
3	G	601	ATP	O4'-C4'-C5'-O5'
3	C	601	ATP	PB-O3A-PA-O1A
3	F	601	ATP	PB-O3B-PG-O2G
3	C	601	ATP	PG-O3B-PB-O1B
6	K	601	ADP	PB-O3A-PA-O2A
3	D	601	ATP	C5'-O5'-PA-O1A
6	H	601	ADP	C5'-O5'-PA-O1A
3	F	601	ATP	C3'-C4'-C5'-O5'
3	A	601	ATP	O4'-C4'-C5'-O5'
3	F	601	ATP	PA-O3A-PB-O1B
6	H	601	ADP	PB-O3A-PA-O2A
6	I	601	ADP	PB-O3A-PA-O2A
6	J	601	ADP	PB-O3A-PA-O2A
6	L	601	ADP	PB-O3A-PA-O2A
6	M	601	ADP	PB-O3A-PA-O2A
6	N	601	ADP	PB-O3A-PA-O2A
3	C	601	ATP	PB-O3A-PA-O2A
3	G	601	ATP	PA-O3A-PB-O1B
3	E	601	ATP	PB-O3B-PG-O2G
3	G	601	ATP	PG-O3B-PB-O3A
3	D	601	ATP	C5'-O5'-PA-O3A
6	L	601	ADP	C5'-O5'-PA-O3A
3	B	601	ATP	PG-O3B-PB-O2B
3	B	601	ATP	PA-O3A-PB-O2B
3	C	601	ATP	PG-O3B-PB-O2B
3	D	601	ATP	PA-O3A-PB-O2B
3	F	601	ATP	PA-O3A-PB-O2B
3	G	601	ATP	PA-O3A-PB-O2B
6	I	601	ADP	PB-O3A-PA-O1A
6	J	601	ADP	PB-O3A-PA-O1A
6	K	601	ADP	PB-O3A-PA-O1A
3	B	601	ATP	C5'-O5'-PA-O1A
3	G	601	ATP	C5'-O5'-PA-O1A

There are no ring outliers.

For Manuscript Review

14 monomers are involved in 81 short contacts:

Mol	Chain	Res	Type	Clashes	Symm-Clashes
3	D	601	ATP	6	0
6	L	601	ADP	4	0
3	G	601	ATP	7	0
6	N	601	ADP	7	0
3	F	601	ATP	6	0
6	I	601	ADP	4	0
6	J	601	ADP	5	0
6	K	601	ADP	5	0
6	M	601	ADP	4	0
3	E	601	ATP	8	0
6	H	601	ADP	5	0
3	A	601	ATP	6	0
3	C	601	ATP	6	0
3	B	601	ATP	8	0

The following is a two-dimensional graphical depiction of Mogul quality analysis of bond lengths, bond angles, torsion angles, and ring geometry for all instances of the Ligand of Interest. In addition, ligands with molecular weight > 250 and outliers as shown on the validation Tables will also be included. For torsion angles, if less then 5% of the Mogul distribution of torsion angles is within 10 degrees of the torsion angle in question, then that torsion angle is considered an outlier. Any bond that is central to one or more torsion angles identified as an outlier by Mogul will be highlighted in the graph. For rings, the root-mean-square deviation (RMSD) between the ring in question and similar rings identified by Mogul is calculated over all ring torsion angles. If the average RMSD is greater than 60 degrees and the minimal RMSD between the ring in question and any Mogul-identified rings is also greater than 60 degrees, then that ring is considered an outlier. The outliers are highlighted in purple. The color gray indicates Mogul did not find sufficient equivalents in the CSD to analyse the geometry.

For Manuscript Review

Ligand ATP D 601

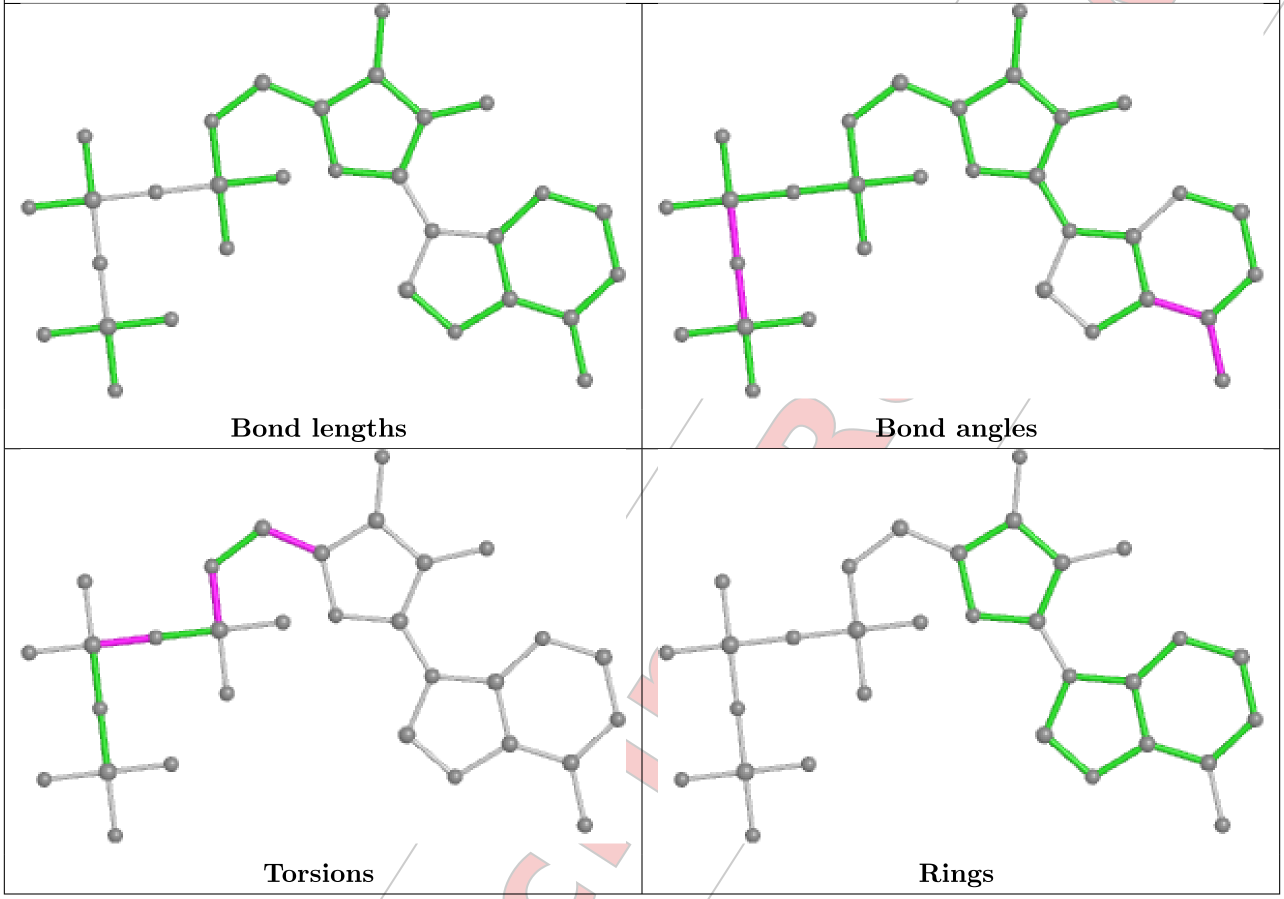

Ligand ADP L 601

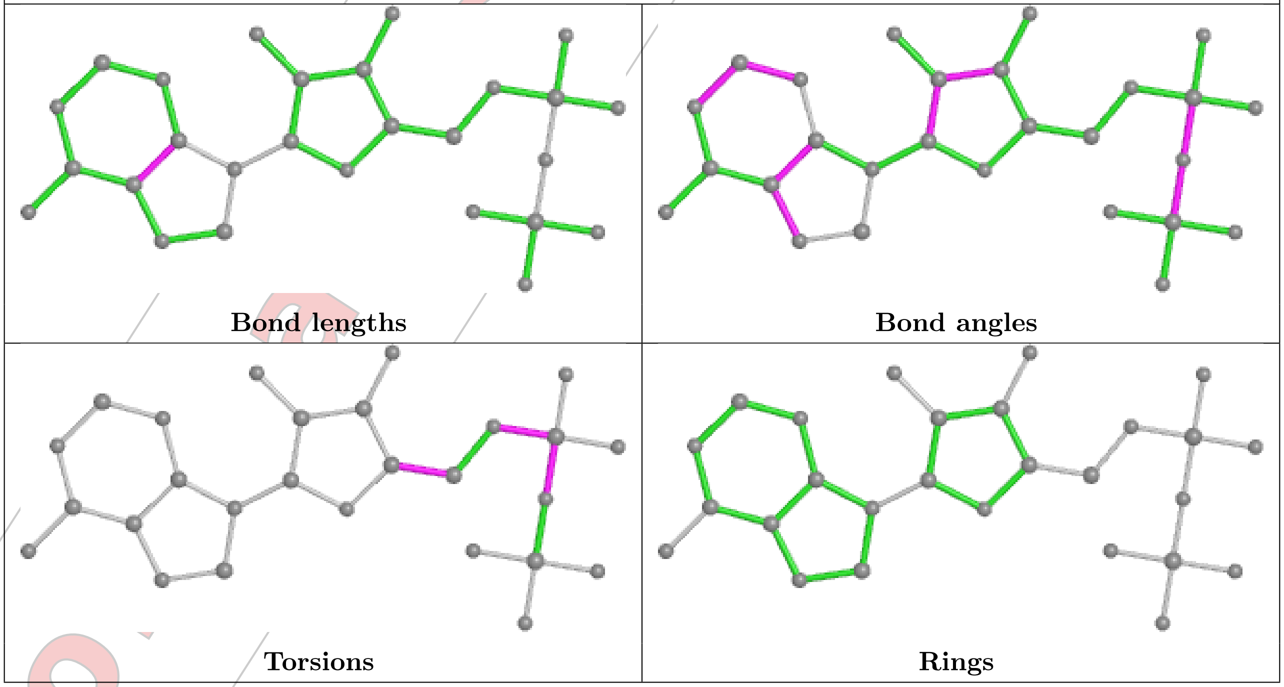

Ligand ATP G 601

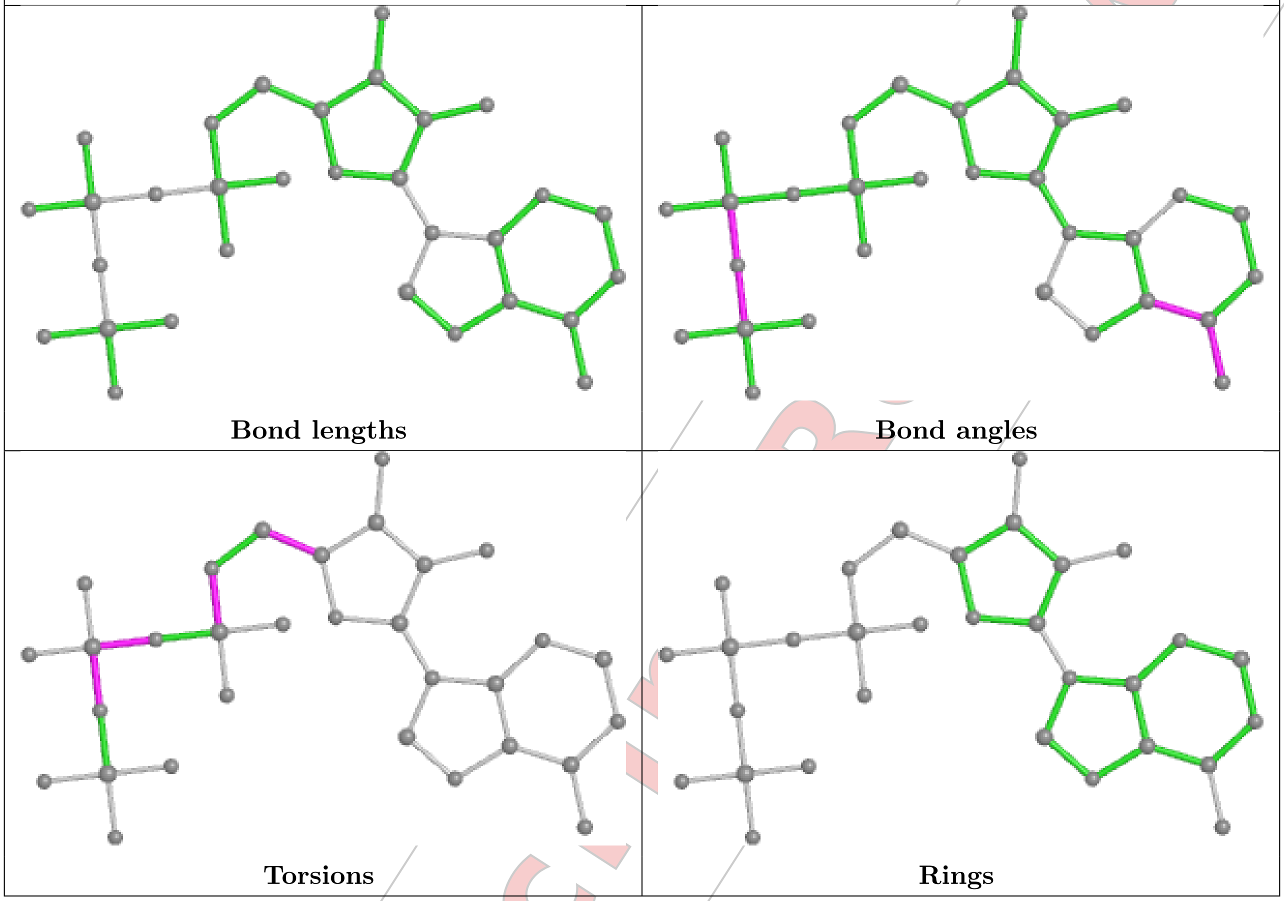

Ligand ADP N 601

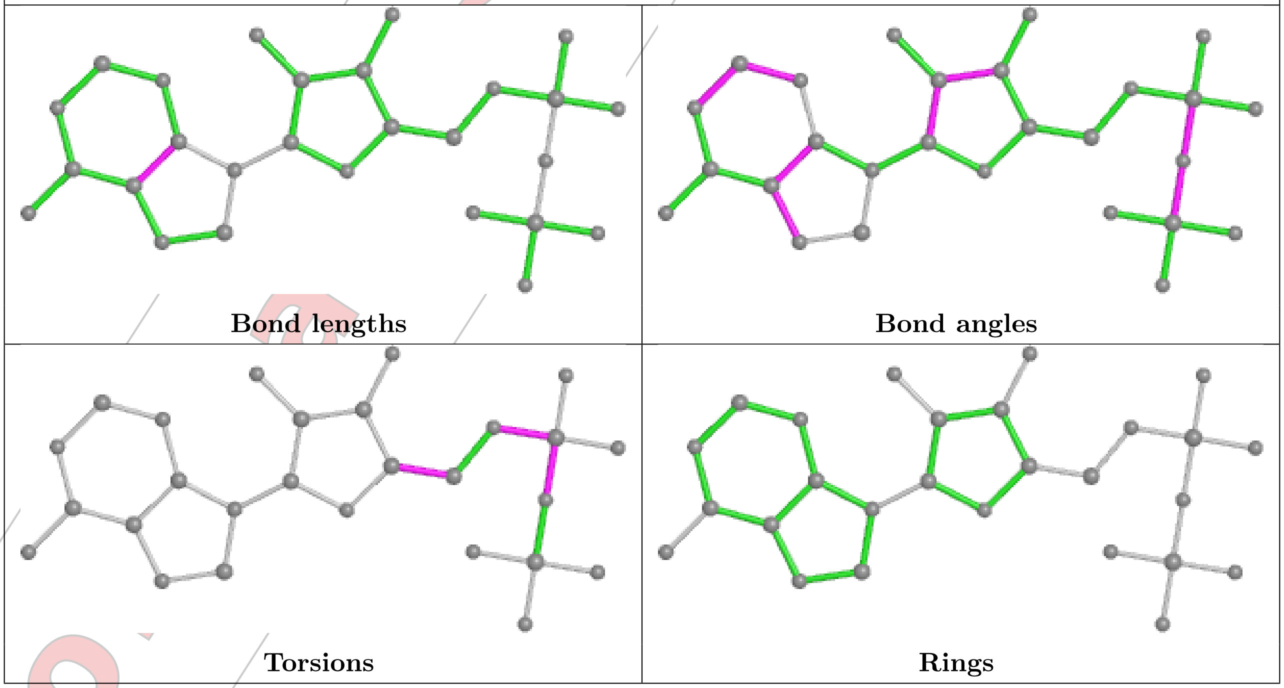

Ligand ATP F 601

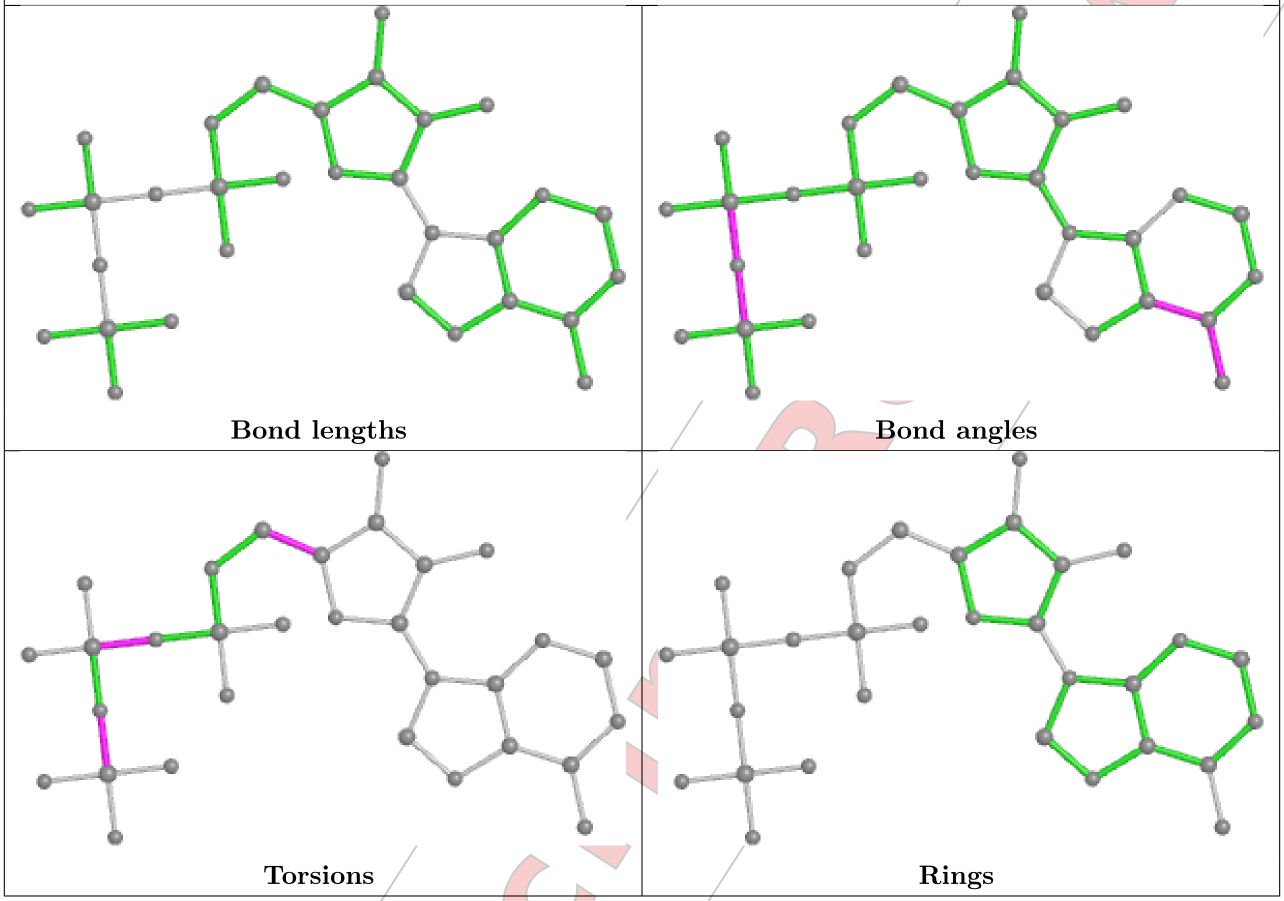

Ligand ADP I 601

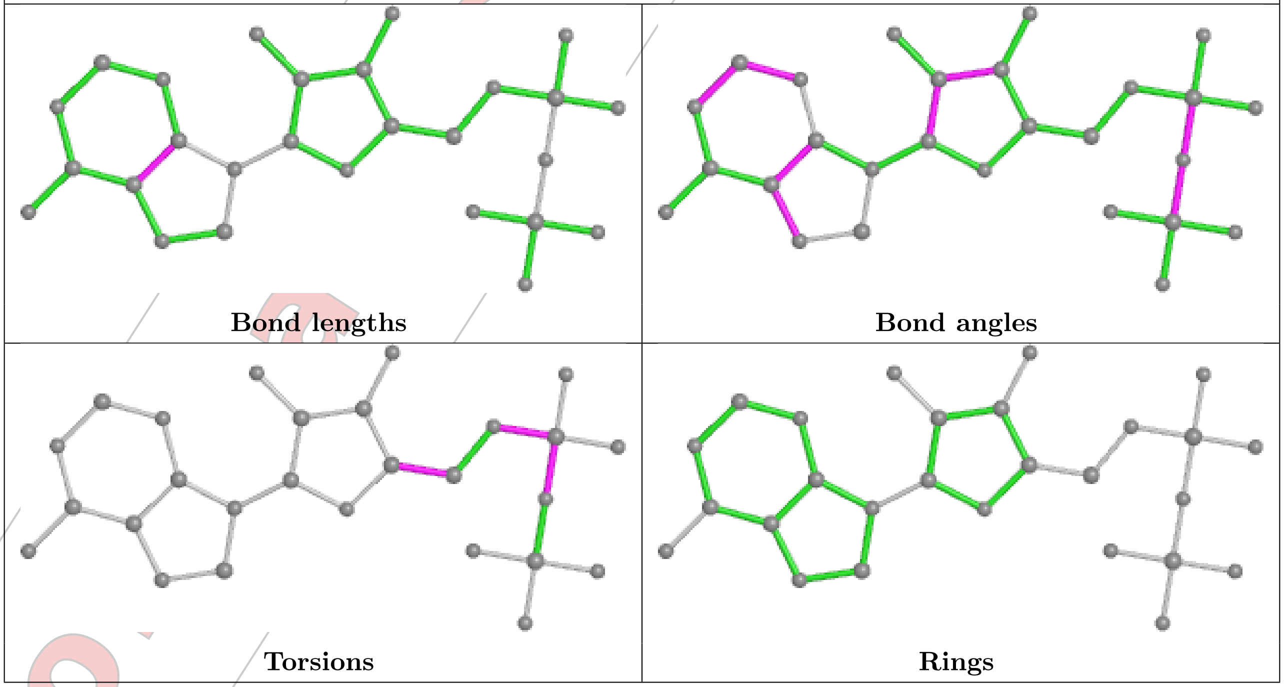

Ligand ADP J 601

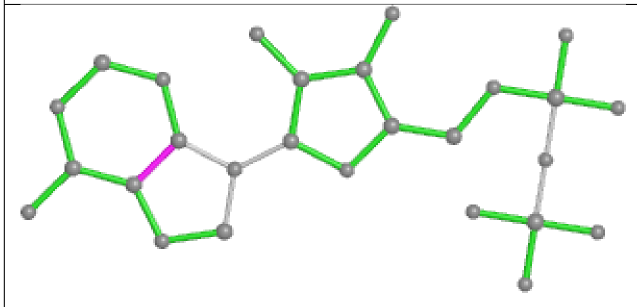

Bond lengths

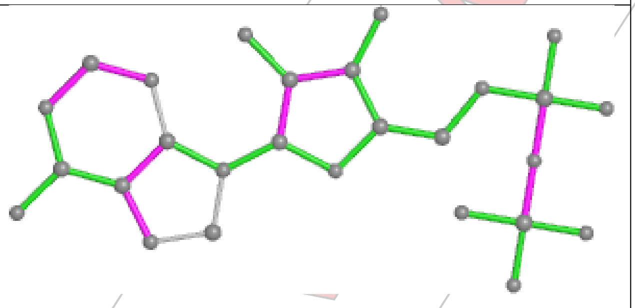

Bond angles

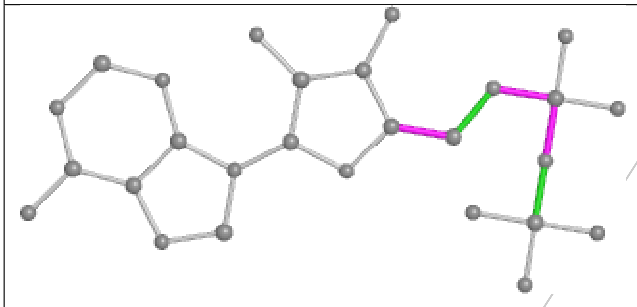

Torsions

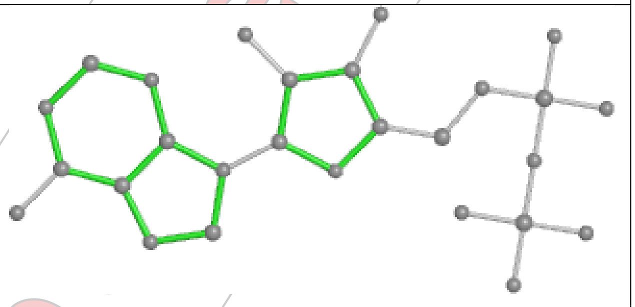

Rings

Ligand ADP K 601

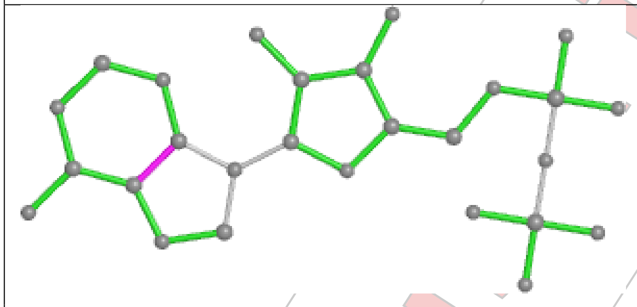

Bond lengths

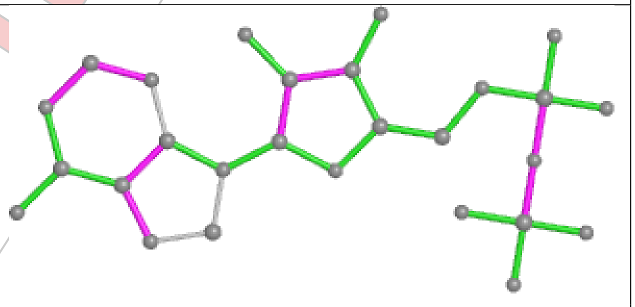

Bond angles

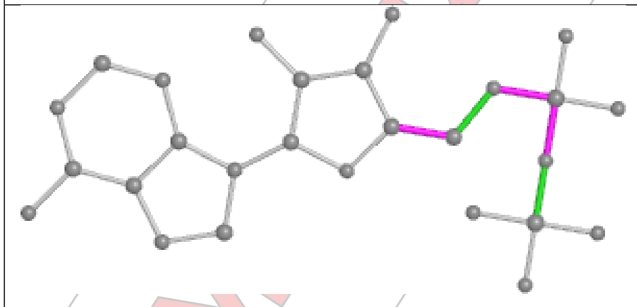

Torsions

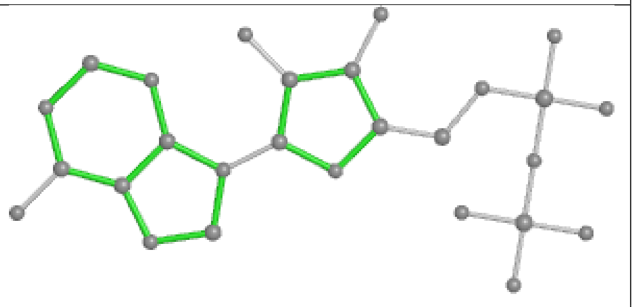

Rings

Ligand ADP M 601

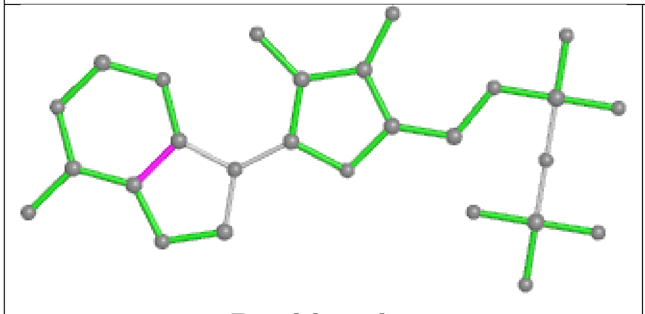
Bond lengths

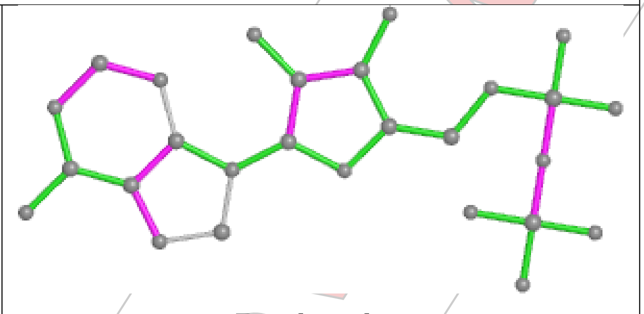
Bond angles

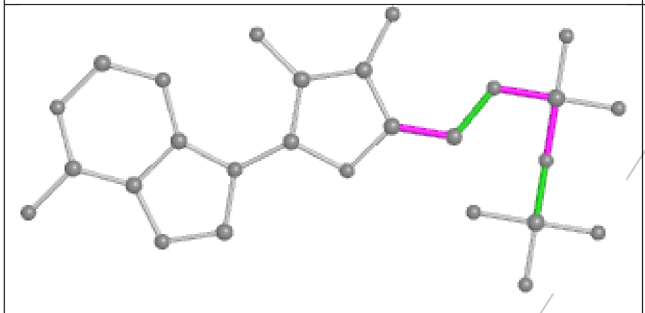
Torsions

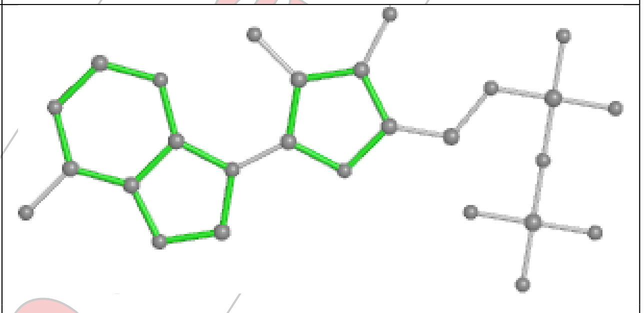
Rings

Ligand ATP E 601

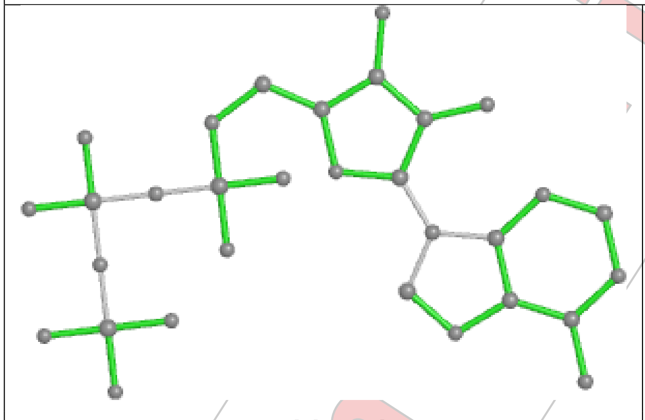
Bond lengths

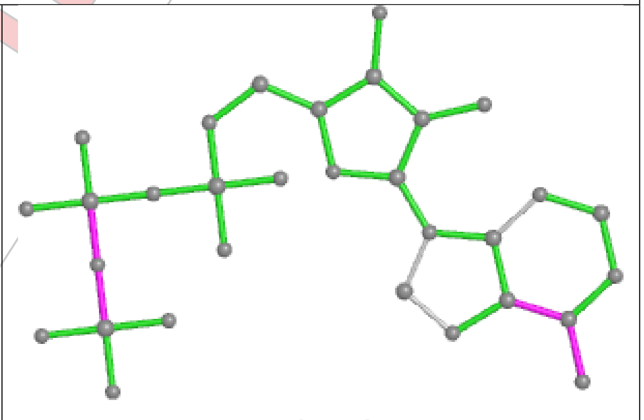
Bond angles

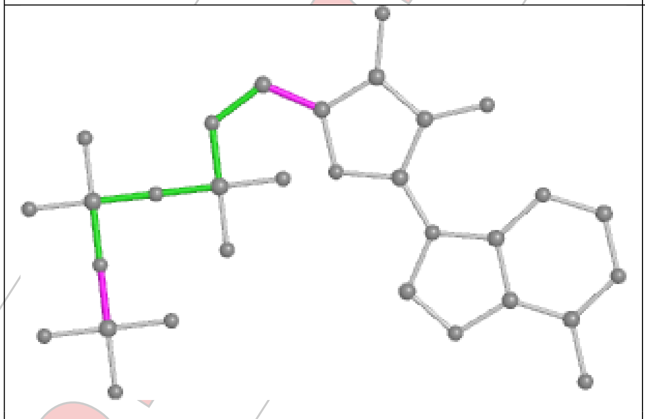
Torsions

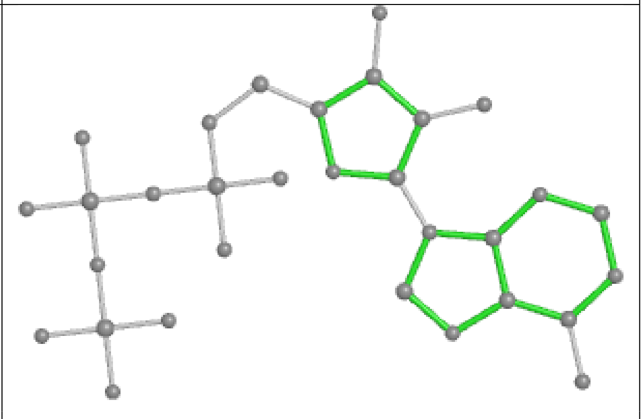
Rings

Ligand ADP H 601

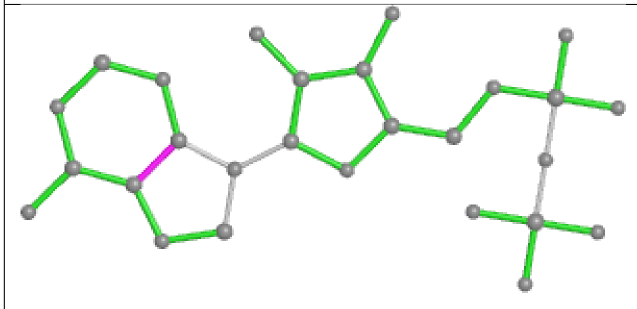
Bond lengths

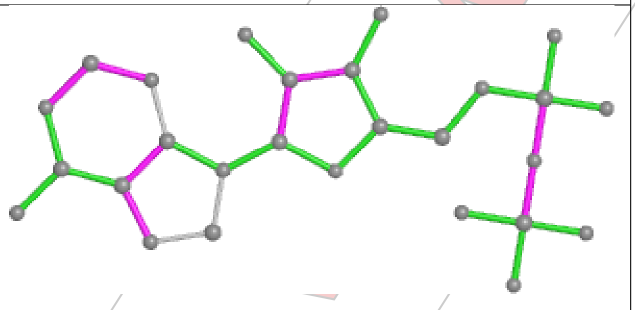
Bond angles

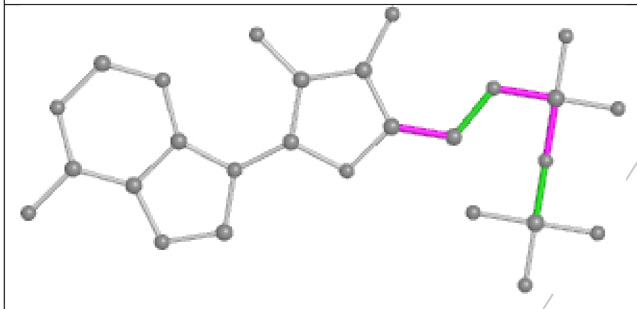
Torsions

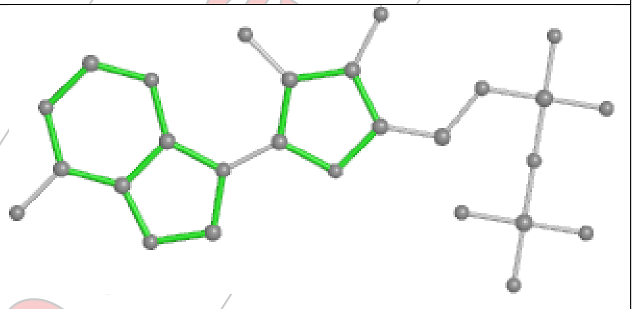
Rings

Ligand ATP A 601

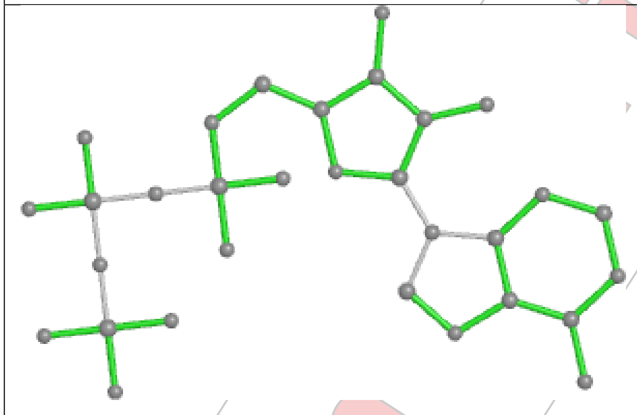
Bond lengths

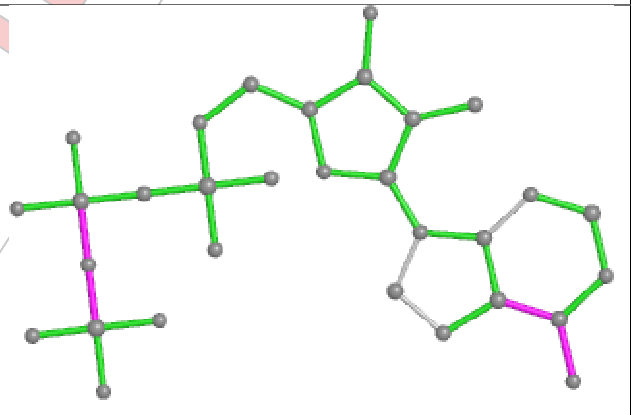
Bond angles

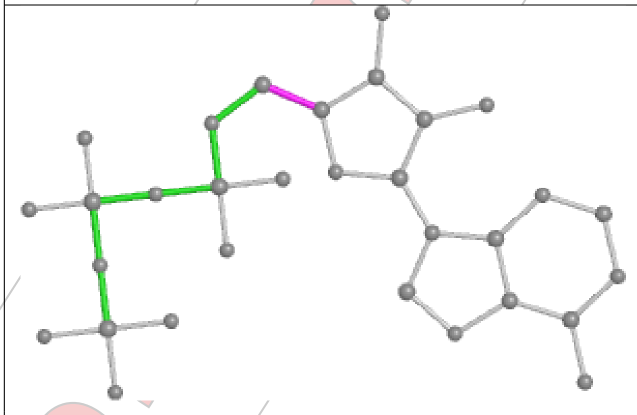
Torsions

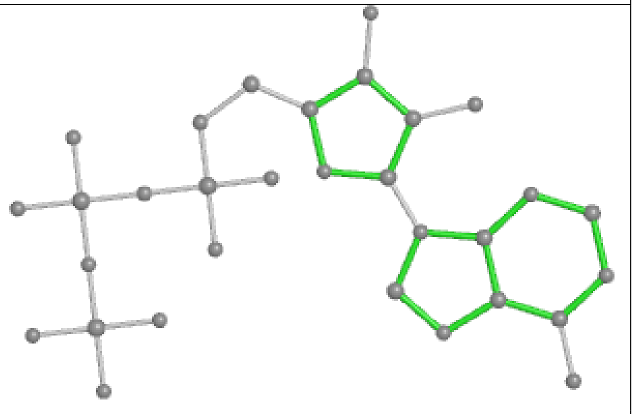
Rings

Ligand ATP C 601

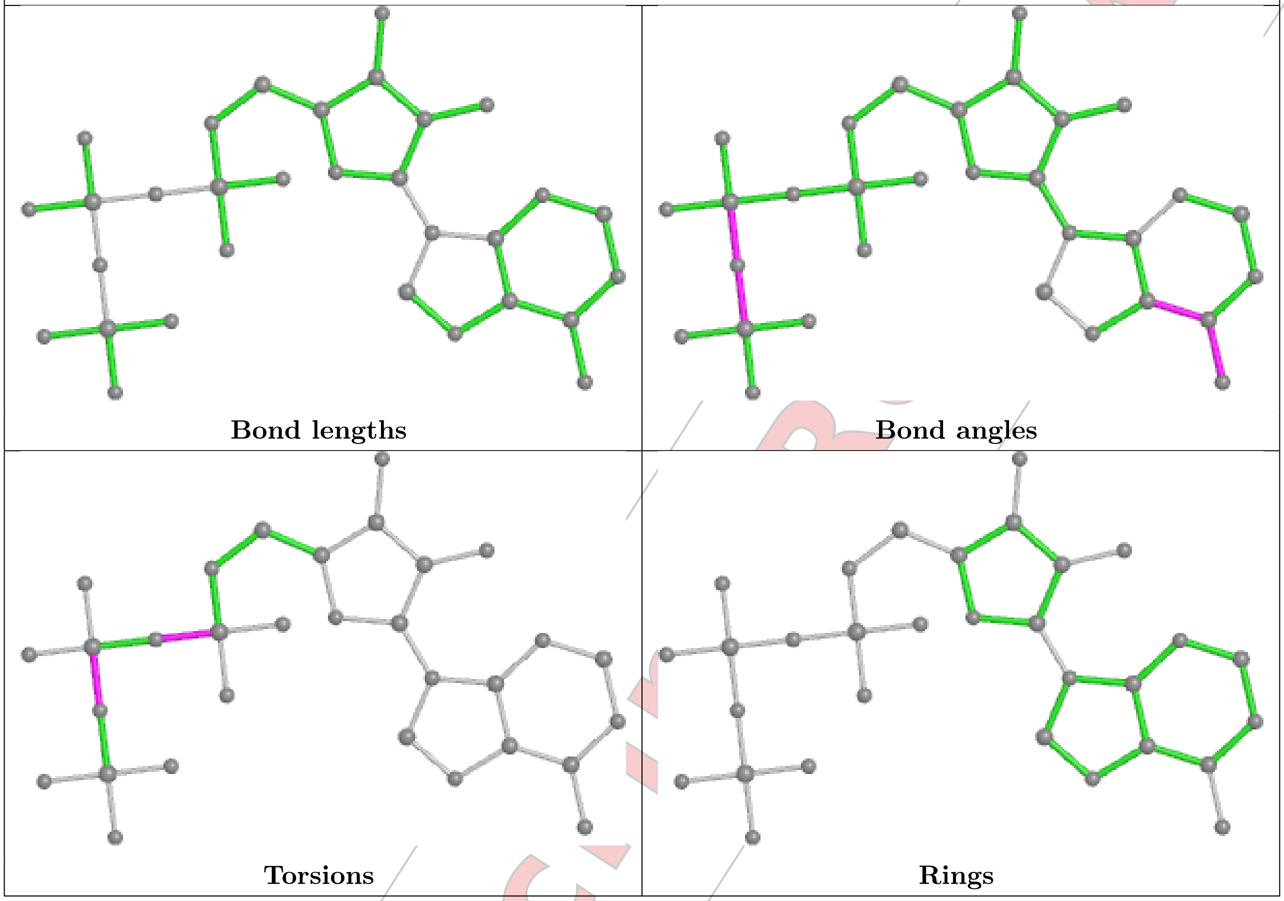

For Manuscript Review

Ligand ATP B 601

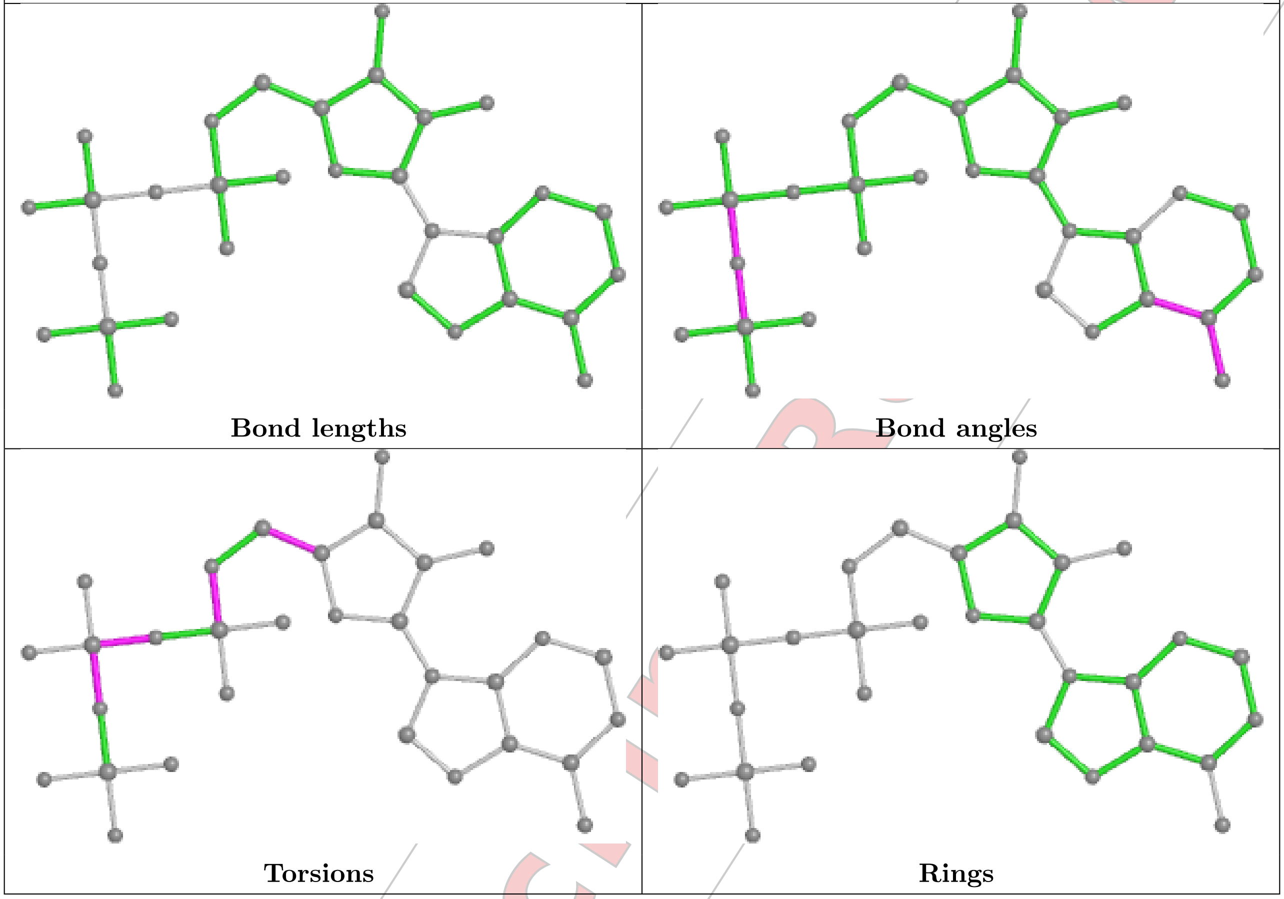

5.7 Other polymers (i)

There are no such residues in this entry.

5.8 Polymer linkage issues (i)

There are no chain breaks in this entry.

For Manuscript Review

6 Map visualisation

This section contains visualisations of the EMDB entry EMD-18737. These allow visual inspection of the internal detail of the map and identification of artifacts.

Images derived from a raw map, generated by summing the deposited half-maps, are presented below the corresponding image components of the primary map to allow further visual inspection and comparison with those of the primary map.

6.1 Orthogonal projections

6.1.1 Primary map

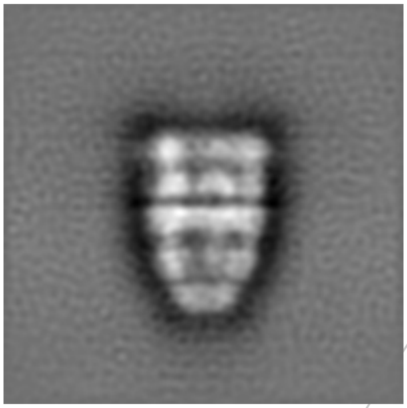
X

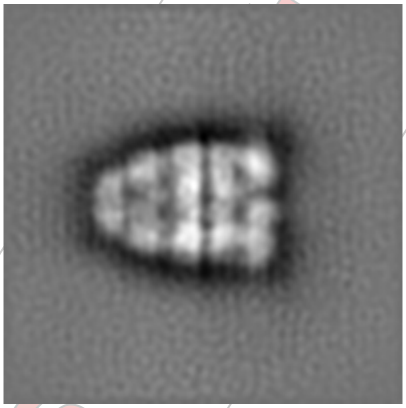
Y

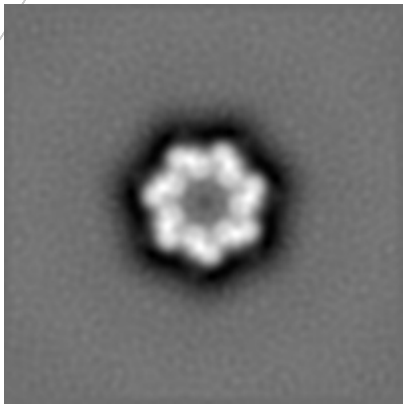
Z

6.1.2 Raw map

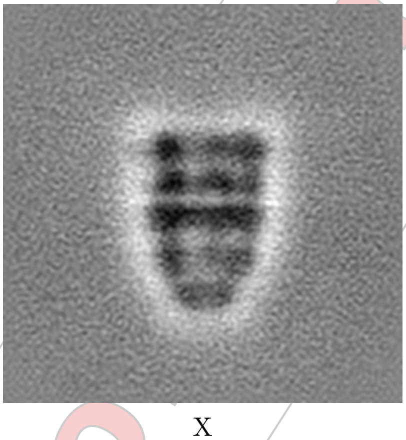
X

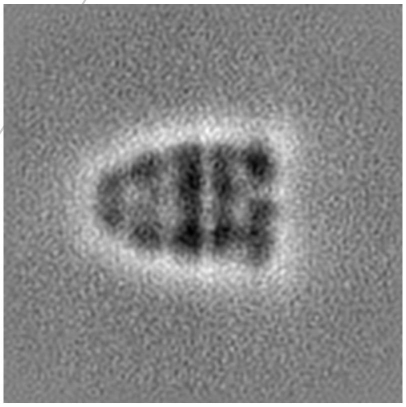
Y

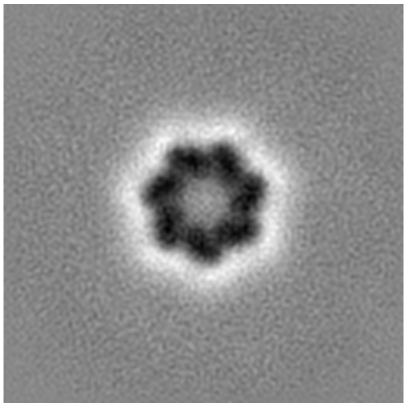
Z

The images above show the map projected in three orthogonal directions.

For Manuscript Review

6.2 Central slices (i)

6.2.1 Primary map

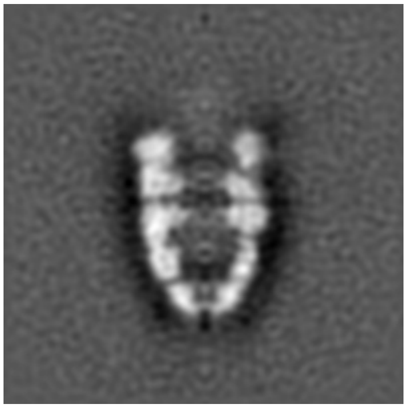

X Index: 64

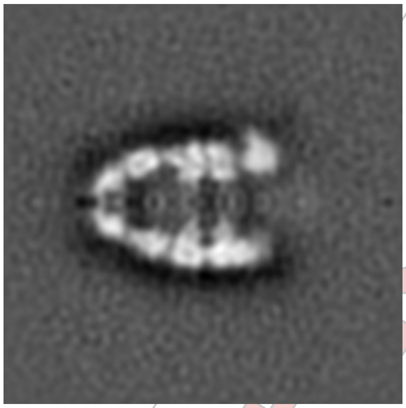

Y Index: 64

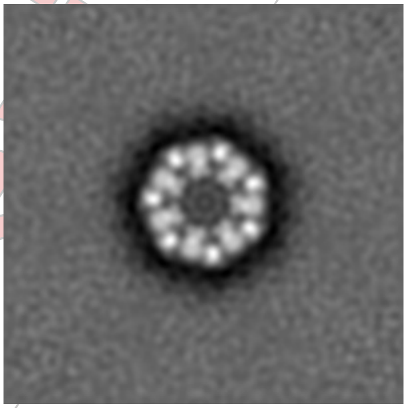

Z Index: 64

6.2.2 Raw map

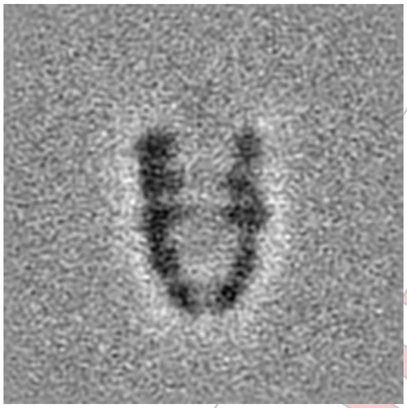

X Index: 64

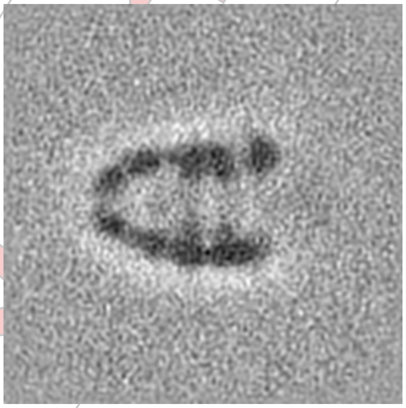

Y Index: 64

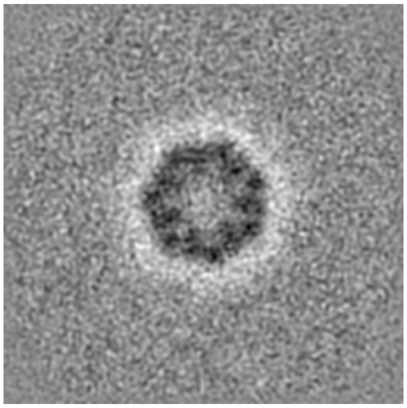

Z Index: 64

The images above show central slices of the map in three orthogonal directions.

For Manuscript Review

6.3 Largest variance slices (i)

6.3.1 Primary map

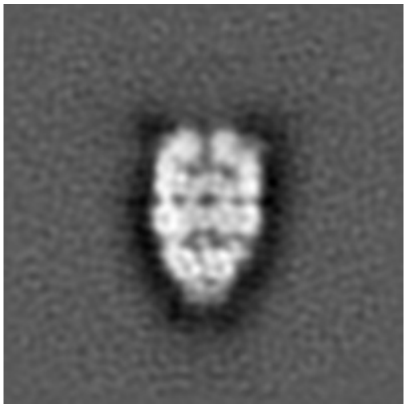
X Index: 53

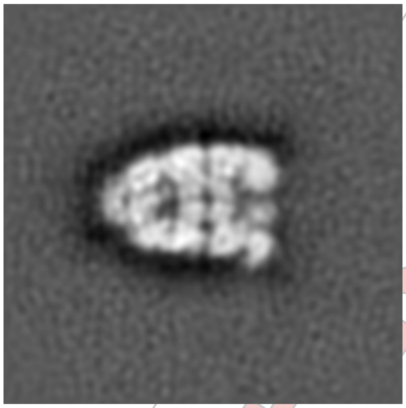
Y Index: 54

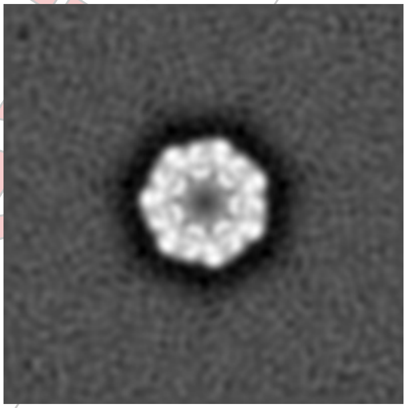
Z Index: 61

6.3.2 Raw map

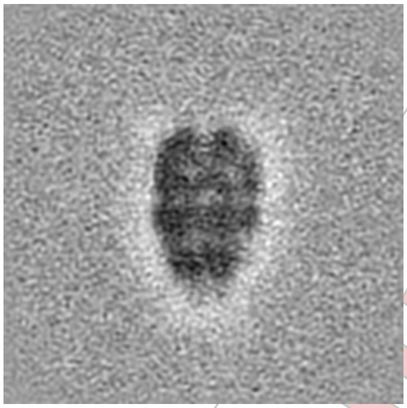
X Index: 52

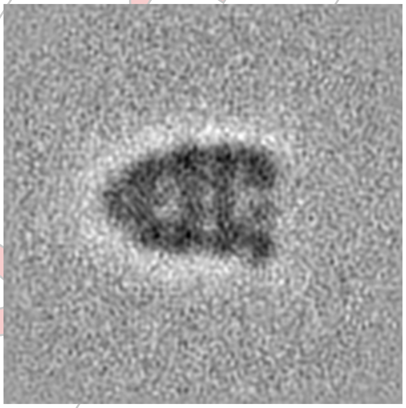
Y Index: 54

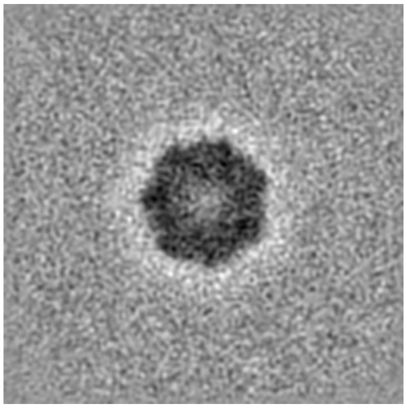
Z Index: 60

The images above show the largest variance slices of the map in three orthogonal directions.

For Manuscript Review

6.4 Orthogonal standard-deviation projections (False-color) (i)

6.4.1 Primary map

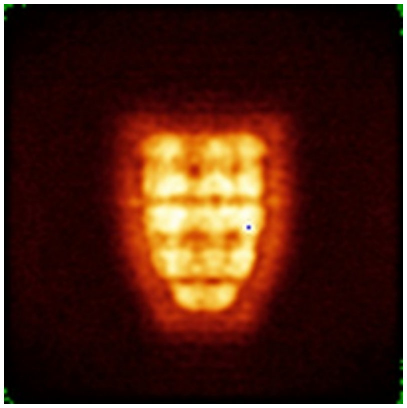
X

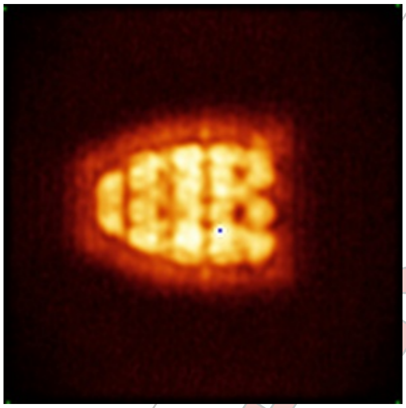
Y

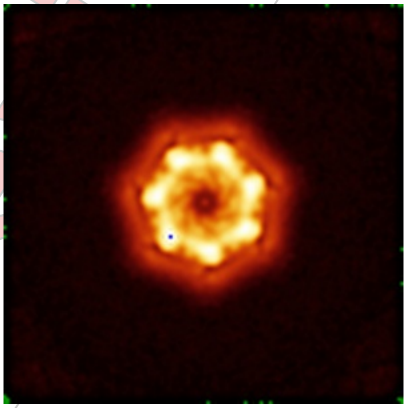
Z

6.4.2 Raw map

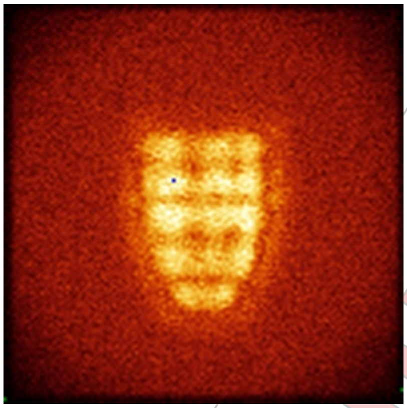
X

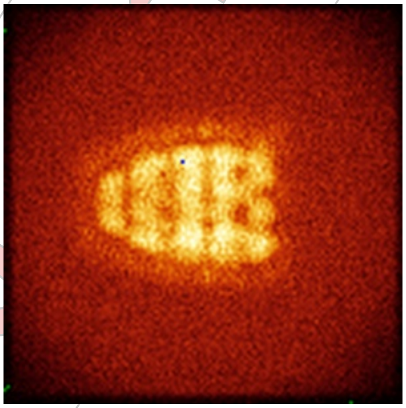
Y

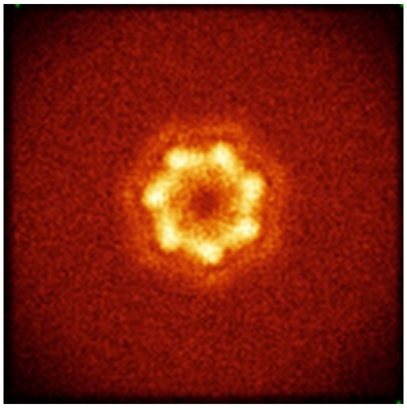
Z

The images above show the map standard deviation projections with false color in three orthogonal directions. Minimum values are shown in green, max in blue, and dark to light orange shades represent small to large values respectively.

For Manuscript Review

6.5 Orthogonal surface views (i)

6.5.1 Primary map

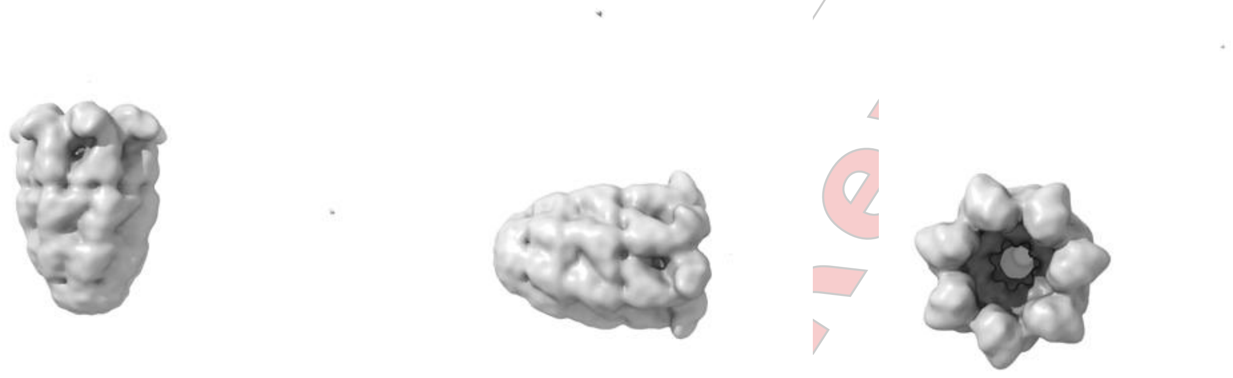

X Y Z

The images above show the 3D surface view of the map at the recommended contour level 0.0981. These images, in conjunction with the slice images, may facilitate assessment of whether an appropriate contour level has been provided.

6.5.2 Raw map

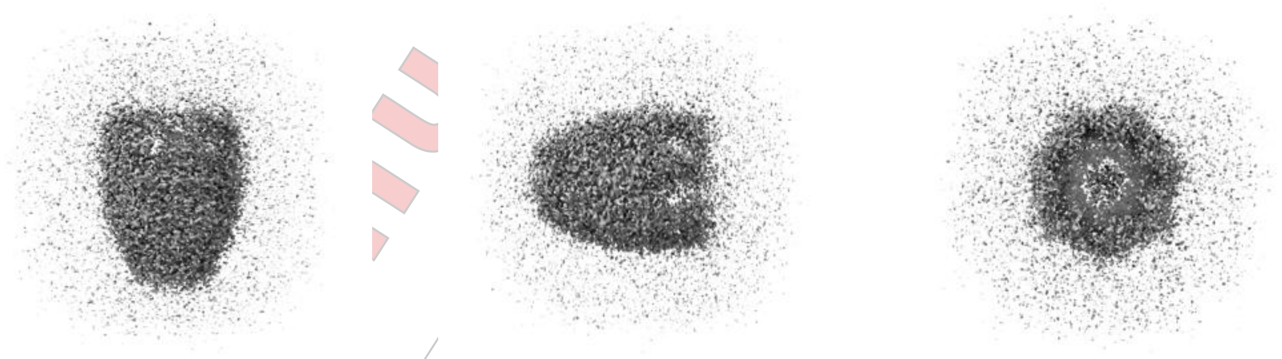

X Y Z

These images show the 3D surface of the raw map. The raw map's contour level was selected so that its surface encloses the same volume as the primary map does at its recommended contour level.

6.6 Mask visualisation (i)

This section was not generated. No masks/segmentation were deposited.

For Manuscript Review

7 Map analysis (i)

This section contains the results of statistical analysis of the map.

7.1 Map-value distribution (i)

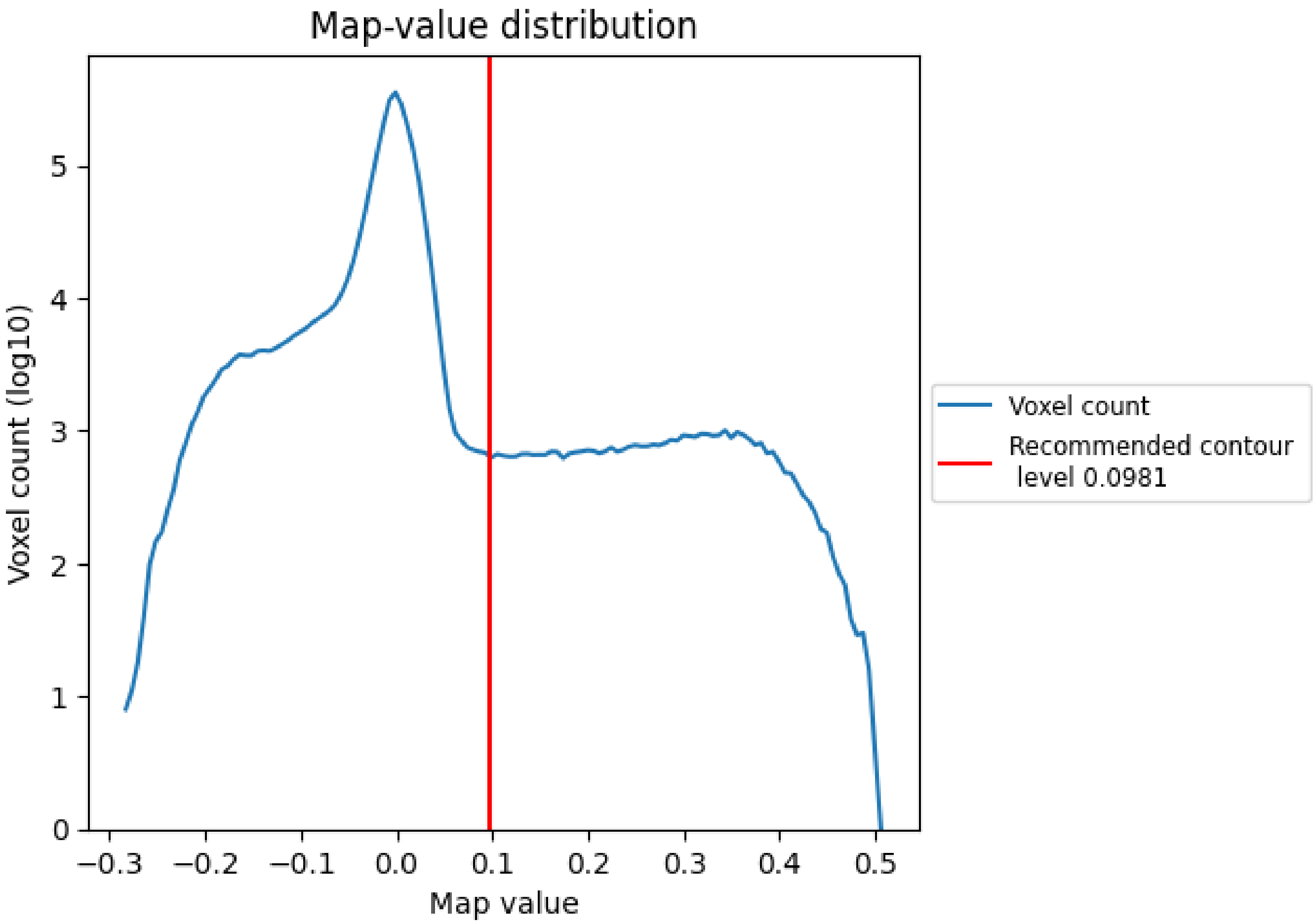

The map-value distribution is plotted in 128 intervals along the x-axis. The y-axis is logarithmic. A spike in this graph at zero usually indicates that the volume has been masked.

For Manuscript Review

7.2 Volume estimate (i)

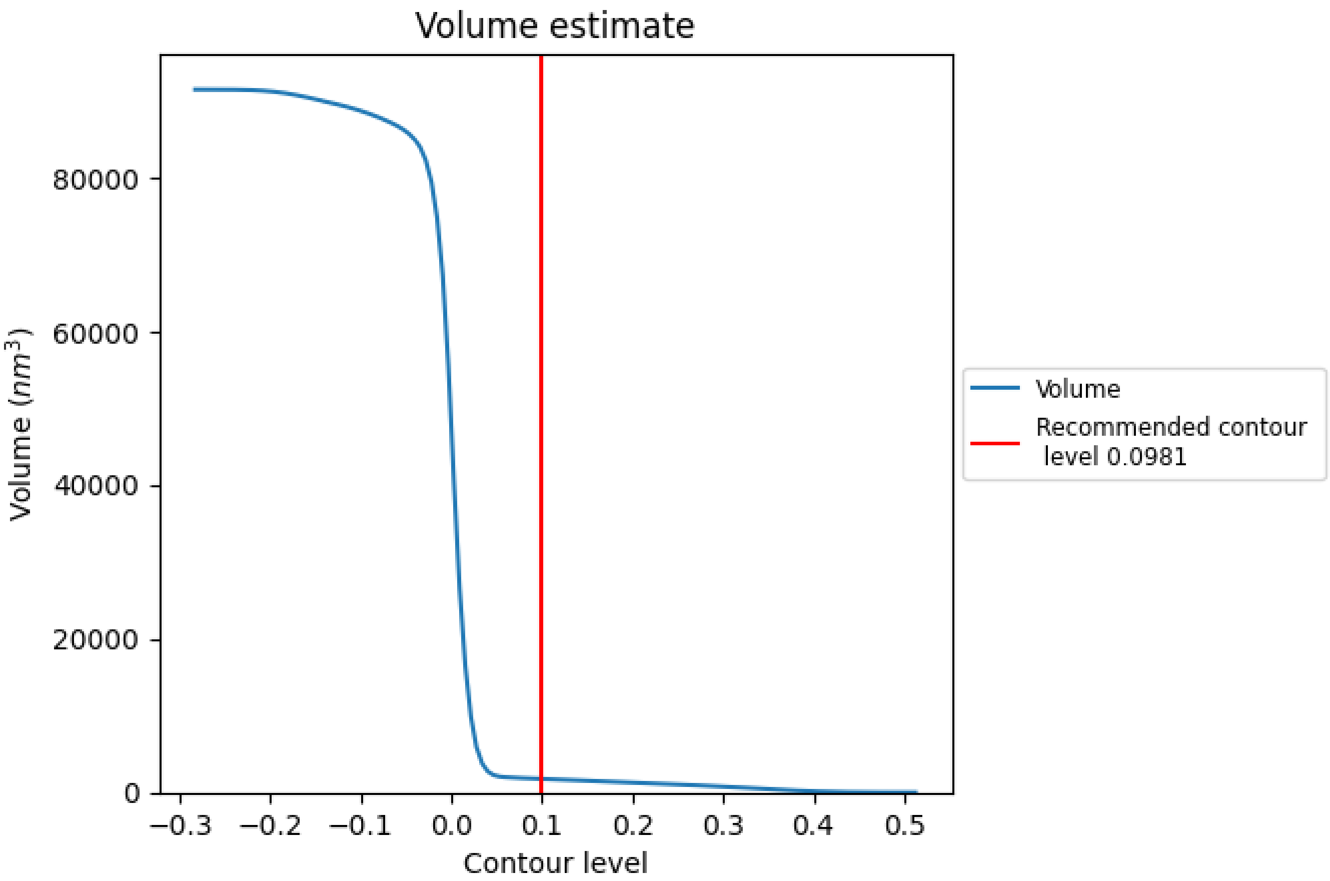

The volume at the recommended contour level is 1763 nm^3; this corresponds to an approximate mass of 1593 kDa.

The volume estimate graph shows how the enclosed volume varies with the contour level. The recommended contour level is shown as a vertical line and the intersection between the line and the curve gives the volume of the enclosed surface at the given level.

For Manuscript Review

7.3 Rotationally averaged power spectrum (i)

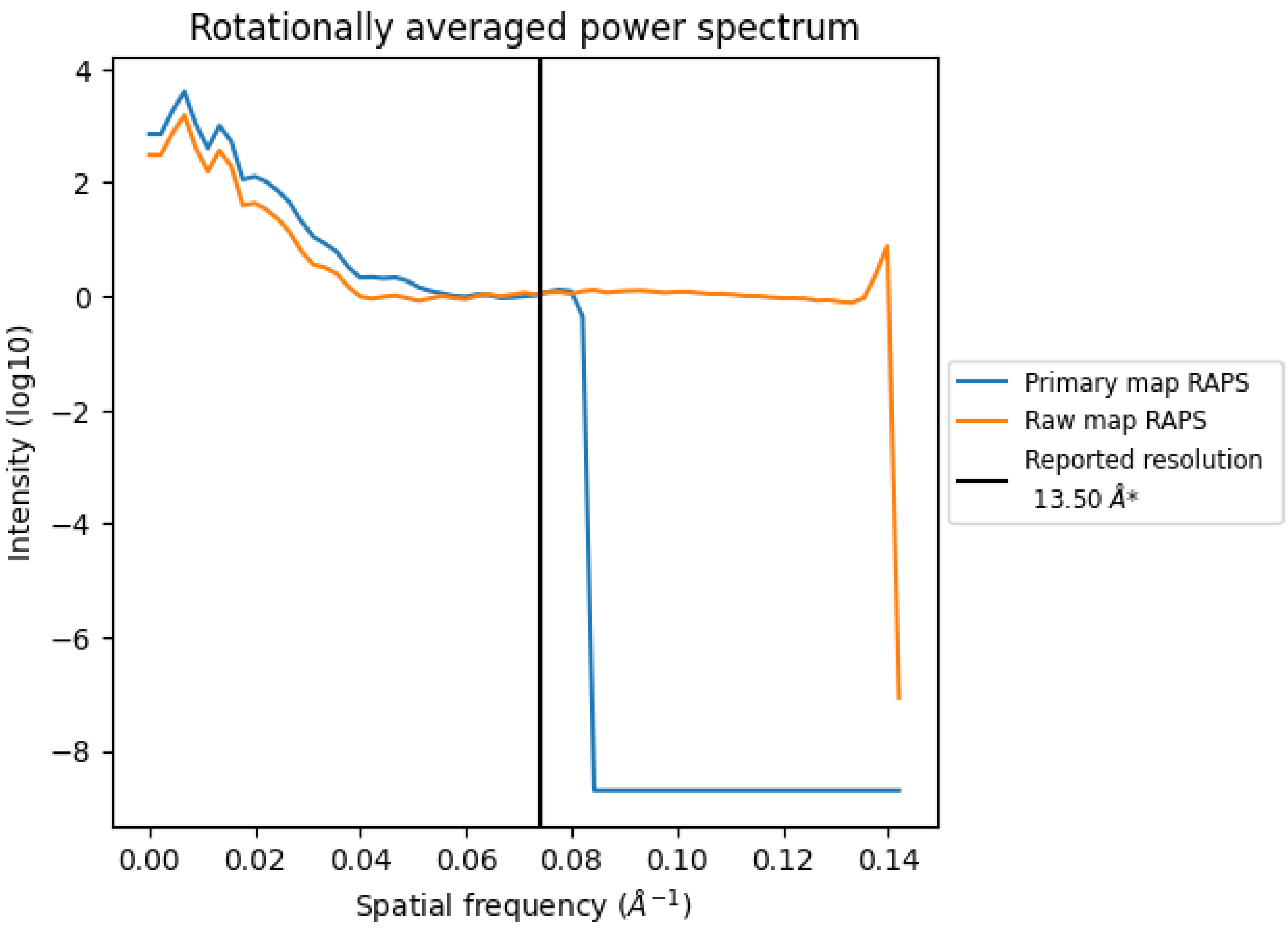

*Reported resolution corresponds to spatial frequency of 0.074 Å^{-1}

For Manuscript Review

8 Fourier-Shell correlation (i)

Fourier-Shell Correlation (FSC) is the most commonly used method to estimate the resolution of single-particle and subtomogram-averaged maps. The shape of the curve depends on the imposed symmetry, mask and whether or not the two 3D reconstructions used were processed from a common reference. The reported resolution is shown as a black line. A curve is displayed for the half-bit criterion in addition to lines showing the 0.143 gold standard cut-off and 0.5 cut-off.

8.1 FSC (i)

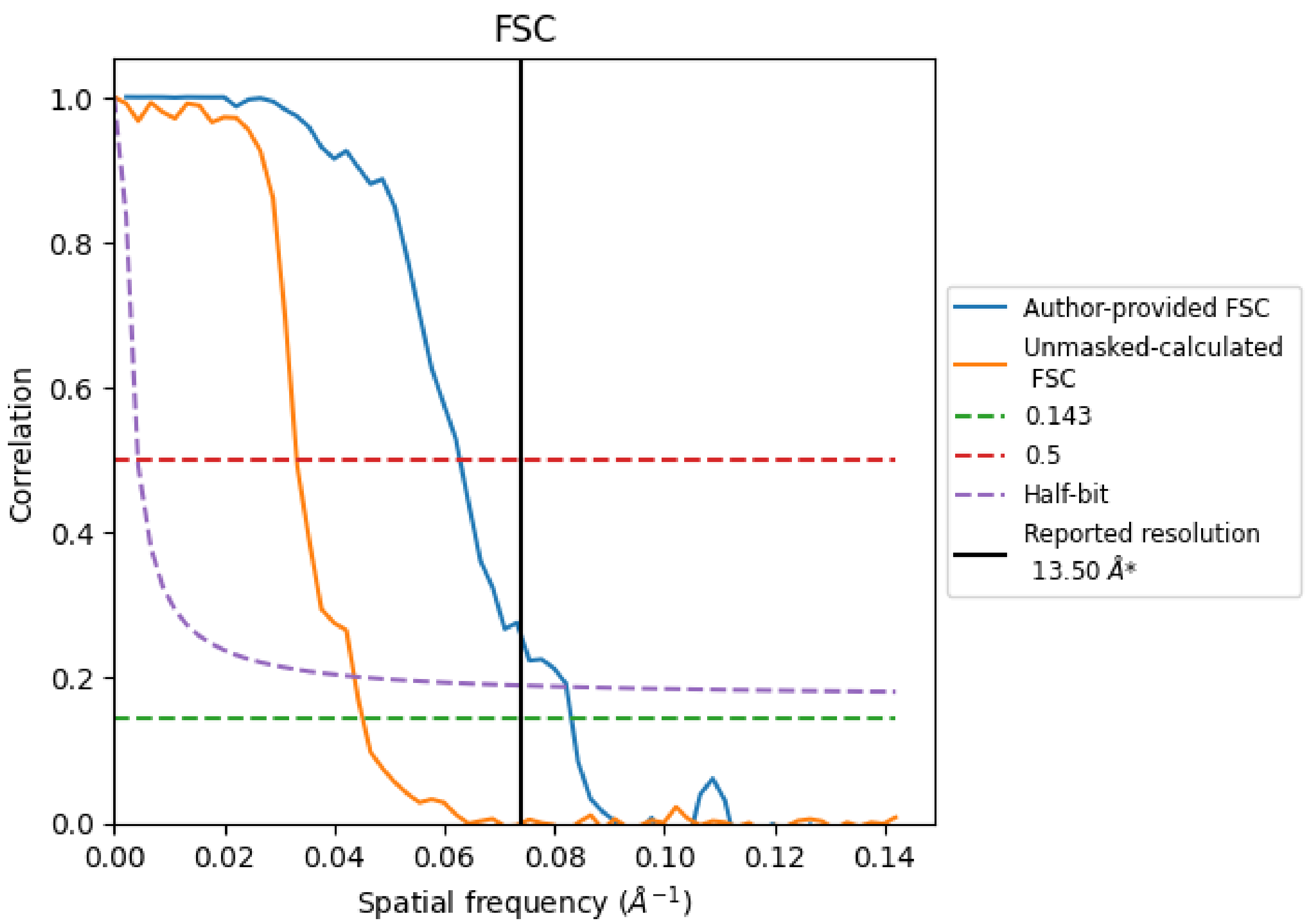

*Reported resolution corresponds to spatial frequency of 0.074 Å^{-1}

For Manuscript Review

8.2 Resolution estimates (i)

Resolution estimate (Å)	Estimation criterion (FSC cut-off)		
	0.143	0.5	Half-bit
Reported by author	13.50	-	-
Author-provided FSC curve	12.03	15.90	12.17
Unmasked-calculated*	22.12	30.12	22.94

*Resolution estimate based on FSC curve calculated by comparison of deposited half-maps. The value from author-provided FSC intersecting FSC 0.143 CUT-OFF 12.03 differs from the reported value 13.5 by more than 10 %

The value from deposited half-maps intersecting FSC 0.143 CUT-OFF 22.12 differs from the reported value 13.5 by more than 10 %

For Manuscript Review

9 Map-model fit (i)

This section contains information regarding the fit between EMDB map EMD-18737 and PDB model 8QXU. Per-residue inclusion information can be found in section 3 on page 10.

9.1 Map-model overlay (i)

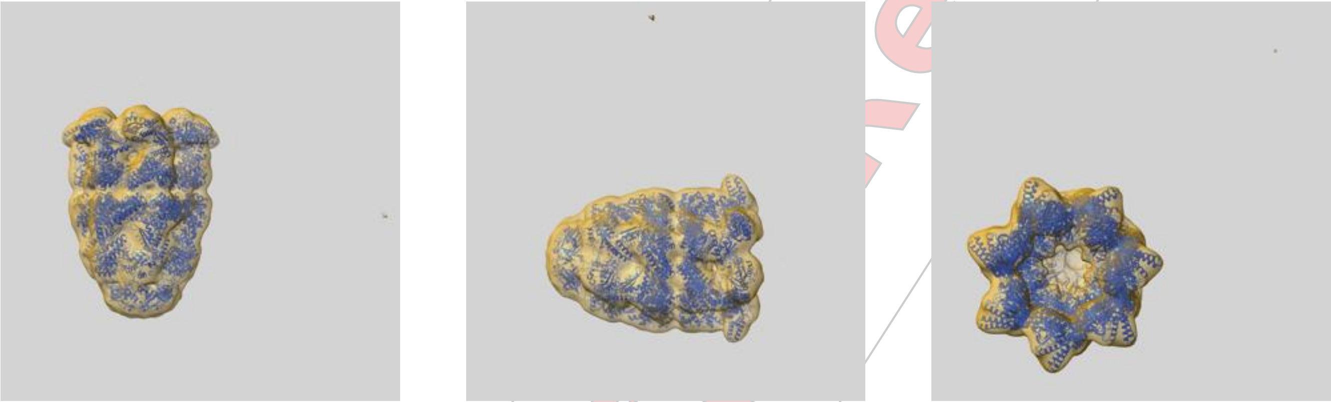

X Y Z

The images above show the 3D surface view of the map at the recommended contour level 0.0981 at 50% transparency in yellow overlaid with a ribbon representation of the model coloured in blue. These images allow for the visual assessment of the quality of fit between the atomic model and the map.

For Manuscript Review

9.2 Q-score mapped to coordinate model (i)

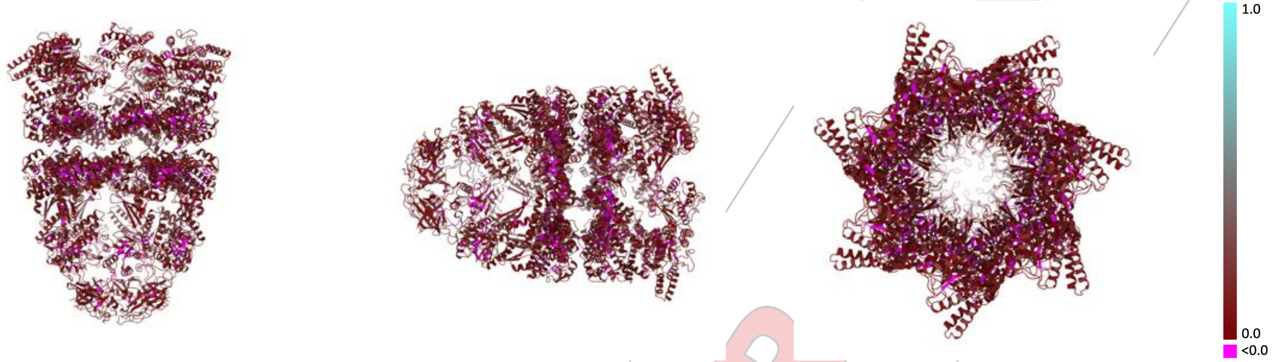

X Y Z

The images above show the model with each residue coloured according its Q-score. This shows their resolvability in the map with higher Q-score values reflecting better resolvability. Please note: Q-score is calculating the resolvability of atoms, and thus high values are only expected at resolutions at which atoms can be resolved. Low Q-score values may therefore be expected for many entries.

9.3 Atom inclusion mapped to coordinate model (i)

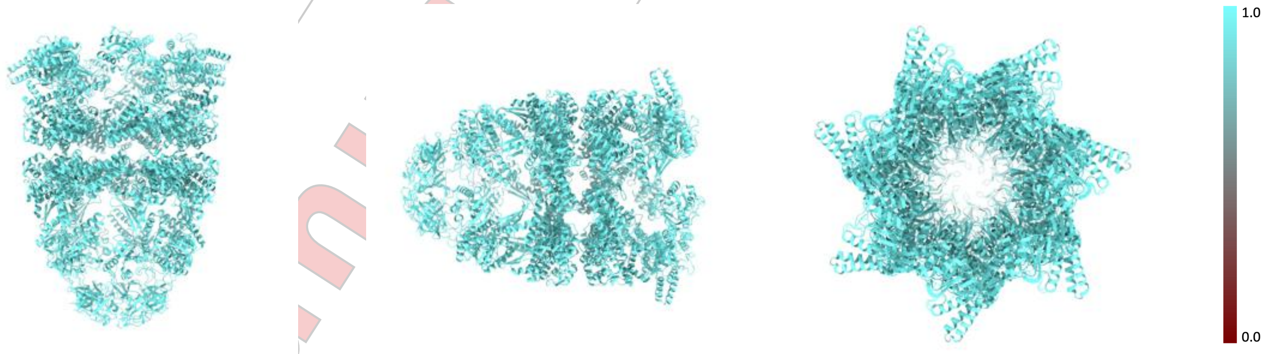

X Y Z

The images above show the model with each residue coloured according to its atom inclusion. This shows to what extent they are inside the map at the recommended contour level (0.0981).

For Manuscript Review

9.4 Atom inclusion (i)

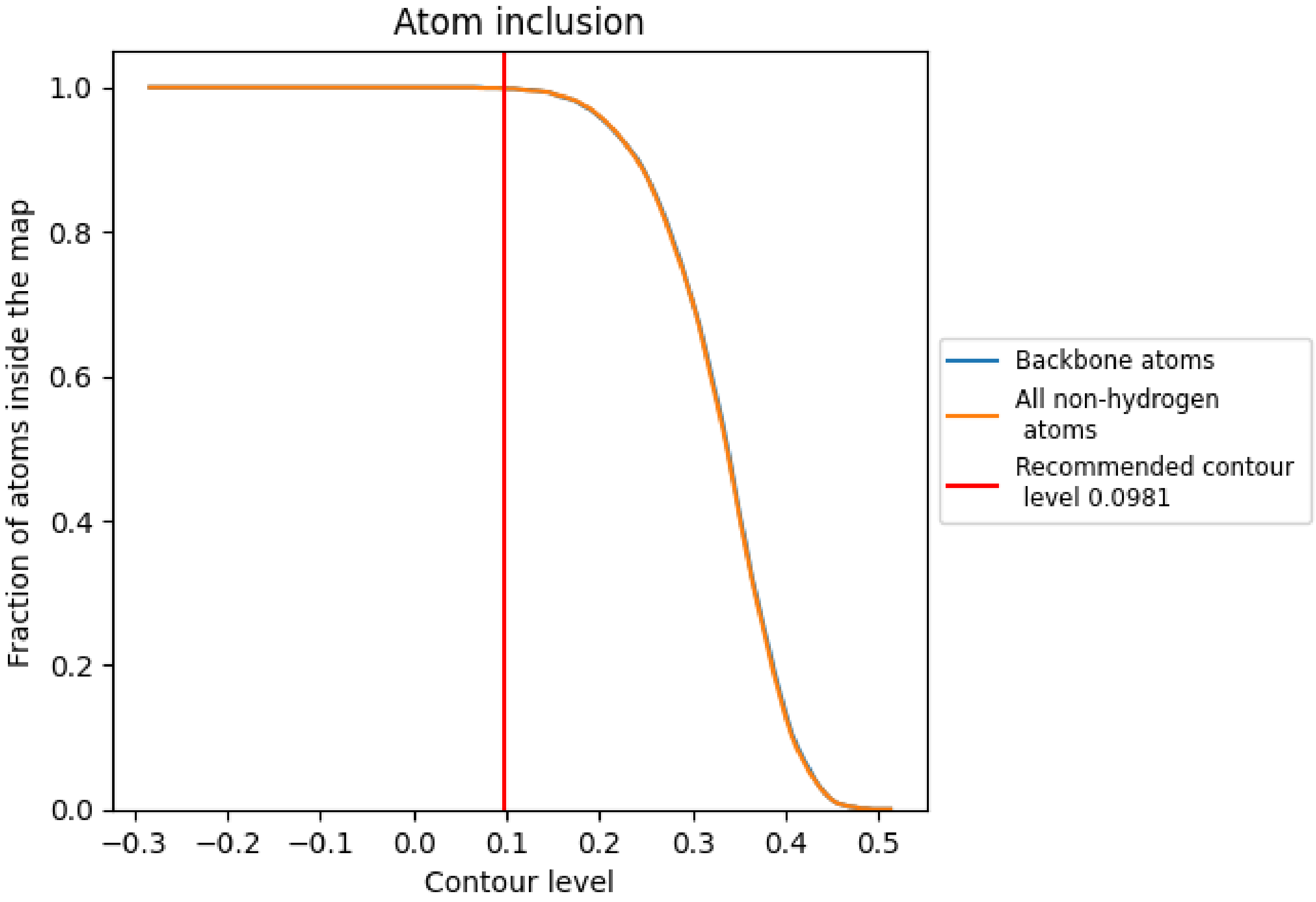

At the recommended contour level, 100% of all backbone atoms, 100% of all non-hydrogen atoms, are inside the map.

For Manuscript Review

9.5 Map-model fit summary (i)

The table lists the average atom inclusion at the recommended contour level (0.0981) and Q-score for the entire model and for each chain.

Chain	Atom inclusion	Q-score
All	0.9980	0.1040
A	1.0000	0.1080
B	1.0000	0.1060
C	1.0000	0.1070
D	1.0000	0.1040
E	1.0000	0.1040
F	1.0000	0.1040
G	1.0000	0.1070
H	0.9970	0.1010
I	0.9960	0.1000
J	0.9960	0.1030
K	0.9960	0.1010
L	0.9960	0.1000
M	0.9960	0.1020
N	0.9960	0.1040
O	1.0000	0.1090
P	1.0000	0.1080
Q	1.0000	0.1030
R	1.0000	0.1090
S	1.0000	0.1090
T	1.0000	0.1070
U	1.0000	0.1040

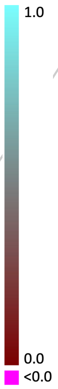

For Manuscript Review
